# *Tethya wilhelma* (Porifera) Is Highly Resistant to Radiation Exposure and Possibly Cancer

**DOI:** 10.3390/biology14020171

**Published:** 2025-02-07

**Authors:** Angelo Fortunato, Jake Taylor, Jonathan Scirone, Sareh Seyedi, Athena Aktipis, Carlo C. Maley

**Affiliations:** 1 Arizona Cancer Evolution Center, Arizona State University, Tempe, AZ 85287, USA; 2 Biodesign Center for Biocomputing, Security and Society, Arizona State University, Tempe, AZ 85287, USA; 3 School of Life Sciences, Arizona State University, Tempe, AZ 85287, USA; 4 Department of Theoretical and Applied Sciences, eCampus University, 22060 Novedrate, Italy; 5 Department of Psychology, Arizona State University, Tempe, AZ 85287, USA

**Keywords:** radio resistance, cancer prevention, DNA repair, sponges

## Abstract

There are no reports of cancer in sponges, despite them having somatic cell turnover, long lifespans, and no specialized adaptive immune cells. In order to investigate whether sponges are cancer resistant, we exposed a species of sponge, *Tethya wilhelma*, to X-rays. We found that *T. wilhelma* can withstand 518 Gy of X-ray radiation. That is approximately 100 times the lethal dose for humans. A single high dose of X-rays did not induce cancer in *T. wilhelma*, providing the first experimental evidence of cancer resistance in the phylum Porifera. Following X-ray exposure, we found an overexpression of genes involved in DNA repair, signaling transduction pathways, and epithelial-to-mesenchymal transition. *T. wilhelma* has the highest level of radiation resistance that has yet been observed in animals that have sustained somatic cell turnover. This may make them an excellent model system for studying cancer resistance and developing new approaches for cancer prevention and treatment.

## Simple Summary

Sponges are among the first animals that evolved on Earth. They are a very diverse group of animals made up of thousands of species. Despite being studied and used since antiquity, the development of tumors has never been described in sponges. Our study has highlighted that sponges are capable of tolerating a very high dose of radiation (equivalent to 60,000 human chest X-rays) never before observed in long-lived multicellular organisms (some sponge species can live hundreds or even thousands of years) with a sustained somatic cell turnover. During the observation period after X-ray exposure, we did not observe any changes in the sponges that suggested the development of tumors. This is the first experimental evidence that sponges are radioresistant, suggesting that the lack of evidence of tumor development in sponges may well be due to cancer prevention mechanisms. This discovery could allow for the identification of new mechanisms of DNA protection or repair, as well as the identification of molecules produced by sponges that could be the basis for the development of new drugs to protect against radiation and cancer.

## 1. Introduction

Since ancient times, sponges have attracted the interest of scholars. They are actively studied in different fields of biology such as ecology, developmental biology, and molecular biology [1].

Invertebrate organisms can develop cancer [2] and the presence of cancer cells can be recognized using the standard morphological and molecular criteria adopted in cancer biology research [3]. Despite the extensive studies on sponges and their ability to recognize cancer in invertebrate animals [3,4], to date, there have been no reports of cancer in sponges [2].

Invertebrates that branched off early in the evolution of animals, such as sponges, that lack immune specialized cells or only have primitive elements of an adaptive immune system [5,6] may lack the ability to detect and eliminate mutant cells. Sponges have a long lifespan and somatic cell turnover [7], which should make them susceptible to cancer because over the course of their lifespans, they would be expected to accumulate carcinogenic mutations. However, the fact that no cancer has been reported in sponges [2] suggests that they might have a physiology that is resilient to mutations or possess effective mechanisms for DNA damage prevention, DNA repair, and tissue homeostasis.

Sponges are an effective model system for studying cancer resistance.

**Figure 1:**
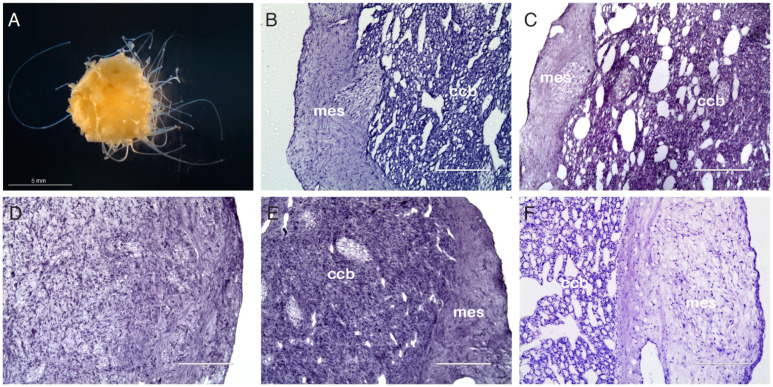
Histological analysis. *T. wilhelma* has a globular shape. It produces filamentous body extensions and is not pigmented. The largest specimens can reach the size of 15–20 mm in diameter (**A**). Histological cross-section of untreated *T. wilhelma* (**B**) after 24 h (**C**), 7 days (**D**), 21 days (**E**), and 838 days from X-ray exposure (**F**). Twenty-four hours after X-ray exposure, control sponges have anatomical features indistinguishable from untreated sponges (**B**) vs. (**C**). The normal anatomy is almost completely lost after 7 days, and cells show an undifferentiated phenotype (**D**). After 21 days, most cells still appear to have an undifferentiated phenotype, but anatomical reorganization has begun (**E**). After 838 days, the sponges appear to have a normal morphology (**F**). Hematoxylin and eosin stain; ccb = choanoderm; mes = peripheral mesohyl; ccb-mes boundary = mesohyl of the cortex–choanoderm boundary. (**B**–**F**) Scale bar = 100 μm. Image (**A**) was taken under a dissecting microscope using dark field illumination.

In order to investigate cancer resistance in sponges and evaluate this hypothesis, we studied the sponge *T. wilhelma*, which is a sessile, filter-feeding demosponge [8] (Figure 1A). Traditionally, sponges are described as lacking true tissues, but mounting molecular and morphological evidence suggests the presence of tissues in sponges, particularly in Demosponges [9,10,11].

Demosponges possess a canal system that is characterized by a highly complex network of chambers lined with choanocytes, which are flagellated cells that are specialized in creating a flow of water and capturing food particles [8]. Water enters from the pores located mainly on the lateral walls of sponges and is discarded through a large medial excurrent canal opening on the apex of sponges, the osculum [8]. *T. wilhelma* has a globular shape, and the largest specimens can reach a size of 15–20 mm in diameter (Figure 1A). *T. wilhelma* only reproduces asexually by budding in the laboratory [12]. These sponges are capable of contractile and slow locomotory behavior [8,13] and adapt well to being cultured in aquaria [8,13].

The genome sequence of *T. wilhelma* is available [14], and genomic analyses have shown that many molecular pathways (e.g., signal transduction mechanisms) are well conserved in sponges [14]. *T. wilhelma* has a remarkably long lifespan but sufficiently short generation time [13] to make it possible to rapidly obtain experimental results.

Here, we set out to investigate whether sponges are particularly cancer resistant, and, if so, what the mechanisms underlying this cancer resistance are. Through a combination of morphological observations and transcriptomics, we were able to describe changes in *Tethya wilhelma* (Demosponge) [8,13] after X-ray exposure and assess the organism level, cell level, and gene expression changes over time.

This is the first study of radiation resistance and cancer resistance in Porifera, the sister group of all animals [15]. Our experimental setting did not identify any neoplasms in the timespan of observation.

## 2. Materials and Methods

### 2.1. Lab Cultures

We built a sponge culture system that consists of a main saltwater aquarium (340 L) that develops an ecosystem (coral reef) capable of supporting the growth of the sponges. The sponges are grown in 3 smaller (30 L) culture aquariums connected to the main one, with controlled temperature (24 °C) and water flow. This setting allows easy access to the sponges, including the observation of the sponges under a microscope without having to remove them from the aquarium.

We fed the sponges with artificial plankton (Aquakultur Genzel GmbH, Freiberg, Germany) twice daily. In order to obtain fine (<25 μm) food particles assimilable by sponges, we homogenized the artificial plankton with an IKA T10 Basic Ultra Turrax homogenizer.

The culturing of the sponges started 3 years prior to this study, with 20 animals. The original specimens are still alive; thus, the lifespan of the sponges in our laboratory setting is >5 years.

### 2.2. DNA Damage by X-Ray Irradiation

We exposed young adult sponges (diameter > 5 mm) to X-rays utilizing an RS-2000 Biological System X-ray irradiator. We exposed the sponges to a single dose of 600 Gy for the experiments. Considering the X-ray absorbance of the 10 mm column of water above the animals during the X-ray irradiation, we estimated the actual X-ray exposure of specimens to be 13.7% lower, 518 Gy.

### 2.3. Experimental Settings

Based on preliminary morphological observations, we selected three time points: 24 h, 7 days, and 21 days after X-ray exposure for both histological and transcriptomic analyses (RNA-seq). We randomly selected 3 sponges for each time point plus 3 specimens of control for both histological and transcriptome analyses, for a total of 36 sponges. We treated 4 additional independent groups of sponges (*n* = 6, *n* = 5, *n* = 5, *n* = 5, a total of 21 sponges) and relative controls (5 sponges for each group, a total of 20 sponges) for long-term morphological observations. Each group was exposed to X-rays at different times.

### 2.4. Morphological Analysis

Sponges are partially translucent, but only superficial structures can be observed in vivo. However, their shape is regular, and any morphological changes are easily observable under a microscope without having to remove them from the aquarium because of the sponge’s culture setting. In order to recognize cancer-like features in sponges, we will focus on any morphological anomalies, such as a local overproliferation of cells or a change in pigmentation other than the acute effect of radiation. We measured the morphological changes in the animals in vivo using ImageJ software version 1.51 [16].

For histological examination, we fixed the specimens with Pampl’s fluid (formalin 11%, ethanol (95%) 27%, acetic acid (100%) 7%, and H_2_O 55%) for 24 h at 4 °C. Then, we dissolved the siliceous spicules that make up its skeleton by submerging the specimens in 4% hydrofluoric acid (MilliporeSigma, Burlington, MA, USA, cat. n. 1.00338) for an additional 24 h at 4 °C. Then, we followed standard histological protocols [10].

For transmission electron microscopy, we fixed specimens in 2.5% glutaraldehyde (Electron Microscopy Sciences, Hatfield, PA, USA, cat. n. 16020) in a 0.2 M Na-cacodylate sucrose buffer (pH 7.2; Electron Microscopy Sciences, cat. n. 12300) for 2.5 h at 4 °C. Then, we rinsed the specimens 3 times with a 0.2 cacodylate sucrose buffer for 45–60 min total, post-fixed them for 2 h in a 1% osmium tetroxide (Electron Microscopy Sciences, cat n. 19150) 0.2 cacodylate sucrose buffer, and washed them 1 time with the buffer and then 3 times with deionized water for 45–60 min total. We stained them en bloc with 1% aqueous uranyl acetate (Electron Microscopy Sciences, cat n. 22400) for 16 h at 4 °C. After washing the specimens 4 times with water for 45–60 min total, we dehydrated them with an ascending ethanol series up to 70% ethanol. Then, we desilicated the specimens with 4% hydrofluoric acid for 1 h at 4 °C. Afterwards, we washed the specimens in 70% ethanol, and we completed the dehydration with an ethanol series up to 100%. Then, we transferred the specimens to anhydrous propylene oxide (cat. n. 14300) for 30 min (replacing the anhydrous propylene oxide with fresh one after 15 min). We infiltrated samples with 5% Spurr’s epoxy resin (in anhydrous propylene oxide 3 h with rotation; 50% resin in anhydrous propylene oxide overnight with rotation (18 h); 75% resin in anhydrous propylene oxide with rotation (6 h); and 100% pure resin 3× for 24 h total (6 h, 12 h, 6 h). Finally, we flat embedded the specimens and polymerized them at 60 °C for 27 h. We used a diamond knife to cut ultrathin sections. We observed the sections under a Philips CM12 transmission electron microscope.

### 2.5. Quantification of DNA Damage

We quantified the DNA damage caused by X-ray exposure by the silver-stained Comet alkaline assay (Travigen^®^, Cat#4251-050-K) [17,18] according to the manufacturer’s specifications, and we used ImageJ software [16] to quantify the DNA fragmentation.

### 2.6. Molecular Genetic Analysis

We treated the *T. wilhelma* specimens with 518 Gy. After 24 h, 7 days, and 21 days following X-ray exposure, we extracted the total RNA (RNeasy^®^ mini kit, Qiagen, Hilden, Germany, cat. n. 74104) from 3 sponges for each treatment and control. After verifying the purity and integrity of the RNA using an Agilent 2200 TapeStation, Santa Clara, CA, USA, part of the extracted RNA (11.06 ng per sample on average) was utilized for RNA-seq analysis. We used the KAPA HyperPlus Library kit (Kapa Biosystems, Wilmington, MA, USA) to prepare the sequencing libraries. We performed 2 × 75 bp paired-end sequencing using an Illumina NextSeq 500 instrument.

We checked the quality of the RNA-seq reads for each sample using FastQC v0.10.1 (Table S1 in the Supplementary Materials), and we aligned the reads with the reference genome (NCBI, SRA, SRR2163223) using STAR v2.5.1b (22.68 million reads uniquely mapped on average per sample). Cufflinks v2.2.1 was used to report FPKM values (Fragments Per Kilobase of transcripts per Million mapped reads) and read counts. We uniquely mapped 17.05 million reads to the reference genome on average, per sample. We performed a differential expression analysis using the EdgeR package from Bioconductor v3.2 in R 3.2.3. For each pairwise comparison, genes with a false discovery rate (FDR) of < 0.05 were considered significant, and 2 log_2_-fold changes of expression between conditions were reported after Bonferroni correction. We analyzed the differentially expressed genes using BLAST [19], Ensembl [20], and their functional annotations, including fold enrichment (FE), using DAVID [21,22] and PANTHER [23] software and protein domains. We focused on the overexpressed genes because the decrease in gene expression can be a nonspecific effect of X-ray exposure due to cellular damage. We made available the full list of differentially expressed genes (Table S2 in the Supplementary Materials). BLAST [19] was used to find genes with a significant E value and S score as determined by the default BLAST settings. To verify, the Pfam domains of each gene in *T. Wilhelma* were compared to those of humans from the UniProt database that have a reviewed status. Human genes were denoted as homologs if they had at least one Pfam domain in common with the *T. Wilhelma* gene. The greatest number of Pfam domains match with the *T. Wilhelma* gene out of all other human genes and were found to have significant sequence similarity through BLAST.

### 2.7. Statistical Analysis

We performed statistical analyses with the IBM SPSS Version 25 statistics software package. Data were expressed as means ± standard deviation (S.D.). A value of *p* < 0.05 was considered statistically significant.

## 3. Results

We initially conducted a dose-finding experiment to quantify the sponges’ maximum tolerance to radiation. A total of 690 Gy is a sublethal dose for sponges (*n* = 5, 80% lethality). In contrast, all sponges (*n* = 7) exposed to 518 Gy suffered transitory morphological changes but survived. We did not observe any lethality or morphological changes in the same number of control sponges. We then conducted the subsequent experiments using a single dose of 518 Gy.

### 3.1. Morphological Observations

**Figure 2:**
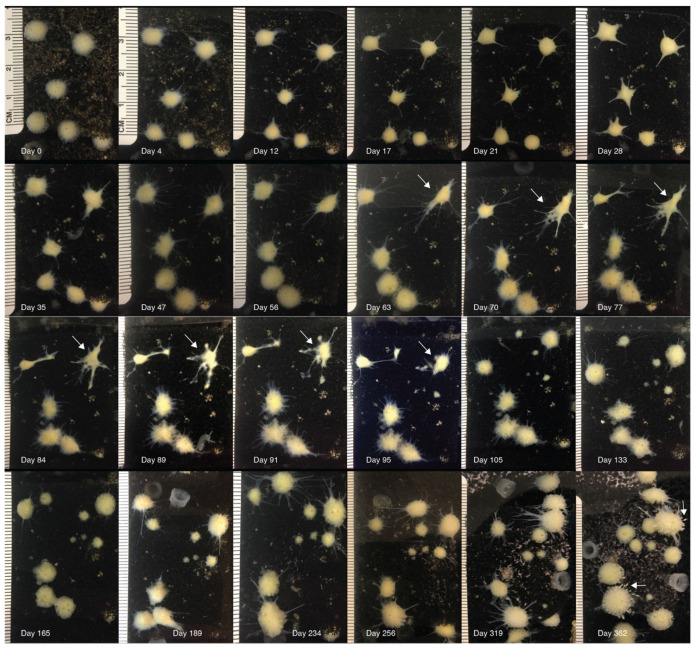
Long-term observation of sponges’ morphology. Images of a representative group (*n* = 6) of X-ray exposed (518 Gy) sponges. The first picture was taken starting before the exposure (day 0), and pictures were taken periodically until 362 days after the exposure. Initially (2–7 days), the sponges began to shrink, producing short and thin body extensions, and the pores and the osculum were no longer visible. Sponges reached their minimum size after 21–25 days, their surface became smooth, and they produced large and long body projections, causing the sponges to acquire an irregular star shape. Then, sponges reversed the shrinking process, but their morphologic features still appeared to be altered or progressed to further dissolvement (63–95 days, arrows indicate an example of the dramatic sponge morphological change; Figure S1 in Supplementary Materials); body extensions were either re-absorbed or broken off, generating new satellite sponges. Sponges gradually reacquired their original anatomical organization and appeared normal after ~180 days. After 362 days, the budding process through which sponges generate new offspring became visible (arrows). *T. wilhelma* sponges are capable of locomotion, and their position can slowly change over time.

We observed a general pattern in the morphological changes over time in sponges after X-ray exposure (Figure 2).

**Figure 3:**
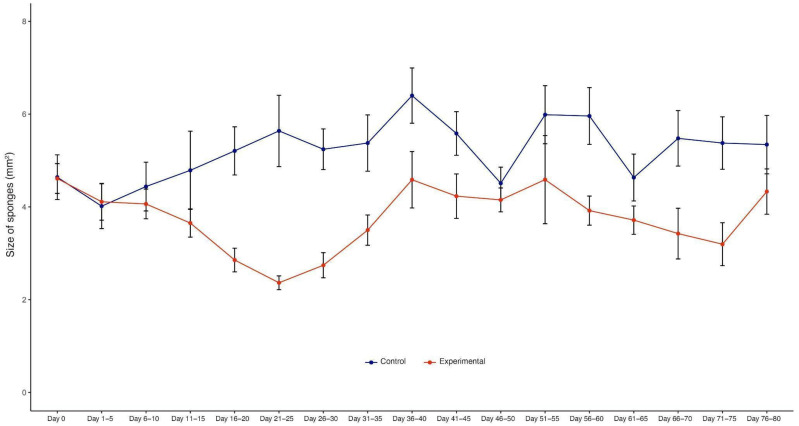
Sponges’ changes in size over time. The size of sponges decreased after X-ray treatment (*t*-test controls vs. X-ray-treated sponges, paired by time point, df = 16, t = 6.341, *p* < 0.0001). The blue line shows the control sponges (*n* = 10) and the red line shows the X-ray-treated sponges (*n* = 21). Sponges reached their smallest size after 21–25 days. Then, they gradually increased in size and recovered their morphological features. Graphs of individual points represent mean ± s.e.m. (error bars).

(a) Initially (2–7 days), the sponges begin to shrink, producing short and thin body extensions, and the pores and the osculum are no longer visible (Figure 2, day 4). Sponges reach their minimum size after 21–25 days (Figure 2); paired *t*-test, df = 16, t = 6.341, and *p* < 0.0001 (Figure 3). Their surface became smooth, and they produced large and long body projections, causing sponges to acquire an irregular star shape (Figure 2, day 28).

(b) Then, sponges reverse the shrinking process, but their morphologic features appear to be still altered or progress to further dissolvement (Figure 2, days 63–95). Body projections increased their surface, and additional body projections were generated (Figure 2 and Figure S1 in Supplementary Materials). Body extensions were either re-absorbed or broken off, generating new satellite sponges, observed in 33.3% of sponges (Figure 2, days 91–105).

(c) Sponges gradually reacquired their original anatomical organization and appeared normal after ~180 days. Four out of the twenty-one treated sponges died after an average of 162 (±30.9 S.D.) days. The sponges were kept in the laboratory for 3 years after the treatment. A total of 17 of the 21 sponges treated with 518 Gy are alive, do not show any atypical morphological changes, and are indistinguishable from untreated sponges and generate new offspring (Figure 2, day 362).

### 3.2. Histological Analysis

After X-ray exposure, sponges lose their typical anatomical organization [24] (Figure 1). The filtering structures of sponges (choanoderm) and the specialized water-flowing and feeding cells (choanocytes) are lost (Figure 1D,E), and the choanoderm appears to be filled with undifferentiated cells [25]. The histological analyses did not show necrotic areas at any time after X-ray exposure (Figure 1C–F).

**Figure 4:**
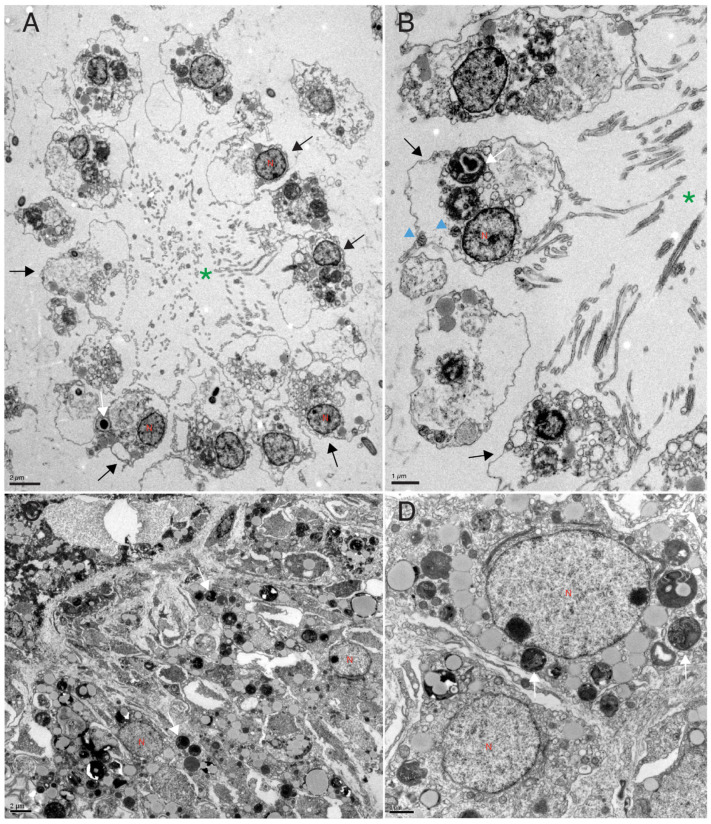
Transmission electron microscopy analysis. TEM analysis of control (**A**,**B**) and X-ray exposed sponges (**C**,**D**) 7 days after exposure. The choanoderm of control sponges is well organized with choanocyte chambers (green *). Instead, the choanoderm of treated sponges (**C**,**D**) is disorganized, and the choanocyte chambers are absent. The nuclei appear to be intact and have different shapes. Black arrows = choanocytes, blue head arrow = mitochondria, N = nucleus, white arrows = bacteria.

Electron microscopy analysis (TEM) of the choanoderm after 7 days from X-ray exposure showed that the choanoderm of treated sponges is disorganized and the choanocyte chambers are deeply altered or absent. The choanocytes are no longer recognizable (Figure 4).

### 3.3. DNA Damage Analysis

DNA fragmentation analysis (Comet assay) showed limited DNA degradation immediately after a submaximal (518 Gy) X-ray exposure (DNA fragmentation, treated: 8.23% ± 16.32 S.D., controls 1.34% ± 6.99 S.D., not statistically significant (Figure S2 in Supplementary Materials)).

### 3.4. Gene Expression Analysis

**Figure 5:**
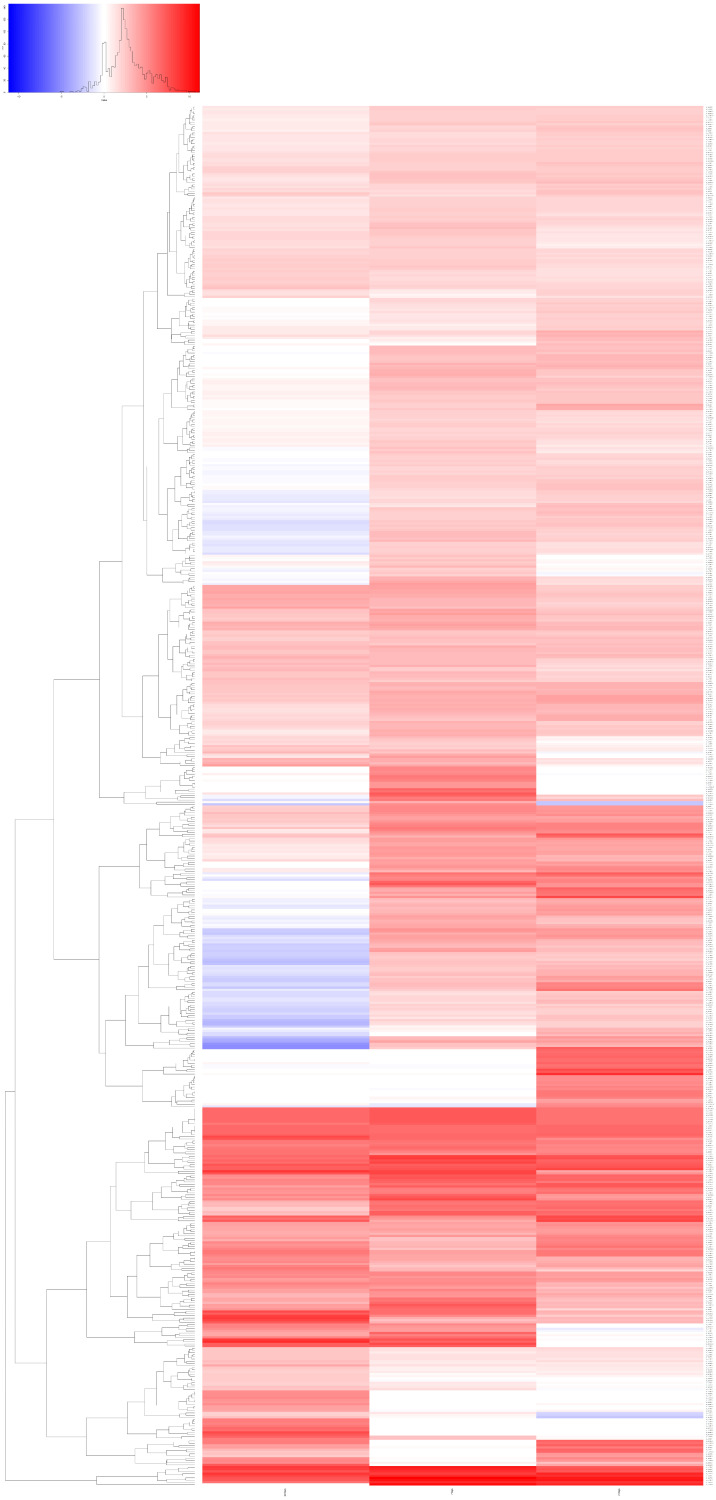
Heat map diagram. Heat map diagram showing the genes that were overexpressed at the 3 different time points (24 h, 7 and 21 days) after X-ray treatment. There are genes that are overexpressed only at certain time points and genes that are only overexpressed at 2 or 3 time points.

**Figure 6:**
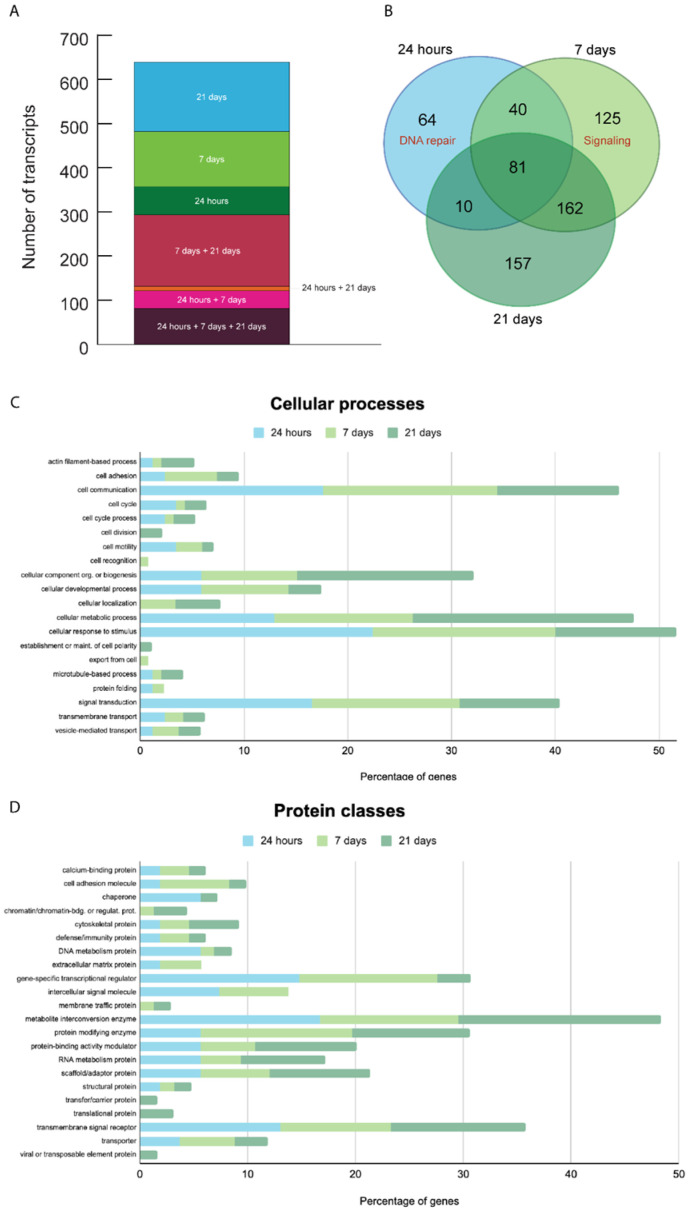
Gene expression analysis. The histogram (**A**) and Venn diagram (**B**) show the number of genes that were overexpressed at the 3 different time points (24 h, 7 and 21 days) after X-ray treatment. There are genes overexpressed only at a specific time point and genes overexpressed at 2 or 3 time points. The number of transcripts detected increased from 24 h to 21 days, and 24 h and 21 days exclusively share only 1.6% of transcripts; on the contrary, 7 days and 21 days have the largest percentage (19.8%) of exclusively overlapping transcripts. Genes can be activated at different times and for different lengths of time after X-ray exposure. The Venn diagram shows the exact number of transcripts expressed. The functional analysis highlights the cellular processes (**C**) and the protein classes (**D**) overexpressed with PANTHER software. ECM = extracellular matrix.

We performed a transcriptome analysis (RNA-seq) of three *T. wilhelma* specimens collected at three different time points (24 h, 7 and 21 days) after X-ray treatment. We found a total of 639 overexpressed transcripts in the three experimental time points compared to untreated sponges at the same time points (Figure 5). Each group had different gene expression levels and differed from the controls (Figure S3 Supplementary Materials). Many of the expressed genes have a human homolog (given in parentheses). There are genes overexpressed only at a specific time point (24 h, 7 or 21 days) and genes overexpressed at two or three time points (Figure 5 and Figure 6, Tables S3 and S4 in Supplementary Materials).

Genes overexpressed 24 h after X-ray exposure. We found 195 transcripts overexpressed 24 h after X-ray exposure, of which 97 have a human homolog (Figure 5 and Figure 6 and Table S3 in Supplementary Materials). A total of 64 of those 195 transcripts were only overexpressed after 24 h but not at later time points. Forty-four of those sixty-four overexpressed transcripts specific to the 24 h time point have a human homolog. We detected an enrichment of human homolog genes involved in DNA repair (FE = 10.3, FDR = 0.037), such as twi_ss.22376.1 (*LIG3*), DNA ligase, and twi_ss.21448.1 (*MRE11*), which are involved in the double-strand break repair mechanism, twi_ss.11375.1 (*PCNA*), the proliferating cell nuclear antigen that has a key role in DNA damage response, twi_ss.28211.1 (*RPA1*), replication protein A1 that is a cofactor of DNA polymerase delta involved in the RAD6-dependent DNA repair pathway, Twi_ss.25822.1-3 (*GINS4*), GINS subunit, domain A, which is involved in double-strand break repair via break-induced replication.

We found that the twi_ss.17326.7 gene (*CUBN*), cubilin, is the most differentially expressed gene (logFC = 8.55) specific to the 24 h time point after X-ray exposure. *CUBN* is an endocytic receptor expressed in the epithelium of the intestines and kidneys [26] and is downregulated in renal cell carcinoma [27].

In addition, we identified the overexpression of genes involved in stress response, such as twi_ss.28284.1 (*HSPA1A*), the heat shock protein family A (Hsp70) member 1A.

Genes overexpressed 7 days after X-ray exposure. We found 408 transcripts overexpressed, of which 141 have a human homolog (Figure 5 and Figure 6 and Table S3 in Supplementary Materials). Among them, 125 transcripts were specifically overexpressed after 7 days, of which 53 have a human homolog. We detected an enrichment of ankyrin-containing domain genes (FE = 19, FDR < 0.001), signal proteins (FE = 2.4, FDR = 0.005), and extracellular matrix remodeling genes (FE = 68.1, FDR = 0.03).

We identified genes specifically overexpressed after 7 days involved in DNA repair, such as twi_ss.10792.1 (*FAN1*), twi_ss.27976.3 (*KAT5*), and twi_ss.19635.1 (*TRIP12*), and, importantly, we found a correlation between the morphological changes observed 7 days after X-ray exposure and the function of genes overexpressed at the same time. For instance, we found the overexpression of genes involved in development, adult tissue homeostasis, and mesenchymal transition, such as twi_ss.2267.1 (*NOTCH1*), twi_ss.5628.6 (*MET*) MET proto-oncogene, receptor tyrosine kinase, twi_ss.6204.1 (*POSTN*), and periostin. We also found genes involved in embryonic stem cell regulation, such as twi_ss.7082.1 (*ETV4*), twi_ss.4355.1 (*TRIM71*), and twi_ss.329.1 (*ZFP36L1*), together with a variety of genes, such as twi_ss.26378.1 (*COL6A3*) collagen type VI alpha 3 chain, twi_ss.31853a.1 (*COL6A6*) collagen type VI alpha 6 chain, twi_ss.25970.2 (*LTBP1*) latent transforming growth factor beta binding protein 1, twi_ss.20516a.5 (*SORL1*) sortilin-related receptor 1, twi_ss.2619.7 (*VWF*) von Willebrand factor, twi_ss.19885.1 (*NRXN3*) neurexin 3, and twi_ss.1244.5 (*ADGRE5*) adhesion G protein-coupled receptor E5, involved with the epithelial-mesenchymal transition.

Genes overexpressed 21 days after X-ray exposure. We found 410 overexpressed transcripts, of which 150 of these have a human homolog (Figure 5 and Figure 6 and Table S3 in Supplementary Materials). We identified 157 transcripts specifically overexpressed after 21 days, of which 72 have a human homolog. There is no functional signature specific to the genes expressed specifically after 21 days. We found the following genes involved in DNA double-strand break repair: twi_ss.10068.1 (*PARP3*) poly(ADP-ribose) polymerase family member 3 and twi_ss.28718.1 (*PARPF19*) PHD finger protein 19. We found the following gene involved in DNA repair: twi_ss.7757.3 (*UBR5*) ubiquitin protein ligase E3 component n-recognin 5.

Genes overexpressed 24 h, 7 and 21 days after X-ray exposure. We found 81 transcripts overexpressed at all three time points, of which 34 have a human homolog (Figure 5 and Figure 6 and Table S3 in Supplementary Materials). For instance, Twi_ss.16656.2 (*PHF8*) and PHD finger protein 8 are one of the most differentially expressed genes (9.3 ± 1.8 S.D. logFC). The *C. elegans* homolog promotes DNA repair via homologous recombination [28]. Twi_ss.4977.9 (8.9 ± 0.4 S.D. logFC) is a homolog of the human gene Spatacsin (*SPG11*). The function of *SPG11* is not well understood. It has a role in a form of spastic paraplegia, a neurodegenerative disorder, and it appears to be also involved in DNA repair [29].

Genes overexpressed 24 h and 7 days after X-ray exposure. We found 40 transcripts overexpressed at both time points, of which 19 of these genes have a human homolog (Figure 5 and Figure 6 and Table S3 in Supplementary Materials). The most differentially expressed gene (8 ± 1.8 S.D. logFC) is twi_ss.12458.1 (*TTN*), a gene unknown to be activated after X-ray exposure.

Genes overexpressed 24 h and 21 days after X-ray exposure. We found only 10 overexpressed transcripts, of which five of these genes have a human homolog gene (Figure 5 and Figure 6 and Table S3 in Supplementary Materials). With only five genes with known homolog functions, there were no statistically significantly enriched pathways. However, twi_ss.19378.2 (*ERCC1*) is involved in DNA repair.

Genes overexpressed 7 days and 21 days after X-ray exposure. We found 162 overexpressed transcripts, of which 45 of these have a human homolog gene (Figure 5 and Figure 6 and Table S3 in Supplementary Materials). Overall, there is an enrichment of genes involved in extracellular matrix organization, such as fibronectin (FE = 15.7, FDR < 0.0001), with an EGF-like domain (FE = 19.7, FDR < 0.0001), and signal peptides (FE = 3.3, FDR < 0.0001). One of the most overexpressed genes (10.1 ± 0.7 S.D. logFC) is twi_ss.21105.9 (*SCUBE1*) signal peptide, CUB, and EGF-like domain-containing protein 1, which may function as an adhesive molecule [30].

## 4. Discussion

*T. wilhelma* sponges can withstand 518 Gy of X-ray radiation. That is approximately 60 times the lethal dose for mice [31,32] and 100 times the lethal dose for humans [33]. This amount of radiation should shatter the sponges’ DNA; however, the Comet assay suggests this does not happen in *T. wilhelma*.

Early organisms evolved in an environment with higher levels of background radiation [34]. Despite the fact that water partially shields aquatic organisms from direct radiation exposure, radionuclides can accumulate in the sea. As filter-feeding animals, sponges could be particularly exposed to the accumulation of radionuclides and other toxic agents [35]. Moreover, sponges are sessile organisms without a nervous system and are thus not capable of rapidly escaping if the concentration of radioactive or other toxic agents increases. For these reasons, sponges are considered biological indicators of environmental pollution, such as radionuclides [36,37].

There are examples of extreme radioresistance in bacteria [38] and multicellular organisms capable of anhydrobiosis (desiccation), such as rotifers [39] and tardigrades [40,41], which are unlikely to apply to *T. wilhelma*. Tolerance for both desiccation and radiation may originate from similar DNA protective or repair mechanisms [39,42]. Indeed, desiccation causes DNA breakage [39,41], similar to damage induced by radiation, which may be repaired upon rehydration [39]. Tardigrades possess molecular mechanisms to prevent DNA damage. Dsup is a tardigrade-specific nucleosome-binding protein that protects chromatin from hydroxyl radicals and contributes to the organism’s radio tolerance [43]. The combination of DNA protective and DNA repair mechanisms determines the level of radioresistance of these organisms. Importantly, rotifers and tardigrades have no or highly restricted somatic cell turnover [35,40], and the species tested for radioresistance have a short (~60 days) lifespan [40,44], which prevents mutant clones and accumulating further mutations, leading to cancer. In these conditions, the DNA damage that occurs does not propagate and so might not be apparent [45]. Thus, there is little chance for cancer to develop in tardigrades.

*Trichoplax adhaerens* (Placozoa) are resistant to X-ray exposure (143.6 Gy induce limited mortality) but cause massive DNA fragmentation [46]. In a similar experimental setting, a 332.5 Gy dose is lethal for *T. adhaerens* after 7 days [46]. Ten Gy of exposure is lethal for *Mnemiopsis leidyi* (Ctenophora) after 24 h (personal observation). *T. wilhelma*, on the other hand, can tolerate a single dose of 518 Gy with minimal DNA fragmentation, suggesting a different and more effective mechanism of radiation resistance. However, the DNA fragmentation analysis and the mechanisms of DNA protection or repair need to be investigated further.

In order to investigate the long-term effect of DNA damage and cancer in invertebrates, somatic cell turnover and a long lifespan should be an essential feature of any experimental organism. Sponges have somatic cell turnover [7] and a remarkably long lifespan: the largest *Xestospongia muta* specimen described on Caribbean reefs is estimated to be more than 2300 years old [47], a specimen of the sponge *Monorhaphis chuni* is thought to be 11,000 years old [48], and radiocarbon dating of the sponge *Rossella racovitzae racovitzae* determined that it was around 440 years old [49]. Selection for this long lifespan may have also been selected for cancer suppression mechanisms.

Considering all these factors, somatic cell turnover, a long life span, and a primitive immune system, we would expect the development of tumors in sponges, but there have been no reports of cancer in the entire Porifera phylum [2] (with 8500 described living species [50]).

In our experimental setting (single high dose), radiation did not induce cancer development in *T. wilhelma* during the 3 years following X-ray exposure. Malignant cancer risk in humans is estimated to be 8% per Gy [51]. A small fraction of the dose to which the sponges have been subjected would generally have given rise to cancer in mice [52] and humans [51].

Additional studies will be necessary to establish if *T. wilhelma* is resistant to different types of radiation exposure, such as repeated exposure to identical or larger doses of radiation, as well as different mutagenic agents.

The limited DNA fragmentation observed in the Comet assay at such high levels of radiation suggests that sponges must have some way of protecting their DNA. It is not expected that a gene expression analysis could be capable of detecting such a mechanism.

It cannot be ruled out that the sponges may have an extremely rapid DNA repair phenotype that acts before the Comet readout because the samples were processed immediately, though this seems unlikely. In either case, the upregulation of DNA repair mechanisms at 24 h, 7 days, and 21 days suggests that there was at least some damage to the DNA that either overpowered the protective mechanisms or remained after extensive repair. Independently of what mechanism renders sponges radioresistant, its identification and molecular characterization would represent an interesting discovery with potential biomedical applications. These findings are the first step in this direction.

The molecular data and morphological observations suggest that sponges protect their DNA from damage in the first place and then activate mechanisms of DNA repair and cell death as they go through a complex phase of tissue reorganization. Finally, they rebuild their tissues’ original features. Our findings suggest that cancer resistance in sponges might be linked to their radioresistance.

Genes involved in DNA repair are activated at different times and for different lengths of time, providing insight into the temporal activation of these genes during the DNA repair process (Figure 5 and Figure 6 and Table S3 in Supplementary Materials). We found the overexpression of genes known to have a role in DNA double-strand break repair (Table S3 in Supplementary Materials), confirming that our experimental setting is capable of inducing the typical DNA damage produced by X-ray exposure and that sponges respond to radiation by upregulating DNA repair. As expected, we observed a higher number of genes involved in DNA repair 24 h and 7 days after X-ray exposure, but we also identified genes involved in this process specifically expressed 21 days after X-ray exposure. This observation suggests that after 21 days, the sponges are still actively repairing the DNA damage induced by radiation. Treated sponges overexpressed only 81 transcripts (12.7% of all overexpressed transcripts) in all three time points but overexpressed 64 transcripts only at 24 h, 125 transcripts only at 7 days, and 157 transcripts only at 21 days, suggesting waves of sequential transcriptional events.

We found overexpressed genes previously not linked to X-ray-induced damage or with unknown functions in humans (Table S3 in Supplementary Materials). For example, twi_ss.17326.7 (*CUBN*) is cubilin in humans. CUBN is an endocytic receptor expressed in the epithelium of the intestines and kidneys [26]. Interestingly, a low expression of *CUBN* in renal cell carcinoma is significantly associated with early disease progression and poor patient outcomes [27]. We hypothesize that the *CUBN* gene has a protective function against DNA damage or is involved in DNA repair and, thus, its downregulation in kidney cancer increases the chance of an aggressive evolution of the disease.

Epigenetic changes, such as methylation, are induced by X-ray exposure and contribute to regulating the cell response to stress [53]. Though we did not directly measure methylation, we detected the overexpression of twi_ss.21704.3 (*NSUN7*), a gene involved in methylation. Methylation induced by radiation can be a persistent epigenetic change after radiation [53], regulating gene activity long after expression normalization.

Future experiments should test if the overexpression of *CUBN* and *NSUN7* generates a radiation resistance phenotype and perhaps even a cancer resistance phenotype in cell lines and mice.

In addition to mechanisms of DNA repair, we detected the overexpression of genes involved in apoptosis and cell death. For example, Twi_ss.31154.2 (*NOX5*), NADPH oxidase-generated ROS, a gene involved in heavy ion irradiation-induced cell death [53] suggest the activation mechanisms of cell death, presumably to remove cells too compromised by radiation.

The gene function of the detected genes was inferred using only bioinformatic tools and needed to be experimentally validated in sponges.

Notably, we observed a distinct pattern in the morphological changes in the treated sponges. Seven days after X-ray exposure, sponges lose their typical anatomical organization, and the choanocytes that represent the most abundant cellular type of the choanoderm are undetectable, at least in their filtering specialized morphology with cilia and flagella. Choanocytes are one of the most specialized types of sponge cells and are capable of transdifferentiating into archaeocytes (stem cells) and serve the same function [54,55]. In transcriptomic analysis 7 days following X-ray exposure, we found that the overexpression of genes known to be involved in cell adhesion, signaling, embryonic cell regulation, and epithelial–mesenchymal transition (EMT), such as *NOTCH1* and *MET*, was consistent with our observations of changes in sponge morphology.

Studying radiation resistance in sponges might help improve radiation therapy. Ionizing radiation is part of the natural environment and evolution of organisms. Thus, the study of radiation responses in sponges could contribute to understanding the molecular evolution of cell radioresistance.

Over the course of a human lifetime, cells can evolve mechanisms that allow them to endure the effects of X-ray exposure [56] through somatic evolution. In human cancers, radiotherapy often induces EMT and leads to increased radioresistance accompanied by increased cell migration and invasion [57,58,59].

The finding that sponges react to X-ray exposure by increasing the number of undifferentiated cells suggests that EMT is not a cancer cell-specific response to radiotherapy but rather a natural cellular response to radiation both in organisms and human cells [59]. Sponges are particularly suitable for studying this process because their simple tissue organization facilitates the identification and analysis of tissue alterations. It is important to note that cancer cells do not utilize de novo cancer-specific mechanisms to survive radiation exposure but rather employ a biological process that is shared with sponges and has been evolutionarily conserved in our genomes for hundreds of millions of years. Understanding this biological process in response to radiotherapy in sponges could provide the basis for new treatment and cancer prevention strategies.

## 5. Conclusions

The study of the evolution of anti-cancer mechanisms, such as radiation resistance, can contribute to the understanding of multicellular organisms more generally, as the evolution of multicellularity itself depends on the multicellular organisms’ ability to prevent somatic cells from proliferating out of control. Our work suggests that sponges may be particularly resistant to cancer because of their radiation resistance and shows that sponges are a viable model system for studying anti-cancer mechanisms and radiation resistance.

## Data Availability

The datasets generated and analyzed during the current study are available from the corresponding author upon reasonable request. The sequencing data that support the findings of this study are openly available. The BioProject accession number is PRJNA800533, ID 800533.

## Supplementary Materials

The following supporting information can be downloaded at: https://www.mdpi.com/article/10.3390/biology14020171/s1, Figure S1: Magnification of 2 sponges with a dramatic morphological change, 91 days after X-ray exposure; Figure S2: The X-ray treatment produces little DNA fragmentation as measured by the Comet assay; Figure S3: Multidimensional scaling (MDS) plot; Table S1: FastQC report; Table S2: Differential expressed genes including the downregulated genes; Table S3: List of genes overexpressed 24 h, 7 and 21 days after X-ray exposure; Table S4: DAVID functional annotation clustering
