# Supplementary material for: Tethya wilhelma (Porifera) Is Highly Resistant to Radiation Exposure and Possibly Cancer
Source: Biology (Basel). 2025 Feb 7;14(2):171. doi: 10.3390/biology14020171 (PMC11851485; doi:10.3390/biology14020171)
Supplement: Supplementary file 1 [file biology-14-00171-s001.zip › biology-3415432-supplementary.pdf]

Supplementary figures and tables.

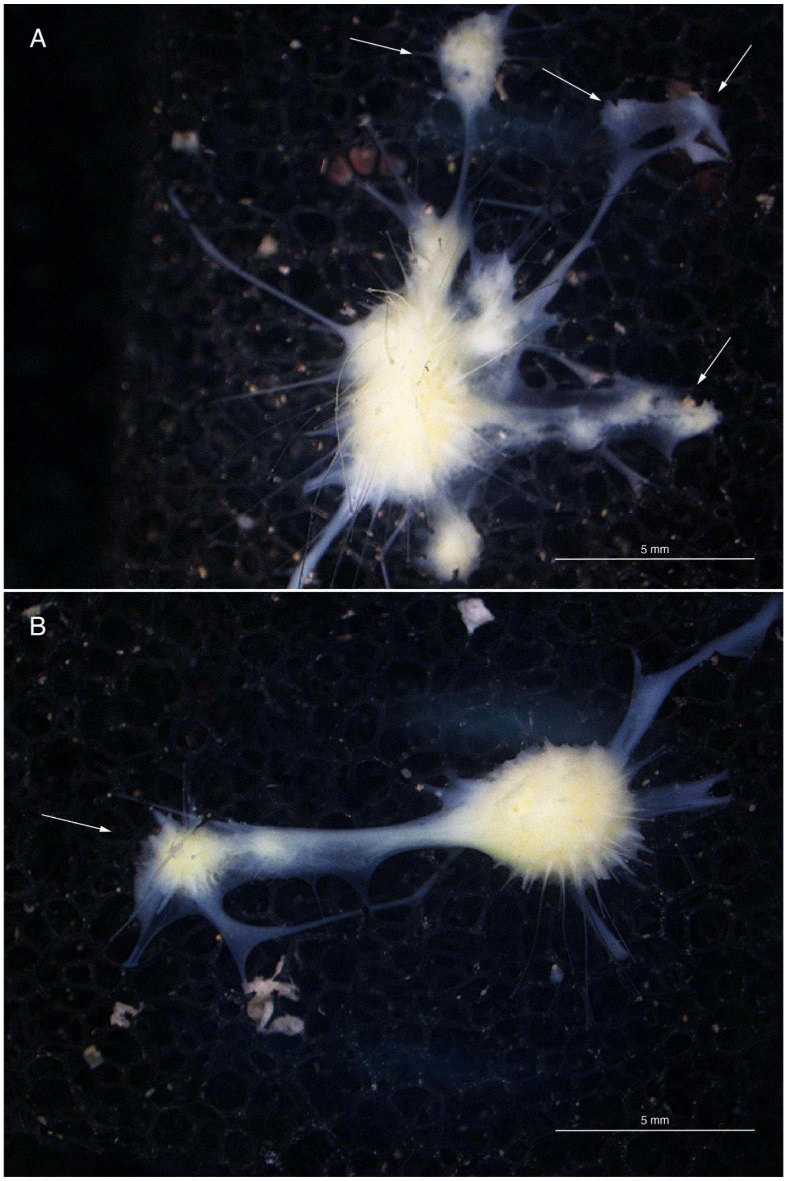

Figure S1. Magnification of 2 sponges (A, B) with a dramatic morphological change, 91 days after X-ray exposure (See figure 2). *T. wilhelma* can develop extensive body projections. The body projections either generate new satellite sponges (arrows) or they are reabsorbed.

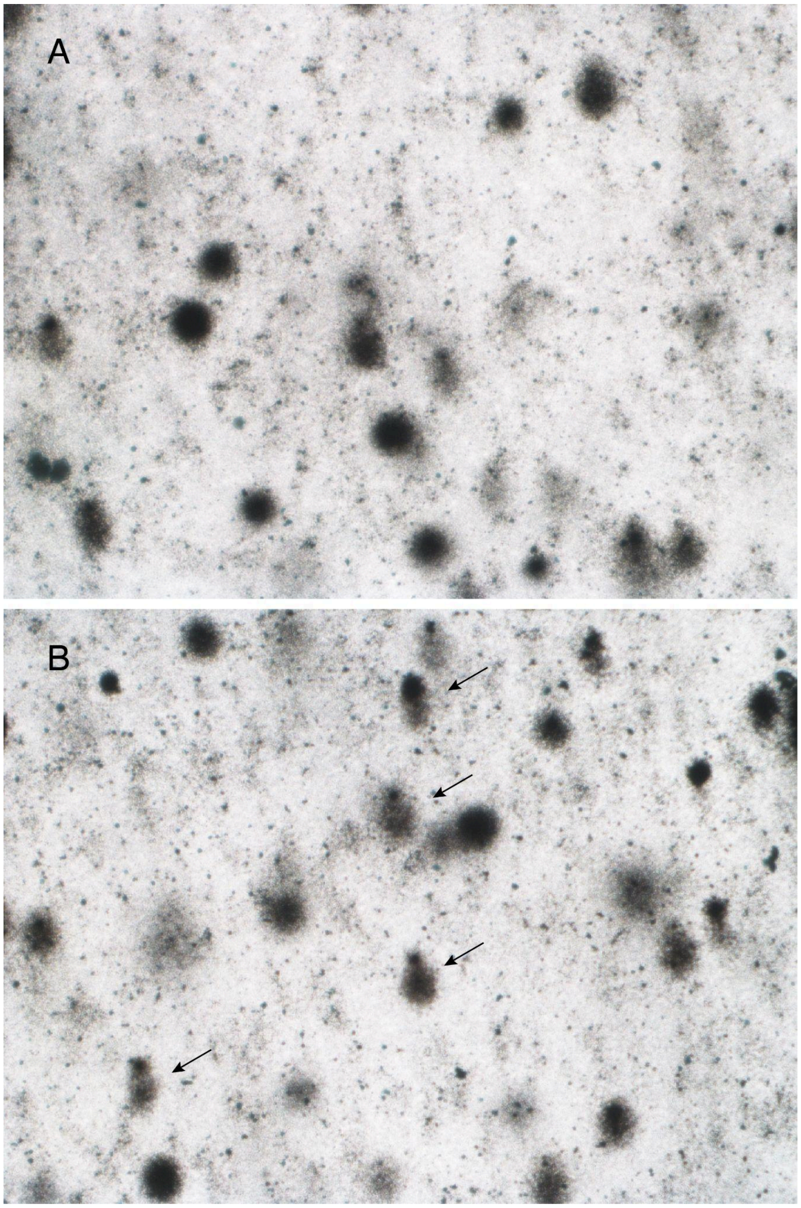

Figure S2. The X-ray treatment produces little DNA fragmentation as measured by the Comet assay. There is evidence of DNA fragmentation in: 8.23% ± 16.32 S.D. of treated sponges, and 1.34% ± 6.99 S.D. controls. Representative images of control (A) and X-ray treated (B) sponge nuclei, arrows indicate examples of partially fragmented nuclei (comets).

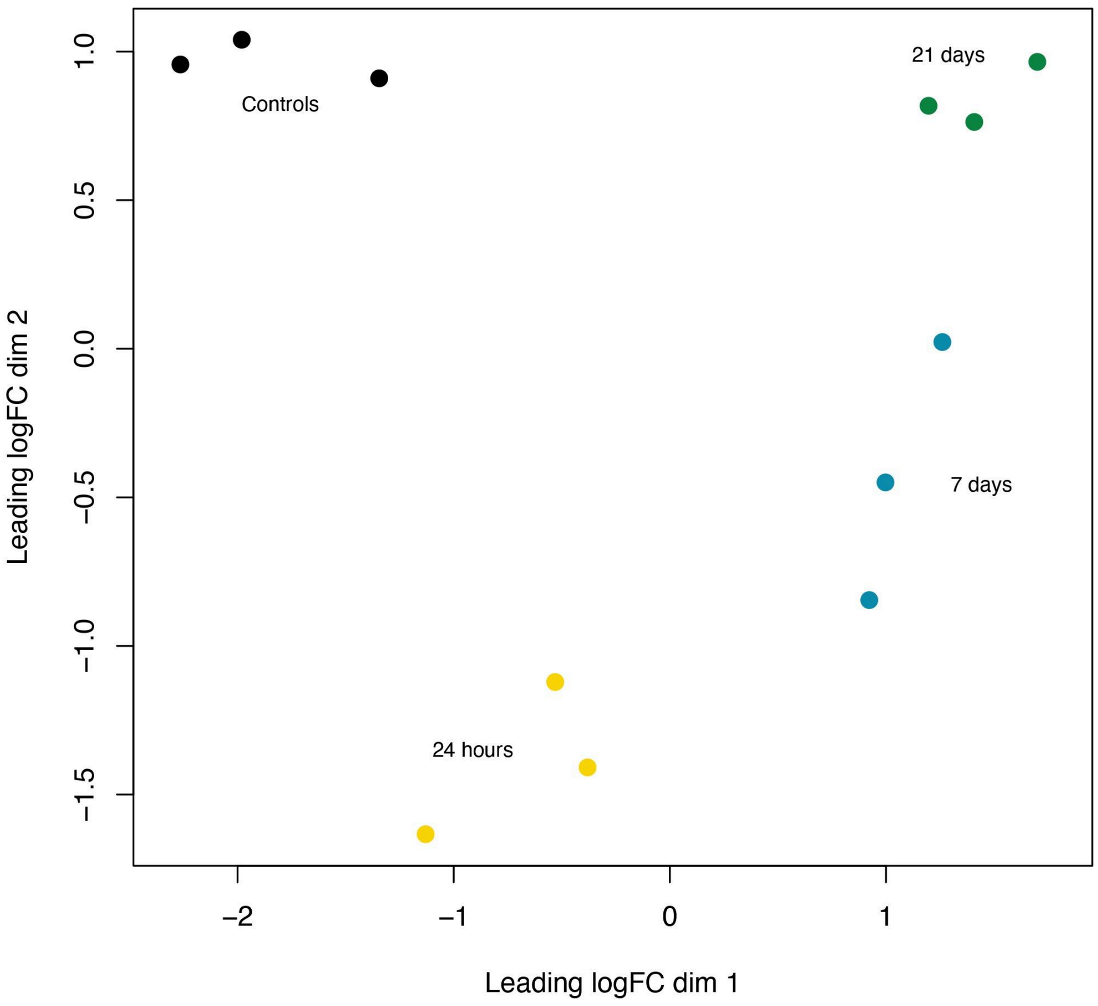

Figure S3. Multidimensional scaling (MDS) plot. There is a gene expression similarity between the samples collected at the same time point and a gene expression distance between the groups: controls, 24 hours, 7 and 21 days.

Table S1: FastQC report						
**Sample**	**% duplicates**	**% GC**	**Avegerage sequence length**	**Median sequence length**	**% fails**	**Total sequences**
C1_S1_SME_L001_R1_001	93.45	51	74.15	76	27.27	29054226
C1_S1_SME_L001_R2_001	91.25	51	74.19	76	18.18	29054226
C4_S2_SME_L001_R1_001	93.48	50	75.05	76	18.18	26224051
C4_S2_SME_L001_R2_001	91.42	49	75.08	76	27.27	26224051
C9_S3_SME_L001_R1_001	94.04	50	75.20	76	27.27	32664788
C9_S3_SME_L001_R2_001	91.96	49	75.23	76	27.27	32664788
XR10_S10_SME_L001_R1_001	94.93	50	74.33	76	18.18	26747359
XR10_S10_SME_L001_R2_001	93.06	50	74.37	76	27.27	26747359
XR11_S11_SME_L001_R1_001	94.78	50	74.98	76	27.27	25806582
XR11_S11_SME_L001_R2_001	92.65	49	75.01	76	27.27	25806582
XR12_S12_SME_L001_R1_001	95.05	50	75.07	76	27.27	27772434
XR12_S12_SME_L001_R2_001	92.87	49	75.10	76	27.27	27772434
XR1_S4_SME_L001_R1_001	94.00	50	75.31	76	27.27	25630219
XR1_S4_SME_L001_R2_001	91.91	49	75.33	76	27.27	25630219
XR2_S5_SME_L001_R1_001	93.58	50	74.76	76	27.27	28578032
XR2_S5_SME_L001_R2_001	91.43	49	74.79	76	27.27	28578032
XR3_S6_SME_L001_R1_001	94.01	51	74.39	76	27.27	21309949
XR3_S6_SME_L001_R2_001	92.04	51	74.41	76	27.27	21309949
XR4_S7_SME_L001_R1_001	94.52	50	74.24	76	18.18	30661793
XR4_S7_SME_L001_R2_001	92.37	49	74.28	76	27.27	30661793
XR5_S8_SME_L001_R1_001	94.71	50	74.42	76	18.18	29073022
XR5_S8_SME_L001_R2_001	92.70	50	74.45	76	27.27	29073022
XR6_S9_SME_L001_R1_001	94.64	50	75.08	76	18.18	23272093
XR6_S9_SME_L001_R2_001	92.72	49	75.11	76	27.27	23272093

Table S2: Differential expressed genes including the downregulated genes.						
**Time**	**Gene ID**	**logFC**	**logCPM**	**F**	**PValue**	**FDR**
24 hours	twi_ss.24166.1|g.31414	5.92	9.99	937.07	1.56E-11	4.74E-07
24 hours	twi_ss.3712.1|g.6096	7.57	10.33	611.10	1.40E-10	1.45E-06
24 hours	twi_ss.11705.1|g.15516	-3.97	8.15	608.58	1.43E-10	1.45E-06
24 hours	twi_ss.14278.1|g.18615	7.83	9.20	439.70	7.52E-10	4.74E-06
24 hours	twi_ss.20282.1|g.26105	3.27	7.34	436.65	7.79E-10	4.74E-06
24 hours	twi_ss.12823b.1|g.16930	-8.53	4.65	292.21	5.96E-09	2.59E-05
24 hours	twi_ss.4874.1|g.7384	-2.89	7.60	300.18	5.20E-09	2.59E-05
24 hours	twi_ss.11802b.2|g.15695	-2.00	7.33	271.18	8.67E-09	3.30E-05
24 hours	twi_ss.1219.1|g.2104	-2.73	8.02	261.53	1.04E-08	3.52E-05
24 hours	twi_ss.13838.1|g.18145	2.97	9.41	237.14	1.70E-08	5.17E-05
24 hours	twi_ss.1765.1|g.3114	2.97	9.24	217.95	2.59E-08	7.17E-05
24 hours	twi_ss.19960a.4|g.25706	7.12	4.70	199.83	3.99E-08	8.67E-05
24 hours	twi_ss.800.2|g.1407	-2.17	6.66	200.25	3.95E-08	8.67E-05
24 hours	twi_ss.26949.1|g.35778	-3.05	6.83	202.05	3.78E-08	8.67E-05
24 hours	twi_ss.9907.1|g.13240	3.59	7.26	193.07	4.73E-08	9.60E-05
24 hours	twi_ss.2969.1|g.5263	-2.39	8.81	187.14	5.52E-08	0.0001
24 hours	twi_ss.19960a.2|g.25708	-6.98	4.09	175.32	7.62E-08	0.0001
24 hours	twi_ss.21316b.3|g.27576	2.97	7.41	176.04	7.47E-08	0.0001
24 hours	twi_ss.4034.1|g.6451	-3.10	6.78	177.79	7.11E-08	0.0001
24 hours	twi_ss.24106.1|g.31280	5.16	6.28	180.59	6.59E-08	0.0001
24 hours	twi_ss.22567.1|g.29150	-2.90	6.27	171.02	8.61E-08	0.0001
24 hours	twi_ss.1226.1|g.2106	-2.56	7.40	168.37	9.31E-08	0.0001
24 hours	twi_ss.20735.1|g.26813	6.20	9.95	152.44	1.52E-07	0.0002
24 hours	twi_ss.2869.2|g.5156	3.57	7.03	144.87	1.94E-07	0.0002
24 hours	twi_ss.3533.1|g.5908	6.70	4.04	132.87	2.96E-07	0.0003
24 hours	twi_ss.3533.2|g.5904	6.70	4.04	132.87	2.96E-07	0.0003
24 hours	twi_ss.3533.3|g.5912	6.70	4.04	132.87	2.96E-07	0.0003
24 hours	twi_ss.3533.4|g.5906	6.70	4.04	132.87	2.96E-07	0.0003
24 hours	twi_ss.25105.9|g.32925	-7.25	3.41	135.10	2.73E-07	0.0003
24 hours	twi_ss.25751.1|g.33988	-1.68	7.05	138.17	2.45E-07	0.0003

24 hours	twi_ss.26746.1|g.35393	1.77	10.41	132.69	2.98E-07	0.0003
24 hours	twi_ss.27063.1|g.35999	8.39	5.95	219.86	2.56E-07	0.0003
24 hours	twi_ss.1289.1|g.2303	-2.43	9.19	133.12	2.93E-07	0.0003
24 hours	twi_ss.13698.1|g.18022	1.66	7.15	131.27	3.14E-07	0.0003
24 hours	twi_ss.26358.1|g.34798	7.40	9.69	126.16	3.81E-07	0.0003
24 hours	twi_ss.115.1|g.206	-2.52	7.10	123.97	4.14E-07	0.0003
24 hours	twi_ss.25745.1|g.33928	2.69	6.35	123.72	4.19E-07	0.0003
24 hours	twi_ss.2869.1|g.5155	5.82	5.94	123.65	4.20E-07	0.0003
24 hours	twi_ss.24962.1|g.32827	2.56	5.87	122.09	4.46E-07	0.0003
24 hours	twi_ss.12661.1|g.16777	-4.30	5.53	121.13	4.63E-07	0.0004
24 hours	twi_ss.503.1|g.907	-3.05	5.54	117.95	5.27E-07	0.0004
24 hours	twi_ss.6452.1|g.9489	3.55	9.20	116.81	5.52E-07	0.0004
24 hours	twi_ss.26516.1|g.35125	2.08	6.15	115.91	5.73E-07	0.0004
24 hours	twi_ss.18299.1|g.23330	-2.38	7.94	112.94	6.49E-07	0.0004
24 hours	twi_ss.26140b.3|g.34607	-1.91	8.94	112.13	6.72E-07	0.0004
24 hours	twi_ss.26950.1|g.35730	-2.82	5.71	113.58	6.32E-07	0.0004
24 hours	twi_ss.19635.1|g.25202	1.20	8.15	112.57	6.59E-07	0.0004
24 hours	twi_ss.2304.1|g.4056	-2.01	7.64	113.82	6.25E-07	0.0004
24 hours	twi_ss.20795.1|g.26925	7.54	10.91	111.33	6.95E-07	0.0004
24 hours	twi_ss.24136.1|g.31452	3.11	6.50	109.92	7.39E-07	0.0004
24 hours	twi_ss.15090.1|g.19568	-3.62	6.16	109.70	7.46E-07	0.0004
24 hours	twi_ss.28414.1|g.38016	3.37	6.71	106.25	8.69E-07	0.0005
24 hours	twi_ss.17348.1|g.22143	-1.22	8.00	102.49	1.03E-06	0.0006
24 hours	twi_ss.1972.1|g.3478	1.79	10.23	102.78	1.02E-06	0.0006
24 hours	twi_ss.1968.1|g.3462	1.79	11.14	102.47	1.03E-06	0.0006
24 hours	twi_ss.28038b.1|g.37328	1.44	8.70	101.62	1.08E-06	0.0006
24 hours	twi_ss.21950.1|g.28445	1.32	7.32	100.76	1.12E-06	0.0006
24 hours	twi_ss.8351.1|g.11463	1.69	6.89	99.82	1.17E-06	0.0006
24 hours	twi_ss.21564.1|g.27969	2.20	6.71	99.88	1.17E-06	0.0006
24 hours	twi_ss.22947.1|g.29606	-3.79	6.59	99.23	1.20E-06	0.0006
24 hours	twi_ss.17134.1|g.21914	-3.48	5.21	98.21	1.26E-06	0.0006
24 hours	twi_ss.4989.1|g.7605	2.21	6.67	97.50	1.31E-06	0.0006
24 hours	twi_ss.5201.1|g.7826	-1.79	8.11	97.29	1.32E-06	0.0006

24 hours	twi_ss.21114b.1|g.27305	-2.45	6.39	95.97	1.41E-06	0.0007
24 hours	twi_ss.25566.1|g.33668	-4.58	4.98	95.66	1.43E-06	0.0007
24 hours	twi_ss.9342.1|g.12619	1.88	6.79	94.40	1.52E-06	0.0007
24 hours	twi_ss.20001.1|g.25769	-4.45	6.01	93.16	1.62E-06	0.0007
24 hours	twi_ss.3352.1|g.5755	1.90	6.76	92.49	1.68E-06	0.0008
24 hours	twi_ss.4598.1|g.7155	-3.76	5.64	90.91	1.82E-06	0.0008
24 hours	twi_ss.30297.1|g.40939	-3.18	5.44	90.23	1.89E-06	0.0008
24 hours	twi_ss.3513.1|g.5884	-1.53	7.03	89.80	1.93E-06	0.0008
24 hours	twi_ss.22376.1|g.28895	2.29	7.47	89.89	1.92E-06	0.0008
24 hours	twi_ss.21105.1|g.27279	6.26	9.10	89.37	1.97E-06	0.0008
24 hours	twi_ss.16520.1|g.21215	-1.47	6.89	88.91	2.02E-06	0.0008
24 hours	twi_ss.1994.1|g.3586	3.03	5.57	86.89	2.25E-06	0.0009
24 hours	twi_ss.30108.1|g.40611	2.14	9.08	87.16	2.22E-06	0.0009
24 hours	twi_ss.6750.1|g.9832	2.52	8.24	86.75	2.27E-06	0.0009
24 hours	twi_ss.27194.1|g.36139	1.95	6.60	87.37	2.20E-06	0.0009
24 hours	twi_ss.28048.1|g.37371	4.00	7.95	86.15	2.34E-06	0.0009
24 hours	twi_ss.5288.1|g.7947	-1.79	6.75	84.82	2.52E-06	0.0010
24 hours	twi_ss.19751.1|g.25411	0.86	7.66	84.50	2.57E-06	0.0010
24 hours	twi_ss.32005b.4|g.43639	-2.99	6.26	84.25	2.60E-06	0.0010
24 hours	twi_ss.16983.1|g.21687	-2.23	6.15	83.76	2.67E-06	0.0010
24 hours	twi_ss.11375.1|g.14976	2.57	7.06	82.95	2.80E-06	0.0010
24 hours	twi_ss.13566.1|g.17913	3.90	8.08	82.89	2.81E-06	0.0010
24 hours	twi_ss.8361b.2|g.11480	2.05	7.66	83.22	2.76E-06	0.0010
24 hours	twi_ss.11135.1|g.14791	1.65	7.50	82.49	2.87E-06	0.0010
24 hours	twi_ss.28991.1|g.38906	-7.19	3.31	80.73	3.18E-06	0.0011
24 hours	twi_ss.28008.1|g.37358	1.30	7.20	80.39	3.24E-06	0.0011
24 hours	twi_ss.17312.1|g.22090	-2.67	8.49	80.09	3.30E-06	0.0011
24 hours	twi_ss.6597.1|g.9622	-1.22	9.42	79.82	3.35E-06	0.0011
24 hours	twi_ss.22743.11|g.29088	-3.08	5.56	79.39	3.43E-06	0.0011
24 hours	twi_ss.22642.1|g.29229	8.77	8.38	78.43	3.63E-06	0.0012
24 hours	twi_ss.20264c.9|g.26053	-2.45	6.16	78.39	3.64E-06	0.0012
24 hours	twi_ss.556.2|g.1009	-3.51	6.22	77.61	3.81E-06	0.0012
24 hours	twi_ss.5387.1|g.8143	-1.45	7.38	77.31	3.88E-06	0.0012

24 hours	twi_ss.2013.1|g.3579	-1.55	6.48	77.12	3.93E-06	0.0012
24 hours	twi_ss.2611.1|g.4635	2.50	8.33	76.03	4.20E-06	0.0012
24 hours	twi_ss.21908.1|g.28418	2.75	5.05	76.14	4.17E-06	0.0012
24 hours	twi_ss.19570.5|g.25098	6.70	4.87	76.02	4.20E-06	0.0012
24 hours	twi_ss.459c.1|g.826	-1.36	8.81	76.23	4.14E-06	0.0012
24 hours	twi_ss.13145.1|g.17465	-2.38	7.70	75.92	4.22E-06	0.0012
24 hours	twi_ss.30071.1|g.40561	-2.32	9.07	76.21	4.15E-06	0.0012
24 hours	twi_ss.29749.1|g.40117	1.74	6.32	75.50	4.34E-06	0.0013
24 hours	twi_ss.10027.1|g.13321	0.95	8.54	75.51	4.33E-06	0.0013
24 hours	twi_ss.19926.1|g.25684	1.36	9.57	74.09	4.73E-06	0.0014
24 hours	twi_ss.15653.1|g.20230	-2.08	7.67	73.38	4.94E-06	0.0014
24 hours	twi_ss.27219.1|g.36158	2.03	10.24	73.34	4.96E-06	0.0014
24 hours	twi_ss.19469.1|g.24973	-1.84	7.59	72.81	5.12E-06	0.0014
24 hours	twi_ss.6830.1|g.9893	1.95	6.09	72.63	5.18E-06	0.0014
24 hours	twi_ss.25773.1|g.33565	1.44	9.26	72.28	5.30E-06	0.0015
24 hours	twi_ss.15820.1|g.20389	-1.22	8.51	72.07	5.37E-06	0.0015
24 hours	twi_ss.30155.1|g.40723	-1.04	7.96	71.98	5.40E-06	0.0015
24 hours	twi_ss.7586.1|g.10632	1.28	8.25	71.68	5.51E-06	0.0015
24 hours	twi_ss.20935.1|g.27048	-4.72	5.89	71.44	5.59E-06	0.0015
24 hours	twi_ss.29730.1|g.40162	-2.55	6.29	71.35	5.62E-06	0.0015
24 hours	twi_ss.2376.1|g.4266	-2.45	6.29	71.05	5.73E-06	0.0015
24 hours	twi_ss.25621.4|g.33682	-1.62	6.33	70.55	5.92E-06	0.0015
24 hours	twi_ss.28506.2|g.38174	-2.47	6.76	70.67	5.88E-06	0.0015
24 hours	twi_ss.9920.1|g.13258	-6.08	2.70	70.14	6.08E-06	0.0015
24 hours	twi_ss.22907.1|g.29571	-1.89	8.99	70.22	6.05E-06	0.0015
24 hours	twi_ss.23722.1|g.30715	2.08	7.62	69.80	6.22E-06	0.0016
24 hours	twi_ss.25333.1|g.33360	1.20	8.35	68.81	6.64E-06	0.0016
24 hours	twi_ss.21437.1|g.27736	1.38	8.44	68.64	6.72E-06	0.0016
24 hours	twi_ss.7998.1|g.11044	-3.07	7.51	68.45	6.80E-06	0.0017
24 hours	twi_ss.2053.1|g.3716	-3.46	4.96	68.32	6.86E-06	0.0017
24 hours	twi_ss.26471.1|g.35054	1.35	7.31	67.80	7.10E-06	0.0017
24 hours	twi_ss.30689.2|g.41599	-2.32	7.89	67.50	7.25E-06	0.0017
24 hours	twi_ss.5561.1|g.8433	-2.47	7.51	67.19	7.40E-06	0.0017

24 hours	twi_ss.800.1|g.1410	-1.81	7.58	66.52	7.75E-06	0.0018
24 hours	twi_ss.28211.1|g.37661	2.14	5.47	66.34	7.84E-06	0.0018
24 hours	twi_ss.14780.1|g.19240	2.28	7.81	66.27	7.89E-06	0.0018
24 hours	twi_ss.9041.1|g.12212	1.35	8.47	66.14	7.95E-06	0.0018
24 hours	twi_ss.3793.1|g.6196	-3.02	4.89	66.00	8.03E-06	0.0018
24 hours	twi_ss.4661.2|g.7185	-1.57	6.38	65.61	8.25E-06	0.0019
24 hours	twi_ss.31777.1|g.43288	4.55	6.49	65.33	8.41E-06	0.0019
24 hours	twi_ss.18906.1|g.24170	0.83	7.78	64.34	9.02E-06	0.0020
24 hours	twi_ss.27006a.1|g.35879	1.52	6.73	64.40	8.98E-06	0.0020
24 hours	twi_ss.20178.1|g.25926	-1.41	8.30	64.16	9.13E-06	0.0020
24 hours	twi_ss.22212.1|g.28787	6.93	5.09	64.00	9.24E-06	0.0020
24 hours	twi_ss.11541a.1|g.15331	1.09	6.61	63.92	9.29E-06	0.0020
24 hours	twi_ss.31732.1|g.43215	-2.24	6.50	63.01	9.91E-06	0.0021
24 hours	twi_ss.19895.1|g.25639	1.38	8.07	63.27	9.73E-06	0.0021
24 hours	twi_ss.18952b.3|g.24232	-2.25	5.38	63.20	9.78E-06	0.0021
24 hours	twi_ss.17802.1|g.22700	6.28	10.31	62.94	9.96E-06	0.0021
24 hours	twi_ss.5306.1|g.7938	1.13	7.21	62.92	9.98E-06	0.0021
24 hours	twi_ss.21030.1|g.27189	-2.27	5.89	62.78	1.01E-05	0.0021
24 hours	twi_ss.8767.1|g.11983	-4.10	5.66	62.08	1.06E-05	0.0022
24 hours	twi_ss.16815.1|g.21469	-1.78	5.95	62.18	1.05E-05	0.0022
24 hours	twi_ss.26446.1|g.35014	1.35	9.82	62.05	1.06E-05	0.0022
24 hours	twi_ss.6950.1|g.10039	1.69	5.85	61.76	1.08E-05	0.0022
24 hours	twi_ss.13604.2|g.17956	1.35	8.04	61.76	1.08E-05	0.0022
24 hours	twi_ss.25742.1|g.33929	2.57	4.74	61.41	1.11E-05	0.0022
24 hours	twi_ss.30096b.2|g.40711	1.91	9.17	61.39	1.12E-05	0.0022
24 hours	twi_ss.2982.1|g.5306	-1.22	9.62	61.13	1.14E-05	0.0022
24 hours	twi_ss.1397.1|g.2511	-4.04	5.86	60.97	1.15E-05	0.0022
24 hours	twi_ss.21438.1|g.27729	1.65	6.44	60.60	1.18E-05	0.0023
24 hours	twi_ss.28158a.3|g.37550	-5.85	2.83	60.21	1.22E-05	0.0023
24 hours	twi_ss.25299b.2|g.33311	2.28	9.98	60.02	1.23E-05	0.0024
24 hours	twi_ss.26139.1|g.34560	-2.03	7.07	59.82	1.25E-05	0.0024
24 hours	twi_ss.10495.1|g.14129	1.57	9.39	59.81	1.25E-05	0.0024
24 hours	twi_ss.14313a.1|g.18652	-1.49	7.05	59.45	1.29E-05	0.0024

24 hours	twi_ss.30481.2|g.41260	2.55	6.73	59.36	1.30E-05	0.0024
24 hours	twi_ss.7980.1|g.11036	-3.04	5.39	58.94	1.34E-05	0.0025
24 hours	twi_ss.20755a.2|g.26865	-1.00	8.81	58.96	1.34E-05	0.0025
24 hours	twi_ss.17501.1|g.22280	1.01	9.69	58.89	1.34E-05	0.0025
24 hours	twi_ss.8770.1|g.11923	-2.98	7.09	58.67	1.37E-05	0.0025
24 hours	twi_ss.3795.1|g.6223	-4.02	3.80	58.29	1.41E-05	0.0025
24 hours	twi_ss.10334.1|g.13899	-2.37	6.98	58.29	1.41E-05	0.0025
24 hours	twi_ss.6554.1|g.9582	-1.60	6.74	58.14	1.42E-05	0.0025
24 hours	twi_ss.23382.1|g.30079	-1.25	9.28	58.14	1.42E-05	0.0025
24 hours	twi_ss.5279.3|g.7978	-6.94	3.31	79.34	1.46E-05	0.0026
24 hours	twi_ss.20152.1|g.25917	-1.29	9.26	57.63	1.48E-05	0.0026
24 hours	twi_ss.7769.1|g.10845	-2.14	6.58	57.68	1.48E-05	0.0026
24 hours	twi_ss.21105.2|g.27285	6.00	7.96	57.29	1.52E-05	0.0026
24 hours	twi_ss.13405.1|g.17801	-3.05	8.97	57.35	1.51E-05	0.0026
24 hours	twi_ss.30543.1|g.41378	-3.21	4.85	57.46	1.50E-05	0.0026
24 hours	twi_ss.11664.1|g.15469	2.54	6.76	57.36	1.51E-05	0.0026
24 hours	twi_ss.5329.1|g.7937	1.71	7.79	57.14	1.54E-05	0.0026
24 hours	twi_ss.20175.1|g.25982	-1.44	6.26	56.40	1.63E-05	0.0027
24 hours	twi_ss.5643.1|g.8453	2.18	10.41	56.23	1.65E-05	0.0027
24 hours	twi_ss.12272.1|g.16279	2.91	5.31	56.27	1.65E-05	0.0027
24 hours	twi_ss.28014.2|g.37321	-6.00	2.61	56.27	1.65E-05	0.0027
24 hours	twi_ss.4684.1|g.7239	-1.71	5.82	55.61	1.74E-05	0.0029
24 hours	twi_ss.25312.3|g.33264	4.87	7.88	55.65	1.73E-05	0.0029
24 hours	twi_ss.817.1|g.1411	-1.85	7.86	55.56	1.74E-05	0.0029
24 hours	twi_ss.12047.1|g.15946	1.65	6.60	55.36	1.77E-05	0.0029
24 hours	twi_ss.20483.1|g.26437	1.41	7.17	55.31	1.78E-05	0.0029
24 hours	twi_ss.21194.1|g.27415	-2.00	6.45	55.21	1.79E-05	0.0029
24 hours	twi_ss.16652.1|g.21306	-1.52	6.63	55.15	1.80E-05	0.0029
24 hours	twi_ss.1036.1|g.1871	4.35	6.08	55.07	1.81E-05	0.0029
24 hours	twi_ss.6204.1|g.9043	1.83	8.39	55.00	1.82E-05	0.0029
24 hours	twi_ss.30032.1|g.40525	-2.07	7.40	54.31	1.93E-05	0.0030
24 hours	twi_ss.26323.1|g.34866	2.02	6.74	54.15	1.95E-05	0.0030
24 hours	twi_ss.22038.1|g.28557	1.32	7.05	54.18	1.95E-05	0.0030

24 hours	twi_ss.23060.1|g.29693	2.77	6.41	54.04	1.97E-05	0.0031
24 hours	twi_ss.24308.1|g.31752	-1.05	7.39	53.76	2.02E-05	0.0031
24 hours	twi_ss.26361a.2|g.34929	-1.97	6.90	53.57	2.05E-05	0.0031
24 hours	twi_ss.23264.1|g.29908	-1.39	6.66	53.61	2.04E-05	0.0031
24 hours	twi_ss.27827b.1|g.37022	-2.59	6.12	53.28	2.10E-05	0.0032
24 hours	twi_ss.22668.4|g.29253	-6.15	3.14	53.03	2.14E-05	0.0032
24 hours	twi_ss.1469.1|g.2686	-1.83	4.99	52.87	2.17E-05	0.0033
24 hours	twi_ss.4815a.1|g.7394	0.99	7.58	52.83	2.18E-05	0.0033
24 hours	twi_ss.15378.1|g.19918	-1.39	6.14	52.39	2.26E-05	0.0034
24 hours	twi_ss.5069.1|g.7717	-2.31	7.16	52.30	2.28E-05	0.0034
24 hours	twi_ss.26491.1|g.35096	-7.53	4.41	70.38	2.30E-05	0.0034
24 hours	twi_ss.3533.5|g.5910	-2.22	5.35	51.98	2.34E-05	0.0034
24 hours	twi_ss.10752.1|g.14417	3.16	6.96	51.62	2.41E-05	0.0035
24 hours	twi_ss.21105.3|g.27275	5.44	6.10	51.50	2.44E-05	0.0035
24 hours	twi_ss.17219a.12|g.21775	0.74	9.25	51.53	2.43E-05	0.0035
24 hours	twi_ss.17195.1|g.21952	-3.62	5.10	51.42	2.45E-05	0.0035
24 hours	twi_ss.29136.1|g.39098	-2.44	5.13	50.99	2.55E-05	0.0036
24 hours	twi_ss.27737.2|g.36921	-1.24	7.98	50.98	2.55E-05	0.0036
24 hours	twi_ss.11279.1|g.14907	-3.65	4.16	50.88	2.57E-05	0.0036
24 hours	twi_ss.22064.1|g.28563	-3.15	5.07	50.85	2.58E-05	0.0036
24 hours	twi_ss.4103.1|g.6462	2.36	9.01	50.60	2.63E-05	0.0037
24 hours	twi_ss.26708.1|g.35428	-3.33	6.20	50.62	2.63E-05	0.0037
24 hours	twi_ss.3197.4|g.5619	-3.23	6.42	50.67	2.62E-05	0.0037
24 hours	twi_ss.28280.1|g.37812	1.06	8.58	50.45	2.67E-05	0.0037
24 hours	twi_ss.29381.1|g.39493	0.89	6.98	50.50	2.66E-05	0.0037
24 hours	twi_ss.24491.1|g.32093	-2.00	6.00	50.24	2.72E-05	0.0037
24 hours	twi_ss.30027b.2|g.40527	2.79	8.55	49.83	2.82E-05	0.0039
24 hours	twi_ss.6495.1|g.9528	-1.67	6.55	49.61	2.87E-05	0.0039
24 hours	twi_ss.24304.1|g.31690	1.15	9.51	49.59	2.88E-05	0.0039
24 hours	twi_ss.21448.1|g.27756	2.37	5.98	49.55	2.89E-05	0.0039
24 hours	twi_ss.4929.1|g.7529	1.58	6.55	49.34	2.94E-05	0.0040
24 hours	twi_ss.1420.1|g.2668	-2.76	5.37	49.19	2.98E-05	0.0040
24 hours	twi_ss.28854.1|g.38686	-1.77	7.67	49.17	2.99E-05	0.0040

24 hours	twi_ss.14756.4|g.19195	3.69	4.93	48.93	3.05E-05	0.0041
24 hours	twi_ss.9089.1|g.12267	-3.43	7.43	48.50	3.17E-05	0.0042
24 hours	twi_ss.4977.9|g.7534	8.79	6.96	48.56	3.15E-05	0.0042
24 hours	twi_ss.5298a.2|g.8044	1.27	6.43	48.47	3.18E-05	0.0042
24 hours	twi_ss.19329a.2|g.24785	1.51	8.63	48.45	3.18E-05	0.0042
24 hours	twi_ss.30546.1|g.41379	4.36	7.52	48.37	3.21E-05	0.0042
24 hours	twi_ss.1474a.1|g.2550	1.17	5.86	47.85	3.36E-05	0.0044
24 hours	twi_ss.8099.3|g.11155	-1.39	6.38	47.72	3.40E-05	0.0044
24 hours	twi_ss.13436.1|g.17773	-2.14	7.23	47.67	3.42E-05	0.0044
24 hours	twi_ss.24967.1|g.32796	5.39	7.66	47.52	3.46E-05	0.0044
24 hours	twi_ss.12458.1|g.16622	9.30	5.22	47.28	3.54E-05	0.0045
24 hours	twi_ss.21339.5|g.27594	-4.80	2.19	46.97	3.64E-05	0.0046
24 hours	twi_ss.1766.1|g.3105	2.24	6.37	46.88	3.67E-05	0.0046
24 hours	twi_ss.14354.1|g.18661	2.54	8.80	46.92	3.66E-05	0.0046
24 hours	twi_ss.21485.1|g.27864	0.98	9.85	47.00	3.63E-05	0.0046
24 hours	twi_ss.207.1|g.332	-1.76	8.02	47.01	3.63E-05	0.0046
24 hours	twi_ss.24299b.2|g.31693	1.01	6.91	46.84	3.69E-05	0.0046
24 hours	twi_ss.27582c.1|g.36775	1.43	7.64	46.85	3.68E-05	0.0046
24 hours	twi_ss.28706.1|g.38429	1.68	6.38	46.78	3.71E-05	0.0046
24 hours	twi_ss.26359.1|g.34933	4.02	3.66	46.63	3.76E-05	0.0046
24 hours	twi_ss.29444.1|g.39654	-0.76	7.31	46.65	3.75E-05	0.0046
24 hours	twi_ss.31219.1|g.42419	2.00	6.87	46.60	3.77E-05	0.0046
24 hours	twi_ss.4874.2|g.7378	-0.84	8.80	46.61	3.77E-05	0.0046
24 hours	twi_ss.8490.1|g.11663	-1.88	5.06	46.52	3.80E-05	0.0046
24 hours	twi_ss.23845.1|g.30845	-5.30	7.37	46.34	3.86E-05	0.0046
24 hours	twi_ss.21231.1|g.27456	1.19	6.70	46.14	3.93E-05	0.0047
24 hours	twi_ss.10044.1|g.13359	-1.97	7.06	45.85	4.04E-05	0.0048
24 hours	twi_ss.31247.1|g.42483	8.30	6.49	45.84	4.05E-05	0.0048
24 hours	twi_ss.23785b.1|g.30813	1.66	7.64	45.77	4.07E-05	0.0048
24 hours	twi_ss.25125.1|g.32989	1.40	7.64	45.66	4.12E-05	0.0049
24 hours	twi_ss.29777.1|g.40102	-4.79	2.32	45.56	4.15E-05	0.0049
24 hours	twi_ss.17899.1|g.22790	3.45	5.10	45.58	4.15E-05	0.0049
24 hours	twi_ss.24847.1|g.32681	1.41	5.83	45.48	4.19E-05	0.0049

24 hours	twi_ss.22497.1|g.29072	1.38	9.61	45.27	4.27E-05	0.0050
24 hours	twi_ss.3881.1|g.6304	1.60	5.65	45.20	4.30E-05	0.0050
24 hours	twi_ss.27594.1|g.36809	-2.75	4.41	45.12	4.34E-05	0.0050
24 hours	twi_ss.8953.1|g.12138	-2.28	5.87	44.75	4.49E-05	0.0051
24 hours	twi_ss.29224.1|g.39184	-1.82	6.29	44.74	4.50E-05	0.0051
24 hours	twi_ss.14946a.1|g.19352	1.12	6.81	44.79	4.47E-05	0.0051
24 hours	twi_ss.30724.1|g.41642	2.55	7.69	44.83	4.46E-05	0.0051
24 hours	twi_ss.13863.1|g.18169	1.18	8.89	44.70	4.51E-05	0.0051
24 hours	twi_ss.27617.1|g.36652	1.67	6.02	44.62	4.55E-05	0.0051
24 hours	twi_ss.9015.1|g.12194	0.90	8.42	44.60	4.55E-05	0.0051
24 hours	twi_ss.17531b.1|g.22398	2.27	7.62	44.50	4.60E-05	0.0051
24 hours	twi_ss.2758.1|g.5058	1.29	6.78	44.48	4.61E-05	0.0051
24 hours	twi_ss.574.2|g.1054	2.33	7.11	44.37	4.66E-05	0.0052
24 hours	twi_ss.6415.1|g.9461	-1.51	8.11	44.30	4.69E-05	0.0052
24 hours	twi_ss.21215.1|g.27452	1.77	6.07	44.25	4.71E-05	0.0052
24 hours	twi_ss.13778.1|g.18098	-2.82	7.32	44.28	4.70E-05	0.0052
24 hours	twi_ss.14178.1|g.18455	-1.94	8.45	44.20	4.74E-05	0.0052
24 hours	twi_ss.25621.2|g.33688	7.11	3.74	44.03	4.81E-05	0.0053
24 hours	twi_ss.30160.1|g.40720	1.05	7.65	43.98	4.83E-05	0.0053
24 hours	twi_ss.2981.1|g.5305	-2.89	5.12	43.86	4.89E-05	0.0053
24 hours	twi_ss.15060.1|g.19559	1.19	6.84	43.89	4.88E-05	0.0053
24 hours	twi_ss.29296.1|g.39294	1.10	9.97	43.90	4.87E-05	0.0053
24 hours	twi_ss.15686.1|g.20257	-2.20	4.88	43.79	4.93E-05	0.0053
24 hours	twi_ss.11312.1|g.14924	-1.64	7.86	43.70	4.97E-05	0.0053
24 hours	twi_ss.8755.1|g.11979	-2.04	5.95	43.55	5.05E-05	0.0054
24 hours	twi_ss.4710.1|g.7263	-2.03	5.16	43.54	5.05E-05	0.0054
24 hours	twi_ss.12027.1|g.15857	1.29	7.26	43.41	5.12E-05	0.0054
24 hours	twi_ss.20452.1|g.26367	2.02	9.11	43.15	5.25E-05	0.0055
24 hours	twi_ss.6603b.1|g.9643	2.99	8.46	43.17	5.24E-05	0.0055
24 hours	twi_ss.24052.1|g.31321	4.98	3.46	43.06	5.30E-05	0.0055
24 hours	twi_ss.24052.2|g.31320	4.98	3.46	43.06	5.30E-05	0.0055
24 hours	twi_ss.11378.3|g.14984	1.05	8.61	43.00	5.33E-05	0.0055
24 hours	twi_ss.23133.1|g.29738	1.73	7.15	42.86	5.40E-05	0.0056

24 hours	twi_ss.2422.1|g.4431	4.03	5.64	42.80	5.43E-05	0.0056
24 hours	twi_ss.2309.1|g.4083	-2.07	7.22	42.64	5.52E-05	0.0057
24 hours	twi_ss.24302.1|g.31671	1.06	10.46	42.56	5.56E-05	0.0057
24 hours	twi_ss.30134.5|g.40699	1.99	8.79	42.50	5.60E-05	0.0057
24 hours	twi_ss.647.1|g.1197	0.71	7.65	42.20	5.77E-05	0.0059
24 hours	twi_ss.20998.1|g.27114	-2.37	4.34	42.06	5.85E-05	0.0059
24 hours	twi_ss.235.6|g.443	1.84	6.70	41.86	5.97E-05	0.0060
24 hours	twi_ss.24909.1|g.32761	-2.01	6.32	41.89	5.95E-05	0.0060
24 hours	twi_ss.12588.1|g.16705	-2.64	4.28	41.89	5.95E-05	0.0060
24 hours	twi_ss.31915.5|g.43431	1.23	8.46	41.75	6.04E-05	0.0060
24 hours	twi_ss.22941.1|g.29608	-4.83	3.75	41.76	6.03E-05	0.0060
24 hours	twi_ss.24587a.1|g.32232	-3.73	4.61	41.66	6.09E-05	0.0061
24 hours	twi_ss.406.1|g.754	1.70	6.22	41.45	6.22E-05	0.0062
24 hours	twi_ss.12889.1|g.17077	-2.65	4.65	41.02	6.50E-05	0.0064
24 hours	twi_ss.25693a.1|g.33896	1.68	11.28	40.98	6.53E-05	0.0064
24 hours	twi_ss.10279.1|g.13918	5.96	3.45	52.99	6.66E-05	0.0065
24 hours	twi_ss.30017.1|g.40502	1.71	7.07	40.67	6.75E-05	0.0066
24 hours	twi_ss.10516.1|g.14150	4.92	5.54	40.59	6.80E-05	0.0066
24 hours	twi_ss.2232.1|g.3943	1.41	6.78	40.55	6.83E-05	0.0066
24 hours	twi_ss.22743.10|g.29084	-3.55	4.68	40.31	7.00E-05	0.0068
24 hours	twi_ss.4286.1|g.6756	-1.09	10.01	40.31	7.00E-05	0.0068
24 hours	twi_ss.26054.4|g.34465	-1.10	9.01	40.29	7.02E-05	0.0068
24 hours	twi_ss.24846a.1|g.32671	-1.80	6.44	40.17	7.11E-05	0.0068
24 hours	twi_ss.27397.1|g.36477	0.88	7.82	40.14	7.13E-05	0.0068
24 hours	twi_ss.22936.1|g.29601	-2.08	4.94	40.17	7.11E-05	0.0068
24 hours	twi_ss.3149.1|g.5588	1.20	7.11	40.19	7.09E-05	0.0068
24 hours	twi_ss.1218.1|g.2194	-3.51	4.38	40.10	7.16E-05	0.0068
24 hours	twi_ss.1035.1|g.1876	4.27	5.08	40.02	7.22E-05	0.0068
24 hours	twi_ss.29512.1|g.39780	2.25	8.31	39.93	7.29E-05	0.0069
24 hours	twi_ss.13619b.2|g.17975	0.98	6.81	39.88	7.33E-05	0.0069
24 hours	twi_ss.18336.1|g.23366	-2.37	6.50	39.84	7.36E-05	0.0069
24 hours	twi_ss.25969.1|g.34284	-1.96	5.36	39.66	7.50E-05	0.0070
24 hours	twi_ss.12025.1|g.15916	1.79	7.28	39.62	7.53E-05	0.0070

24 hours	twi_ss.14648.1|g.19094	-2.40	5.24	39.59	7.56E-05	0.0070
24 hours	twi_ss.12970.1|g.17215	-1.86	6.09	39.51	7.62E-05	0.0070
24 hours	twi_ss.3973.1|g.6373	3.28	4.08	39.50	7.63E-05	0.0070
24 hours	twi_ss.14090.1|g.18371	2.51	8.15	39.50	7.63E-05	0.0070
24 hours	twi_ss.31673.1|g.43122	1.61	8.01	39.44	7.68E-05	0.0070
24 hours	twi_ss.28364.1|g.37940	-1.68	5.82	39.46	7.66E-05	0.0070
24 hours	twi_ss.30031.1|g.40544	-1.93	5.60	39.39	7.72E-05	0.0070
24 hours	twi_ss.22201a.1|g.28770	-1.47	6.17	39.37	7.74E-05	0.0070
24 hours	twi_ss.24167.1|g.31522	1.54	7.58	39.26	7.82E-05	0.0071
24 hours	twi_ss.12144.1|g.16027	0.81	7.87	39.14	7.93E-05	0.0072
24 hours	twi_ss.19471.1|g.24990	-1.55	5.77	39.08	7.98E-05	0.0072
24 hours	twi_ss.7837.1|g.10927	-2.26	4.50	39.09	7.97E-05	0.0072
24 hours	twi_ss.22049a.2|g.28532	-1.19	8.29	38.81	8.22E-05	0.0074
24 hours	twi_ss.18869.1|g.24133	-1.55	6.98	38.61	8.39E-05	0.0075
24 hours	twi_ss.2375a.2|g.4144	-1.08	6.79	38.62	8.38E-05	0.0075
24 hours	twi_ss.11249.1|g.14908	1.73	4.36	38.55	8.45E-05	0.0075
24 hours	twi_ss.7206.1|g.10257	-1.79	6.36	38.47	8.53E-05	0.0075
24 hours	twi_ss.11446.1|g.15098	-2.52	4.91	38.29	8.70E-05	0.0077
24 hours	twi_ss.6365.1|g.9392	1.86	8.35	38.14	8.84E-05	0.0078
24 hours	twi_ss.571.1|g.1040	1.76	4.71	38.04	8.93E-05	0.0078
24 hours	twi_ss.13571.1|g.17935	-2.81	6.07	38.04	8.93E-05	0.0078
24 hours	twi_ss.26382.1|g.34889	-1.00	6.31	37.98	9.00E-05	0.0078
24 hours	twi_ss.32072.1|g.43738	-2.62	3.91	37.91	9.06E-05	0.0079
24 hours	twi_ss.29266.1|g.39292	-2.09	4.58	37.69	9.28E-05	0.0080
24 hours	twi_ss.6558.5|g.9595	-5.04	4.82	48.36	9.31E-05	0.0080
24 hours	twi_ss.28156a.2|g.37510	-4.80	9.33	37.64	9.34E-05	0.0080
24 hours	twi_ss.8303a.1|g.11337	0.78	11.05	37.62	9.35E-05	0.0080
24 hours	twi_ss.25693b.2|g.33886	1.58	9.40	37.52	9.47E-05	0.0081
24 hours	twi_ss.9822.1|g.13153	-2.74	4.90	37.39	9.60E-05	0.0082
24 hours	twi_ss.637.1|g.1141	1.10	7.56	37.34	9.65E-05	0.0082
24 hours	twi_ss.3009.2|g.5339	-4.57	5.25	37.27	9.73E-05	0.0083
24 hours	twi_ss.23460.1|g.30091	1.81	6.15	37.11	9.91E-05	0.0084
24 hours	twi_ss.28020.1|g.37348	1.75	5.36	37.06	9.96E-05	0.0084

24 hours	twi_ss.24735.5|g.32332	2.59	5.60	36.96	0.0001	0.0085
24 hours	twi_ss.21214.1|g.27436	-1.59	7.28	36.92	0.0001	0.0085
24 hours	twi_ss.31490.1|g.42882	-4.06	4.19	36.83	0.0001	0.0086
24 hours	twi_ss.30862.1|g.42102	-1.54	5.47	36.67	0.0001	0.0087
24 hours	twi_ss.8759.1|g.11942	5.57	4.86	36.63	0.0001	0.0087
24 hours	twi_ss.28214.1|g.37656	2.49	5.22	36.48	0.0001	0.0088
24 hours	twi_ss.6620.1|g.9636	1.25	9.55	36.38	0.0001	0.0089
24 hours	twi_ss.20681.1|g.26792	1.56	6.36	36.40	0.0001	0.0089
24 hours	twi_ss.23775.1|g.30662	0.84	7.75	36.40	0.0001	0.0089
24 hours	twi_ss.6679.1|g.9780	1.21	7.29	36.30	0.0001	0.0089
24 hours	twi_ss.5056.1|g.7705	-2.60	6.57	36.23	0.0001	0.0090
24 hours	twi_ss.20287b.1|g.26122	0.87	7.52	35.96	0.0001	0.0092
24 hours	twi_ss.21865.1|g.28349	-1.95	7.57	35.76	0.0001	0.0094
24 hours	twi_ss.24154.1|g.31475	1.07	6.63	35.76	0.0001	0.0094
24 hours	twi_ss.6075.1|g.8954	-2.79	6.85	35.72	0.0001	0.0094
24 hours	twi_ss.26932.1|g.35737	2.60	4.03	35.68	0.0001	0.0094
24 hours	twi_ss.24804.1|g.32562	0.79	7.86	35.65	0.0001	0.0094
24 hours	twi_ss.20501.1|g.26442	1.65	5.64	35.42	0.0001	0.0096
24 hours	twi_ss.19882.1|g.25643	1.60	6.84	35.46	0.0001	0.0096
24 hours	twi_ss.17326.7|g.22111	8.55	5.90	35.43	0.0001	0.0096
24 hours	twi_ss.2218.1|g.3944	-3.67	4.37	35.37	0.0001	0.0096
24 hours	twi_ss.6610.1|g.9638	2.83	5.00	35.35	0.0001	0.0096
24 hours	twi_ss.20264c.8|g.26050	7.15	4.91	44.93	0.0001	0.0097
24 hours	twi_ss.25320.1|g.33273	1.50	6.76	35.20	0.0001	0.0097
24 hours	twi_ss.5820.1|g.8724	-1.36	7.97	35.24	0.0001	0.0097
24 hours	twi_ss.30242b.2|g.40842	7.17	5.72	35.19	0.0001	0.0097
24 hours	twi_ss.30768.1|g.41766	-1.97	4.82	35.17	0.0001	0.0097
24 hours	twi_ss.23465.1|g.30092	-1.23	5.75	35.12	0.0001	0.0097
24 hours	twi_ss.24809.1|g.32606	-1.76	8.19	35.12	0.0001	0.0097
24 hours	twi_ss.2800a.2|g.5025	1.82	9.06	34.97	0.0001	0.0099
24 hours	twi_ss.12689.1|g.16748	-1.79	5.29	34.95	0.0001	0.0099
24 hours	twi_ss.13293.1|g.17661	-0.67	9.72	34.75	0.0001	0.0101
24 hours	twi_ss.2739b.1|g.4925	0.97	6.06	34.72	0.0001	0.0101

24 hours	twi_ss.20485.1|g.26435	1.53	7.15	34.68	0.0001	0.0101
24 hours	twi_ss.25119.3|g.32978	0.77	8.31	34.68	0.0001	0.0101
24 hours	twi_ss.4876.1|g.7475	2.48	5.29	34.62	0.0001	0.0101
24 hours	twi_ss.16184.1|g.20768	-2.02	5.70	43.79	0.0001	0.0102
24 hours	twi_ss.544.1|g.968	-1.93	5.47	34.55	0.0001	0.0102
24 hours	twi_ss.30540.1|g.41328	0.60	9.47	34.52	0.0001	0.0102
24 hours	twi_ss.27006a.3|g.35875	-4.40	3.86	34.49	0.0001	0.0102
24 hours	twi_ss.14752.1|g.19217	1.08	6.03	34.47	0.0001	0.0102
24 hours	twi_ss.26616.1|g.35296	-1.96	7.34	34.41	0.0001	0.0102
24 hours	twi_ss.1272.1|g.2274	1.34	7.59	34.31	0.0001	0.0103
24 hours	twi_ss.13330.1|g.17615	-2.01	5.33	34.28	0.0001	0.0103
24 hours	twi_ss.16421.1|g.21003	-2.97	5.22	34.14	0.0001	0.0105
24 hours	twi_ss.11442.1|g.15086	-1.44	7.83	34.12	0.0001	0.0105
24 hours	twi_ss.852.1|g.1428	-0.90	9.60	34.11	0.0001	0.0105
24 hours	twi_ss.5041.1|g.7696	-2.33	5.56	33.96	0.0001	0.0106
24 hours	twi_ss.23691.1|g.30491	0.99	11.80	33.97	0.0001	0.0106
24 hours	twi_ss.8401.1|g.11548	1.09	6.15	33.85	0.0001	0.0107
24 hours	twi_ss.1625.1|g.2826	1.01	7.16	33.86	0.0001	0.0107
24 hours	twi_ss.11367.1|g.14997	-1.31	7.72	33.51	0.0002	0.0111
24 hours	twi_ss.29979.1|g.40463	-2.37	4.62	33.50	0.0002	0.0111
24 hours	twi_ss.20279.1|g.26099	1.33	4.82	33.40	0.0002	0.0112
24 hours	twi_ss.31055a.1|g.42228	-2.71	5.90	33.28	0.0002	0.0114
24 hours	twi_ss.1823.1|g.3220	-2.90	3.69	33.26	0.0002	0.0114
24 hours	twi_ss.26855.1|g.35618	-3.36	4.45	33.22	0.0002	0.0114
24 hours	twi_ss.9756.1|g.13098	-2.47	4.88	33.06	0.0002	0.0116
24 hours	twi_ss.23575.1|g.30271	2.35	6.28	32.98	0.0002	0.0117
24 hours	twi_ss.27049.1|g.35935	-1.81	6.23	32.85	0.0002	0.0118
24 hours	twi_ss.19488.1|g.25029	-1.56	7.09	32.86	0.0002	0.0118
24 hours	twi_ss.27814.1|g.37026	-1.69	5.37	32.85	0.0002	0.0118
24 hours	twi_ss.2026.1|g.3585	0.66	7.72	32.74	0.0002	0.0119
24 hours	twi_ss.24903.1|g.32760	-2.80	4.48	32.57	0.0002	0.0121
24 hours	twi_ss.11691.1|g.15523	-1.37	5.69	32.54	0.0002	0.0122
24 hours	twi_ss.10317.1|g.13799	-3.10	4.50	32.29	0.0002	0.0124

24 hours	twi_ss.558.1|g.1024	2.11	4.96	32.31	0.0002	0.0124
24 hours	twi_ss.2178.9|g.3858	-1.64	5.69	32.24	0.0002	0.0124
24 hours	twi_ss.3498.1|g.5871	0.93	7.09	32.25	0.0002	0.0124
24 hours	twi_ss.1403.2|g.2525	8.60	4.86	32.25	0.0002	0.0124
24 hours	twi_ss.6316.1|g.9338	1.05	8.96	32.28	0.0002	0.0124
24 hours	twi_ss.14940.1|g.19451	-2.30	6.20	32.27	0.0002	0.0124
24 hours	twi_ss.18795.1|g.24044	-2.53	6.73	32.01	0.0002	0.0127
24 hours	twi_ss.6427.1|g.9474	-1.31	8.14	32.02	0.0002	0.0127
24 hours	twi_ss.14969.1|g.19471	1.12	7.00	32.03	0.0002	0.0127
24 hours	twi_ss.21114a.2|g.27303	-2.43	5.50	32.02	0.0002	0.0127
24 hours	twi_ss.20296.3|g.26134	1.99	6.26	31.81	0.0002	0.0130
24 hours	twi_ss.17309.1|g.22092	1.03	6.81	31.63	0.0002	0.0132
24 hours	twi_ss.21635b.1|g.28037	0.66	9.47	31.59	0.0002	0.0133
24 hours	twi_ss.26363.1|g.34804	0.74	6.52	31.43	0.0002	0.0135
24 hours	twi_ss.6359.1|g.9419	1.75	8.55	31.39	0.0002	0.0135
24 hours	twi_ss.30372.1|g.41048	-2.97	4.59	31.36	0.0002	0.0136
24 hours	twi_ss.5283.1|g.8000	-8.32	5.30	31.30	0.0002	0.0136
24 hours	twi_ss.25871.1|g.34134	0.67	7.99	31.26	0.0002	0.0136
24 hours	twi_ss.31260.1|g.42493	-1.70	5.11	31.27	0.0002	0.0136
24 hours	twi_ss.28259.1|g.37792	0.76	7.59	31.22	0.0002	0.0137
24 hours	twi_ss.5526b.1|g.8322	4.23	6.41	31.17	0.0002	0.0137
24 hours	twi_ss.8570.1|g.11715	-0.97	7.71	31.19	0.0002	0.0137
24 hours	twi_ss.5404.1|g.8149	-1.14	5.66	31.10	0.0002	0.0138
24 hours	twi_ss.22195.1|g.28756	-2.21	5.17	31.00	0.0002	0.0139
24 hours	twi_ss.23941.1|g.31111	-1.64	5.30	31.02	0.0002	0.0139
24 hours	twi_ss.14851.1|g.19306	2.49	4.66	31.00	0.0002	0.0139
24 hours	twi_ss.29246.1|g.39251	-1.56	6.12	31.02	0.0002	0.0139
24 hours	twi_ss.14250.1|g.18561	1.14	5.91	30.83	0.0002	0.0141
24 hours	twi_ss.19857.1|g.25621	1.50	7.39	30.77	0.0002	0.0142
24 hours	twi_ss.24834.1|g.32673	1.12	6.39	30.71	0.0002	0.0143
24 hours	twi_ss.19925.1|g.25689	1.49	5.31	30.64	0.0002	0.0144
24 hours	twi_ss.25703.4|g.33870	-3.41	4.92	30.60	0.0002	0.0144
24 hours	twi_ss.14580.1|g.19046	1.51	5.46	30.56	0.0002	0.0145

24 hours	twi_ss.7142.1|g.10189	0.64	7.28	30.48	0.0002	0.0145
24 hours	twi_ss.28912.1|g.38856	5.22	2.81	38.00	0.0002	0.0145
24 hours	twi_ss.28284.1|g.37584	5.07	6.04	30.49	0.0002	0.0145
24 hours	twi_ss.28506.3|g.38172	-1.38	6.07	30.45	0.0002	0.0145
24 hours	twi_ss.27170a.3|g.36112	-2.03	5.32	30.43	0.0002	0.0146
24 hours	twi_ss.29653.2|g.39980	-2.84	4.39	30.40	0.0002	0.0146
24 hours	twi_ss.23457.1|g.30113	0.90	6.65	30.31	0.0002	0.0147
24 hours	twi_ss.22572.1|g.29155	-3.59	5.01	30.31	0.0002	0.0147
24 hours	twi_ss.1778.2|g.3111	1.77	5.70	30.23	0.0002	0.0148
24 hours	twi_ss.29211.1|g.39148	2.40	7.87	30.11	0.0002	0.0150
24 hours	twi_ss.14231.1|g.18498	-1.78	5.07	30.08	0.0002	0.0150
24 hours	twi_ss.21299.1|g.27525	-2.73	3.92	30.08	0.0002	0.0150
24 hours	twi_ss.24532a.1|g.32203	-0.81	7.04	30.02	0.0002	0.0151
24 hours	twi_ss.27336a.3|g.36383	4.73	4.68	29.99	0.0002	0.0151
24 hours	twi_ss.12972.1|g.17144	2.23	4.99	29.93	0.0002	0.0152
24 hours	twi_ss.18235.1|g.23204	-2.56	6.46	29.86	0.0002	0.0153
24 hours	twi_ss.9257.1|g.12473	-1.92	5.70	29.78	0.0002	0.0154
24 hours	twi_ss.5155.1|g.7788	0.97	6.27	29.78	0.0002	0.0154
24 hours	twi_ss.26622.1|g.35284	1.11	7.49	29.77	0.0002	0.0154
24 hours	twi_ss.19774.1|g.25443	0.67	8.09	29.75	0.0002	0.0154
24 hours	twi_ss.16663.1|g.21329	-1.03	9.94	29.81	0.0002	0.0154
24 hours	twi_ss.30027b.1|g.40526	2.80	7.72	29.71	0.0002	0.0154
24 hours	twi_ss.26881.2|g.35642	-2.68	4.51	29.71	0.0002	0.0154
24 hours	twi_ss.14300.1|g.18619	-3.69	3.13	36.84	0.0002	0.0155
24 hours	twi_ss.3782.2|g.6191	0.80	7.62	29.63	0.0002	0.0155
24 hours	twi_ss.4437.1|g.6935	1.47	5.75	29.62	0.0002	0.0155
24 hours	twi_ss.110a.2|g.198	5.04	5.24	29.56	0.0002	0.0156
24 hours	twi_ss.28936.1|g.38782	-1.29	8.53	29.50	0.0003	0.0157
24 hours	twi_ss.29617.1|g.39880	2.76	9.59	29.42	0.0003	0.0158
24 hours	twi_ss.28225.3|g.37758	7.90	3.74	52.61	0.0003	0.0161
24 hours	twi_ss.21458.1|g.27724	2.11	5.53	29.27	0.0003	0.0161
24 hours	twi_ss.23705.1|g.30520	-1.39	6.01	29.25	0.0003	0.0161
24 hours	twi_ss.8151.1|g.11240	1.09	6.18	29.21	0.0003	0.0162

24 hours	twi_ss.15599.1|g.20166	0.74	7.05	29.12	0.0003	0.0162
24 hours	twi_ss.13003.1|g.17229	0.52	7.60	29.12	0.0003	0.0162
24 hours	twi_ss.20270.1|g.26073	1.96	4.86	29.15	0.0003	0.0162
24 hours	twi_ss.31896.1|g.43422	0.83	6.27	29.09	0.0003	0.0163
24 hours	twi_ss.20513.1|g.26432	-1.43	4.89	29.03	0.0003	0.0164
24 hours	twi_ss.10271a.1|g.13759	0.98	7.13	29.01	0.0003	0.0164
24 hours	twi_ss.8443.1|g.11629	-1.54	6.09	29.01	0.0003	0.0164
24 hours	twi_ss.8446.1|g.11632	-2.29	3.93	28.98	0.0003	0.0164
24 hours	twi_ss.21482.1|g.27787	2.44	4.96	28.97	0.0003	0.0164
24 hours	twi_ss.22111.1|g.28614	5.44	3.51	28.95	0.0003	0.0164
24 hours	twi_ss.7645.1|g.10747	-1.12	7.48	28.92	0.0003	0.0164
24 hours	twi_ss.12020.1|g.15854	1.03	6.75	28.86	0.0003	0.0165
24 hours	twi_ss.29485.1|g.39690	-2.31	4.49	28.86	0.0003	0.0165
24 hours	twi_ss.10703.10|g.14318	-1.42	5.29	28.87	0.0003	0.0165
24 hours	twi_ss.30512b.1|g.41315	-1.90	5.12	28.70	0.0003	0.0168
24 hours	twi_ss.8912.1|g.12098	-1.11	6.22	28.66	0.0003	0.0169
24 hours	twi_ss.19378.2|g.24898	4.46	2.72	28.62	0.0003	0.0169
24 hours	twi_ss.824.2|g.1435	-1.57	5.36	28.60	0.0003	0.0169
24 hours	twi_ss.18478.1|g.23540	-1.53	7.38	28.56	0.0003	0.0170
24 hours	twi_ss.30803a.1|g.41752	2.86	6.26	28.50	0.0003	0.0170
24 hours	twi_ss.7460.1|g.10475	-0.91	7.52	28.53	0.0003	0.0170
24 hours	twi_ss.14445.2|g.18827	-0.98	7.40	28.51	0.0003	0.0170
24 hours	twi_ss.19345.12|g.24807	-6.59	2.90	50.74	0.0003	0.0170
24 hours	twi_ss.6155.1|g.9054	-1.59	4.86	28.45	0.0003	0.0171
24 hours	twi_ss.15781.1|g.20345	2.08	5.60	28.43	0.0003	0.0171
24 hours	twi_ss.27241.3|g.36167	-1.51	5.66	28.40	0.0003	0.0171
24 hours	twi_ss.11609.1|g.15428	1.44	5.92	28.40	0.0003	0.0171
24 hours	twi_ss.16737.1|g.21429	1.23	7.44	28.37	0.0003	0.0171
24 hours	twi_ss.15332.1|g.19854	-0.65	9.12	28.36	0.0003	0.0171
24 hours	twi_ss.6264.1|g.9307	0.97	7.31	28.32	0.0003	0.0172
24 hours	twi_ss.28761.2|g.38490	-1.60	5.07	28.23	0.0003	0.0174
24 hours	twi_ss.9920.2|g.13257	5.02	2.76	28.18	0.0003	0.0175
24 hours	twi_ss.6907b.2|g.9981	-0.76	8.85	28.15	0.0003	0.0175

24 hours	twi_ss.31289.1|g.42555	-2.74	6.26	28.14	0.0003	0.0175
24 hours	twi_ss.30137.1|g.40684	-3.12	4.89	28.10	0.0003	0.0176
24 hours	twi_ss.23452.1|g.30105	-2.36	5.22	28.06	0.0003	0.0176
24 hours	twi_ss.24525.1|g.32198	-1.02	9.46	28.01	0.0003	0.0177
24 hours	twi_ss.24322.1|g.31835	1.16	4.93	28.00	0.0003	0.0177
24 hours	twi_ss.21657.1|g.28088	2.22	5.80	27.96	0.0003	0.0178
24 hours	twi_ss.19228.1|g.24602	-1.75	6.55	27.88	0.0003	0.0179
24 hours	twi_ss.6933.1|g.9993	1.22	5.35	27.86	0.0003	0.0179
24 hours	twi_ss.32100.1|g.43763	-2.97	4.96	27.80	0.0003	0.0181
24 hours	twi_ss.6252.3|g.9287	1.08	6.97	27.75	0.0003	0.0181
24 hours	twi_ss.26719.1|g.35434	-1.90	8.58	27.76	0.0003	0.0181
24 hours	twi_ss.670.2|g.1158	-1.00	6.07	27.55	0.0003	0.0186
24 hours	twi_ss.12376a.1|g.16510	-1.31	5.29	27.50	0.0003	0.0187
24 hours	twi_ss.22383.1|g.28898	-3.04	4.26	27.47	0.0003	0.0187
24 hours	twi_ss.31795.1|g.43313	-2.50	4.62	27.42	0.0003	0.0188
24 hours	twi_ss.8875.1|g.12104	-2.20	6.38	27.42	0.0003	0.0188
24 hours	twi_ss.10644.1|g.14288	1.59	5.72	27.44	0.0003	0.0188
24 hours	twi_ss.1006.1|g.1827	0.62	8.35	27.38	0.0003	0.0188
24 hours	twi_ss.25519.1|g.33528	1.53	4.96	27.35	0.0003	0.0189
24 hours	twi_ss.2174.1|g.3848	0.79	7.66	27.30	0.0003	0.0190
24 hours	twi_ss.6521.2|g.9553	1.05	9.92	27.29	0.0003	0.0190
24 hours	twi_ss.3491.1|g.5867	1.70	4.48	27.26	0.0003	0.0190
24 hours	twi_ss.3274.1|g.5683	1.01	6.69	27.13	0.0003	0.0193
24 hours	twi_ss.6152b.1|g.9127	0.59	8.40	27.13	0.0003	0.0193
24 hours	twi_ss.9652.1|g.12945	-4.44	4.17	27.09	0.0004	0.0194
24 hours	twi_ss.7080.1|g.10166	1.24	6.64	27.08	0.0004	0.0194
24 hours	twi_ss.978.1|g.1784	2.31	10.48	27.05	0.0004	0.0194
24 hours	twi_ss.5112.1|g.7738	2.65	5.43	27.02	0.0004	0.0195
24 hours	twi_ss.16288.1|g.20863	-4.75	4.77	33.13	0.0004	0.0195
24 hours	twi_ss.5622.1|g.8380	0.97	6.08	26.99	0.0004	0.0195
24 hours	twi_ss.6368.1|g.9413	0.55	7.59	26.92	0.0004	0.0196
24 hours	twi_ss.16705.3|g.21383	-6.57	2.97	26.92	0.0004	0.0196
24 hours	twi_ss.24823.1|g.32552	2.35	5.26	26.87	0.0004	0.0197

24 hours	twi_ss.22647.1|g.29224	3.96	4.20	26.80	0.0004	0.0199
24 hours	twi_ss.26983.1|g.35830	-1.19	6.04	26.75	0.0004	0.0200
24 hours	twi_ss.21105.6|g.27278	3.25	5.41	26.63	0.0004	0.0203
24 hours	twi_ss.7764.1|g.10838	-3.56	3.96	26.61	0.0004	0.0203
24 hours	twi_ss.3054.1|g.5433	-6.55	3.59	26.60	0.0004	0.0203
24 hours	twi_ss.12625.1|g.16729	0.58	9.41	26.62	0.0004	0.0203
24 hours	twi_ss.21076.1|g.27226	-0.88	6.23	26.55	0.0004	0.0203
24 hours	twi_ss.5526a.2|g.8328	3.58	4.67	26.53	0.0004	0.0203
24 hours	twi_ss.20143.1|g.25882	0.92	6.40	26.48	0.0004	0.0203
24 hours	twi_ss.20143.2|g.25887	0.92	6.40	26.48	0.0004	0.0203
24 hours	twi_ss.20143.3|g.25884	0.92	6.40	26.48	0.0004	0.0203
24 hours	twi_ss.20143.4|g.25883	0.92	6.40	26.48	0.0004	0.0203
24 hours	twi_ss.7454.1|g.10476	2.71	5.71	26.57	0.0004	0.0203
24 hours	twi_ss.30947a.1|g.41936	0.67	7.07	26.52	0.0004	0.0203
24 hours	twi_ss.12345.1|g.16456	0.97	5.87	26.49	0.0004	0.0203
24 hours	twi_ss.7115.1|g.10182	-2.49	5.46	26.46	0.0004	0.0203
24 hours	twi_ss.25622.1|g.33721	-2.98	4.95	26.45	0.0004	0.0203
24 hours	twi_ss.16171.1|g.20756	1.90	6.65	26.36	0.0004	0.0206
24 hours	twi_ss.2291.1|g.4028	-1.81	6.45	26.32	0.0004	0.0207
24 hours	twi_ss.24029.1|g.31245	-1.88	4.92	26.25	0.0004	0.0208
24 hours	twi_ss.19943.1|g.25743	0.80	6.83	26.16	0.0004	0.0211
24 hours	twi_ss.18098.1|g.23015	-2.14	4.89	26.10	0.0004	0.0211
24 hours	twi_ss.23703.1|g.30716	0.66	8.20	26.10	0.0004	0.0211
24 hours	twi_ss.14795.1|g.19270	-1.63	6.34	26.12	0.0004	0.0211
24 hours	twi_ss.31044.1|g.42224	-8.93	4.84	26.07	0.0004	0.0212
24 hours	twi_ss.11056b.2|g.14704	0.80	6.52	26.08	0.0004	0.0212
24 hours	twi_ss.6762.1|g.9844	-3.00	3.91	26.04	0.0004	0.0212
24 hours	twi_ss.27623.6|g.36625	1.08	8.21	26.01	0.0004	0.0213
24 hours	twi_ss.6314.1|g.9339	2.07	8.84	26.00	0.0004	0.0213
24 hours	twi_ss.25220.1|g.33111	-1.12	7.16	25.97	0.0004	0.0214
24 hours	twi_ss.5058.2|g.7681	3.62	6.26	25.93	0.0004	0.0214
24 hours	twi_ss.10702.1|g.14335	-1.44	5.87	25.92	0.0004	0.0214
24 hours	twi_ss.30481.1|g.41261	0.71	9.16	25.89	0.0004	0.0215

24 hours	twi_ss.28736.1|g.38496	-0.77	6.41	25.85	0.0004	0.0216
24 hours	twi_ss.3049.4|g.5406	-1.41	7.94	25.83	0.0004	0.0216
24 hours	twi_ss.6189.1|g.9149	1.39	7.12	25.79	0.0004	0.0217
24 hours	twi_ss.29938.1|g.40363	-0.86	6.62	25.78	0.0004	0.0217
24 hours	twi_ss.20032.1|g.25789	-1.96	6.07	25.74	0.0004	0.0218
24 hours	twi_ss.12283.1|g.16263	1.32	5.76	25.73	0.0004	0.0218
24 hours	twi_ss.5305.2|g.7948	1.81	6.12	25.66	0.0004	0.0220
24 hours	twi_ss.26361b.4|g.34823	-3.89	7.24	25.63	0.0004	0.0220
24 hours	twi_ss.5433b.1|g.8210	-1.42	6.33	25.61	0.0004	0.0220
24 hours	twi_ss.22780.1|g.29402	-1.31	5.65	25.62	0.0004	0.0220
24 hours	twi_ss.21393.1|g.27680	1.72	6.75	25.64	0.0004	0.0220
24 hours	twi_ss.2565.1|g.4595	-2.43	5.04	25.58	0.0004	0.0220
24 hours	twi_ss.19219.2|g.24607	-7.54	5.27	25.57	0.0004	0.0220
24 hours	twi_ss.8819.1|g.12011	6.15	3.28	25.48	0.0004	0.0222
24 hours	twi_ss.4192.1|g.6605	-3.32	5.26	25.49	0.0004	0.0222
24 hours	twi_ss.29908.1|g.40336	-1.45	4.69	25.50	0.0004	0.0222
24 hours	twi_ss.10318.1|g.13805	-2.85	6.78	25.42	0.0004	0.0224
24 hours	twi_ss.29486.2|g.39693	-2.43	4.38	25.37	0.0005	0.0225
24 hours	twi_ss.22899.1|g.29570	-2.35	5.52	25.33	0.0005	0.0226
24 hours	twi_ss.26267.1|g.34786	1.15	5.96	25.33	0.0005	0.0226
24 hours	twi_ss.18548.1|g.23653	-2.10	5.37	25.34	0.0005	0.0226
24 hours	twi_ss.20029.1|g.25796	-1.67	4.78	25.30	0.0005	0.0226
24 hours	twi_ss.30565a.6|g.41425	-1.96	8.28	25.30	0.0005	0.0226
24 hours	twi_ss.10837.3|g.14465	-1.68	6.52	25.28	0.0005	0.0226
24 hours	twi_ss.31525.1|g.42924	-1.91	4.71	25.25	0.0005	0.0226
24 hours	twi_ss.26402.1|g.34995	0.84	6.93	25.25	0.0005	0.0226
24 hours	twi_ss.10747a.2|g.14392	-2.03	5.71	25.20	0.0005	0.0227
24 hours	twi_ss.5530.1|g.8370	-1.96	5.16	25.16	0.0005	0.0227
24 hours	twi_ss.26810.1|g.35505	-3.54	4.04	25.16	0.0005	0.0227
24 hours	twi_ss.28282.1|g.37778	0.80	6.72	25.18	0.0005	0.0227
24 hours	twi_ss.16751a.2|g.21416	0.80	8.72	25.17	0.0005	0.0227
24 hours	twi_ss.5423.1|g.8189	-0.84	5.99	25.18	0.0005	0.0227
24 hours	twi_ss.1961.1|g.3439	-1.36	5.48	25.15	0.0005	0.0227

24 hours	twi_ss.22168.1|g.28705	-2.22	4.85	25.09	0.0005	0.0229
24 hours	twi_ss.5087.1|g.7753	-6.58	4.31	30.49	0.0005	0.0229
24 hours	twi_ss.1961.2|g.3438	-1.04	5.64	25.03	0.0005	0.0230
24 hours	twi_ss.6152b.2|g.9126	1.29	6.76	25.02	0.0005	0.0230
24 hours	twi_ss.13116b.1|g.17388	1.48	8.13	24.98	0.0005	0.0231
24 hours	twi_ss.6498.1|g.9563	-2.07	4.74	24.96	0.0005	0.0231
24 hours	twi_ss.10802.1|g.14436	1.69	4.66	24.97	0.0005	0.0231
24 hours	twi_ss.21978.1|g.28482	-3.93	3.45	24.95	0.0005	0.0231
24 hours	twi_ss.19617b.2|g.25195	1.66	5.13	24.90	0.0005	0.0232
24 hours	twi_ss.31743.1|g.43225	-1.75	5.86	24.91	0.0005	0.0232
24 hours	twi_ss.28429.1|g.38122	2.67	5.57	24.88	0.0005	0.0233
24 hours	twi_ss.31358.1|g.42626	-1.79	5.99	24.84	0.0005	0.0234
24 hours	twi_ss.17695.1|g.22566	-2.38	3.55	24.75	0.0005	0.0236
24 hours	twi_ss.26448.1|g.35056	0.75	8.38	24.75	0.0005	0.0236
24 hours	twi_ss.8754.1|g.11980	-0.88	7.04	24.74	0.0005	0.0236
24 hours	twi_ss.31830.1|g.43358	0.76	7.46	24.72	0.0005	0.0236
24 hours	twi_ss.18290.1|g.23321	-4.80	2.86	24.66	0.0005	0.0238
24 hours	twi_ss.14763.1|g.19250	-1.04	6.23	24.60	0.0005	0.0240
24 hours	twi_ss.21975.1|g.28476	1.36	5.77	24.58	0.0005	0.0240
24 hours	twi_ss.31441.1|g.42801	1.99	6.16	24.56	0.0005	0.0240
24 hours	twi_ss.31159.1|g.42402	0.65	7.87	24.56	0.0005	0.0240
24 hours	twi_ss.17303.1|g.22085	-1.62	11.19	24.55	0.0005	0.0240
24 hours	twi_ss.22414.1|g.28958	-2.67	4.92	24.48	0.0005	0.0243
24 hours	twi_ss.2601.1|g.4625	0.92	6.38	24.46	0.0005	0.0243
24 hours	twi_ss.8819.2|g.12010	-6.21	2.83	24.41	0.0005	0.0244
24 hours	twi_ss.20330.1|g.26166	0.85	6.18	24.42	0.0005	0.0244
24 hours	twi_ss.24252.2|g.31673	0.95	6.45	24.40	0.0005	0.0244
24 hours	twi_ss.19415.1|g.24943	3.99	4.02	24.37	0.0005	0.0245
24 hours	twi_ss.15807.1|g.20367	-6.47	3.45	24.37	0.0005	0.0245
24 hours	twi_ss.24730.1|g.32444	1.84	6.34	24.31	0.0005	0.0247
24 hours	twi_ss.15213.1|g.19802	-1.70	5.49	24.29	0.0005	0.0247
24 hours	twi_ss.11436.1|g.15091	-1.20	6.93	24.27	0.0005	0.0247
24 hours	twi_ss.2790.1|g.5020	0.80	6.34	24.26	0.0005	0.0247

24 hours	twi_ss.27562.2|g.36725	3.81	7.95	24.25	0.0005	0.0247
24 hours	twi_ss.30158.1|g.40726	0.84	7.49	24.24	0.0005	0.0247
24 hours	twi_ss.20587.1|g.26691	-3.13	4.40	24.21	0.0005	0.0248
24 hours	twi_ss.9923.1|g.13253	0.67	7.65	24.19	0.0005	0.0248
24 hours	twi_ss.5977.2|g.8846	-2.53	3.79	24.20	0.0005	0.0248
24 hours	twi_ss.18776.1|g.23925	-0.95	5.46	24.13	0.0005	0.0248
24 hours	twi_ss.4304a.1|g.6726	2.26	5.36	24.13	0.0005	0.0248
24 hours	twi_ss.5133.1|g.7779	-4.51	3.46	24.16	0.0005	0.0248
24 hours	twi_ss.18744.1|g.23953	-1.94	5.05	24.13	0.0005	0.0248
24 hours	twi_ss.7208.1|g.10259	-2.08	5.49	24.16	0.0005	0.0248
24 hours	twi_ss.18626b.1|g.23809	-3.85	4.14	24.13	0.0005	0.0248
24 hours	twi_ss.6244b.3|g.9269	-2.64	3.63	24.10	0.0005	0.0249
24 hours	twi_ss.8873.1|g.12071	-1.75	9.97	24.08	0.0005	0.0249
24 hours	twi_ss.19834.1|g.25530	-2.54	6.64	24.02	0.0006	0.0250
24 hours	twi_ss.23297.1|g.29984	1.32	6.26	24.02	0.0006	0.0250
24 hours	twi_ss.22735.1|g.29345	-2.19	4.67	24.02	0.0006	0.0250
24 hours	twi_ss.2269.2|g.4022	-2.20	4.47	24.02	0.0006	0.0250
24 hours	twi_ss.16157.1|g.20755	-4.79	3.98	23.93	0.0006	0.0253
24 hours	twi_ss.23834.1|g.30873	1.37	7.71	23.94	0.0006	0.0253
24 hours	twi_ss.8322.1|g.11421	-2.48	3.98	23.81	0.0006	0.0256
24 hours	twi_ss.10023.1|g.13343	1.14	5.13	23.81	0.0006	0.0256
24 hours	twi_ss.22439.1|g.29005	-8.06	4.24	23.82	0.0006	0.0256
24 hours	twi_ss.1817b.1|g.3214	-1.54	6.61	23.81	0.0006	0.0256
24 hours	twi_ss.8634.1|g.11782	-1.92	4.44	23.79	0.0006	0.0257
24 hours	twi_ss.20961a.1|g.27085	1.26	6.27	23.78	0.0006	0.0257
24 hours	twi_ss.19004.1|g.24419	-2.61	4.53	23.74	0.0006	0.0258
24 hours	twi_ss.21016.1|g.27185	1.09	6.25	23.69	0.0006	0.0260
24 hours	twi_ss.22218e.7|g.28734	6.86	4.53	28.51	0.0006	0.0262
24 hours	twi_ss.28816.1|g.38576	-3.66	4.75	23.60	0.0006	0.0263
24 hours	twi_ss.31409.1|g.42778	-2.58	4.09	23.56	0.0006	0.0263
24 hours	twi_ss.23257.1|g.29910	-1.55	4.82	23.56	0.0006	0.0263
24 hours	twi_ss.29955.1|g.40412	0.97	5.48	23.57	0.0006	0.0263
24 hours	twi_ss.25137.1|g.33020	1.30	9.36	23.56	0.0006	0.0263

24 hours	twi_ss.15844b.3|g.20393	5.80	3.96	23.54	0.0006	0.0263
24 hours	twi_ss.15661.2|g.20228	1.93	5.75	23.49	0.0006	0.0265
24 hours	twi_ss.5357.1|g.8107	1.06	6.95	23.49	0.0006	0.0265
24 hours	twi_ss.31516.1|g.42907	-2.09	4.78	23.46	0.0006	0.0265
24 hours	twi_ss.28509b.1|g.38043	-1.48	7.20	23.45	0.0006	0.0266
24 hours	twi_ss.31286.2|g.42540	-8.64	4.66	23.43	0.0006	0.0266
24 hours	twi_ss.29672.3|g.40009	-1.74	4.70	23.42	0.0006	0.0266
24 hours	twi_ss.12872.1|g.17046	-4.80	3.48	23.39	0.0006	0.0267
24 hours	twi_ss.23142.2|g.29796	-1.36	6.61	23.35	0.0006	0.0268
24 hours	twi_ss.10058.3|g.13419	-5.84	2.98	23.32	0.0006	0.0269
24 hours	twi_ss.26662.1|g.35468	-3.56	4.06	23.27	0.0006	0.0271
24 hours	twi_ss.21800.1|g.28295	-1.42	5.80	23.27	0.0006	0.0271
24 hours	twi_ss.1037.1|g.1879	-1.79	3.93	23.20	0.0006	0.0273
24 hours	twi_ss.16987.1|g.21688	-2.27	5.42	23.17	0.0006	0.0274
24 hours	twi_ss.24825.1|g.32667	-1.35	5.13	23.17	0.0006	0.0274
24 hours	twi_ss.28797.1|g.38582	-4.90	2.58	27.81	0.0006	0.0275
24 hours	twi_ss.28424.1|g.38003	1.61	6.85	23.11	0.0006	0.0275
24 hours	twi_ss.19776.2|g.25452	0.77	7.26	23.11	0.0006	0.0275
24 hours	twi_ss.7711.1|g.10773	1.53	5.80	23.06	0.0006	0.0277
24 hours	twi_ss.21704.3|g.28113	6.57	2.95	23.00	0.0007	0.0279
24 hours	twi_ss.3480b.2|g.5863	-0.86	7.73	23.00	0.0007	0.0279
24 hours	twi_ss.20545.1|g.26514	-1.35	5.39	23.00	0.0007	0.0279
24 hours	twi_ss.14839.1|g.19294	6.03	3.62	22.97	0.0007	0.0279
24 hours	twi_ss.22776.1|g.29409	0.99	6.33	22.96	0.0007	0.0279
24 hours	twi_ss.14119.1|g.18425	2.65	4.54	22.97	0.0007	0.0279
24 hours	twi_ss.10467.1|g.14103	1.10	5.97	22.96	0.0007	0.0279
24 hours	twi_ss.6682.1|g.9749	1.09	7.81	22.95	0.0007	0.0279
24 hours	twi_ss.20081.1|g.25853	0.84	6.31	22.94	0.0007	0.0279
24 hours	twi_ss.25510.1|g.33545	1.66	4.91	22.90	0.0007	0.0279
24 hours	twi_ss.29126.1|g.39032	3.86	4.25	22.90	0.0007	0.0279
24 hours	twi_ss.5452.1|g.8239	-1.48	5.41	22.90	0.0007	0.0279
24 hours	twi_ss.24905.1|g.32762	-4.01	3.63	22.92	0.0007	0.0279
24 hours	twi_ss.23880.1|g.31029	-3.83	11.32	22.85	0.0007	0.0280

24 hours	twi_ss.24236.1|g.31566	-3.72	11.20	22.84	0.0007	0.0280
24 hours	twi_ss.16258.1|g.20833	-2.53	5.05	22.86	0.0007	0.0280
24 hours	twi_ss.5199.1|g.7825	-2.31	4.95	22.79	0.0007	0.0283
24 hours	twi_ss.3592.1|g.5939	0.83	9.61	22.78	0.0007	0.0283
24 hours	twi_ss.28999a.2|g.38913	-4.51	2.66	22.76	0.0007	0.0283
24 hours	twi_ss.11565.12|g.15312	-6.90	5.17	22.75	0.0007	0.0283
24 hours	twi_ss.18584.1|g.23663	7.89	4.22	22.74	0.0007	0.0283
24 hours	twi_ss.18596b.2|g.23770	-5.74	4.17	22.72	0.0007	0.0284
24 hours	twi_ss.20410.1|g.26292	-4.16	3.46	27.28	0.0007	0.0284
24 hours	twi_ss.1003.1|g.1833	0.93	6.19	22.71	0.0007	0.0284
24 hours	twi_ss.24245.1|g.31625	-3.92	10.63	22.70	0.0007	0.0284
24 hours	twi_ss.670.1|g.1159	-1.20	5.90	22.68	0.0007	0.0284
24 hours	twi_ss.7349.1|g.10353	-2.78	6.25	22.65	0.0007	0.0284
24 hours	twi_ss.13440.1|g.17763	0.67	7.10	22.63	0.0007	0.0284
24 hours	twi_ss.28506.9|g.38184	-6.14	2.65	37.21	0.0007	0.0284
24 hours	twi_ss.28156a.1|g.37511	-5.28	6.91	27.17	0.0007	0.0284
24 hours	twi_ss.5201.2|g.7830	-1.41	6.34	22.64	0.0007	0.0284
24 hours	twi_ss.10298a.1|g.13815	0.50	8.79	22.58	0.0007	0.0287
24 hours	twi_ss.24304.3|g.31689	1.30	7.50	22.55	0.0007	0.0287
24 hours	twi_ss.7759.1|g.10821	-1.02	6.68	22.55	0.0007	0.0287
24 hours	twi_ss.30027a.5|g.40531	6.26	4.96	22.53	0.0007	0.0288
24 hours	twi_ss.20467.1|g.26380	-1.60	5.78	22.50	0.0007	0.0288
24 hours	twi_ss.11197.1|g.14847	-1.44	5.94	22.46	0.0007	0.0290
24 hours	twi_ss.29475.1|g.39689	-2.62	7.92	22.43	0.0007	0.0291
24 hours	twi_ss.1441.1|g.2632	1.05	5.51	22.41	0.0007	0.0292
24 hours	twi_ss.1034.1|g.1887	-3.93	8.05	22.35	0.0007	0.0294
24 hours	twi_ss.8091.1|g.11167	-0.54	7.41	22.29	0.0007	0.0295
24 hours	twi_ss.13681.2|g.18027	-5.59	2.47	22.29	0.0007	0.0295
24 hours	twi_ss.827.1|g.1458	1.03	6.07	22.31	0.0007	0.0295
24 hours	twi_ss.13102a.1|g.17415	1.24	9.23	22.30	0.0007	0.0295
24 hours	twi_ss.1543.4|g.2786	6.99	3.88	22.29	0.0007	0.0295
24 hours	twi_ss.13471.2|g.17804	1.17	6.04	22.32	0.0007	0.0295
24 hours	twi_ss.21301.1|g.27540	-1.79	4.58	22.26	0.0007	0.0296

24 hours	twi_ss.29868.1|g.40338	-7.85	3.81	26.66	0.0007	0.0296
24 hours	twi_ss.18630.1|g.23736	-0.84	9.37	22.23	0.0007	0.0296
24 hours	twi_ss.15787.1|g.20355	-1.75	6.60	22.21	0.0007	0.0297
24 hours	twi_ss.25062.2|g.32886	-1.30	4.72	22.20	0.0007	0.0297
24 hours	twi_ss.4279.1|g.6783	-1.84	6.30	22.20	0.0007	0.0297
24 hours	twi_ss.969.1|g.1738	-2.14	3.90	22.20	0.0007	0.0297
24 hours	twi_ss.4859.1|g.7471	0.57	8.15	22.15	0.0007	0.0298
24 hours	twi_ss.20877.1|g.27012	-2.27	3.96	22.12	0.0008	0.0299
24 hours	twi_ss.27268a.1|g.36251	-1.07	7.46	22.10	0.0008	0.0300
24 hours	twi_ss.28938.1|g.38803	-2.19	4.93	22.04	0.0008	0.0303
24 hours	twi_ss.28808.1|g.38601	-3.21	4.54	22.01	0.0008	0.0304
24 hours	twi_ss.12294.1|g.16393	0.83	6.99	22.01	0.0008	0.0304
24 hours	twi_ss.26905.1|g.35685	1.47	5.60	21.96	0.0008	0.0306
24 hours	twi_ss.17277a.22|g.22028	1.35	5.71	21.95	0.0008	0.0306
24 hours	twi_ss.3456.1|g.5831	-0.72	6.62	21.94	0.0008	0.0306
24 hours	twi_ss.31377.1|g.42709	-1.90	5.70	21.90	0.0008	0.0307
24 hours	twi_ss.10703.1|g.14317	-1.76	5.11	21.90	0.0008	0.0307
24 hours	twi_ss.7261.1|g.10294	-4.90	2.29	21.90	0.0008	0.0307
24 hours	twi_ss.17334a.1|g.22135	-1.57	6.74	21.86	0.0008	0.0308
24 hours	twi_ss.26361a.1|g.34928	-4.28	6.15	26.08	0.0008	0.0310
24 hours	twi_ss.12447.4|g.16561	5.27	2.47	21.82	0.0008	0.0310
24 hours	twi_ss.19701b.2|g.25349	-0.89	8.39	21.79	0.0008	0.0310
24 hours	twi_ss.16245.2|g.20824	-1.28	5.60	21.79	0.0008	0.0310
24 hours	twi_ss.21349.1|g.27560	-3.87	8.36	21.80	0.0008	0.0310
24 hours	twi_ss.29230.1|g.39192	-2.34	3.63	21.78	0.0008	0.0310
24 hours	twi_ss.29971.4|g.40439	1.01	6.26	21.75	0.0008	0.0311
24 hours	twi_ss.29971.5|g.40444	1.01	6.26	21.75	0.0008	0.0311
24 hours	twi_ss.20387.1|g.26195	0.69	7.49	21.76	0.0008	0.0311
24 hours	twi_ss.12226.1|g.16336	2.26	6.55	21.68	0.0008	0.0314
24 hours	twi_ss.29980.2|g.40459	-1.98	5.07	21.65	0.0008	0.0315
24 hours	twi_ss.24228.1|g.31598	0.74	8.99	21.65	0.0008	0.0315
24 hours	twi_ss.925.5|g.1614	-4.28	4.20	21.64	0.0008	0.0315
24 hours	twi_ss.31729.1|g.43227	2.07	4.47	21.63	0.0008	0.0315

24 hours	twi_ss.22588a.2|g.29168	-1.46	5.11	21.59	0.0008	0.0316
24 hours	twi_ss.17839.1|g.22730	-1.13	9.85	21.59	0.0008	0.0316
24 hours	twi_ss.16144.1|g.20741	-1.09	8.99	21.54	0.0008	0.0318
24 hours	twi_ss.22097.1|g.28604	-2.03	4.45	21.52	0.0008	0.0318
24 hours	twi_ss.4253.1|g.6682	-2.13	4.31	21.52	0.0008	0.0318
24 hours	twi_ss.31453.1|g.42823	-1.54	7.75	21.52	0.0008	0.0318
24 hours	twi_ss.4400.1|g.6899	-2.97	3.85	21.53	0.0008	0.0318
24 hours	twi_ss.24710.1|g.32405	-3.09	4.52	21.44	0.0008	0.0321
24 hours	twi_ss.18747.1|g.23950	-1.58	4.90	21.45	0.0008	0.0321
24 hours	twi_ss.16353.1|g.20942	-2.30	6.87	25.59	0.0008	0.0321
24 hours	twi_ss.26704b.1|g.35420	0.93	10.59	21.43	0.0008	0.0321
24 hours	twi_ss.21473.1|g.27813	1.32	5.92	21.41	0.0008	0.0322
24 hours	twi_ss.19359.1|g.24865	0.71	6.55	21.37	0.0009	0.0322
24 hours	twi_ss.21051.1|g.27249	3.65	3.47	21.38	0.0009	0.0322
24 hours	twi_ss.6802.1|g.9869	-1.44	4.24	21.38	0.0009	0.0322
24 hours	twi_ss.21453b.2|g.27711	-2.66	4.85	21.39	0.0008	0.0322
24 hours	twi_ss.7286.1|g.10310	-0.78	5.48	21.37	0.0009	0.0322
24 hours	twi_ss.888.2|g.1581	-5.07	2.60	25.46	0.0009	0.0323
24 hours	twi_ss.24933b.4|g.32801	-1.01	5.89	21.32	0.0009	0.0324
24 hours	twi_ss.24989.1|g.32831	-4.53	2.40	25.43	0.0009	0.0324
24 hours	twi_ss.18618.1|g.23814	-2.62	5.20	21.27	0.0009	0.0326
24 hours	twi_ss.31444.1|g.42805	1.69	9.81	21.25	0.0009	0.0327
24 hours	twi_ss.4881.1|g.7484	-1.26	8.71	21.24	0.0009	0.0327
24 hours	twi_ss.30173.6|g.40745	-1.37	5.06	21.22	0.0009	0.0327
24 hours	twi_ss.19594.1|g.25172	-0.92	6.66	21.22	0.0009	0.0327
24 hours	twi_ss.14467.1|g.18875	0.67	7.53	21.16	0.0009	0.0328
24 hours	twi_ss.16756.1|g.21449	-1.41	5.11	21.12	0.0009	0.0328
24 hours	twi_ss.24352c.3|g.31772	7.07	4.34	33.98	0.0009	0.0328
24 hours	twi_ss.8902.2|g.12085	4.68	2.03	21.16	0.0009	0.0328
24 hours	twi_ss.30095.1|g.40635	1.14	6.19	21.16	0.0009	0.0328
24 hours	twi_ss.469.1|g.851	-1.43	5.67	21.14	0.0009	0.0328
24 hours	twi_ss.5796.2|g.8652	-6.33	5.59	21.12	0.0009	0.0328
24 hours	twi_ss.1421.1|g.2545	-1.32	5.86	21.14	0.0009	0.0328

24 hours	twi_ss.12318.1|g.16442	-1.11	6.49	21.11	0.0009	0.0328
24 hours	twi_ss.5143.1|g.7782	-1.56	5.55	21.15	0.0009	0.0328
24 hours	twi_ss.7858.12|g.10915	3.93	6.25	21.15	0.0009	0.0328
24 hours	twi_ss.31899b.1|g.43490	5.72	2.54	21.15	0.0009	0.0328
24 hours	twi_ss.6472.1|g.9500	0.75	9.46	21.11	0.0009	0.0328
24 hours	twi_ss.30850.1|g.42013	0.94	5.54	20.99	0.0009	0.0334
24 hours	twi_ss.10009.1|g.13306	-1.02	5.38	20.97	0.0009	0.0335
24 hours	twi_ss.31838.1|g.43349	-2.41	3.41	20.98	0.0009	0.0335
24 hours	twi_ss.8150.1|g.11239	1.15	6.70	20.97	0.0009	0.0335
24 hours	twi_ss.6345.1|g.9359	0.56	7.13	20.96	0.0009	0.0335
24 hours	twi_ss.28647.1|g.38357	0.66	8.32	20.94	0.0009	0.0335
24 hours	twi_ss.24311.2|g.31768	7.22	3.26	20.93	0.0009	0.0336
24 hours	twi_ss.8361a.1|g.11511	0.58	7.31	20.91	0.0009	0.0336
24 hours	twi_ss.2795.1|g.5006	0.96	6.49	20.91	0.0009	0.0336
24 hours	twi_ss.25634b.10|g.33766	-3.72	3.42	24.81	0.0009	0.0339
24 hours	twi_ss.20867.1|g.26998	-2.93	4.96	20.85	0.0009	0.0339
24 hours	twi_ss.26881.1|g.35643	-2.16	4.80	20.79	0.0009	0.0341
24 hours	twi_ss.437.1|g.823	0.76	9.22	20.79	0.0009	0.0341
24 hours	twi_ss.16656.2|g.21332	7.26	7.44	20.80	0.0009	0.0341
24 hours	twi_ss.1168.1|g.2027	1.37	6.63	20.79	0.0009	0.0341
24 hours	twi_ss.25299a.1|g.33316	-0.89	7.03	20.75	0.0009	0.0343
24 hours	twi_ss.19282.2|g.24761	5.64	2.41	20.73	0.0010	0.0343
24 hours	twi_ss.3944.1|g.6399	1.37	6.88	20.72	0.0010	0.0343
24 hours	twi_ss.18500.1|g.23592	-1.15	7.56	20.72	0.0010	0.0343
24 hours	twi_ss.811.1|g.1442	-2.33	4.06	20.69	0.0010	0.0344
24 hours	twi_ss.14427.1|g.18832	-1.34	4.89	20.66	0.0010	0.0346
24 hours	twi_ss.29653.1|g.39982	-2.04	4.08	20.61	0.0010	0.0348
24 hours	twi_ss.6207.1|g.9205	0.77	7.63	20.58	0.0010	0.0350
24 hours	twi_ss.1424.2|g.2514	-7.56	3.74	20.56	0.0010	0.0351
24 hours	twi_ss.23723.1|g.30643	1.68	6.10	20.51	0.0010	0.0354
24 hours	twi_ss.20099.1|g.25857	-4.76	2.06	32.65	0.0010	0.0354
24 hours	twi_ss.17252.1|g.22048	1.42	6.96	20.48	0.0010	0.0354
24 hours	twi_ss.19384.1|g.24874	8.66	6.19	24.30	0.0010	0.0355

24 hours	twi_ss.31154.2|g.42361	7.17	3.37	20.43	0.0010	0.0357
24 hours	twi_ss.10965.1|g.14538	1.08	12.07	20.42	0.0010	0.0357
24 hours	twi_ss.25606.1|g.33725	-1.49	5.87	20.37	0.0010	0.0360
24 hours	twi_ss.30923.2|g.41912	4.13	1.88	20.33	0.0010	0.0361
24 hours	twi_ss.21577.1|g.27967	-2.57	4.21	20.34	0.0010	0.0361
24 hours	twi_ss.19960b.5|g.25659	-3.31	5.06	24.00	0.0010	0.0363
24 hours	twi_ss.27272.1|g.36217	-2.78	3.63	20.25	0.0010	0.0363
24 hours	twi_ss.24526c.3|g.32158	-2.98	5.14	20.27	0.0010	0.0363
24 hours	twi_ss.26088.1|g.34578	-1.08	5.60	20.27	0.0010	0.0363
24 hours	twi_ss.4977.8|g.7536	-2.41	5.84	20.25	0.0010	0.0363
24 hours	twi_ss.30134.4|g.40703	3.26	8.64	20.27	0.0010	0.0363
24 hours	twi_ss.13114.1|g.17313	-2.04	4.02	20.26	0.0010	0.0363
24 hours	twi_ss.14576.1|g.19038	-2.89	6.88	20.25	0.0010	0.0363
24 hours	twi_ss.30983a.1|g.41800	1.85	6.46	20.23	0.0010	0.0364
24 hours	twi_ss.30834b.2|g.42027	1.30	6.00	20.23	0.0010	0.0364
24 hours	twi_ss.21156.2|g.27391	-4.88	2.66	23.93	0.0010	0.0365
24 hours	twi_ss.21611.1|g.28009	-0.79	7.71	20.17	0.0011	0.0365
24 hours	twi_ss.1818.2|g.3205	-1.55	5.72	20.16	0.0011	0.0365
24 hours	twi_ss.8930b.1|g.12126	-1.53	5.19	20.16	0.0011	0.0365
24 hours	twi_ss.2663.1|g.4820	0.97	6.80	20.18	0.0010	0.0365
24 hours	twi_ss.21329.1|g.27617	6.77	4.10	20.16	0.0011	0.0365
24 hours	twi_ss.27031.1|g.35920	1.13	7.00	20.17	0.0011	0.0365
24 hours	twi_ss.10882.1|g.14512	2.40	9.11	20.14	0.0011	0.0366
24 hours	twi_ss.18736.1|g.23916	0.91	7.13	20.12	0.0011	0.0367
24 hours	twi_ss.2352.1|g.4268	0.56	8.42	20.10	0.0011	0.0368
24 hours	twi_ss.1489.2|g.2552	-6.89	3.14	20.08	0.0011	0.0368
24 hours	twi_ss.1343.1|g.2422	0.42	8.24	20.08	0.0011	0.0368
24 hours	twi_ss.3480b.3|g.5862	-7.09	3.31	20.07	0.0011	0.0368
24 hours	twi_ss.1977.1|g.3449	0.52	9.41	20.06	0.0011	0.0369
24 hours	twi_ss.9661.1|g.12991	0.85	6.09	20.02	0.0011	0.0370
24 hours	twi_ss.7858.13|g.10902	1.80	6.11	20.02	0.0011	0.0370
24 hours	twi_ss.23881.2|g.31020	1.22	4.95	20.00	0.0011	0.0371
24 hours	twi_ss.24661.1|g.32358	1.00	5.48	19.98	0.0011	0.0372

24 hours	twi_ss.6781.1|g.9766	1.18	6.59	19.91	0.0011	0.0377
24 hours	twi_ss.12822.1|g.16932	-1.62	4.94	19.87	0.0011	0.0378
24 hours	twi_ss.28579.4|g.38269	1.36	7.55	19.88	0.0011	0.0378
24 hours	twi_ss.17793a.3|g.22692	-0.78	6.38	19.87	0.0011	0.0378
24 hours	twi_ss.16705.1|g.21381	-4.53	5.42	19.85	0.0011	0.0379
24 hours	twi_ss.2005.1|g.3565	1.08	5.20	19.81	0.0011	0.0380
24 hours	twi_ss.2005.2|g.3573	1.08	5.20	19.81	0.0011	0.0380
24 hours	twi_ss.2005.3|g.3577	1.08	5.20	19.81	0.0011	0.0380
24 hours	twi_ss.4127.1|g.6552	-1.79	5.25	19.79	0.0011	0.0381
24 hours	twi_ss.11132.1|g.14796	0.97	5.79	19.77	0.0011	0.0382
24 hours	twi_ss.10701.1|g.14357	1.03	5.89	19.75	0.0011	0.0383
24 hours	twi_ss.3645.1|g.6020	0.61	7.42	19.74	0.0011	0.0383
24 hours	twi_ss.14866.1|g.19312	-0.84	7.60	19.73	0.0011	0.0383
24 hours	twi_ss.16791.1|g.21460	-3.06	3.44	19.73	0.0011	0.0383
24 hours	twi_ss.3623.1|g.5992	5.71	3.53	19.73	0.0011	0.0383
24 hours	twi_ss.22596.1|g.29198	-4.58	2.50	23.28	0.0011	0.0385
24 hours	twi_ss.16580.1|g.21169	0.65	9.36	19.69	0.0011	0.0385
24 hours	twi_ss.23644.1|g.30455	0.73	7.35	19.67	0.0011	0.0386
24 hours	twi_ss.2348.1|g.4257	-3.24	4.12	19.62	0.0012	0.0389
24 hours	twi_ss.25312.2|g.33268	-2.05	4.57	19.59	0.0012	0.0391
24 hours	twi_ss.11378.4|g.14981	-5.79	3.57	23.09	0.0012	0.0392
24 hours	twi_ss.12874.1|g.17069	-0.84	7.27	19.54	0.0012	0.0393
24 hours	twi_ss.29991.1|g.40468	0.85	5.51	19.54	0.0012	0.0393
24 hours	twi_ss.31144.1|g.42355	-1.45	5.39	19.54	0.0012	0.0393
24 hours	twi_ss.22665.1|g.29268	-2.37	4.18	19.52	0.0012	0.0393
24 hours	twi_ss.25893.1|g.34159	-1.38	5.79	19.51	0.0012	0.0393
24 hours	twi_ss.28999a.1|g.38914	4.70	2.05	19.51	0.0012	0.0393
24 hours	twi_ss.22200.1|g.28758	-1.66	5.33	19.50	0.0012	0.0394
24 hours	twi_ss.17485.1|g.22307	-1.06	6.02	19.49	0.0012	0.0394
24 hours	twi_ss.29717.1|g.40099	-5.12	4.13	19.48	0.0012	0.0394
24 hours	twi_ss.6134.2|g.9005	1.11	5.85	19.47	0.0012	0.0394
24 hours	twi_ss.493.1|g.898	0.83	6.48	19.47	0.0012	0.0394
24 hours	twi_ss.28144.1|g.37496	-1.20	5.42	19.44	0.0012	0.0395

24 hours	twi_ss.20085.1|g.25836	-1.83	4.83	19.44	0.0012	0.0395
24 hours	twi_ss.17494.1|g.22269	1.11	6.54	19.43	0.0012	0.0395
24 hours	twi_ss.17326.6|g.22117	8.17	4.08	19.41	0.0012	0.0396
24 hours	twi_ss.22556.1|g.29135	-1.57	7.02	22.85	0.0012	0.0398
24 hours	twi_ss.4226.1|g.6652	0.68	8.06	19.37	0.0012	0.0398
24 hours	twi_ss.21425.2|g.27686	3.79	4.31	19.35	0.0012	0.0398
24 hours	twi_ss.30578.1|g.41416	-0.72	7.54	19.35	0.0012	0.0398
24 hours	twi_ss.20449b.1|g.26353	-3.18	4.07	19.35	0.0012	0.0398
24 hours	twi_ss.10199.1|g.13714	1.66	4.62	19.34	0.0012	0.0399
24 hours	twi_ss.21314.1|g.27582	1.23	5.25	19.32	0.0012	0.0399
24 hours	twi_ss.849.1|g.1397	-0.96	5.99	19.33	0.0012	0.0399
24 hours	twi_ss.5273.1|g.7993	-4.25	10.33	22.77	0.0012	0.0400
24 hours	twi_ss.23624.2|g.30356	-5.95	3.31	19.25	0.0012	0.0404
24 hours	twi_ss.24304.2|g.31685	0.90	12.39	19.23	0.0012	0.0405
24 hours	twi_ss.19070a.4|g.24462	1.11	6.00	19.23	0.0012	0.0405
24 hours	twi_ss.27706.1|g.36891	0.71	6.56	19.21	0.0012	0.0406
24 hours	twi_ss.4977.2|g.7539	-6.02	3.31	22.58	0.0013	0.0407
24 hours	twi_ss.24280.1|g.31710	-3.11	3.29	19.17	0.0013	0.0407
24 hours	twi_ss.9747.1|g.13086	0.62	6.43	19.18	0.0013	0.0407
24 hours	twi_ss.8278.1|g.11392	-3.46	3.09	19.18	0.0013	0.0407
24 hours	twi_ss.12332.9|g.16421	7.51	5.36	19.17	0.0013	0.0407
24 hours	twi_ss.31134.2|g.42327	-6.31	3.92	19.14	0.0013	0.0408
24 hours	twi_ss.24497.2|g.32088	2.71	5.08	19.10	0.0013	0.0411
24 hours	twi_ss.31636.1|g.43103	1.15	6.90	19.09	0.0013	0.0411
24 hours	twi_ss.17140.1|g.21918	-1.73	4.50	19.05	0.0013	0.0413
24 hours	twi_ss.3981.1|g.6392	-1.69	4.33	19.04	0.0013	0.0414
24 hours	twi_ss.22264.1|g.28821	-2.23	5.59	19.03	0.0013	0.0414
24 hours	twi_ss.6018.1|g.8879	4.87	6.32	19.03	0.0013	0.0414
24 hours	twi_ss.23710.1|g.30738	0.72	7.86	18.98	0.0013	0.0416
24 hours	twi_ss.25600.1|g.33726	0.85	5.41	18.98	0.0013	0.0416
24 hours	twi_ss.8447.2|g.11635	-1.37	5.62	18.97	0.0013	0.0416
24 hours	twi_ss.258.2|g.525	5.43	2.69	18.97	0.0013	0.0416
24 hours	twi_ss.21155.1|g.27367	0.62	8.77	18.99	0.0013	0.0416

24 hours	twi_ss.24627.2|g.32262	5.80	3.56	18.96	0.0013	0.0417
24 hours	twi_ss.18368.5|g.23387	6.31	2.65	18.93	0.0013	0.0418
24 hours	twi_ss.17564a.2|g.22358	0.80	6.57	18.93	0.0013	0.0418
24 hours	twi_ss.28187.1|g.37571	-3.13	5.82	18.92	0.0013	0.0418
24 hours	twi_ss.13607.1|g.17960	0.71	6.49	18.92	0.0013	0.0418
24 hours	twi_ss.16733.1|g.21393	0.75	7.00	18.91	0.0013	0.0418
24 hours	twi_ss.10122.1|g.13573	-3.81	11.62	18.85	0.0013	0.0420
24 hours	twi_ss.1295.2|g.2339	4.06	1.86	19.72	0.0013	0.0420
24 hours	twi_ss.14343.4|g.18656	-1.26	5.33	18.88	0.0013	0.0420
24 hours	twi_ss.4757.1|g.7349	0.92	6.13	18.85	0.0013	0.0420
24 hours	twi_ss.13862.1|g.18177	1.33	4.54	18.83	0.0013	0.0420
24 hours	twi_ss.30562.1|g.41410	-0.94	6.60	18.86	0.0013	0.0420
24 hours	twi_ss.26090b.4|g.34552	5.43	2.72	18.85	0.0013	0.0420
24 hours	twi_ss.30890.1|g.41957	0.92	7.10	18.86	0.0013	0.0420
24 hours	twi_ss.23138.1|g.29797	-1.51	6.55	18.84	0.0013	0.0420
24 hours	twi_ss.12946.2|g.17136	-4.18	3.75	18.75	0.0014	0.0425
24 hours	twi_ss.7929.1|g.10983	0.71	6.20	18.76	0.0014	0.0425
24 hours	twi_ss.7795.1|g.10876	1.47	4.89	18.75	0.0014	0.0425
24 hours	twi_ss.24393.1|g.31956	1.05	5.67	18.75	0.0014	0.0425
24 hours	twi_ss.3520.1|g.5891	1.60	5.16	18.70	0.0014	0.0429
24 hours	twi_ss.5182.1|g.7812	3.85	4.72	18.69	0.0014	0.0429
24 hours	twi_ss.27364.1|g.36414	0.61	9.50	18.65	0.0014	0.0431
24 hours	twi_ss.6137.1|g.9055	0.87	5.52	18.64	0.0014	0.0432
24 hours	twi_ss.7122a.1|g.10138	0.83	7.65	18.63	0.0014	0.0433
24 hours	twi_ss.11112.1|g.14736	-2.89	4.76	18.60	0.0014	0.0434
24 hours	twi_ss.718.2|g.1251	-4.01	7.70	21.84	0.0014	0.0434
24 hours	twi_ss.14256.1|g.18564	1.70	4.28	18.60	0.0014	0.0434
24 hours	twi_ss.31062.2|g.42240	-6.46	2.86	18.58	0.0014	0.0435
24 hours	twi_ss.17908.1|g.22797	2.64	4.70	18.56	0.0014	0.0435
24 hours	twi_ss.16547.1|g.21223	-1.03	6.36	18.56	0.0014	0.0435
24 hours	twi_ss.25303.1|g.33274	-6.63	2.98	21.80	0.0014	0.0435
24 hours	twi_ss.10747b.1|g.14386	-0.70	8.96	18.55	0.0014	0.0435
24 hours	twi_ss.17654.1|g.22531	-1.30	6.59	18.55	0.0014	0.0435

24 hours	twi_ss.9389.1|g.12651	1.35	4.58	18.52	0.0014	0.0436
24 hours	twi_ss.29469.1|g.39702	-1.81	5.46	18.52	0.0014	0.0436
24 hours	twi_ss.29937.1|g.40386	-3.29	4.23	18.51	0.0014	0.0436
24 hours	twi_ss.27159a.4|g.36070	-1.40	5.89	18.50	0.0014	0.0437
24 hours	twi_ss.21054.4|g.27165	5.55	2.56	21.68	0.0014	0.0438
24 hours	twi_ss.9071a.2|g.12243	0.95	5.73	18.48	0.0014	0.0438
24 hours	twi_ss.6556.1|g.9581	-1.52	4.44	18.47	0.0014	0.0438
24 hours	twi_ss.10837.5|g.14461	-3.32	5.41	18.47	0.0014	0.0438
24 hours	twi_ss.23331.1|g.30011	-7.00	6.22	18.44	0.0014	0.0440
24 hours	twi_ss.24735.8|g.32337	2.43	6.08	18.43	0.0014	0.0441
24 hours	twi_ss.22246.1|g.28786	-1.93	4.10	18.38	0.0015	0.0443
24 hours	twi_ss.7196.1|g.10248	0.65	7.15	18.39	0.0015	0.0443
24 hours	twi_ss.31635.1|g.43104	-2.61	4.12	18.35	0.0015	0.0445
24 hours	twi_ss.28745.6|g.38458	5.21	2.21	18.35	0.0015	0.0445
24 hours	twi_ss.3945.1|g.6368	1.39	5.19	18.35	0.0015	0.0445
24 hours	twi_ss.10657.1|g.14300	1.10	6.46	18.34	0.0015	0.0445
24 hours	twi_ss.12866.1|g.17033	2.32	4.46	18.33	0.0015	0.0446
24 hours	twi_ss.30390.1|g.41135	1.05	6.97	18.30	0.0015	0.0447
24 hours	twi_ss.15156.3|g.19688	-6.61	3.46	18.27	0.0015	0.0449
24 hours	twi_ss.23425b.1|g.30058	0.86	7.15	18.27	0.0015	0.0449
24 hours	twi_ss.12396.1|g.16520	-3.86	2.68	18.24	0.0015	0.0451
24 hours	twi_ss.22433.3|g.28978	-6.34	4.05	18.24	0.0015	0.0451
24 hours	twi_ss.1490.1|g.2655	3.14	4.63	18.22	0.0015	0.0452
24 hours	twi_ss.22209.1|g.28765	-2.22	4.30	18.21	0.0015	0.0453
24 hours	twi_ss.29511.1|g.39820	-6.25	3.30	18.21	0.0015	0.0453
24 hours	twi_ss.4126.1|g.6550	-2.79	2.97	18.19	0.0015	0.0453
24 hours	twi_ss.31853b.2|g.43360	-1.29	7.38	18.20	0.0015	0.0453
24 hours	twi_ss.25842.1|g.34070	7.30	3.42	21.30	0.0015	0.0453
24 hours	twi_ss.12990.2|g.17197	5.94	3.59	18.16	0.0015	0.0454
24 hours	twi_ss.12990.3|g.17194	5.94	3.59	18.16	0.0015	0.0454
24 hours	twi_ss.14964a.2|g.19368	1.76	5.63	18.15	0.0015	0.0454
24 hours	twi_ss.29721.1|g.40080	2.22	4.52	18.12	0.0015	0.0457
24 hours	twi_ss.9681.3|g.12959	5.06	2.23	18.11	0.0015	0.0457

24 hours	twi_ss.18990a.1|g.24426	-0.60	7.72	18.10	0.0015	0.0458
24 hours	twi_ss.23535.5|g.30208	5.33	2.51	18.08	0.0015	0.0459
24 hours	twi_ss.18817.1|g.24072	1.53	5.48	18.05	0.0016	0.0462
24 hours	twi_ss.10511.1|g.14144	0.45	7.98	18.04	0.0016	0.0462
24 hours	twi_ss.23805.1|g.30764	3.89	3.94	18.02	0.0016	0.0462
24 hours	twi_ss.3849.1|g.6270	-5.80	4.97	18.02	0.0016	0.0462
24 hours	twi_ss.25764.1|g.33997	0.89	6.32	17.96	0.0016	0.0466
24 hours	twi_ss.25822.1|g.34042	4.97	2.07	17.96	0.0016	0.0466
24 hours	twi_ss.25822.3|g.34043	4.97	2.07	17.96	0.0016	0.0466
24 hours	twi_ss.30222a.5|g.40814	-5.50	2.37	17.95	0.0016	0.0466
24 hours	twi_ss.14396.2|g.18739	0.47	8.46	17.95	0.0016	0.0466
24 hours	twi_ss.14541.1|g.19000	5.53	2.34	19.14	0.0016	0.0474
24 hours	twi_ss.182.1|g.283	-7.04	3.45	20.89	0.0016	0.0474
24 hours	twi_ss.23081.1|g.29724	-0.61	6.57	17.86	0.0016	0.0474
24 hours	twi_ss.26634.1|g.35287	-1.23	5.73	17.84	0.0016	0.0474
24 hours	twi_ss.7156.2|g.10228	4.99	2.15	17.84	0.0016	0.0474
24 hours	twi_ss.12609.2|g.16717	-5.74	2.72	17.83	0.0016	0.0475
24 hours	twi_ss.31306.1|g.42632	0.95	7.27	17.81	0.0016	0.0476
24 hours	twi_ss.1962.1|g.3437	-2.86	4.38	17.80	0.0016	0.0476
24 hours	twi_ss.10988.1|g.14628	1.06	4.86	17.78	0.0016	0.0477
24 hours	twi_ss.29566.1|g.39761	-1.12	7.88	17.77	0.0016	0.0478
24 hours	twi_ss.627.1|g.1124	-5.02	2.50	17.74	0.0016	0.0480
24 hours	twi_ss.1832.2|g.3219	0.87	6.02	17.72	0.0016	0.0482
24 hours	twi_ss.20781.1|g.26879	0.43	7.26	17.72	0.0017	0.0482
24 hours	twi_ss.30997.9|g.42043	5.95	2.63	20.67	0.0017	0.0483
24 hours	twi_ss.28372.1|g.37964	3.79	5.38	17.69	0.0017	0.0483
24 hours	twi_ss.22914.1|g.29581	1.40	4.98	17.67	0.0017	0.0485
24 hours	twi_ss.13995.3|g.18296	-5.82	3.05	20.59	0.0017	0.0488
24 hours	twi_ss.17491.1|g.22319	1.89	4.58	17.61	0.0017	0.0489
24 hours	twi_ss.26806.2|g.35580	-4.97	2.15	17.58	0.0017	0.0492
24 hours	twi_ss.14228.1|g.18486	-1.13	5.45	17.57	0.0017	0.0492
24 hours	twi_ss.18252.1|g.23230	-1.06	5.57	17.55	0.0017	0.0494
24 hours	twi_ss.2409a.1|g.4153	0.57	8.17	17.54	0.0017	0.0494

24 hours	twi_ss.9899.2|g.13226	-7.04	3.40	17.52	0.0017	0.0495
24 hours	twi_ss.11371.1|g.15014	-3.03	4.00	17.51	0.0017	0.0496
24 hours	twi_ss.29926.1|g.40436	-4.20	2.63	23.74	0.0017	0.0496
24 hours	twi_ss.19449.1|g.24980	4.24	4.31	17.47	0.0017	0.0497
24 hours	twi_ss.20491.1|g.26448	2.29	4.40	17.49	0.0017	0.0497
24 hours	twi_ss.28270a.6|g.37797	-4.50	1.99	26.54	0.0017	0.0497
24 hours	twi_ss.31964.1|g.43602	-2.19	4.21	17.48	0.0017	0.0497
24 hours	twi_ss.21197a.2|g.27421	2.10	4.81	17.47	0.0017	0.0497
24 hours	twi_ss.6079.1|g.8932	0.89	7.81	17.47	0.0017	0.0497
24 hours	twi_ss.25085a.1|g.32915	-1.18	5.04	17.45	0.0017	0.0498
24 hours	twi_ss.31934.1|g.43538	-0.82	8.79	17.44	0.0017	0.0499
24 hours	twi_ss.26015c.3|g.34432	1.46	4.92	17.42	0.0017	0.0500
24 hours	twi_ss.26417.1|g.35010	1.58	5.03	17.41	0.0018	0.0501
24 hours	twi_ss.22735.2|g.29344	-5.49	2.34	17.40	0.0018	0.0501
24 hours	twi_ss.2655.1|g.4837	1.24	5.39	17.39	0.0018	0.0502
24 hours	twi_ss.28666.1|g.38373	-2.07	5.56	17.38	0.0018	0.0502
24 hours	twi_ss.30867.2|g.41899	0.83	5.78	17.37	0.0018	0.0502
7 days	twi_ss.1219.1|g.2104	-4.71	8.02	591.30	1.66E-10	0.0000
7 days	twi_ss.4874.1|g.7384	-5.06	7.60	664.01	9.14E-11	0.0000
7 days	twi_ss.800.2|g.1407	-4.26	6.66	570.14	2.00E-10	0.0000
7 days	twi_ss.20755a.2|g.26865	-3.32	8.81	522.58	3.11E-10	0.0000
7 days	twi_ss.3712.1|g.6096	6.57	10.33	489.65	4.34E-10	0.0000
7 days	twi_ss.5201.1|g.7826	-4.29	8.11	409.97	1.07E-09	0.0000
7 days	twi_ss.1226.1|g.2106	-4.24	7.40	362.73	2.00E-09	0.0000
7 days	twi_ss.13838.1|g.18145	3.60	9.41	333.40	3.06E-09	0.0000
7 days	twi_ss.14278.1|g.18615	6.65	9.20	328.52	3.30E-09	0.0000
7 days	twi_ss.19635.1|g.25202	2.05	8.15	324.94	3.49E-09	0.0000
7 days	twi_ss.20735.1|g.26813	9.33	9.95	330.11	3.22E-09	0.0000
7 days	twi_ss.24166.1|g.31414	3.14	9.99	318.44	3.86E-09	0.0000
7 days	twi_ss.25299a.1|g.33316	-4.57	7.03	335.46	2.97E-09	0.0000
7 days	twi_ss.25693a.1|g.33896	5.53	11.28	342.40	2.68E-09	0.0000
7 days	twi_ss.28038b.1|g.37328	2.59	8.70	319.89	3.77E-09	0.0000
7 days	twi_ss.4034.1|g.6451	-4.32	6.78	284.36	6.83E-09	0.0000

7 days	twi_ss.12823b.1|g.16930	-8.35	4.65	275.35	8.03E-09	0.0000
7 days	twi_ss.5643.1|g.8453	5.30	10.41	274.74	8.12E-09	0.0000
7 days	twi_ss.13698.1|g.18022	2.38	7.15	268.62	9.10E-09	0.0000
7 days	twi_ss.11722.2|g.15540	6.10	9.40	259.14	1.09E-08	0.0000
7 days	twi_ss.25299b.2|g.33311	5.17	9.98	257.69	1.12E-08	0.0000
7 days	twi_ss.25693b.2|g.33886	4.37	9.40	256.42	1.15E-08	0.0000
7 days	twi_ss.20282.1|g.26105	2.40	7.34	239.69	1.61E-08	0.0000
7 days	twi_ss.31790b.1|g.43276	-3.66	7.71	237.21	1.70E-08	0.0000
7 days	twi_ss.800.1|g.1410	-3.91	7.58	236.23	1.73E-08	0.0000
7 days	twi_ss.9907.1|g.13240	3.99	7.26	235.70	1.75E-08	0.0000
7 days	twi_ss.20795.1|g.26925	11.19	10.91	228.85	2.03E-08	0.0000
7 days	twi_ss.647.1|g.1197	1.64	7.65	226.19	2.15E-08	0.0000
7 days	twi_ss.18478.1|g.23540	-5.72	7.38	217.92	2.59E-08	0.0000
7 days	twi_ss.120.2|g.195	2.28	6.95	214.63	2.80E-08	0.0000
7 days	twi_ss.19960a.4|g.25706	7.30	4.70	211.37	3.02E-08	0.0000
7 days	twi_ss.4463.1|g.6986	3.46	10.14	212.57	2.93E-08	0.0000
7 days	twi_ss.15729.1|g.20309	3.09	9.44	207.86	3.28E-08	0.0000
7 days	twi_ss.10965.1|g.14538	3.67	12.07	199.06	4.07E-08	0.0000
7 days	twi_ss.11312.1|g.14924	2.91	7.86	195.27	4.47E-08	0.0000
7 days	twi_ss.14730.1|g.19254	2.21	6.87	192.56	4.79E-08	0.0000
7 days	twi_ss.4874.2|g.7378	-1.74	8.80	189.50	5.19E-08	0.0000
7 days	twi_ss.12661.1|g.16777	-6.42	5.53	180.94	6.52E-08	0.0000
7 days	twi_ss.21060a.1|g.27171	1.83	7.92	183.50	6.09E-08	0.0000
7 days	twi_ss.25751.1|g.33988	-1.97	7.05	180.95	6.52E-08	0.0000
7 days	twi_ss.26358.1|g.34798	9.12	9.69	182.45	6.26E-08	0.0000
7 days	twi_ss.27063.1|g.35999	9.58	5.95	308.40	6.42E-08	0.0000
7 days	twi_ss.28048.1|g.37371	5.96	7.95	182.13	6.32E-08	0.0000
7 days	twi_ss.7586.1|g.10632	2.05	8.25	181.88	6.36E-08	0.0000
7 days	twi_ss.2376.1|g.4266	2.73	6.29	174.00	7.91E-08	0.0001
7 days	twi_ss.21316b.3|g.27576	2.93	7.41	171.48	8.50E-08	0.0001
7 days	twi_ss.6620.1|g.9636	2.80	9.55	171.30	8.55E-08	0.0001
7 days	twi_ss.21114b.1|g.27305	-3.53	6.39	169.73	8.94E-08	0.0001
7 days	twi_ss.19960a.2|g.25708	-6.90	4.09	166.22	9.91E-08	0.0001

7 days	twi_ss.978.1|g.1784	6.26	10.48	165.01	1.03E-07	0.0001
7 days	twi_ss.2232.1|g.3943	2.83	6.78	162.15	1.12E-07	0.0001
7 days	twi_ss.26446.1|g.35014	2.22	9.82	159.67	1.21E-07	0.0001
7 days	twi_ss.3382.1|g.5769	3.05	7.96	159.24	1.22E-07	0.0001
7 days	twi_ss.20513.1|g.26432	-3.98	4.89	153.83	1.45E-07	0.0001
7 days	twi_ss.2869.2|g.5156	3.69	7.03	153.96	1.44E-07	0.0001
7 days	twi_ss.1765.1|g.3114	2.45	9.24	153.06	1.49E-07	0.0001
7 days	twi_ss.6452.1|g.9489	4.12	9.20	151.55	1.56E-07	0.0001
7 days	twi_ss.8361b.2|g.11480	2.77	7.66	149.77	1.65E-07	0.0001
7 days	twi_ss.7858.1|g.10912	1.92	8.17	148.72	1.71E-07	0.0001
7 days	twi_ss.9362.1|g.12634	-4.70	8.80	148.92	1.70E-07	0.0001
7 days	twi_ss.27768.1|g.37001	5.43	6.17	147.95	1.75E-07	0.0001
7 days	twi_ss.24106.1|g.31280	4.65	6.28	146.23	1.86E-07	0.0001
7 days	twi_ss.28647.1|g.38357	1.74	8.32	144.65	1.96E-07	0.0001
7 days	twi_ss.4710.1|g.7263	-4.26	5.16	136.41	2.61E-07	0.0001
7 days	twi_ss.6045.1|g.8903	2.67	7.02	136.42	2.61E-07	0.0001
7 days	twi_ss.24532a.1|g.32203	-1.82	7.04	135.74	2.67E-07	0.0001
7 days	twi_ss.329.1|g.608	2.15	8.45	134.33	2.81E-07	0.0001
7 days	twi_ss.12226.1|g.16336	5.30	6.55	133.02	2.95E-07	0.0001
7 days	twi_ss.2611.1|g.4635	3.36	8.33	132.66	2.98E-07	0.0001
7 days	twi_ss.2869.1|g.5155	6.02	5.94	132.30	3.02E-07	0.0001
7 days	twi_ss.22567.1|g.29150	-2.49	6.27	130.47	3.24E-07	0.0001
7 days	twi_ss.17561.1|g.22399	-1.48	9.15	129.91	3.30E-07	0.0001
7 days	twi_ss.23402.1|g.30075	2.68	6.81	129.27	3.38E-07	0.0001
7 days	twi_ss.16738.1|g.21427	2.39	6.91	128.39	3.50E-07	0.0001
7 days	twi_ss.19570.5|g.25098	8.29	4.87	128.60	3.47E-07	0.0001
7 days	twi_ss.6037.1|g.8896	2.97	5.97	127.74	3.58E-07	0.0001
7 days	twi_ss.25105.9|g.32925	-7.18	3.41	126.83	3.71E-07	0.0001
7 days	twi_ss.5820.1|g.8724	-2.78	7.97	126.75	3.72E-07	0.0001
7 days	twi_ss.13405.1|g.17801	-5.17	8.97	126.41	3.77E-07	0.0001
7 days	twi_ss.10440.1|g.14080	1.27	8.43	124.88	4.00E-07	0.0001
7 days	twi_ss.21105.9|g.27276	10.58	6.72	435.59	3.94E-07	0.0001
7 days	twi_ss.2619.3|g.4738	2.43	7.74	124.88	4.00E-07	0.0001

7 days	twi_ss.6884.1|g.9949	3.99	6.45	124.65	4.04E-07	0.0001
7 days	twi_ss.21635b.1|g.28037	1.32	9.47	121.62	4.55E-07	0.0002
7 days	twi_ss.6750.1|g.9832	3.00	8.24	120.69	4.72E-07	0.0002
7 days	twi_ss.1469.1|g.2686	-3.00	4.99	119.90	4.87E-07	0.0002
7 days	twi_ss.556.2|g.1009	-4.87	6.22	119.25	5.00E-07	0.0002
7 days	twi_ss.10882.1|g.14512	6.00	9.11	118.17	5.22E-07	0.0002
7 days	twi_ss.20450.1|g.26352	1.66	7.40	118.12	5.23E-07	0.0002
7 days	twi_ss.31673.1|g.43122	2.77	8.01	114.17	6.16E-07	0.0002
7 days	twi_ss.3533.1|g.5908	6.30	4.04	114.24	6.14E-07	0.0002
7 days	twi_ss.3533.2|g.5904	6.30	4.04	114.24	6.14E-07	0.0002
7 days	twi_ss.3533.3|g.5912	6.30	4.04	114.24	6.14E-07	0.0002
7 days	twi_ss.3533.4|g.5906	6.30	4.04	114.24	6.14E-07	0.0002
7 days	twi_ss.1152.1|g.2029	1.44	7.01	111.98	6.76E-07	0.0002
7 days	twi_ss.2375a.2|g.4144	1.70	6.79	111.89	6.79E-07	0.0002
7 days	twi_ss.5155.1|g.7788	1.86	6.27	111.82	6.81E-07	0.0002
7 days	twi_ss.16547.1|g.21223	-2.77	6.36	109.92	7.39E-07	0.0002
7 days	twi_ss.27219.1|g.36158	2.54	10.24	110.80	7.12E-07	0.0002
7 days	twi_ss.28657.1|g.38361	1.79	7.33	110.34	7.26E-07	0.0002
7 days	twi_ss.29169b.4|g.39088	1.84	9.29	109.95	7.38E-07	0.0002
7 days	twi_ss.6091.1|g.8963	1.89	7.70	109.93	7.39E-07	0.0002
7 days	twi_ss.3782.2|g.6191	1.54	7.62	109.51	7.52E-07	0.0002
7 days	twi_ss.22556.1|g.29135	-5.80	7.02	165.06	8.15E-07	0.0002
7 days	twi_ss.3513.1|g.5884	-1.68	7.03	106.11	8.75E-07	0.0003
7 days	twi_ss.7206.1|g.10257	2.43	6.36	106.32	8.67E-07	0.0003
7 days	twi_ss.22497.1|g.29072	2.15	9.61	105.24	9.10E-07	0.0003
7 days	twi_ss.29381.1|g.39493	1.29	6.98	105.13	9.15E-07	0.0003
7 days	twi_ss.6040.1|g.8889	2.10	9.34	105.36	9.05E-07	0.0003
7 days	twi_ss.10495.1|g.14129	2.10	9.39	104.21	9.54E-07	0.0003
7 days	twi_ss.19895.1|g.25639	1.78	8.07	104.25	9.52E-07	0.0003
7 days	twi_ss.30027b.2|g.40527	4.20	8.55	104.52	9.40E-07	0.0003
7 days	twi_ss.19329a.2|g.24785	2.24	8.63	103.60	9.81E-07	0.0003
7 days	twi_ss.2053.1|g.3716	-4.81	4.96	103.51	9.85E-07	0.0003
7 days	twi_ss.12047.1|g.15946	2.26	6.60	102.80	1.02E-06	0.0003

7 days	twi_ss.26616.1|g.35296	-3.82	7.34	102.12	1.05E-06	0.0003
7 days	twi_ss.29475.1|g.39689	3.80	7.92	101.93	1.06E-06	0.0003
7 days	twi_ss.13436.1|g.17773	2.55	7.23	100.55	1.13E-06	0.0003
7 days	twi_ss.16612.1|g.21263	-2.44	6.64	100.36	1.14E-06	0.0003
7 days	twi_ss.21059.1|g.27172	1.80	7.39	99.84	1.17E-06	0.0003
7 days	twi_ss.29759b.1|g.40113	3.62	6.10	99.77	1.17E-06	0.0003
7 days	twi_ss.11060.1|g.14695	1.75	7.03	98.70	1.23E-06	0.0003
7 days	twi_ss.178.1|g.287	1.65	6.66	98.65	1.24E-06	0.0003
7 days	twi_ss.6204.1|g.9043	2.49	8.39	98.68	1.24E-06	0.0003
7 days	twi_ss.7208.2|g.10260	4.19	5.93	98.69	1.24E-06	0.0003
7 days	twi_ss.4014.3|g.6432	-2.31	6.61	97.46	1.31E-06	0.0003
7 days	twi_ss.3793.1|g.6196	-4.01	4.89	97.10	1.33E-06	0.0003
7 days	twi_ss.25566.1|g.33668	-4.69	4.98	95.37	1.45E-06	0.0003
7 days	twi_ss.19875.1|g.25622	1.52	8.29	94.67	1.50E-06	0.0003
7 days	twi_ss.28616.1|g.38309	2.61	6.73	94.98	1.48E-06	0.0003
7 days	twi_ss.30724.1|g.41642	3.74	7.69	94.71	1.50E-06	0.0003
7 days	twi_ss.4286.1|g.6756	1.66	10.01	94.94	1.48E-06	0.0003
7 days	twi_ss.28736.1|g.38496	-1.52	6.41	94.52	1.52E-06	0.0003
7 days	twi_ss.17802.1|g.22700	8.06	10.31	93.97	1.56E-06	0.0004
7 days	twi_ss.26111.1|g.34564	3.59	6.64	94.12	1.55E-06	0.0004
7 days	twi_ss.2019.2|g.3587	1.18	9.70	93.42	1.60E-06	0.0004
7 days	twi_ss.21611.1|g.28009	1.63	7.71	93.40	1.60E-06	0.0004
7 days	twi_ss.30108.1|g.40611	2.22	9.08	93.25	1.62E-06	0.0004
7 days	twi_ss.31789.1|g.43293	-4.03	7.55	93.04	1.63E-06	0.0004
7 days	twi_ss.9089.1|g.12267	-5.70	7.43	92.78	1.65E-06	0.0004
7 days	twi_ss.28936.1|g.38782	-2.39	8.53	92.43	1.68E-06	0.0004
7 days	twi_ss.8873.1|g.12071	3.03	9.97	92.44	1.68E-06	0.0004
7 days	twi_ss.18522.1|g.23566	-6.22	7.52	137.18	1.71E-06	0.0004
7 days	twi_ss.14325.1|g.18631	1.99	7.48	91.21	1.79E-06	0.0004
7 days	twi_ss.24809.1|g.32606	-3.04	8.19	91.56	1.76E-06	0.0004
7 days	twi_ss.26117a.1|g.34555	2.35	5.20	91.37	1.78E-06	0.0004
7 days	twi_ss.28424.1|g.38003	3.23	6.85	91.30	1.79E-06	0.0004
7 days	twi_ss.26622.1|g.35284	1.96	7.49	90.84	1.83E-06	0.0004

7 days	twi_ss.13619b.2|g.17975	1.48	6.81	90.06	1.90E-06	0.0004
7 days	twi_ss.3335.2|g.5740	-2.04	7.29	90.02	1.91E-06	0.0004
7 days	twi_ss.26297.1|g.34808	-7.74	5.79	89.61	1.95E-06	0.0004
7 days	twi_ss.9647.1|g.12933	1.45	7.92	89.70	1.94E-06	0.0004
7 days	twi_ss.2517.1|g.4486	-2.11	6.72	89.23	1.99E-06	0.0004
7 days	twi_ss.2518.1|g.4528	3.44	5.44	88.99	2.01E-06	0.0004
7 days	twi_ss.13566.1|g.17913	4.04	8.08	88.22	2.10E-06	0.0004
7 days	twi_ss.21610.6|g.28011	3.53	6.06	87.96	2.13E-06	0.0004
7 days	twi_ss.2312b.3|g.4065	-6.44	3.27	88.01	2.12E-06	0.0004
7 days	twi_ss.11664.1|g.15469	3.16	6.76	87.32	2.20E-06	0.0004
7 days	twi_ss.12243.1|g.16329	2.40	6.81	86.88	2.25E-06	0.0004
7 days	twi_ss.12889.1|g.17077	-4.57	4.65	86.77	2.27E-06	0.0004
7 days	twi_ss.21105.1|g.27279	6.15	9.10	86.66	2.28E-06	0.0004
7 days	twi_ss.22049a.2|g.28532	-1.83	8.29	86.71	2.28E-06	0.0004
7 days	twi_ss.23514.1|g.30170	4.41	6.85	86.88	2.25E-06	0.0004
7 days	twi_ss.23722.1|g.30715	2.33	7.62	86.78	2.27E-06	0.0004
7 days	twi_ss.5069.1|g.7717	-3.16	7.16	86.81	2.26E-06	0.0004
7 days	twi_ss.24296.1|g.31742	1.53	8.01	86.21	2.34E-06	0.0004
7 days	twi_ss.9661.1|g.12991	1.76	6.09	85.64	2.41E-06	0.0004
7 days	twi_ss.10837.3|g.14465	2.57	6.52	85.42	2.44E-06	0.0004
7 days	twi_ss.17485.1|g.22307	2.02	6.02	85.15	2.48E-06	0.0004
7 days	twi_ss.1871.1|g.3293	3.01	8.47	84.89	2.51E-06	0.0004
7 days	twi_ss.28414.1|g.38016	3.01	6.71	84.55	2.56E-06	0.0005
7 days	twi_ss.23644.1|g.30455	1.51	7.35	84.43	2.58E-06	0.0005
7 days	twi_ss.14780.1|g.19240	2.58	7.81	84.24	2.60E-06	0.0005
7 days	twi_ss.8200.1|g.11287	3.08	5.60	84.08	2.63E-06	0.0005
7 days	twi_ss.6877.1|g.9936	3.71	5.24	83.95	2.65E-06	0.0005
7 days	twi_ss.10945.1|g.14555	5.34	6.62	83.39	2.73E-06	0.0005
7 days	twi_ss.11864.1|g.15756	2.02	6.14	83.54	2.71E-06	0.0005
7 days	twi_ss.23257.1|g.29910	-3.31	4.82	83.43	2.73E-06	0.0005
7 days	twi_ss.4929.1|g.7529	2.05	6.55	82.32	2.90E-06	0.0005
7 days	twi_ss.5295.1|g.8034	-4.40	6.25	82.10	2.94E-06	0.0005
7 days	twi_ss.22219.1|g.28723	2.30	6.13	81.69	3.01E-06	0.0005

7 days	twi_ss.14612.3|g.19088	2.95	6.41	81.44	3.05E-06	0.0005
7 days	twi_ss.17801.1|g.22698	3.52	8.98	81.25	3.08E-06	0.0005
7 days	twi_ss.30312.8|g.40958	1.75	6.50	81.21	3.09E-06	0.0005
7 days	twi_ss.459c.1|g.826	-1.40	8.81	80.51	3.22E-06	0.0005
7 days	twi_ss.30096b.2|g.40711	2.20	9.17	80.10	3.29E-06	0.0005
7 days	twi_ss.431.1|g.791	-2.56	5.28	80.18	3.28E-06	0.0005
7 days	twi_ss.10752.1|g.14417	3.97	6.96	79.98	3.32E-06	0.0005
7 days	twi_ss.31777.1|g.43288	5.05	6.49	79.90	3.33E-06	0.0005
7 days	twi_ss.21214.1|g.27436	2.05	7.28	79.47	3.42E-06	0.0005
7 days	twi_ss.3152.1|g.5557	2.74	7.62	79.54	3.40E-06	0.0005
7 days	twi_ss.9862.1|g.13198	1.13	7.60	79.08	3.50E-06	0.0006
7 days	twi_ss.1191.1|g.2086	1.39	6.81	78.76	3.56E-06	0.0006
7 days	twi_ss.20391.1|g.26269	-5.54	6.60	113.55	3.61E-06	0.0006
7 days	twi_ss.21205.1|g.27437	3.01	6.27	78.64	3.59E-06	0.0006
7 days	twi_ss.8570.1|g.11715	-1.59	7.71	78.60	3.60E-06	0.0006
7 days	twi_ss.824.2|g.1435	-2.81	5.36	78.25	3.67E-06	0.0006
7 days	twi_ss.3922a.1|g.6353	1.56	6.88	78.06	3.71E-06	0.0006
7 days	twi_ss.21105.2|g.27285	6.98	7.96	77.47	3.85E-06	0.0006
7 days	twi_ss.9313.4|g.12557	-4.52	8.39	111.42	3.89E-06	0.0006
7 days	twi_ss.1168.1|g.2027	2.65	6.63	77.02	3.95E-06	0.0006
7 days	twi_ss.22264.1|g.28821	-6.30	5.59	76.73	4.02E-06	0.0006
7 days	twi_ss.2690.3|g.4893	2.34	7.26	76.85	3.99E-06	0.0006
7 days	twi_ss.28922.1|g.38780	-6.54	5.38	110.45	4.03E-06	0.0006
7 days	twi_ss.28991.1|g.38906	-7.19	3.31	76.31	4.13E-06	0.0006
7 days	twi_ss.8351.1|g.11463	1.48	6.89	76.20	4.15E-06	0.0006
7 days	twi_ss.8912.1|g.12098	-1.89	6.22	76.22	4.15E-06	0.0006
7 days	twi_ss.16184.1|g.20768	-3.57	5.70	109.38	4.18E-06	0.0006
7 days	twi_ss.16353.1|g.20942	-7.00	6.87	108.75	4.28E-06	0.0006
7 days	twi_ss.27979b.1|g.37212	1.06	9.27	75.62	4.30E-06	0.0006
7 days	twi_ss.18881a.22|g.23994	-2.34	6.71	75.37	4.37E-06	0.0006
7 days	twi_ss.26069.1|g.34483	-2.80	5.50	75.28	4.39E-06	0.0006
7 days	twi_ss.21437.1|g.27736	1.45	8.44	74.78	4.53E-06	0.0006
7 days	twi_ss.24236.1|g.31566	-9.10	11.20	74.89	4.50E-06	0.0006

7 days	twi_ss.28156a.2|g.37510	-9.73	9.33	74.78	4.53E-06	0.0006
7 days	twi_ss.7769.1|g.10845	-2.52	6.58	74.91	4.50E-06	0.0006
7 days	twi_ss.28938.1|g.38803	-5.15	4.93	74.62	4.57E-06	0.0006
7 days	twi_ss.7711.1|g.10773	2.76	5.80	74.55	4.59E-06	0.0006
7 days	twi_ss.21950.1|g.28445	1.14	7.32	74.46	4.62E-06	0.0006
7 days	twi_ss.247.1|g.483	1.72	7.87	74.40	4.64E-06	0.0006
7 days	twi_ss.13863.1|g.18169	1.53	8.89	73.75	4.83E-06	0.0007
7 days	twi_ss.18952b.3|g.24232	-2.49	5.38	73.74	4.83E-06	0.0007
7 days	twi_ss.24294.1|g.31738	1.40	7.66	73.82	4.81E-06	0.0007
7 days	twi_ss.852.1|g.1428	-1.34	9.60	73.71	4.84E-06	0.0007
7 days	twi_ss.11366.1|g.15001	2.05	9.43	73.57	4.88E-06	0.0007
7 days	twi_ss.16258.1|g.20833	-6.29	5.05	73.35	4.95E-06	0.0007
7 days	twi_ss.15379.1|g.19919	-2.83	5.86	72.62	5.19E-06	0.0007
7 days	twi_ss.1579a.1|g.2780	3.00	6.85	72.67	5.17E-06	0.0007
7 days	twi_ss.27046.1|g.35942	1.22	6.75	72.70	5.16E-06	0.0007
7 days	twi_ss.7626.1|g.10685	2.02	7.31	72.17	5.34E-06	0.0007
7 days	twi_ss.7998.1|g.11044	-3.19	7.51	71.64	5.52E-06	0.0007
7 days	twi_ss.7142.1|g.10189	0.99	7.28	71.51	5.57E-06	0.0007
7 days	twi_ss.25312.3|g.33264	5.53	7.88	71.38	5.61E-06	0.0007
7 days	twi_ss.623.1|g.1129	-3.55	6.36	71.30	5.64E-06	0.0007
7 days	twi_ss.15332.1|g.19854	-1.04	9.12	70.80	5.83E-06	0.0008
7 days	twi_ss.12222.1|g.16260	2.00	7.14	70.57	5.92E-06	0.0008
7 days	twi_ss.14354.1|g.18661	3.17	8.80	70.42	5.97E-06	0.0008
7 days	twi_ss.10122.1|g.13573	-11.17	11.62	70.09	6.10E-06	0.0008
7 days	twi_ss.1994.1|g.3586	2.72	5.57	69.92	6.17E-06	0.0008
7 days	twi_ss.30512b.1|g.41315	-3.26	5.12	69.94	6.16E-06	0.0008
7 days	twi_ss.19882.1|g.25643	2.25	6.84	69.68	6.27E-06	0.0008
7 days	twi_ss.25457.1|g.33489	2.12	8.38	69.74	6.25E-06	0.0008
7 days	twi_ss.4017.1|g.6439	-3.55	5.71	69.11	6.51E-06	0.0008
7 days	twi_ss.19594.1|g.25172	-1.73	6.66	68.94	6.58E-06	0.0008
7 days	twi_ss.22642.1|g.29229	8.22	8.38	68.86	6.62E-06	0.0008
7 days	twi_ss.12669.1|g.16768	-3.47	4.72	68.48	6.79E-06	0.0008
7 days	twi_ss.23845.1|g.30845	-9.76	7.37	68.54	6.76E-06	0.0008

7 days	twi_ss.24823.1|g.32552	3.77	5.26	68.44	6.80E-06	0.0008
7 days	twi_ss.17277a.5|g.22017	-7.27	4.13	95.99	6.98E-06	0.0009
7 days	twi_ss.16476.1|g.21059	-2.91	6.38	68.00	7.01E-06	0.0009
7 days	twi_ss.3533.5|g.5910	-2.62	5.35	67.83	7.09E-06	0.0009
7 days	twi_ss.12025.1|g.15916	2.35	7.28	67.45	7.27E-06	0.0009
7 days	twi_ss.18548.1|g.23653	-3.95	5.37	67.53	7.23E-06	0.0009
7 days	twi_ss.20961a.1|g.27085	2.13	6.27	67.48	7.26E-06	0.0009
7 days	twi_ss.31453.1|g.42823	-2.96	7.75	67.50	7.25E-06	0.0009
7 days	twi_ss.16737.1|g.21429	1.88	7.44	66.54	7.74E-06	0.0009
7 days	twi_ss.9920.1|g.13258	-6.08	2.70	66.21	7.91E-06	0.0009
7 days	twi_ss.8875.1|g.12104	2.38	6.38	65.99	8.04E-06	0.0009
7 days	twi_ss.30338.1|g.41031	-1.98	6.34	65.26	8.45E-06	0.0010
7 days	twi_ss.27582c.1|g.36775	1.69	7.64	65.09	8.55E-06	0.0010
7 days	twi_ss.21231.1|g.27456	1.41	6.70	64.73	8.77E-06	0.0010
7 days	twi_ss.24245.1|g.31625	-8.93	10.63	64.83	8.71E-06	0.0010
7 days	twi_ss.4881.1|g.7484	-2.30	8.71	64.76	8.76E-06	0.0010
7 days	twi_ss.7791.1|g.10881	1.41	7.26	64.67	8.81E-06	0.0010
7 days	twi_ss.24308.1|g.31752	-1.16	7.39	64.48	8.93E-06	0.0010
7 days	twi_ss.10271b.2|g.13825	1.80	8.06	64.14	9.14E-06	0.0010
7 days	twi_ss.1442.2|g.2639	3.60	8.14	64.16	9.13E-06	0.0010
7 days	twi_ss.21105.3|g.27275	6.05	6.10	63.96	9.26E-06	0.0011
7 days	twi_ss.30546.1|g.41379	5.04	7.52	63.90	9.30E-06	0.0011
7 days	twi_ss.28008.1|g.37358	1.16	7.20	63.76	9.39E-06	0.0011
7 days	twi_ss.28158a.3|g.37550	-6.44	2.83	63.77	9.39E-06	0.0011
7 days	twi_ss.5590a.7|g.8350	-8.89	5.11	158.20	9.41E-06	0.0011
7 days	twi_ss.14116b.2|g.18421	3.21	4.82	63.67	9.45E-06	0.0011
7 days	twi_ss.8806.1|g.11996	-3.70	7.04	63.61	9.49E-06	0.0011
7 days	twi_ss.4989.1|g.7605	1.78	6.67	63.04	9.89E-06	0.0011
7 days	twi_ss.16656.2|g.21332	10.43	7.44	62.70	1.01E-05	0.0011
7 days	twi_ss.7331.1|g.10349	1.66	6.55	62.56	1.02E-05	0.0011
7 days	twi_ss.11488c.5|g.15145	1.37	5.74	62.19	1.05E-05	0.0012
7 days	twi_ss.25312.2|g.33268	-4.47	4.57	62.12	1.06E-05	0.0012
7 days	twi_ss.31729.1|g.43227	3.43	4.47	62.04	1.06E-05	0.0012

7 days	twi_ss.23740.1|g.30719	1.18	10.16	61.94	1.07E-05	0.0012
7 days	twi_ss.6541.1|g.9566	1.01	9.15	61.95	1.07E-05	0.0012
7 days	twi_ss.1972.1|g.3478	1.37	10.23	61.62	1.10E-05	0.0012
7 days	twi_ss.21348.1|g.27573	-2.62	5.71	61.61	1.10E-05	0.0012
7 days	twi_ss.4178.1|g.6595	-3.83	5.24	61.53	1.10E-05	0.0012
7 days	twi_ss.10838.1|g.14453	2.13	5.42	61.04	1.14E-05	0.0012
7 days	twi_ss.25826.1|g.34060	2.32	4.87	61.01	1.15E-05	0.0012
7 days	twi_ss.27783a.2|g.36942	1.64	7.56	60.99	1.15E-05	0.0012
7 days	twi_ss.9775.1|g.13150	4.10	5.03	61.09	1.14E-05	0.0012
7 days	twi_ss.2422.1|g.4431	4.83	5.64	60.70	1.17E-05	0.0012
7 days	twi_ss.29444.1|g.39654	0.84	7.31	60.61	1.18E-05	0.0012
7 days	twi_ss.21105.6|g.27278	4.87	5.41	60.46	1.19E-05	0.0012
7 days	twi_ss.22010.1|g.28502	1.33	6.59	60.42	1.20E-05	0.0012
7 days	twi_ss.23516.1|g.30163	2.30	7.31	60.42	1.20E-05	0.0012
7 days	twi_ss.25703.1|g.33871	-3.63	6.17	60.34	1.21E-05	0.0012
7 days	twi_ss.13445.1|g.17741	1.60	6.40	59.76	1.26E-05	0.0013
7 days	twi_ss.20094.1|g.25861	-5.78	5.74	59.73	1.26E-05	0.0013
7 days	twi_ss.24697.1|g.32404	1.90	6.36	59.82	1.25E-05	0.0013
7 days	twi_ss.25233.1|g.33148	-2.03	6.15	59.85	1.25E-05	0.0013
7 days	twi_ss.31796.1|g.43314	-4.13	8.77	59.83	1.25E-05	0.0013
7 days	twi_ss.9756.1|g.13098	-3.66	4.88	59.93	1.24E-05	0.0013
7 days	twi_ss.17094.1|g.21879	-5.93	4.78	59.51	1.28E-05	0.0013
7 days	twi_ss.10271a.1|g.13759	1.41	7.13	59.30	1.30E-05	0.0013
7 days	twi_ss.20991.1|g.27100	-9.48	5.78	59.36	1.30E-05	0.0013
7 days	twi_ss.28666.1|g.38373	-4.69	5.56	59.43	1.29E-05	0.0013
7 days	twi_ss.8788.1|g.11924	1.43	6.58	59.33	1.30E-05	0.0013
7 days	twi_ss.12972.1|g.17144	3.15	4.99	59.15	1.32E-05	0.0013
7 days	twi_ss.23514.2|g.30168	4.71	5.15	59.17	1.32E-05	0.0013
7 days	twi_ss.11909.1|g.15789	3.08	5.69	58.95	1.34E-05	0.0013
7 days	twi_ss.20001.1|g.25769	-3.26	6.01	58.91	1.34E-05	0.0013
7 days	twi_ss.20963.1|g.27081	1.97	4.56	58.68	1.37E-05	0.0013
7 days	twi_ss.7122a.1|g.10138	1.47	7.65	58.61	1.37E-05	0.0013
7 days	twi_ss.469.1|g.851	2.11	5.67	58.52	1.38E-05	0.0013

7 days	twi_ss.12318.1|g.16442	-1.93	6.49	58.41	1.39E-05	0.0014
7 days	twi_ss.16285.1|g.20874	2.46	8.02	58.25	1.41E-05	0.0014
7 days	twi_ss.20998.1|g.27114	-2.95	4.34	58.14	1.42E-05	0.0014
7 days	twi_ss.4179.1|g.6597	3.42	6.34	58.14	1.42E-05	0.0014
7 days	twi_ss.6495.1|g.9528	1.55	6.55	57.94	1.45E-05	0.0014
7 days	twi_ss.26378.1|g.34868	2.40	6.94	57.87	1.45E-05	0.0014
7 days	twi_ss.6094.1|g.8967	-4.35	4.30	57.84	1.46E-05	0.0014
7 days	twi_ss.2464.1|g.4481	1.90	6.51	57.62	1.48E-05	0.0014
7 days	twi_ss.1968.1|g.3462	1.32	11.14	57.10	1.54E-05	0.0015
7 days	twi_ss.4103.1|g.6462	2.52	9.01	57.14	1.54E-05	0.0015
7 days	twi_ss.31795.1|g.43313	-4.11	4.62	57.03	1.55E-05	0.0015
7 days	twi_ss.28854.1|g.38686	-1.93	7.67	56.90	1.57E-05	0.0015
7 days	twi_ss.7080.1|g.10166	1.80	6.64	56.89	1.57E-05	0.0015
7 days	twi_ss.5279.3|g.7978	-7.16	3.31	77.39	1.60E-05	0.0015
7 days	twi_ss.12008.1|g.15934	1.49	6.44	56.53	1.61E-05	0.0015
7 days	twi_ss.26361b.4|g.34823	-9.57	7.24	56.35	1.64E-05	0.0015
7 days	twi_ss.27617.1|g.36652	1.88	6.02	56.33	1.64E-05	0.0015
7 days	twi_ss.1625.1|g.2826	1.31	7.16	56.27	1.65E-05	0.0015
7 days	twi_ss.7858.6|g.10905	1.62	6.84	56.15	1.66E-05	0.0015
7 days	twi_ss.28816.2|g.38575	2.80	5.27	56.05	1.68E-05	0.0015
7 days	twi_ss.25621.4|g.33682	-1.44	6.33	55.91	1.69E-05	0.0015
7 days	twi_ss.11705.1|g.15516	-1.03	8.15	55.69	1.72E-05	0.0016
7 days	twi_ss.12970.1|g.17215	1.92	6.09	55.64	1.73E-05	0.0016
7 days	twi_ss.17733.1|g.22605	-3.62	4.23	55.72	1.72E-05	0.0016
7 days	twi_ss.18540b.1|g.23638	1.73	6.83	55.68	1.73E-05	0.0016
7 days	twi_ss.22668.4|g.29253	-6.85	3.14	55.67	1.73E-05	0.0016
7 days	twi_ss.8953.1|g.12138	-2.61	5.87	55.67	1.73E-05	0.0016
7 days	twi_ss.31289.1|g.42555	-4.35	6.26	55.54	1.75E-05	0.0016
7 days	twi_ss.27623.2|g.36632	-1.23	7.22	55.48	1.75E-05	0.0016
7 days	twi_ss.15378.1|g.19918	-1.44	6.14	55.33	1.78E-05	0.0016
7 days	twi_ss.25745.1|g.33928	1.80	6.35	55.17	1.80E-05	0.0016
7 days	twi_ss.23517.1|g.30164	1.55	5.92	54.97	1.83E-05	0.0016
7 days	twi_ss.29547.1|g.39665	7.02	3.61	74.72	1.83E-05	0.0016

7 days	twi_ss.22914.1|g.29581	2.46	4.98	54.85	1.85E-05	0.0016
7 days	twi_ss.1036.1|g.1871	4.35	6.08	54.78	1.86E-05	0.0016
7 days	twi_ss.8099.3|g.11155	-1.50	6.38	54.77	1.86E-05	0.0016
7 days	twi_ss.21790.1|g.28289	-1.46	6.43	54.63	1.88E-05	0.0016
7 days	twi_ss.29546.1|g.39653	5.38	5.60	54.35	1.92E-05	0.0017
7 days	twi_ss.5716.1|g.8554	3.88	5.71	54.36	1.92E-05	0.0017
7 days	twi_ss.27623.1|g.36629	-2.74	9.16	54.17	1.95E-05	0.0017
7 days	twi_ss.21799.1|g.28294	1.17	7.61	54.11	1.96E-05	0.0017
7 days	twi_ss.29617.1|g.39880	3.89	9.59	54.07	1.97E-05	0.0017
7 days	twi_ss.2969.1|g.5263	-1.23	8.81	54.05	1.97E-05	0.0017
7 days	twi_ss.23297.1|g.29984	1.98	6.26	53.96	1.98E-05	0.0017
7 days	twi_ss.28014.2|g.37321	-6.05	2.61	53.63	2.04E-05	0.0017
7 days	twi_ss.9430.1|g.12699	4.46	4.18	53.50	2.06E-05	0.0017
7 days	twi_ss.17312.1|g.22090	-2.13	8.49	53.45	2.07E-05	0.0018
7 days	twi_ss.28101b.1|g.37486	3.76	5.15	53.05	2.14E-05	0.0018
7 days	twi_ss.30565a.6|g.41425	-3.02	8.28	52.97	2.15E-05	0.0018
7 days	twi_ss.16158.1|g.20747	1.59	6.81	52.88	2.17E-05	0.0018
7 days	twi_ss.19023.1|g.24422	2.58	6.51	52.84	2.18E-05	0.0018
7 days	twi_ss.1218.1|g.2194	-4.39	4.38	52.57	2.23E-05	0.0019
7 days	twi_ss.4484.1|g.6998	-3.29	6.38	52.56	2.23E-05	0.0019
7 days	twi_ss.14300.1|g.18619	-6.85	3.13	70.84	2.25E-05	0.0019
7 days	twi_ss.6603b.1|g.9643	3.31	8.46	52.09	2.32E-05	0.0019
7 days	twi_ss.160.1|g.288	-2.60	5.76	51.83	2.37E-05	0.0020
7 days	twi_ss.19956b.2|g.25691	1.41	6.65	51.78	2.38E-05	0.0020
7 days	twi_ss.23264.1|g.29908	-1.37	6.66	51.77	2.38E-05	0.0020
7 days	twi_ss.544.1|g.968	-2.46	5.47	51.70	2.40E-05	0.0020
7 days	twi_ss.21485.1|g.27864	1.02	9.85	51.50	2.44E-05	0.0020
7 days	twi_ss.24735.5|g.32332	3.07	5.60	51.49	2.44E-05	0.0020
7 days	twi_ss.31059.1|g.42230	-3.91	5.50	51.30	2.48E-05	0.0020
7 days	twi_ss.6274b.5|g.9297	0.74	7.53	51.35	2.47E-05	0.0020
7 days	twi_ss.8432.1|g.11616	0.74	7.20	51.31	2.48E-05	0.0020
7 days	twi_ss.13409.1|g.17726	-2.45	5.79	51.19	2.50E-05	0.0020
7 days	twi_ss.20452.1|g.26367	2.21	9.11	51.11	2.52E-05	0.0020

7 days	twi_ss.24955.1|g.32806	2.24	5.88	50.87	2.57E-05	0.0021
7 days	twi_ss.3973.1|g.6373	3.74	4.08	50.82	2.58E-05	0.0021
7 days	twi_ss.10588.1|g.14230	-8.17	6.24	50.64	2.63E-05	0.0021
7 days	twi_ss.11794.1|g.15702	0.99	7.47	50.66	2.62E-05	0.0021
7 days	twi_ss.28055.1|g.37390	1.31	7.97	50.63	2.63E-05	0.0021
7 days	twi_ss.31219.1|g.42419	2.09	6.87	50.66	2.62E-05	0.0021
7 days	twi_ss.1034.1|g.1887	-9.06	8.05	50.38	2.68E-05	0.0021
7 days	twi_ss.29206.1|g.39146	-1.95	6.90	50.19	2.73E-05	0.0021
7 days	twi_ss.10511.1|g.14144	0.76	7.98	50.14	2.74E-05	0.0022
7 days	twi_ss.2565.1|g.4595	-3.78	5.04	50.08	2.76E-05	0.0022
7 days	twi_ss.28752.1|g.38495	1.80	6.53	50.05	2.76E-05	0.0022
7 days	twi_ss.27012.1|g.35895	3.83	5.07	49.84	2.81E-05	0.0022
7 days	twi_ss.21349.1|g.27560	-8.26	8.36	49.76	2.83E-05	0.0022
7 days	twi_ss.22907.1|g.29571	-1.57	8.99	49.55	2.89E-05	0.0022
7 days	twi_ss.25248.1|g.33251	1.51	7.85	49.56	2.88E-05	0.0022
7 days	twi_ss.25248.2|g.33248	1.51	7.85	49.56	2.88E-05	0.0022
7 days	twi_ss.25939.3|g.34238	-3.86	4.95	49.39	2.93E-05	0.0023
7 days	twi_ss.21610.1|g.28014	2.18	5.82	49.18	2.98E-05	0.0023
7 days	twi_ss.28724.1|g.38426	-2.29	8.98	49.20	2.98E-05	0.0023
7 days	twi_ss.6818.1|g.9874	-1.40	6.84	49.14	2.99E-05	0.0023
7 days	twi_ss.26260.1|g.34793	-1.27	6.42	49.01	3.03E-05	0.0023
7 days	twi_ss.30027a.5|g.40531	8.52	4.96	49.02	3.03E-05	0.0023
7 days	twi_ss.20695.1|g.26791	1.66	6.71	48.95	3.05E-05	0.0023
7 days	twi_ss.28573.1|g.38252	1.86	6.36	48.89	3.06E-05	0.0023
7 days	twi_ss.14116a.1|g.18422	2.09	6.53	48.59	3.14E-05	0.0023
7 days	twi_ss.14119.1|g.18425	3.83	4.54	48.60	3.14E-05	0.0023
7 days	twi_ss.31413.1|g.42762	2.26	5.05	48.60	3.14E-05	0.0023
7 days	twi_ss.4716a.1|g.7265	0.91	6.46	48.58	3.15E-05	0.0023
7 days	twi_ss.5199.1|g.7825	-3.78	4.95	48.62	3.13E-05	0.0023
7 days	twi_ss.15787.1|g.20355	2.26	6.60	48.53	3.16E-05	0.0024
7 days	twi_ss.26110.1|g.34567	4.48	4.03	48.43	3.19E-05	0.0024
7 days	twi_ss.26323.1|g.34866	1.91	6.74	48.14	3.27E-05	0.0024
7 days	twi_ss.18906.1|g.24170	0.72	7.78	47.98	3.32E-05	0.0025

7 days	twi_ss.29246.1|g.39251	-2.00	6.12	47.94	3.33E-05	0.0025
7 days	twi_ss.1832.2|g.3219	1.43	6.02	47.91	3.34E-05	0.0025
7 days	twi_ss.26480.1|g.35123	2.70	5.79	47.87	3.35E-05	0.0025
7 days	twi_ss.29777.1|g.40102	-5.19	2.32	47.81	3.37E-05	0.0025
7 days	twi_ss.30351.1|g.41066	1.95	6.73	47.78	3.38E-05	0.0025
7 days	twi_ss.2830.1|g.5082	0.96	9.93	47.56	3.45E-05	0.0025
7 days	twi_ss.13807.1|g.18125	3.14	7.05	47.43	3.49E-05	0.0025
7 days	twi_ss.20951.1|g.27056	1.32	5.77	47.45	3.49E-05	0.0025
7 days	twi_ss.23705.1|g.30520	-1.83	6.01	47.26	3.55E-05	0.0026
7 days	twi_ss.14250.1|g.18561	1.41	5.91	47.17	3.58E-05	0.0026
7 days	twi_ss.6182.1|g.9161	-3.65	8.14	47.19	3.57E-05	0.0026
7 days	twi_ss.20204.1|g.26000	1.88	8.93	47.02	3.63E-05	0.0026
7 days	twi_ss.21016.1|g.27185	1.53	6.25	47.00	3.63E-05	0.0026
7 days	twi_ss.12162.1|g.16141	0.86	7.74	46.85	3.68E-05	0.0026
7 days	twi_ss.28150.1|g.37508	-2.41	5.63	46.89	3.67E-05	0.0026
7 days	twi_ss.29566.1|g.39761	1.71	7.88	46.87	3.68E-05	0.0026
7 days	twi_ss.9393.1|g.12653	1.28	6.60	46.89	3.67E-05	0.0026
7 days	twi_ss.4370.1|g.6876	3.65	6.49	46.76	3.71E-05	0.0026
7 days	twi_ss.6109.1|g.8976	1.94	6.25	46.74	3.72E-05	0.0026
7 days	twi_ss.19960b.5|g.25659	-9.23	5.06	61.87	3.74E-05	0.0026
7 days	twi_ss.5298a.2|g.8044	1.25	6.43	46.60	3.77E-05	0.0027
7 days	twi_ss.26771.1|g.35501	1.93	5.31	46.51	3.80E-05	0.0027
7 days	twi_ss.2764.1|g.4998	0.89	8.88	46.53	3.80E-05	0.0027
7 days	twi_ss.2800a.2|g.5025	2.12	9.06	46.56	3.79E-05	0.0027
7 days	twi_ss.28494.1|g.38157	1.70	6.61	46.45	3.82E-05	0.0027
7 days	twi_ss.5273.1|g.7993	-10.46	10.33	61.47	3.84E-05	0.0027
7 days	twi_ss.18595.1|g.23781	4.52	5.80	46.36	3.86E-05	0.0027
7 days	twi_ss.23805.1|g.30764	6.06	3.94	46.30	3.88E-05	0.0027
7 days	twi_ss.20935.1|g.27048	-3.46	5.89	46.15	3.93E-05	0.0027
7 days	twi_ss.6155.1|g.9054	-2.10	4.86	45.99	3.99E-05	0.0027
7 days	twi_ss.25143.1|g.33053	-3.02	4.85	45.46	4.20E-05	0.0029
7 days	twi_ss.8361a.1|g.11511	0.85	7.31	45.43	4.21E-05	0.0029
7 days	twi_ss.2531.2|g.4548	5.64	3.08	59.74	4.27E-05	0.0029

7 days	twi_ss.30372.1|g.41048	-3.85	4.59	45.18	4.31E-05	0.0029
7 days	twi_ss.20345.1|g.26181	-1.25	5.89	44.99	4.39E-05	0.0030
7 days	twi_ss.11279.1|g.14907	-3.41	4.16	44.79	4.47E-05	0.0030
7 days	twi_ss.24962.1|g.32827	1.55	5.87	44.62	4.54E-05	0.0031
7 days	twi_ss.23853b.2|g.30943	2.13	6.69	44.58	4.56E-05	0.0031
7 days	twi_ss.24028.2|g.31262	3.30	5.27	44.52	4.59E-05	0.0031
7 days	twi_ss.21748.1|g.28212	-3.75	4.40	44.38	4.65E-05	0.0031
7 days	twi_ss.28487.3|g.38053	2.41	6.91	44.12	4.77E-05	0.0032
7 days	twi_ss.24443a.2|g.32034	-3.68	4.93	44.07	4.80E-05	0.0032
7 days	twi_ss.11541a.1|g.15331	0.91	6.61	43.99	4.83E-05	0.0032
7 days	twi_ss.4977.9|g.7534	8.49	6.96	43.92	4.87E-05	0.0032
7 days	twi_ss.235.6|g.443	1.89	6.70	43.78	4.93E-05	0.0033
7 days	twi_ss.19481.1|g.25027	-4.87	4.64	43.68	4.98E-05	0.0033
7 days	twi_ss.1877.1|g.3338	-2.52	6.06	43.61	5.01E-05	0.0033
7 days	twi_ss.18605.1|g.23719	-6.01	3.01	57.07	5.06E-05	0.0033
7 days	twi_ss.24622.1|g.32277	-5.06	3.97	43.50	5.07E-05	0.0033
7 days	twi_ss.24847.1|g.32681	1.39	5.83	43.53	5.06E-05	0.0033
7 days	twi_ss.478.1|g.870	-3.60	4.63	43.41	5.11E-05	0.0034
7 days	twi_ss.27868.1|g.37038	1.43	8.63	43.25	5.19E-05	0.0034
7 days	twi_ss.29971.4|g.40439	1.43	6.26	43.23	5.21E-05	0.0034
7 days	twi_ss.29971.5|g.40444	1.43	6.26	43.23	5.21E-05	0.0034
7 days	twi_ss.19210.1|g.24594	0.76	8.35	43.14	5.25E-05	0.0034
7 days	twi_ss.4614.1|g.7136	-2.52	4.70	43.05	5.30E-05	0.0034
7 days	twi_ss.14947.1|g.19373	-2.35	5.13	42.95	5.35E-05	0.0035
7 days	twi_ss.31321a.2|g.42685	-3.46	4.83	42.95	5.35E-05	0.0035
7 days	twi_ss.27623.4|g.36634	-2.28	6.29	42.87	5.39E-05	0.0035
7 days	twi_ss.10279.1|g.13918	6.13	3.45	56.04	5.41E-05	0.0035
7 days	twi_ss.29980.2|g.40459	-2.99	5.07	42.70	5.49E-05	0.0035
7 days	twi_ss.1289.1|g.2303	-1.32	9.19	42.64	5.52E-05	0.0035
7 days	twi_ss.28921.1|g.38793	-8.14	4.19	88.55	5.54E-05	0.0036
7 days	twi_ss.17219a.12|g.21775	0.67	9.25	42.57	5.56E-05	0.0036
7 days	twi_ss.6923.2|g.10001	-3.04	7.64	42.53	5.58E-05	0.0036
7 days	twi_ss.12144.1|g.16027	0.85	7.87	42.44	5.63E-05	0.0036

7 days	twi_ss.3076.1|g.5470	3.54	4.69	42.45	5.63E-05	0.0036
7 days	twi_ss.24304.1|g.31690	1.06	9.51	42.39	5.66E-05	0.0036
7 days	twi_ss.3975a.1|g.6406	1.12	7.31	42.36	5.68E-05	0.0036
7 days	twi_ss.4579.1|g.7109	-2.93	5.37	42.35	5.68E-05	0.0036
7 days	twi_ss.27005.1|g.35887	-1.89	5.08	42.25	5.74E-05	0.0036
7 days	twi_ss.2412.1|g.4290	1.24	6.36	42.20	5.77E-05	0.0036
7 days	twi_ss.25737.1|g.33901	-1.19	6.95	42.17	5.79E-05	0.0036
7 days	twi_ss.14825.1|g.19285	-2.21	4.19	42.10	5.83E-05	0.0036
7 days	twi_ss.14795.1|g.19270	-2.14	6.34	41.91	5.94E-05	0.0037
7 days	twi_ss.27159a.4|g.36070	1.89	5.89	41.88	5.96E-05	0.0037
7 days	twi_ss.28361.1|g.37927	1.45	6.65	41.84	5.98E-05	0.0037
7 days	twi_ss.3000.1|g.5329	0.79	6.76	41.85	5.98E-05	0.0037
7 days	twi_ss.28429.1|g.38122	3.45	5.57	41.75	6.04E-05	0.0037
7 days	twi_ss.2981.1|g.5305	-2.84	5.12	41.76	6.03E-05	0.0037
7 days	twi_ss.30027b.1|g.40526	3.36	7.72	41.78	6.02E-05	0.0037
7 days	twi_ss.28187.1|g.37571	-5.97	5.82	41.70	6.07E-05	0.0037
7 days	twi_ss.7858.3|g.10907	2.50	6.05	41.56	6.15E-05	0.0038
7 days	twi_ss.23671.1|g.30499	1.15	7.19	41.39	6.27E-05	0.0038
7 days	twi_ss.12505a.1|g.16597	1.12	6.85	41.36	6.28E-05	0.0038
7 days	twi_ss.21339.5|g.27594	-4.89	2.19	45.32	6.38E-05	0.0039
7 days	twi_ss.5001.1|g.7619	1.69	7.18	41.05	6.49E-05	0.0040
7 days	twi_ss.28364.1|g.37940	1.53	5.82	41.02	6.51E-05	0.0040
7 days	twi_ss.27731.1|g.36953	2.73	5.07	40.87	6.61E-05	0.0040
7 days	twi_ss.16912.2|g.21548	-5.83	3.67	40.63	6.78E-05	0.0041
7 days	twi_ss.22992.1|g.29637	-3.16	5.79	40.66	6.76E-05	0.0041
7 days	twi_ss.26161.1|g.34639	-2.78	6.66	40.62	6.78E-05	0.0041
7 days	twi_ss.24384.1|g.31948	1.26	7.13	40.54	6.84E-05	0.0041
7 days	twi_ss.17899.1|g.22790	3.26	5.10	40.44	6.91E-05	0.0042
7 days	twi_ss.12920.1|g.17109	2.77	5.05	40.31	7.00E-05	0.0042
7 days	twi_ss.6575.1|g.9608	-4.05	4.06	40.15	7.12E-05	0.0043
7 days	twi_ss.12104.1|g.16038	-2.92	3.98	40.06	7.19E-05	0.0043
7 days	twi_ss.12874.1|g.17069	-1.23	7.27	39.90	7.31E-05	0.0043
7 days	twi_ss.28839.1|g.38609	-3.34	4.86	39.90	7.32E-05	0.0043

7 days	twi_ss.503.1|g.907	-1.64	5.54	39.92	7.30E-05	0.0043
7 days	twi_ss.6558.5|g.9595	2.69	4.82	51.69	7.29E-05	0.0043
7 days	twi_ss.14756.4|g.19195	3.34	4.93	39.84	7.36E-05	0.0044
7 days	twi_ss.25742.1|g.33929	2.08	4.74	39.82	7.38E-05	0.0044
7 days	twi_ss.22274.1|g.28804	1.48	6.72	39.79	7.40E-05	0.0044
7 days	twi_ss.6079.1|g.8932	1.35	7.81	39.74	7.44E-05	0.0044
7 days	twi_ss.908.1|g.1722	1.86	5.39	39.61	7.54E-05	0.0044
7 days	twi_ss.31037.1|g.42133	1.47	5.86	39.50	7.63E-05	0.0045
7 days	twi_ss.7858.13|g.10902	2.54	6.11	39.43	7.69E-05	0.0045
7 days	twi_ss.29133.1|g.39099	1.19	5.51	39.30	7.80E-05	0.0046
7 days	twi_ss.3088.1|g.5481	-3.88	4.60	39.20	7.88E-05	0.0046
7 days	twi_ss.2312b.1|g.4062	4.80	2.45	53.96	7.97E-05	0.0046
7 days	twi_ss.3007.1|g.5346	2.18	5.33	39.10	7.96E-05	0.0046
7 days	twi_ss.20264c.8|g.26050	7.48	4.91	50.24	8.09E-05	0.0047
7 days	twi_ss.17758.1|g.22589	1.06	10.53	38.89	8.14E-05	0.0047
7 days	twi_ss.24269.1|g.31712	2.15	6.62	38.86	8.17E-05	0.0047
7 days	twi_ss.9086.2|g.12276	1.04	7.83	38.81	8.22E-05	0.0047
7 days	twi_ss.28487.2|g.38056	2.25	7.83	38.70	8.31E-05	0.0048
7 days	twi_ss.8324b.1|g.11420	1.00	6.13	38.69	8.32E-05	0.0048
7 days	twi_ss.5561.1|g.8433	-1.82	7.51	38.66	8.35E-05	0.0048
7 days	twi_ss.10516.1|g.14150	4.81	5.54	38.60	8.40E-05	0.0048
7 days	twi_ss.2045.1|g.3673	-1.41	5.26	38.59	8.41E-05	0.0048
7 days	twi_ss.20175.1|g.25982	1.10	6.26	38.53	8.47E-05	0.0048
7 days	twi_ss.25669.1|g.33849	-2.60	4.53	38.48	8.51E-05	0.0048
7 days	twi_ss.5415.4|g.8178	3.58	5.56	38.50	8.50E-05	0.0048
7 days	twi_ss.25137.1|g.33020	1.67	9.36	38.46	8.53E-05	0.0048
7 days	twi_ss.27170b.2|g.36114	1.44	6.83	38.40	8.59E-05	0.0049
7 days	twi_ss.22791.1|g.29420	-3.62	4.45	38.29	8.69E-05	0.0049
7 days	twi_ss.18299.1|g.23330	-1.32	7.94	38.18	8.79E-05	0.0049
7 days	twi_ss.31288.1|g.42543	-7.84	4.01	49.12	8.79E-05	0.0049
7 days	twi_ss.1051.1|g.1890	1.60	4.96	38.16	8.82E-05	0.0050
7 days	twi_ss.4654.1|g.7188	2.42	6.57	38.13	8.85E-05	0.0050
7 days	twi_ss.23802.1|g.30763	5.84	3.57	48.99	8.87E-05	0.0050

7 days	twi_ss.22383.1|g.28898	-3.88	4.26	38.05	8.92E-05	0.0050
7 days	twi_ss.23819.1|g.30881	2.63	4.36	38.08	8.90E-05	0.0050
7 days	twi_ss.29236.1|g.39201	1.12	7.03	38.05	8.93E-05	0.0050
7 days	twi_ss.25372.1|g.33400	-2.29	6.66	38.02	8.96E-05	0.0050
7 days	twi_ss.27170b.1|g.36115	1.37	7.08	37.86	9.11E-05	0.0051
7 days	twi_ss.20867.1|g.26998	-4.61	4.96	37.81	9.16E-05	0.0051
7 days	twi_ss.1966.1|g.3484	1.94	6.32	37.74	9.23E-05	0.0051
7 days	twi_ss.5387.1|g.8143	-1.00	7.38	37.70	9.27E-05	0.0051
7 days	twi_ss.1881.1|g.3310	-2.38	5.98	37.67	9.31E-05	0.0051
7 days	twi_ss.524.1|g.947	-3.18	4.40	37.64	9.34E-05	0.0051
7 days	twi_ss.7808.1|g.10888	0.90	6.42	37.62	9.36E-05	0.0051
7 days	twi_ss.982c.1|g.1756	2.18	6.42	37.41	9.58E-05	0.0052
7 days	twi_ss.718.2|g.1251	-10.05	7.70	47.94	9.60E-05	0.0052
7 days	twi_ss.21280.1|g.27544	-5.26	4.47	37.35	9.65E-05	0.0053
7 days	twi_ss.18751.1|g.23960	-4.24	3.34	37.32	9.67E-05	0.0053
7 days	twi_ss.25926.1|g.34242	-1.99	5.49	37.20	9.81E-05	0.0053
7 days	twi_ss.16815.1|g.21469	-1.35	5.95	37.17	9.84E-05	0.0053
7 days	twi_ss.14588.3|g.19041	-8.14	5.00	37.15	9.86E-05	0.0053
7 days	twi_ss.28038b.2|g.37332	5.08	4.50	37.11	9.90E-05	0.0054
7 days	twi_ss.29460.1|g.39706	-5.87	3.22	47.50	9.93E-05	0.0054
7 days	twi_ss.21631b.4|g.28006	3.89	5.43	37.05	9.97E-05	0.0054
7 days	twi_ss.13032.1|g.17262	-1.66	5.69	36.97	0.0001	0.0054
7 days	twi_ss.4355.1|g.6871	2.37	5.77	36.96	0.0001	0.0054
7 days	twi_ss.6950.1|g.10039	1.31	5.85	36.94	0.0001	0.0054
7 days	twi_ss.30657b.1|g.41506	-2.24	5.94	36.83	0.0001	0.0055
7 days	twi_ss.7929.1|g.10983	0.99	6.20	36.71	0.0001	0.0055
7 days	twi_ss.9652.1|g.12945	-6.23	4.17	36.70	0.0001	0.0055
7 days	twi_ss.3976.1|g.6397	-3.06	4.71	36.65	0.0001	0.0056
7 days	twi_ss.1660.1|g.2916	-3.60	5.19	36.56	0.0001	0.0056
7 days	twi_ss.21575.1|g.27963	-2.46	4.48	36.53	0.0001	0.0056
7 days	twi_ss.23575.1|g.30271	2.49	6.28	36.54	0.0001	0.0056
7 days	twi_ss.26633b.1|g.35303	-3.33	10.12	36.47	0.0001	0.0056
7 days	twi_ss.13587.1|g.17952	-3.01	7.70	36.43	0.0001	0.0056

7 days	twi_ss.3117.1|g.5501	2.05	5.94	36.40	0.0001	0.0057
7 days	twi_ss.22636.1|g.29230	-1.98	6.48	36.36	0.0001	0.0057
7 days	twi_ss.8401.1|g.11548	1.13	6.15	36.16	0.0001	0.0058
7 days	twi_ss.18630.1|g.23736	1.05	9.37	36.06	0.0001	0.0058
7 days	twi_ss.28605.1|g.38302	-7.07	4.94	36.04	0.0001	0.0058
7 days	twi_ss.31999.1|g.43620	1.25	7.54	36.00	0.0001	0.0059
7 days	twi_ss.23880.1|g.31029	4.23	11.32	35.96	0.0001	0.0059
7 days	twi_ss.849.1|g.1397	-1.34	5.99	35.93	0.0001	0.0059
7 days	twi_ss.15781.1|g.20345	2.35	5.60	35.87	0.0001	0.0059
7 days	twi_ss.25128.1|g.33004	-1.52	5.38	35.87	0.0001	0.0059
7 days	twi_ss.29224.1|g.39184	-1.62	6.29	35.75	0.0001	0.0060
7 days	twi_ss.558.1|g.1024	2.23	4.96	35.78	0.0001	0.0060
7 days	twi_ss.58.1|g.80	-1.85	7.27	35.73	0.0001	0.0060
7 days	twi_ss.6881.1|g.9943	3.25	5.46	35.73	0.0001	0.0060
7 days	twi_ss.8151.1|g.11240	1.22	6.18	35.73	0.0001	0.0060
7 days	twi_ss.20081.1|g.25853	1.04	6.31	35.61	0.0001	0.0060
7 days	twi_ss.17786.1|g.22684	-5.43	4.35	35.48	0.0001	0.0061
7 days	twi_ss.21577.1|g.27967	-3.76	4.21	35.48	0.0001	0.0061
7 days	twi_ss.23961.1|g.31180	1.56	6.32	35.48	0.0001	0.0061
7 days	twi_ss.6472.1|g.9500	0.98	9.46	35.49	0.0001	0.0061
7 days	twi_ss.25621.2|g.33688	6.45	3.74	35.33	0.0001	0.0062
7 days	twi_ss.5112.1|g.7738	3.05	5.43	35.33	0.0001	0.0062
7 days	twi_ss.21211.1|g.27438	1.87	5.60	35.25	0.0001	0.0062
7 days	twi_ss.7409.1|g.10417	3.01	4.68	35.26	0.0001	0.0062
7 days	twi_ss.6152b.2|g.9126	1.54	6.76	35.19	0.0001	0.0062
7 days	twi_ss.22375.1|g.28920	-3.04	4.87	35.15	0.0001	0.0063
7 days	twi_ss.12187.1|g.16151	1.69	5.09	35.08	0.0001	0.0063
7 days	twi_ss.173.1|g.271	0.90	8.46	35.10	0.0001	0.0063
7 days	twi_ss.25472.1|g.33509	-2.00	4.13	35.10	0.0001	0.0063
7 days	twi_ss.30491.1|g.41266	1.75	5.67	35.09	0.0001	0.0063
7 days	twi_ss.11861.1|g.15753	-1.86	6.78	35.06	0.0001	0.0063
7 days	twi_ss.2321.1|g.4060	-1.62	5.52	35.05	0.0001	0.0063
7 days	twi_ss.31088b.4|g.42266	-1.99	4.74	35.00	0.0001	0.0063

7 days	twi_ss.11539a.1|g.15322	3.90	7.86	34.93	0.0001	0.0063
7 days	twi_ss.21579.1|g.27977	-3.01	4.77	34.91	0.0001	0.0063
7 days	twi_ss.6345.1|g.9359	0.72	7.13	34.88	0.0001	0.0063
7 days	twi_ss.19926.1|g.25684	0.93	9.57	34.85	0.0001	0.0064
7 days	twi_ss.22947.1|g.29606	-2.00	6.59	34.84	0.0001	0.0064
7 days	twi_ss.27582a.2|g.36743	1.60	5.93	34.79	0.0001	0.0064
7 days	twi_ss.6682.1|g.9749	1.35	7.81	34.74	0.0001	0.0064
7 days	twi_ss.11466.1|g.15114	-2.32	3.88	34.72	0.0001	0.0064
7 days	twi_ss.10775.1|g.14409	2.11	6.71	34.68	0.0001	0.0064
7 days	twi_ss.12698.1|g.16804	1.57	4.80	34.67	0.0001	0.0064
7 days	twi_ss.17799.1|g.22676	-3.04	10.10	34.66	0.0001	0.0064
7 days	twi_ss.981.1|g.1783	5.20	5.46	34.70	0.0001	0.0064
7 days	twi_ss.14576.1|g.19038	2.74	6.88	34.59	0.0001	0.0065
7 days	twi_ss.12248a.3|g.16318	1.41	7.10	34.54	0.0001	0.0065
7 days	twi_ss.23785a.2|g.30828	2.09	5.38	34.50	0.0001	0.0065
7 days	twi_ss.28878.1|g.38683	-2.54	3.89	34.48	0.0001	0.0065
7 days	twi_ss.11931.1|g.15823	0.78	7.86	34.40	0.0001	0.0066
7 days	twi_ss.18869.1|g.24133	-1.47	6.98	34.38	0.0001	0.0066
7 days	twi_ss.30219.1|g.40817	-1.30	6.67	34.39	0.0001	0.0066
7 days	twi_ss.27689.1|g.36875	0.82	7.68	34.26	0.0001	0.0067
7 days	twi_ss.5863.1|g.8715	-1.03	9.63	34.24	0.0001	0.0067
7 days	twi_ss.16288.1|g.20863	2.83	4.77	43.31	0.0001	0.0067
7 days	twi_ss.31680.4|g.43131	1.22	6.44	34.21	0.0001	0.0067
7 days	twi_ss.11553.1|g.15368	1.35	6.71	34.15	0.0001	0.0067
7 days	twi_ss.30809.1|g.41716	2.18	5.84	34.11	0.0001	0.0067
7 days	twi_ss.27247.2|g.36186	-2.85	7.65	33.94	0.0001	0.0068
7 days	twi_ss.1035.1|g.1876	3.94	5.08	33.90	0.0001	0.0069
7 days	twi_ss.23557.1|g.30254	-1.66	5.37	33.91	0.0001	0.0069
7 days	twi_ss.29979.1|g.40463	-2.43	4.62	33.81	0.0001	0.0069
7 days	twi_ss.26361a.1|g.34928	-7.13	6.15	42.62	0.0001	0.0070
7 days	twi_ss.19070a.4|g.24462	1.47	6.00	33.60	0.0001	0.0071
7 days	twi_ss.29908.1|g.40336	-1.71	4.69	33.60	0.0001	0.0071
7 days	twi_ss.6018.1|g.8879	6.47	6.32	33.58	0.0001	0.0071

7 days	twi_ss.31762.1|g.43257	1.26	6.74	33.43	0.0002	0.0072
7 days	twi_ss.5288.1|g.7947	-1.10	6.75	33.42	0.0002	0.0072
7 days	twi_ss.23834.1|g.30873	1.62	7.71	33.23	0.0002	0.0073
7 days	twi_ss.1956.1|g.3454	-1.24	5.91	33.10	0.0002	0.0074
7 days	twi_ss.22413.1|g.28953	1.30	6.69	33.10	0.0002	0.0074
7 days	twi_ss.25132.1|g.33040	-1.94	7.05	33.11	0.0002	0.0074
7 days	twi_ss.18781.1|g.23962	-0.92	7.23	33.06	0.0002	0.0075
7 days	twi_ss.2415.2|g.4288	0.82	7.75	33.04	0.0002	0.0075
7 days	twi_ss.14231.1|g.18498	-1.89	5.07	32.92	0.0002	0.0075
7 days	twi_ss.17937.1|g.22815	-3.75	5.29	32.96	0.0002	0.0075
7 days	twi_ss.26160.1|g.34649	-2.28	6.24	32.93	0.0002	0.0075
7 days	twi_ss.28156a.1|g.37511	-11.30	6.91	41.48	0.0002	0.0075
7 days	twi_ss.22743.11|g.29088	-1.84	5.56	32.89	0.0002	0.0075
7 days	twi_ss.11056b.2|g.14704	0.91	6.52	32.87	0.0002	0.0075
7 days	twi_ss.16520.1|g.21215	0.83	6.89	32.85	0.0002	0.0076
7 days	twi_ss.10027.1|g.13321	0.63	8.54	32.83	0.0002	0.0076
7 days	twi_ss.26444a.1|g.35016	1.05	7.98	32.73	0.0002	0.0076
7 days	twi_ss.11038.1|g.14666	1.31	5.27	32.67	0.0002	0.0077
7 days	twi_ss.30390.1|g.41135	1.41	6.97	32.67	0.0002	0.0077
7 days	twi_ss.108.1|g.188	3.00	5.72	32.62	0.0002	0.0077
7 days	twi_ss.18795.4|g.24047	3.01	7.57	32.61	0.0002	0.0077
7 days	twi_ss.24497.2|g.32088	3.54	5.08	32.62	0.0002	0.0077
7 days	twi_ss.28010.1|g.37326	1.48	5.77	32.64	0.0002	0.0077
7 days	twi_ss.1766.1|g.3105	1.87	6.37	32.49	0.0002	0.0078
7 days	twi_ss.30027c.4|g.40535	3.62	6.28	32.49	0.0002	0.0078
7 days	twi_ss.13145.1|g.17465	1.28	7.70	32.45	0.0002	0.0078
7 days	twi_ss.718.1|g.1252	-9.54	7.23	40.80	0.0002	0.0078
7 days	twi_ss.16059.1|g.20655	-1.05	6.89	32.34	0.0002	0.0079
7 days	twi_ss.29230.1|g.39192	-3.05	3.63	32.33	0.0002	0.0079
7 days	twi_ss.22038.1|g.28557	1.03	7.05	32.29	0.0002	0.0079
7 days	twi_ss.14577.1|g.19060	-1.90	5.49	32.18	0.0002	0.0080
7 days	twi_ss.21610.4|g.28010	2.69	6.64	32.11	0.0002	0.0081
7 days	twi_ss.2218.1|g.3944	-3.48	4.37	32.10	0.0002	0.0081

7 days	twi_ss.6415.1|g.9461	-1.28	8.11	32.11	0.0002	0.0081
7 days	twi_ss.21665.4|g.27899	2.39	4.65	32.08	0.0002	0.0081
7 days	twi_ss.13330.1|g.17615	-1.94	5.33	32.02	0.0002	0.0081
7 days	twi_ss.1966.2|g.3482	1.71	6.10	31.93	0.0002	0.0082
7 days	twi_ss.1931.1|g.3453	1.29	4.97	31.90	0.0002	0.0082
7 days	twi_ss.27392.1|g.36463	-2.21	4.60	31.90	0.0002	0.0082
7 days	twi_ss.15124.1|g.19641	2.05	7.05	31.83	0.0002	0.0083
7 days	twi_ss.28839.2|g.38607	-2.49	4.20	31.80	0.0002	0.0083
7 days	twi_ss.4860.1|g.7466	-1.54	6.00	31.80	0.0002	0.0083
7 days	twi_ss.3349.1|g.5752	-3.11	4.43	31.79	0.0002	0.0083
7 days	twi_ss.11428.1|g.15070	1.04	6.23	31.76	0.0002	0.0083
7 days	twi_ss.15661.2|g.20228	2.25	5.75	31.74	0.0002	0.0083
7 days	twi_ss.16421.1|g.21003	-2.88	5.22	31.74	0.0002	0.0083
7 days	twi_ss.8759.1|g.11942	5.21	4.86	31.75	0.0002	0.0083
7 days	twi_ss.14291a.1|g.18602	-2.33	4.24	31.67	0.0002	0.0083
7 days	twi_ss.30872.1|g.41915	0.99	6.70	31.69	0.0002	0.0083
7 days	twi_ss.8878b.2|g.12094	-5.20	3.67	31.67	0.0002	0.0083
7 days	twi_ss.28912.1|g.38856	5.34	2.81	39.55	0.0002	0.0084
7 days	twi_ss.4127.1|g.6552	-2.36	5.25	31.57	0.0002	0.0084
7 days	twi_ss.17303.1|g.22085	1.81	11.19	31.49	0.0002	0.0084
7 days	twi_ss.19469.1|g.24973	1.09	7.59	31.50	0.0002	0.0084
7 days	twi_ss.19501.1|g.25037	1.48	5.66	31.53	0.0002	0.0084
7 days	twi_ss.20264c.13|g.26051	1.39	7.54	31.51	0.0002	0.0084
7 days	twi_ss.23133.1|g.29738	1.49	7.15	31.51	0.0002	0.0084
7 days	twi_ss.11112.1|g.14736	-4.24	4.76	31.45	0.0002	0.0084
7 days	twi_ss.17908.1|g.22797	3.45	4.70	31.44	0.0002	0.0084
7 days	twi_ss.29223.2|g.39194	-6.50	3.92	31.41	0.0002	0.0085
7 days	twi_ss.27700.1|g.36906	0.88	6.19	31.36	0.0002	0.0085
7 days	twi_ss.481.1|g.863	0.60	8.63	31.30	0.0002	0.0086
7 days	twi_ss.27413.1|g.36501	-3.73	4.31	31.28	0.0002	0.0086
7 days	twi_ss.2820.1|g.5093	-1.41	5.23	31.21	0.0002	0.0086
7 days	twi_ss.24776.1|g.32540	-2.37	6.84	31.16	0.0002	0.0087
7 days	twi_ss.25090.1|g.32912	1.78	5.95	31.13	0.0002	0.0087

7 days	twi_ss.10005.1|g.13311	-2.75	3.84	31.06	0.0002	0.0088
7 days	twi_ss.22218e.7|g.28734	7.81	4.53	38.77	0.0002	0.0088
7 days	twi_ss.24735.7|g.32340	2.68	5.84	31.00	0.0002	0.0088
7 days	twi_ss.11539b.4|g.15321	4.07	8.46	30.95	0.0002	0.0089
7 days	twi_ss.28476.1|g.38101	1.19	6.68	30.89	0.0002	0.0089
7 days	twi_ss.30803a.1|g.41752	2.99	6.26	30.86	0.0002	0.0089
7 days	twi_ss.19570.3|g.25107	1.17	6.27	30.83	0.0002	0.0090
7 days	twi_ss.2067.1|g.3656	-1.48	5.41	30.79	0.0002	0.0090
7 days	twi_ss.3244.1|g.5651	-1.68	5.73	30.77	0.0002	0.0090
7 days	twi_ss.26746.1|g.35393	0.84	10.41	30.71	0.0002	0.0091
7 days	twi_ss.28280.1|g.37812	0.82	8.58	30.65	0.0002	0.0091
7 days	twi_ss.16055.1|g.20656	-3.79	4.03	30.61	0.0002	0.0091
7 days	twi_ss.24621.1|g.32287	-2.47	4.90	30.62	0.0002	0.0091
7 days	twi_ss.30982.1|g.42019	0.69	9.70	30.61	0.0002	0.0091
7 days	twi_ss.3009.2|g.5339	-3.95	5.25	30.55	0.0002	0.0092
7 days	twi_ss.9920.2|g.13257	5.23	2.76	30.47	0.0002	0.0093
7 days	twi_ss.805.1|g.1398	1.05	7.93	30.31	0.0002	0.0095
7 days	twi_ss.1117.1|g.1988	-2.02	6.37	30.27	0.0002	0.0095
7 days	twi_ss.20469.1|g.26384	-4.86	4.98	30.27	0.0002	0.0095
7 days	twi_ss.22436.1|g.28973	-1.74	7.76	30.23	0.0002	0.0095
7 days	twi_ss.20330.1|g.26166	0.94	6.18	30.21	0.0002	0.0095
7 days	twi_ss.5416.1|g.8182	1.97	6.06	30.19	0.0002	0.0095
7 days	twi_ss.19822b.1|g.25495	-3.82	5.27	30.17	0.0002	0.0095
7 days	twi_ss.29239.1|g.39216	-5.11	3.77	37.51	0.0002	0.0096
7 days	twi_ss.30208.2|g.40799	-2.85	4.65	30.13	0.0002	0.0096
7 days	twi_ss.686.1|g.1225	-2.59	4.34	30.07	0.0002	0.0096
7 days	twi_ss.31144.1|g.42355	-1.85	5.39	30.01	0.0002	0.0097
7 days	twi_ss.21664c.8|g.27921	2.81	5.60	29.95	0.0002	0.0098
7 days	twi_ss.29590a.5|g.39853	4.27	6.74	29.87	0.0002	0.0099
7 days	twi_ss.4264.1|g.6704	3.71	4.78	29.79	0.0002	0.0099
7 days	twi_ss.12538.1|g.16681	-2.02	4.16	29.76	0.0002	0.0100
7 days	twi_ss.27006a.1|g.35879	1.04	6.73	29.72	0.0002	0.0100
7 days	twi_ss.30485.1|g.41291	-2.90	3.72	29.71	0.0002	0.0100

7 days	twi_ss.19899.1|g.25651	1.43	6.74	29.68	0.0002	0.0100
7 days	twi_ss.20029.1|g.25796	-1.84	4.78	29.66	0.0002	0.0100
7 days	twi_ss.27623.6|g.36625	1.15	8.21	29.67	0.0002	0.0100
7 days	twi_ss.9859.1|g.13191	1.71	5.04	29.62	0.0002	0.0101
7 days	twi_ss.12861.1|g.17029	0.91	6.75	29.60	0.0002	0.0101
7 days	twi_ss.13791.2|g.18112	1.89	5.47	29.57	0.0002	0.0101
7 days	twi_ss.19562.1|g.25130	0.96	7.69	29.57	0.0002	0.0101
7 days	twi_ss.22201a.1|g.28770	-1.27	6.17	29.56	0.0002	0.0101
7 days	twi_ss.32100.1|g.43763	-3.14	4.96	29.58	0.0002	0.0101
7 days	twi_ss.2485.1|g.4550	2.32	4.35	29.45	0.0003	0.0102
7 days	twi_ss.115.1|g.206	-1.14	7.10	29.43	0.0003	0.0102
7 days	twi_ss.984.1|g.1782	4.58	4.43	29.35	0.0003	0.0103
7 days	twi_ss.23836a.2|g.30870	-1.19	7.43	29.33	0.0003	0.0104
7 days	twi_ss.12345.1|g.16456	1.02	5.87	29.23	0.0003	0.0105
7 days	twi_ss.7082.1|g.10151	2.55	6.32	29.19	0.0003	0.0105
7 days	twi_ss.9709.1|g.13070	6.46	3.43	29.15	0.0003	0.0106
7 days	twi_ss.261.1|g.548	-1.53	5.84	29.04	0.0003	0.0107
7 days	twi_ss.29544b.2|g.39679	1.38	6.83	28.97	0.0003	0.0108
7 days	twi_ss.110b.1|g.193	2.85	7.30	28.94	0.0003	0.0108
7 days	twi_ss.36.1|g.14	1.36	5.70	28.92	0.0003	0.0108
7 days	twi_ss.30423.1|g.41204	-1.62	7.12	28.90	0.0003	0.0109
7 days	twi_ss.24330.1|g.31799	1.31	5.61	28.89	0.0003	0.0109
7 days	twi_ss.27216.1|g.36124	2.08	4.56	28.80	0.0003	0.0110
7 days	twi_ss.13842.1|g.18147	-2.38	10.84	28.77	0.0003	0.0110
7 days	twi_ss.17104.1|g.21883	-3.85	4.23	28.77	0.0003	0.0110
7 days	twi_ss.2604.1|g.4680	2.12	6.16	28.76	0.0003	0.0110
7 days	twi_ss.12876.1|g.17052	-1.39	6.47	28.65	0.0003	0.0111
7 days	twi_ss.13654.1|g.18011	-3.35	4.27	28.66	0.0003	0.0111
7 days	twi_ss.19934.1|g.25674	1.02	6.77	28.66	0.0003	0.0111
7 days	twi_ss.10988.1|g.14628	1.34	4.86	28.62	0.0003	0.0112
7 days	twi_ss.31732.1|g.43215	-1.46	6.50	28.61	0.0003	0.0112
7 days	twi_ss.26983.1|g.35830	-1.23	6.04	28.55	0.0003	0.0112
7 days	twi_ss.30133a.1|g.40634	-0.95	7.29	28.50	0.0003	0.0113

7 days	twi_ss.30890.1|g.41957	1.13	7.10	28.49	0.0003	0.0113
7 days	twi_ss.14818.1|g.19280	-2.81	5.62	28.48	0.0003	0.0113
7 days	twi_ss.14839.1|g.19294	6.68	3.62	28.47	0.0003	0.0113
7 days	twi_ss.1770b.2|g.3120	-1.92	4.07	28.45	0.0003	0.0113
7 days	twi_ss.19607.1|g.25175	0.63	7.80	28.44	0.0003	0.0113
7 days	twi_ss.15849.1|g.20416	2.35	5.05	28.42	0.0003	0.0113
7 days	twi_ss.3562.1|g.5942	-1.49	5.20	28.40	0.0003	0.0113
7 days	twi_ss.4165.1|g.6566	0.74	7.60	28.40	0.0003	0.0113
7 days	twi_ss.19384.1|g.24874	9.98	6.19	35.00	0.0003	0.0114
7 days	twi_ss.11751.1|g.15579	-3.54	4.15	28.33	0.0003	0.0114
7 days	twi_ss.3937.1|g.6352	-1.02	5.43	28.30	0.0003	0.0114
7 days	twi_ss.5058.2|g.7681	3.80	6.26	28.29	0.0003	0.0115
7 days	twi_ss.12114.1|g.16084	1.21	7.05	28.24	0.0003	0.0115
7 days	twi_ss.20449b.1|g.26353	-4.26	4.07	28.25	0.0003	0.0115
7 days	twi_ss.12164.1|g.16143	0.89	6.83	28.22	0.0003	0.0115
7 days	twi_ss.25370.1|g.33401	-4.60	4.50	28.20	0.0003	0.0115
7 days	twi_ss.30862.1|g.42102	-1.35	5.47	28.20	0.0003	0.0115
7 days	twi_ss.5694.1|g.8531	-3.61	3.98	28.20	0.0003	0.0115
7 days	twi_ss.2800a.4|g.5028	2.41	8.46	28.18	0.0003	0.0115
7 days	twi_ss.32005b.4|g.43639	1.39	6.26	28.15	0.0003	0.0116
7 days	twi_ss.12009.1|g.15919	1.06	7.46	28.11	0.0003	0.0116
7 days	twi_ss.16882.1|g.21509	2.03	5.32	28.11	0.0003	0.0116
7 days	twi_ss.19030.3|g.24370	-2.57	9.66	28.11	0.0003	0.0116
7 days	twi_ss.29748.1|g.40169	1.37	6.02	28.08	0.0003	0.0116
7 days	twi_ss.10376.1|g.14023	1.57	5.44	28.07	0.0003	0.0116
7 days	twi_ss.1414.1|g.2446	0.88	7.82	28.04	0.0003	0.0116
7 days	twi_ss.25312.1|g.33265	2.79	5.02	28.03	0.0003	0.0117
7 days	twi_ss.12814.1|g.16931	-1.92	5.64	28.01	0.0003	0.0117
7 days	twi_ss.4149.1|g.6565	0.73	9.21	27.99	0.0003	0.0117
7 days	twi_ss.28187.2|g.37570	-8.08	4.09	27.97	0.0003	0.0117
7 days	twi_ss.26924.1|g.35731	1.06	6.17	27.96	0.0003	0.0117
7 days	twi_ss.25771b.1|g.33558	-2.31	8.46	27.93	0.0003	0.0117
7 days	twi_ss.28547.1|g.38243	-1.89	7.24	27.94	0.0003	0.0117

7 days	twi_ss.18124.1|g.23044	-4.47	3.79	27.92	0.0003	0.0117
7 days	twi_ss.20410.1|g.26292	-5.27	3.46	34.37	0.0003	0.0117
7 days	twi_ss.25132.2|g.33043	-1.64	5.65	27.91	0.0003	0.0117
7 days	twi_ss.7489.1|g.10522	-3.63	5.65	27.89	0.0003	0.0117
7 days	twi_ss.14763.1|g.19250	-1.12	6.23	27.87	0.0003	0.0117
7 days	twi_ss.8843.1|g.12032	6.82	3.16	34.29	0.0003	0.0118
7 days	twi_ss.29330.1|g.39379	0.97	7.58	27.78	0.0003	0.0119
7 days	twi_ss.5087.1|g.7753	-8.47	4.31	34.21	0.0003	0.0119
7 days	twi_ss.15760.1|g.20329	-5.32	4.25	27.71	0.0003	0.0120
7 days	twi_ss.9509.1|g.12773	3.54	4.42	27.65	0.0003	0.0120
7 days	twi_ss.28025.1|g.37315	-1.61	5.73	27.64	0.0003	0.0120
7 days	twi_ss.22789.1|g.29433	-2.56	5.87	27.62	0.0003	0.0121
7 days	twi_ss.31822.1|g.43347	1.07	6.20	27.57	0.0003	0.0121
7 days	twi_ss.5977.2|g.8846	-2.78	3.79	27.52	0.0003	0.0122
7 days	twi_ss.16056.1|g.20654	-4.77	4.00	27.50	0.0003	0.0122
7 days	twi_ss.4661.2|g.7185	0.93	6.38	27.44	0.0003	0.0123
7 days	twi_ss.17964.1|g.22828	2.84	8.67	27.42	0.0003	0.0123
7 days	twi_ss.5622.1|g.8380	0.98	6.08	27.37	0.0003	0.0124
7 days	twi_ss.25056.1|g.32945	0.86	6.62	27.32	0.0003	0.0125
7 days	twi_ss.1441.1|g.2632	1.17	5.51	27.31	0.0003	0.0125
7 days	twi_ss.16604.1|g.21254	-1.98	7.50	27.29	0.0003	0.0125
7 days	twi_ss.27562.2|g.36725	4.06	7.95	27.25	0.0003	0.0125
7 days	twi_ss.8930b.1|g.12126	-1.81	5.19	27.26	0.0003	0.0125
7 days	twi_ss.27814.1|g.37026	-1.53	5.37	27.22	0.0003	0.0126
7 days	twi_ss.19345.12|g.24807	-6.57	2.90	47.62	0.0003	0.0126
7 days	twi_ss.21760.1|g.28222	0.93	7.59	27.12	0.0003	0.0127
7 days	twi_ss.19625.1|g.25181	2.24	5.01	27.09	0.0004	0.0128
7 days	twi_ss.21412b.2|g.27660	1.78	4.73	27.09	0.0004	0.0128
7 days	twi_ss.1346.1|g.2430	-2.77	4.25	27.08	0.0004	0.0128
7 days	twi_ss.8193.1|g.11281	1.00	5.72	27.07	0.0004	0.0128
7 days	twi_ss.6679.1|g.9780	1.04	7.29	27.06	0.0004	0.0128
7 days	twi_ss.5526b.1|g.8322	3.94	6.41	27.03	0.0004	0.0128
7 days	twi_ss.13856.1|g.18170	2.30	4.29	26.97	0.0004	0.0129

7 days	twi_ss.3408.1|g.5790	-2.97	4.19	26.95	0.0004	0.0129
7 days	twi_ss.29443.2|g.39650	1.97	6.06	26.93	0.0004	0.0130
7 days	twi_ss.22308.1|g.28854	-6.35	3.23	46.70	0.0004	0.0131
7 days	twi_ss.27674.1|g.36836	-1.02	5.99	26.80	0.0004	0.0131
7 days	twi_ss.13146.9|g.17473	4.39	5.22	26.79	0.0004	0.0132
7 days	twi_ss.19885.1|g.25650	2.96	3.94	26.78	0.0004	0.0132
7 days	twi_ss.3645.1|g.6020	0.71	7.42	26.74	0.0004	0.0132
7 days	twi_ss.31896.1|g.43422	0.80	6.27	26.72	0.0004	0.0132
7 days	twi_ss.13471.2|g.17804	1.28	6.04	26.70	0.0004	0.0133
7 days	twi_ss.5271.1|g.7892	-0.80	6.87	26.69	0.0004	0.0133
7 days	twi_ss.20959.1|g.27071	5.37	4.73	26.66	0.0004	0.0133
7 days	twi_ss.443.1|g.815	1.46	5.40	26.66	0.0004	0.0133
7 days	twi_ss.16049.1|g.20645	3.42	3.95	26.59	0.0004	0.0134
7 days	twi_ss.2383.1|g.4297	-4.23	4.09	26.59	0.0004	0.0134
7 days	twi_ss.14757.1|g.19189	1.71	6.12	26.57	0.0004	0.0134
7 days	twi_ss.9963.1|g.13288	1.84	6.05	26.57	0.0004	0.0134
7 days	twi_ss.30194.1|g.40782	-2.35	5.19	26.52	0.0004	0.0135
7 days	twi_ss.15380.1|g.19920	-1.85	5.36	26.50	0.0004	0.0135
7 days	twi_ss.31253.1|g.42479	1.24	5.30	26.50	0.0004	0.0135
7 days	twi_ss.10507.1|g.14141	0.81	6.10	26.41	0.0004	0.0136
7 days	twi_ss.10507.2|g.14143	0.81	6.10	26.41	0.0004	0.0136
7 days	twi_ss.10507.3|g.14140	0.81	6.10	26.41	0.0004	0.0136
7 days	twi_ss.10507.4|g.14139	0.81	6.10	26.41	0.0004	0.0136
7 days	twi_ss.10507.5|g.14142	0.81	6.10	26.41	0.0004	0.0136
7 days	twi_ss.16407.1|g.20962	1.32	9.11	26.45	0.0004	0.0136
7 days	twi_ss.13574.1|g.17954	-5.17	3.49	26.35	0.0004	0.0137
7 days	twi_ss.13944a.4|g.18232	1.10	8.29	26.32	0.0004	0.0137
7 days	twi_ss.18468.1|g.23509	-0.85	6.98	26.30	0.0004	0.0137
7 days	twi_ss.21215.1|g.27452	1.37	6.07	26.31	0.0004	0.0137
7 days	twi_ss.24183.1|g.31560	-5.20	5.27	26.32	0.0004	0.0137
7 days	twi_ss.4756.2|g.7341	1.90	5.15	26.30	0.0004	0.0137
7 days	twi_ss.4918.1|g.7509	7.30	3.54	26.29	0.0004	0.0137
7 days	twi_ss.6610.1|g.9638	2.46	5.00	26.33	0.0004	0.0137

7 days	twi_ss.7326.1|g.10332	1.28	7.18	26.30	0.0004	0.0137
7 days	twi_ss.923.1|g.1720	-2.65	6.81	26.31	0.0004	0.0137
7 days	twi_ss.30156.5|g.40644	0.83	7.34	26.26	0.0004	0.0137
7 days	twi_ss.10089.1|g.13504	-1.47	5.85	26.24	0.0004	0.0137
7 days	twi_ss.21610.5|g.28012	2.93	6.22	26.20	0.0004	0.0138
7 days	twi_ss.30551.1|g.41380	4.53	5.98	26.17	0.0004	0.0138
7 days	twi_ss.4163.1|g.6586	-1.93	4.87	26.15	0.0004	0.0139
7 days	twi_ss.12665.1|g.16747	-2.05	4.68	26.12	0.0004	0.0139
7 days	twi_ss.2639.1|g.4771	1.08	5.68	26.13	0.0004	0.0139
7 days	twi_ss.24352c.3|g.31772	8.00	4.34	45.05	0.0004	0.0139
7 days	twi_ss.1584.2|g.2844	1.06	6.16	26.07	0.0004	0.0140
7 days	twi_ss.9992.1|g.13302	-1.11	5.45	26.06	0.0004	0.0140
7 days	twi_ss.28413.1|g.37989	-2.35	4.52	25.98	0.0004	0.0141
7 days	twi_ss.27729.1|g.36923	2.55	5.81	25.94	0.0004	0.0142
7 days	twi_ss.5411.1|g.8165	0.96	6.88	25.94	0.0004	0.0142
7 days	twi_ss.6728.1|g.9797	-2.16	4.37	25.93	0.0004	0.0142
7 days	twi_ss.27407.1|g.36535	-3.07	3.87	25.89	0.0004	0.0142
7 days	twi_ss.4014.5|g.6429	-1.73	8.86	25.90	0.0004	0.0142
7 days	twi_ss.15151.1|g.19718	-3.98	3.50	25.86	0.0004	0.0143
7 days	twi_ss.2587.1|g.4722	1.57	5.80	25.85	0.0004	0.0143
7 days	twi_ss.27504.2|g.36604	1.10	6.24	25.85	0.0004	0.0143
7 days	twi_ss.4602.1|g.7125	-2.27	4.30	25.87	0.0004	0.0143
7 days	twi_ss.7685.1|g.10766	-2.33	4.01	25.83	0.0004	0.0143
7 days	twi_ss.12872.3|g.17048	2.74	6.27	25.80	0.0004	0.0143
7 days	twi_ss.29504.1|g.39730	1.68	5.26	25.80	0.0004	0.0143
7 days	twi_ss.4304a.1|g.6726	2.35	5.36	25.80	0.0004	0.0143
7 days	twi_ss.15261.1|g.19837	-3.35	4.21	25.75	0.0004	0.0144
7 days	twi_ss.20445.1|g.26342	0.90	6.64	25.75	0.0004	0.0144
7 days	twi_ss.23511.1|g.30171	1.85	4.90	25.75	0.0004	0.0144
7 days	twi_ss.28592.1|g.38300	2.32	4.04	25.74	0.0004	0.0144
7 days	twi_ss.1156.2|g.2061	-1.51	5.84	25.70	0.0004	0.0144
7 days	twi_ss.14178.1|g.18455	-1.45	8.45	25.71	0.0004	0.0144
7 days	twi_ss.6265.1|g.9248	-0.91	6.20	25.70	0.0004	0.0144

7 days	twi_ss.12377.1|g.16507	2.10	5.24	25.67	0.0004	0.0144
7 days	twi_ss.8770.1|g.11923	-1.85	7.09	25.65	0.0004	0.0145
7 days	twi_ss.11446.1|g.15098	1.66	4.91	25.63	0.0004	0.0145
7 days	twi_ss.30355.1|g.41052	-2.00	5.61	25.61	0.0004	0.0145
7 days	twi_ss.25773.1|g.33565	0.85	9.26	25.60	0.0004	0.0145
7 days	twi_ss.574.2|g.1054	1.77	7.11	25.59	0.0004	0.0145
7 days	twi_ss.30001b.1|g.40489	1.54	5.32	25.57	0.0004	0.0146
7 days	twi_ss.9407.1|g.12694	-1.62	4.42	25.56	0.0004	0.0146
7 days	twi_ss.14580.1|g.19046	1.39	5.46	25.47	0.0004	0.0147
7 days	twi_ss.25028.1|g.32853	-4.27	4.20	25.47	0.0004	0.0147
7 days	twi_ss.7858.12|g.10915	4.33	6.25	25.44	0.0004	0.0148
7 days	twi_ss.7777.1|g.10852	3.46	4.97	25.43	0.0004	0.0148
7 days	twi_ss.2662.1|g.4821	1.01	6.95	25.35	0.0005	0.0150
7 days	twi_ss.29442.1|g.39647	-1.96	5.56	25.32	0.0005	0.0150
7 days	twi_ss.9412.1|g.12748	-4.37	4.23	25.31	0.0005	0.0150
7 days	twi_ss.24188.1|g.31533	-4.51	3.67	25.30	0.0005	0.0150
7 days	twi_ss.4477.1|g.6994	4.83	5.40	25.28	0.0005	0.0150
7 days	twi_ss.26821.1|g.35546	1.68	5.91	25.25	0.0005	0.0151
7 days	twi_ss.27336a.3|g.36383	4.36	4.68	25.21	0.0005	0.0152
7 days	twi_ss.16983.1|g.21687	-1.16	6.15	25.16	0.0005	0.0153
7 days	twi_ss.20516a.5|g.26426	6.77	3.34	30.60	0.0005	0.0153
7 days	twi_ss.2291.1|g.4028	-1.78	6.45	25.15	0.0005	0.0153
7 days	twi_ss.4691.1|g.7242	1.05	8.31	25.13	0.0005	0.0153
7 days	twi_ss.14648.1|g.19094	-1.86	5.24	25.09	0.0005	0.0153
7 days	twi_ss.22647.1|g.29224	3.84	4.20	25.09	0.0005	0.0153
7 days	twi_ss.661.1|g.1181	-1.02	5.95	25.08	0.0005	0.0154
7 days	twi_ss.28564.1|g.38234	1.79	8.40	25.07	0.0005	0.0154
7 days	twi_ss.17959.1|g.22843	-2.14	5.07	25.05	0.0005	0.0154
7 days	twi_ss.18321.1|g.23341	-3.75	4.48	25.04	0.0005	0.0154
7 days	twi_ss.29406a.1|g.39525	2.23	5.48	25.04	0.0005	0.0154
7 days	twi_ss.31853a.1|g.43364	5.66	3.97	25.05	0.0005	0.0154
7 days	twi_ss.1880.1|g.3316	-4.85	3.93	24.98	0.0005	0.0155
7 days	twi_ss.21288a.1|g.27523	2.34	3.84	24.95	0.0005	0.0156

7 days	twi_ss.10323.1|g.13907	1.18	6.39	24.92	0.0005	0.0156
7 days	twi_ss.20992.1|g.27093	-4.45	3.01	24.92	0.0005	0.0156
7 days	twi_ss.5304a.1|g.8061	-2.25	7.92	24.93	0.0005	0.0156
7 days	twi_ss.10276.1|g.13757	1.18	6.35	24.87	0.0005	0.0156
7 days	twi_ss.1217.1|g.2100	-7.14	3.36	30.19	0.0005	0.0156
7 days	twi_ss.12842.2|g.16927	-1.30	6.46	24.86	0.0005	0.0156
7 days	twi_ss.15847.1|g.20403	-2.34	5.71	24.85	0.0005	0.0156
7 days	twi_ss.23723.1|g.30643	1.86	6.10	24.89	0.0005	0.0156
7 days	twi_ss.25813.1|g.34079	2.34	4.67	24.86	0.0005	0.0156
7 days	twi_ss.29027.1|g.38937	1.64	5.34	24.87	0.0005	0.0156
7 days	twi_ss.7208.1|g.10259	1.74	5.49	24.86	0.0005	0.0156
7 days	twi_ss.13154.1|g.17467	1.03	5.31	24.83	0.0005	0.0156
7 days	twi_ss.29211.1|g.39148	2.17	7.87	24.83	0.0005	0.0156
7 days	twi_ss.2715.1|g.4854	3.22	4.83	24.82	0.0005	0.0156
7 days	twi_ss.28804.1|g.38536	1.82	5.54	24.64	0.0005	0.0160
7 days	twi_ss.30137.1|g.40684	2.05	4.89	24.65	0.0005	0.0160
7 days	twi_ss.21625.1|g.28032	1.46	6.44	24.59	0.0005	0.0161
7 days	twi_ss.2267.1|g.4011	2.75	3.68	24.58	0.0005	0.0162
7 days	twi_ss.4535.1|g.7042	0.73	8.35	24.56	0.0005	0.0162
7 days	twi_ss.12733.1|g.16821	2.31	4.41	24.54	0.0005	0.0162
7 days	twi_ss.28144.1|g.37496	-1.37	5.42	24.51	0.0005	0.0163
7 days	twi_ss.5045.1|g.7648	-3.65	4.81	24.50	0.0005	0.0163
7 days	twi_ss.24052.1|g.31321	3.81	3.46	24.48	0.0005	0.0163
7 days	twi_ss.24052.2|g.31320	3.81	3.46	24.48	0.0005	0.0163
7 days	twi_ss.3473.1|g.5859	-2.57	4.12	24.49	0.0005	0.0163
7 days	twi_ss.2273.1|g.4015	-1.66	5.60	24.47	0.0005	0.0163
7 days	twi_ss.29136.1|g.39098	-1.61	5.13	24.46	0.0005	0.0163
7 days	twi_ss.1830.1|g.3223	1.16	6.11	24.43	0.0005	0.0164
7 days	twi_ss.19831.1|g.25472	-1.64	6.83	24.39	0.0005	0.0164
7 days	twi_ss.19831.8|g.25466	-1.64	6.83	24.39	0.0005	0.0164
7 days	twi_ss.6931.1|g.9997	-2.01	5.58	24.40	0.0005	0.0164
7 days	twi_ss.7010.1|g.10061	-1.64	5.61	24.38	0.0005	0.0164
7 days	twi_ss.30540.1|g.41328	0.50	9.47	24.37	0.0005	0.0164

7 days	twi_ss.10196.1|g.13703	0.77	7.16	24.34	0.0005	0.0165
7 days	twi_ss.28002.1|g.37275	1.56	6.21	24.34	0.0005	0.0165
7 days	twi_ss.1818.1|g.3203	-6.89	3.34	40.85	0.0005	0.0166
7 days	twi_ss.21114a.2|g.27303	-2.09	5.50	24.23	0.0005	0.0167
7 days	twi_ss.24378.1|g.31976	-1.49	6.72	24.21	0.0005	0.0167
7 days	twi_ss.27290.1|g.36273	-2.22	4.87	24.21	0.0005	0.0167
7 days	twi_ss.27575.1|g.36770	1.70	4.53	24.14	0.0005	0.0168
7 days	twi_ss.4335.1|g.6842	3.10	6.39	24.15	0.0005	0.0168
7 days	twi_ss.7242.1|g.10281	-1.93	5.41	24.15	0.0005	0.0168
7 days	twi_ss.20484.1|g.26433	0.92	6.43	24.14	0.0005	0.0168
7 days	twi_ss.27170a.3|g.36112	-1.79	5.32	24.10	0.0005	0.0169
7 days	twi_ss.6621.1|g.9671	-1.73	5.37	24.03	0.0006	0.0171
7 days	twi_ss.25342.1|g.33330	-2.06	4.76	23.94	0.0006	0.0173
7 days	twi_ss.19219.2|g.24607	-7.20	5.27	23.92	0.0006	0.0174
7 days	twi_ss.32065.2|g.43721	4.12	6.06	23.83	0.0006	0.0176
7 days	twi_ss.12230.1|g.16255	1.58	6.91	23.80	0.0006	0.0177
7 days	twi_ss.5433b.1|g.8210	1.23	6.33	23.79	0.0006	0.0177
7 days	twi_ss.25806.1|g.34080	-3.49	4.73	23.72	0.0006	0.0178
7 days	twi_ss.27971.1|g.37244	1.27	6.73	23.72	0.0006	0.0178
7 days	twi_ss.28408.1|g.37982	1.71	4.89	23.73	0.0006	0.0178
7 days	twi_ss.6778a.3|g.9735	1.45	4.99	23.73	0.0006	0.0178
7 days	twi_ss.23638.1|g.30408	1.54	4.99	23.71	0.0006	0.0178
7 days	twi_ss.12332.9|g.16421	8.22	5.36	23.62	0.0006	0.0180
7 days	twi_ss.4535.2|g.7041	0.69	7.87	23.62	0.0006	0.0180
7 days	twi_ss.28345a.1|g.37870	-4.31	3.62	23.60	0.0006	0.0180
7 days	twi_ss.4279.1|g.6783	1.63	6.30	23.60	0.0006	0.0180
7 days	twi_ss.5108.1|g.7749	-5.82	2.65	39.28	0.0006	0.0181
7 days	twi_ss.3881.1|g.6304	1.16	5.65	23.56	0.0006	0.0181
7 days	twi_ss.29675.1|g.39998	-2.27	6.80	23.54	0.0006	0.0182
7 days	twi_ss.10747a.2|g.14392	-1.98	5.71	23.53	0.0006	0.0182
7 days	twi_ss.24497.5|g.32090	5.65	3.34	23.50	0.0006	0.0182
7 days	twi_ss.4916.1|g.7514	1.86	4.33	23.49	0.0006	0.0183
7 days	twi_ss.23081.1|g.29724	-0.71	6.57	23.46	0.0006	0.0183

7 days	twi_ss.26090a.1|g.34602	-4.74	5.73	23.47	0.0006	0.0183
7 days	twi_ss.5500.1|g.8279	-3.21	3.96	23.46	0.0006	0.0183
7 days	twi_ss.30429.1|g.41205	-2.21	6.15	23.44	0.0006	0.0183
7 days	twi_ss.27071a.1|g.36012	1.65	6.10	23.43	0.0006	0.0183
7 days	twi_ss.4660.1|g.7190	0.89	7.39	23.40	0.0006	0.0184
7 days	twi_ss.2348.1|g.4257	-3.69	4.12	23.36	0.0006	0.0185
7 days	twi_ss.6781.1|g.9766	1.28	6.59	23.34	0.0006	0.0185
7 days	twi_ss.2815.1|g.5084	-1.19	6.08	23.30	0.0006	0.0186
7 days	twi_ss.28186a.1|g.37576	-4.84	5.36	23.30	0.0006	0.0186
7 days	twi_ss.13791.1|g.18111	1.83	6.00	23.29	0.0006	0.0186
7 days	twi_ss.19415.1|g.24943	3.93	4.02	23.26	0.0006	0.0187
7 days	twi_ss.23153.1|g.29790	3.97	5.77	23.26	0.0006	0.0187
7 days	twi_ss.20151.1|g.25922	-4.26	3.80	23.23	0.0006	0.0187
7 days	twi_ss.2619.7|g.4737	3.59	5.73	23.23	0.0006	0.0187
7 days	twi_ss.8819.2|g.12010	-6.24	2.83	23.20	0.0006	0.0188
7 days	twi_ss.30134.5|g.40699	1.46	8.79	23.18	0.0006	0.0188
7 days	twi_ss.18212.3|g.23144	6.43	3.09	23.15	0.0006	0.0189
7 days	twi_ss.11878.1|g.15764	-2.53	3.99	23.13	0.0006	0.0190
7 days	twi_ss.24043.1|g.31303	-2.84	4.50	23.06	0.0006	0.0192
7 days	twi_ss.20172.1|g.25984	1.46	5.59	23.04	0.0006	0.0192
7 days	twi_ss.11082.1|g.14675	-1.59	4.86	23.01	0.0006	0.0192
7 days	twi_ss.5399.1|g.8148	-1.64	4.06	23.02	0.0006	0.0192
7 days	twi_ss.1543.4|g.2786	7.11	3.88	22.99	0.0007	0.0193
7 days	twi_ss.1403.2|g.2525	7.46	4.86	22.99	0.0007	0.0193
7 days	twi_ss.30222b.6|g.40808	-2.27	5.47	22.98	0.0007	0.0193
7 days	twi_ss.11555.4|g.15340	-3.26	3.71	22.97	0.0007	0.0193
7 days	twi_ss.17531b.1|g.22398	1.63	7.62	22.96	0.0007	0.0193
7 days	twi_ss.30689.2|g.41599	-1.29	7.89	22.96	0.0007	0.0193
7 days	twi_ss.27000.1|g.35874	1.88	6.94	22.91	0.0007	0.0195
7 days	twi_ss.13937.1|g.18267	-3.09	3.87	22.89	0.0007	0.0195
7 days	twi_ss.21564.1|g.27969	1.06	6.71	22.89	0.0007	0.0195
7 days	twi_ss.4368.1|g.6882	-4.02	3.86	27.49	0.0007	0.0195
7 days	twi_ss.16229.2|g.20811	1.06	6.55	22.85	0.0007	0.0196

7 days	twi_ss.20345.2|g.26180	-3.41	4.93	22.84	0.0007	0.0196
7 days	twi_ss.4825.1|g.7403	-1.83	4.72	22.82	0.0007	0.0196
7 days	twi_ss.27066.1|g.35987	2.03	6.45	22.79	0.0007	0.0197
7 days	twi_ss.1343.1|g.2422	0.44	8.24	22.74	0.0007	0.0198
7 days	twi_ss.25295.1|g.33312	-4.62	2.44	27.13	0.0007	0.0198
7 days	twi_ss.6192.1|g.9196	-2.40	9.99	22.75	0.0007	0.0198
7 days	twi_ss.4284.1|g.6781	-1.38	5.39	22.68	0.0007	0.0200
7 days	twi_ss.31013.1|g.42205	-3.36	4.95	22.66	0.0007	0.0200
7 days	twi_ss.25703.4|g.33870	-2.85	4.92	22.63	0.0007	0.0201
7 days	twi_ss.30590.1|g.41391	-1.07	5.45	22.62	0.0007	0.0201
7 days	twi_ss.3922b.2|g.6356	1.97	4.94	22.61	0.0007	0.0201
7 days	twi_ss.5201.2|g.7830	-1.41	6.34	22.61	0.0007	0.0201
7 days	twi_ss.33.1|g.70	1.34	6.06	22.58	0.0007	0.0202
7 days	twi_ss.17473.4|g.22296	5.53	2.25	27.04	0.0007	0.0202
7 days	twi_ss.18006.1|g.22906	1.47	4.73	22.53	0.0007	0.0202
7 days	twi_ss.18250.1|g.23153	2.55	7.02	22.52	0.0007	0.0202
7 days	twi_ss.22439.1|g.29005	-8.05	4.24	22.55	0.0007	0.0202
7 days	twi_ss.24730.1|g.32444	1.78	6.34	22.53	0.0007	0.0202
7 days	twi_ss.4477.2|g.6993	4.79	3.83	27.07	0.0007	0.0202
7 days	twi_ss.4977.8|g.7536	-2.58	5.84	22.53	0.0007	0.0202
7 days	twi_ss.9501.1|g.12770	1.98	4.76	22.55	0.0007	0.0202
7 days	twi_ss.161.1|g.272	-3.83	4.56	22.52	0.0007	0.0202
7 days	twi_ss.10467.1|g.14103	1.09	5.97	22.49	0.0007	0.0203
7 days	twi_ss.7624.1|g.10679	0.88	7.52	22.48	0.0007	0.0203
7 days	twi_ss.13146.1|g.17471	2.57	4.88	22.46	0.0007	0.0203
7 days	twi_ss.26801.1|g.35584	-0.88	6.35	22.44	0.0007	0.0204
7 days	twi_ss.21051.1|g.27249	3.76	3.47	22.42	0.0007	0.0204
7 days	twi_ss.25032.1|g.32863	-4.91	3.23	22.43	0.0007	0.0204
7 days	twi_ss.32017.1|g.43662	-5.61	3.74	26.89	0.0007	0.0204
7 days	twi_ss.30543.1|g.41378	-1.84	4.85	22.39	0.0007	0.0205
7 days	twi_ss.6820.2|g.9882	1.49	5.46	22.40	0.0007	0.0205
7 days	twi_ss.20312.1|g.26146	0.94	5.49	22.37	0.0007	0.0205
7 days	twi_ss.26855.1|g.35618	-2.64	4.45	22.31	0.0007	0.0206

7 days	twi_ss.2798.1|g.5037	1.17	5.03	22.31	0.0007	0.0206
7 days	twi_ss.2798.2|g.5032	1.17	5.03	22.31	0.0007	0.0206
7 days	twi_ss.4746.1|g.7318	1.04	8.54	22.31	0.0007	0.0206
7 days	twi_ss.26468.1|g.35099	0.99	5.46	22.30	0.0007	0.0206
7 days	twi_ss.21657.1|g.28088	1.99	5.80	22.28	0.0007	0.0207
7 days	twi_ss.27827b.1|g.37022	-1.58	6.12	22.27	0.0007	0.0207
7 days	twi_ss.31286.2|g.42540	-8.67	4.66	22.27	0.0007	0.0207
7 days	twi_ss.5521.1|g.8291	0.69	8.48	22.26	0.0007	0.0207
7 days	twi_ss.22572.1|g.29155	-2.96	5.01	22.25	0.0007	0.0207
7 days	twi_ss.10077.1|g.13429	-0.76	7.11	22.22	0.0007	0.0208
7 days	twi_ss.31879.2|g.43447	1.54	7.02	22.17	0.0007	0.0209
7 days	twi_ss.24154.1|g.31475	0.85	6.63	22.17	0.0007	0.0210
7 days	twi_ss.1144.1|g.2031	-4.81	2.78	26.45	0.0008	0.0212
7 days	twi_ss.9065.1|g.12260	1.15	6.61	22.10	0.0008	0.0212
7 days	twi_ss.20287b.1|g.26122	0.69	7.52	22.09	0.0008	0.0212
7 days	twi_ss.2655.1|g.4837	1.41	5.39	22.07	0.0008	0.0212
7 days	twi_ss.31934.1|g.43538	-0.92	8.79	22.07	0.0008	0.0212
7 days	twi_ss.24983.1|g.32837	1.46	5.13	22.04	0.0008	0.0213
7 days	twi_ss.22388.1|g.28909	-6.69	3.13	35.89	0.0008	0.0213
7 days	twi_ss.26710.2|g.35422	-7.24	3.57	22.02	0.0008	0.0213
7 days	twi_ss.28292.9|g.37672	-4.06	4.11	22.02	0.0008	0.0213
7 days	twi_ss.18575.1|g.23655	-2.50	5.19	22.01	0.0008	0.0213
7 days	twi_ss.29749.1|g.40117	0.95	6.32	22.00	0.0008	0.0213
7 days	twi_ss.7889.1|g.10952	-4.79	3.35	21.99	0.0008	0.0213
7 days	twi_ss.28510a.22|g.38076	4.49	1.91	35.77	0.0008	0.0214
7 days	twi_ss.4076.2|g.6495	6.21	2.64	21.95	0.0008	0.0214
7 days	twi_ss.8805.1|g.11998	-1.92	5.31	21.95	0.0008	0.0214
7 days	twi_ss.12011.1|g.15932	1.04	6.13	21.93	0.0008	0.0215
7 days	twi_ss.2836.1|g.5119	1.43	5.20	21.92	0.0008	0.0215
7 days	twi_ss.110a.2|g.198	4.33	5.24	21.87	0.0008	0.0216
7 days	twi_ss.17829.1|g.22743	1.93	5.13	21.87	0.0008	0.0216
7 days	twi_ss.29296.1|g.39294	0.77	9.97	21.87	0.0008	0.0216
7 days	twi_ss.8473.1|g.11545	-1.25	5.43	21.88	0.0008	0.0216

7 days	twi_ss.23734.4|g.30617	-0.70	7.33	21.86	0.0008	0.0216
7 days	twi_ss.31915.5|g.43431	0.89	8.46	21.84	0.0008	0.0217
7 days	twi_ss.17490.1|g.22321	-2.00	4.37	21.83	0.0008	0.0217
7 days	twi_ss.31893.1|g.43466	-3.01	4.83	21.83	0.0008	0.0217
7 days	twi_ss.24976.2|g.32815	6.77	2.95	21.82	0.0008	0.0217
7 days	twi_ss.4337.1|g.6840	0.90	5.94	21.81	0.0008	0.0217
7 days	twi_ss.26097.1|g.34566	0.81	8.20	21.76	0.0008	0.0219
7 days	twi_ss.2549.1|g.4559	0.75	8.20	21.75	0.0008	0.0219
7 days	twi_ss.20209.2|g.26002	7.30	3.47	21.74	0.0008	0.0219
7 days	twi_ss.21425.2|g.27686	4.01	4.31	21.73	0.0008	0.0219
7 days	twi_ss.24815.1|g.32609	1.56	5.55	21.71	0.0008	0.0220
7 days	twi_ss.24470.1|g.32125	1.39	5.81	21.69	0.0008	0.0220
7 days	twi_ss.25939.1|g.34234	-4.68	3.64	21.68	0.0008	0.0220
7 days	twi_ss.25212.1|g.33076	-1.14	6.13	21.66	0.0008	0.0221
7 days	twi_ss.18795.3|g.24038	3.35	8.19	21.64	0.0008	0.0222
7 days	twi_ss.28940.1|g.38796	-5.04	3.43	25.82	0.0008	0.0222
7 days	twi_ss.7151.1|g.10200	-6.33	2.94	35.03	0.0008	0.0222
7 days	twi_ss.31815.1|g.43335	-1.40	4.84	21.61	0.0008	0.0222
7 days	twi_ss.18451.1|g.23500	0.98	6.31	21.59	0.0008	0.0222
7 days	twi_ss.22950.1|g.29594	-2.50	6.51	21.56	0.0008	0.0223
7 days	twi_ss.25263.1|g.33243	-2.48	3.45	21.56	0.0008	0.0223
7 days	twi_ss.26694b.3|g.35409	1.26	4.37	22.39	0.0008	0.0223
7 days	twi_ss.7443.1|g.10451	2.04	4.01	21.55	0.0008	0.0223
7 days	twi_ss.14522.1|g.18995	-2.30	5.24	21.53	0.0008	0.0224
7 days	twi_ss.23649.1|g.30466	2.10	5.58	21.52	0.0008	0.0224
7 days	twi_ss.31218.1|g.42418	1.53	5.58	21.47	0.0008	0.0226
7 days	twi_ss.5526a.2|g.8328	3.24	4.67	21.46	0.0008	0.0226
7 days	twi_ss.5357.1|g.8107	1.02	6.95	21.43	0.0008	0.0227
7 days	twi_ss.26938.1|g.35766	-4.53	3.65	21.41	0.0008	0.0227
7 days	twi_ss.16171.1|g.20756	1.71	6.65	21.38	0.0009	0.0228
7 days	twi_ss.2917.1|g.5212	-1.48	5.84	21.37	0.0009	0.0228
7 days	twi_ss.21292.1|g.27529	-2.36	5.83	21.35	0.0009	0.0229
7 days	twi_ss.23113.2|g.29758	-1.41	6.76	21.35	0.0009	0.0229

7 days	twi_ss.14339b.2|g.18702	-6.96	3.29	34.41	0.0009	0.0230
7 days	twi_ss.25120.1|g.32969	1.13	6.53	21.31	0.0009	0.0230
7 days	twi_ss.476.1|g.879	1.38	4.84	21.31	0.0009	0.0230
7 days	twi_ss.9337.1|g.12618	1.49	4.84	21.25	0.0009	0.0232
7 days	twi_ss.18704.1|g.23877	0.45	9.08	21.23	0.0009	0.0232
7 days	twi_ss.2601.1|g.4625	0.86	6.38	21.23	0.0009	0.0232
7 days	twi_ss.27025.1|g.35925	-0.87	5.99	21.22	0.0009	0.0232
7 days	twi_ss.4826.1|g.7400	0.92	6.68	21.22	0.0009	0.0232
7 days	twi_ss.117.1|g.209	0.66	6.91	21.20	0.0009	0.0233
7 days	twi_ss.17570.1|g.22349	-0.75	5.95	21.20	0.0009	0.0233
7 days	twi_ss.10437.2|g.14071	1.43	5.77	21.17	0.0009	0.0233
7 days	twi_ss.15060.1|g.19559	0.83	6.84	21.18	0.0009	0.0233
7 days	twi_ss.22195.1|g.28756	1.59	5.17	21.18	0.0009	0.0233
7 days	twi_ss.2301.1|g.4043	1.24	6.49	21.17	0.0009	0.0233
7 days	twi_ss.12129.2|g.16018	5.23	2.17	21.15	0.0009	0.0234
7 days	twi_ss.29994.1|g.40475	1.72	7.77	21.13	0.0009	0.0234
7 days	twi_ss.16506.3|g.20586	-1.97	9.24	21.11	0.0009	0.0235
7 days	twi_ss.20341.1|g.26189	-3.03	4.63	21.11	0.0009	0.0235
7 days	twi_ss.5603.1|g.8310	-4.17	3.80	21.10	0.0009	0.0235
7 days	twi_ss.12458.1|g.16622	6.77	5.22	21.09	0.0009	0.0235
7 days	twi_ss.30705.1|g.41592	-2.04	4.80	21.09	0.0009	0.0235
7 days	twi_ss.23400.1|g.30059	1.19	5.30	21.08	0.0009	0.0235
7 days	twi_ss.32111.1|g.43773	0.68	6.54	21.07	0.0009	0.0235
7 days	twi_ss.8321.1|g.11424	0.80	6.79	21.07	0.0009	0.0235
7 days	twi_ss.16652.3|g.21312	1.54	6.61	21.05	0.0009	0.0236
7 days	twi_ss.28686.1|g.38419	-2.02	4.71	21.05	0.0009	0.0236
7 days	twi_ss.4992.1|g.7612	1.87	4.84	21.04	0.0009	0.0236
7 days	twi_ss.26708.1|g.35428	1.58	6.20	21.02	0.0009	0.0236
7 days	twi_ss.20396.1|g.26276	0.97	5.90	20.99	0.0009	0.0237
7 days	twi_ss.31675.1|g.43127	-2.96	4.52	20.99	0.0009	0.0237
7 days	twi_ss.13607.1|g.17960	-0.78	6.49	20.96	0.0009	0.0237
7 days	twi_ss.18476.1|g.23508	-5.53	3.68	20.97	0.0009	0.0237
7 days	twi_ss.25504.1|g.33563	-2.92	5.57	20.98	0.0009	0.0237

7 days	twi_ss.28866.1|g.38753	3.24	4.51	20.96	0.0009	0.0237
7 days	twi_ss.4547.1|g.7064	0.92	6.64	20.97	0.0009	0.0237
7 days	twi_ss.6765.1|g.9843	-6.30	3.18	24.94	0.0009	0.0237
7 days	twi_ss.17482.1|g.22316	1.21	6.76	20.94	0.0009	0.0237
7 days	twi_ss.2115.1|g.3816	-3.95	4.98	20.94	0.0009	0.0237
7 days	twi_ss.26715.1|g.35440	-6.50	3.66	20.95	0.0009	0.0237
7 days	twi_ss.28187.4|g.37572	-5.91	5.11	20.94	0.0009	0.0237
7 days	twi_ss.11017.1|g.14636	1.11	5.30	20.91	0.0009	0.0238
7 days	twi_ss.26301.1|g.34878	0.71	7.18	20.91	0.0009	0.0238
7 days	twi_ss.20132.2|g.25898	-1.81	5.27	20.90	0.0009	0.0238
7 days	twi_ss.17946.1|g.22825	-2.26	4.21	20.88	0.0009	0.0239
7 days	twi_ss.31358.1|g.42626	1.41	5.99	20.88	0.0009	0.0239
7 days	twi_ss.9015.1|g.12194	0.62	8.42	20.88	0.0009	0.0239
7 days	twi_ss.18687.1|g.23851	3.38	3.90	20.86	0.0009	0.0239
7 days	twi_ss.5738.2|g.8559	2.22	7.96	20.85	0.0009	0.0239
7 days	twi_ss.7795.1|g.10876	1.56	4.89	20.85	0.0009	0.0239
7 days	twi_ss.15005a.1|g.19501	1.91	5.67	20.84	0.0009	0.0239
7 days	twi_ss.1269.2|g.2279	-3.06	4.46	20.82	0.0009	0.0240
7 days	twi_ss.16497.1|g.21089	-2.26	4.01	20.82	0.0009	0.0240
7 days	twi_ss.9201.1|g.12410	-1.27	6.34	20.77	0.0009	0.0241
7 days	twi_ss.26050.1|g.34513	-1.09	4.82	20.73	0.0010	0.0243
7 days	twi_ss.5653.1|g.8500	0.98	5.87	20.72	0.0010	0.0243
7 days	twi_ss.16708.1|g.21389	-2.34	4.39	20.71	0.0010	0.0244
7 days	twi_ss.28007.1|g.37283	1.99	8.60	20.70	0.0010	0.0244
7 days	twi_ss.22177.1|g.28693	-2.09	4.14	20.69	0.0010	0.0244
7 days	twi_ss.6762.1|g.9844	-2.60	3.91	20.68	0.0010	0.0244
7 days	twi_ss.15820.1|g.20389	0.63	8.51	20.66	0.0010	0.0245
7 days	twi_ss.17095.1|g.21886	-3.15	4.67	20.66	0.0010	0.0245
7 days	twi_ss.26709.1|g.35442	0.76	6.12	20.66	0.0010	0.0245
7 days	twi_ss.10747b.1|g.14386	-0.75	8.96	20.65	0.0010	0.0245
7 days	twi_ss.10128a.1|g.13547	1.11	8.36	20.63	0.0010	0.0245
7 days	twi_ss.15596.1|g.20167	0.74	6.38	20.62	0.0010	0.0245
7 days	twi_ss.22989.1|g.29643	0.96	5.58	20.62	0.0010	0.0245

7 days	twi_ss.27562.1|g.36720	-2.40	6.47	20.60	0.0010	0.0246
7 days	twi_ss.18100.4|g.22999	5.01	3.58	24.45	0.0010	0.0246
7 days	twi_ss.8576.1|g.11730	1.81	4.75	20.58	0.0010	0.0246
7 days	twi_ss.18535.1|g.23608	-1.11	7.40	20.57	0.0010	0.0247
7 days	twi_ss.11333.1|g.14956	0.96	6.70	20.55	0.0010	0.0247
7 days	twi_ss.16186.2|g.20767	-4.60	3.08	20.55	0.0010	0.0247
7 days	twi_ss.19499.1|g.25032	1.13	5.25	20.51	0.0010	0.0247
7 days	twi_ss.19617b.2|g.25195	1.51	5.13	20.52	0.0010	0.0247
7 days	twi_ss.2734.1|g.4902	1.30	5.41	20.53	0.0010	0.0247
7 days	twi_ss.29566.2|g.39762	2.55	6.66	20.52	0.0010	0.0247
7 days	twi_ss.30095.1|g.40635	1.12	6.19	20.51	0.0010	0.0247
7 days	twi_ss.3845.1|g.6251	0.68	6.50	20.53	0.0010	0.0247
7 days	twi_ss.6244a.2|g.9240	-2.80	4.39	20.51	0.0010	0.0247
7 days	twi_ss.827.1|g.1458	1.00	6.07	20.52	0.0010	0.0247
7 days	twi_ss.27195.1|g.36148	-3.45	3.67	20.51	0.0010	0.0247
7 days	twi_ss.30983b.3|g.41861	-4.99	4.02	20.48	0.0010	0.0248
7 days	twi_ss.5474.4|g.8258	0.86	8.15	20.47	0.0010	0.0248
7 days	twi_ss.21299.1|g.27525	-2.20	3.92	20.46	0.0010	0.0248
7 days	twi_ss.28506.9|g.38184	-5.79	2.65	32.55	0.0010	0.0249
7 days	twi_ss.7359.1|g.10368	-2.17	5.18	20.44	0.0010	0.0249
7 days	twi_ss.28950.1|g.38870	1.71	5.28	20.43	0.0010	0.0249
7 days	twi_ss.28950.2|g.38871	1.71	5.28	20.43	0.0010	0.0249
7 days	twi_ss.1115.1|g.1955	1.28	8.53	20.42	0.0010	0.0249
7 days	twi_ss.16506.2|g.20591	-1.76	8.21	20.41	0.0010	0.0250
7 days	twi_ss.1636.1|g.2868	-3.30	3.74	20.38	0.0010	0.0250
7 days	twi_ss.30966.1|g.42009	-2.77	5.45	20.38	0.0010	0.0250
7 days	twi_ss.6031.1|g.8907	-1.23	7.22	20.38	0.0010	0.0250
7 days	twi_ss.6830.1|g.9893	1.04	6.09	20.39	0.0010	0.0250
7 days	twi_ss.25893.1|g.34159	-1.42	5.79	20.36	0.0010	0.0250
7 days	twi_ss.29906.1|g.40322	1.92	7.55	20.36	0.0010	0.0250
7 days	twi_ss.863.1|g.1528	1.06	5.38	20.35	0.0010	0.0251
7 days	twi_ss.22925.1|g.29592	-3.57	3.64	20.33	0.0010	0.0251
7 days	twi_ss.7349.1|g.10353	-2.63	6.25	20.33	0.0010	0.0251

7 days	twi_ss.10185.1|g.13679	0.82	8.05	20.28	0.0010	0.0252
7 days	twi_ss.11707.2|g.15505	-6.42	3.62	20.29	0.0010	0.0252
7 days	twi_ss.11938.1|g.15800	-1.74	5.45	20.28	0.0010	0.0252
7 days	twi_ss.15686.1|g.20257	-1.44	4.88	20.29	0.0010	0.0252
7 days	twi_ss.31287.1|g.42554	-4.44	3.96	20.28	0.0010	0.0252
7 days	twi_ss.3795.1|g.6223	-2.05	3.80	20.30	0.0010	0.0252
7 days	twi_ss.20264a.2|g.26040	1.87	4.32	20.26	0.0010	0.0253
7 days	twi_ss.31465.1|g.42876	-5.45	3.87	20.20	0.0010	0.0255
7 days	twi_ss.22192.1|g.28721	1.47	5.37	20.17	0.0010	0.0256
7 days	twi_ss.17834.2|g.22742	0.85	6.20	20.16	0.0011	0.0257
7 days	twi_ss.11538.1|g.15391	1.69	6.49	20.15	0.0011	0.0257
7 days	twi_ss.5925.1|g.8812	0.79	6.33	20.15	0.0011	0.0257
7 days	twi_ss.20162.1|g.25923	-4.82	3.23	23.86	0.0011	0.0257
7 days	twi_ss.30027a.7|g.40534	3.21	4.31	20.11	0.0011	0.0258
7 days	twi_ss.31962.2|g.43579	-5.42	3.57	20.04	0.0011	0.0261
7 days	twi_ss.14260.2|g.18549	2.16	4.23	20.04	0.0011	0.0261
7 days	twi_ss.1717.1|g.3035	-6.36	3.26	20.03	0.0011	0.0261
7 days	twi_ss.12588.1|g.16705	-1.73	4.28	20.02	0.0011	0.0262
7 days	twi_ss.3404.1|g.5787	-6.07	2.77	31.66	0.0011	0.0262
7 days	twi_ss.7573.1|g.10627	-2.44	4.32	19.98	0.0011	0.0263
7 days	twi_ss.25969.1|g.34284	-1.36	5.36	19.97	0.0011	0.0263
7 days	twi_ss.31618.1|g.43019	0.89	7.34	19.97	0.0011	0.0263
7 days	twi_ss.5784.2|g.8639	5.27	2.17	19.95	0.0011	0.0264
7 days	twi_ss.8819.1|g.12011	5.49	3.28	19.95	0.0011	0.0264
7 days	twi_ss.19241.2|g.24668	-7.88	4.31	19.92	0.0011	0.0264
7 days	twi_ss.21635a.2|g.28002	0.78	7.15	19.92	0.0011	0.0264
7 days	twi_ss.30222b.8|g.40809	-1.85	4.72	19.93	0.0011	0.0264
7 days	twi_ss.31691.1|g.43139	1.33	9.12	19.92	0.0011	0.0264
7 days	twi_ss.4014.1|g.6434	-1.68	5.37	19.93	0.0011	0.0264
7 days	twi_ss.459b.3|g.814	-1.22	6.78	19.93	0.0011	0.0264
7 days	twi_ss.7858.4|g.10914	3.28	5.42	19.93	0.0011	0.0264
7 days	twi_ss.19202.1|g.24617	0.63	6.38	19.91	0.0011	0.0264
7 days	twi_ss.5628.6|g.8385	6.49	3.90	19.88	0.0011	0.0265

7 days	twi_ss.6506.1|g.9518	0.98	6.73	19.86	0.0011	0.0265
7 days	twi_ss.8695.1|g.11850	-3.29	3.83	19.87	0.0011	0.0265
7 days	twi_ss.21663.2|g.27929	7.55	4.05	23.48	0.0011	0.0266
7 days	twi_ss.8118.1|g.11181	0.64	7.87	19.82	0.0011	0.0267
7 days	twi_ss.14090.1|g.18371	1.76	8.15	19.80	0.0011	0.0267
7 days	twi_ss.14962.1|g.19413	0.81	6.30	19.79	0.0011	0.0267
7 days	twi_ss.27090.1|g.36008	-1.26	7.36	19.79	0.0011	0.0267
7 days	twi_ss.670.2|g.1158	-0.84	6.07	19.80	0.0011	0.0267
7 days	twi_ss.9193.1|g.12381	-2.58	4.31	19.81	0.0011	0.0267
7 days	twi_ss.10334.1|g.13899	1.18	6.98	19.78	0.0011	0.0267
7 days	twi_ss.25151.1|g.33065	-3.07	3.84	19.77	0.0011	0.0268
7 days	twi_ss.9071a.3|g.12242	1.43	5.93	19.77	0.0011	0.0268
7 days	twi_ss.20032.1|g.25789	-1.70	6.07	19.76	0.0011	0.0268
7 days	twi_ss.26361a.2|g.34929	-1.15	6.90	19.74	0.0011	0.0269
7 days	twi_ss.8150.1|g.11239	1.11	6.70	19.74	0.0011	0.0269
7 days	twi_ss.16506.1|g.20593	-2.43	10.69	19.71	0.0011	0.0270
7 days	twi_ss.27854.5|g.37041	-1.25	7.13	19.71	0.0011	0.0270
7 days	twi_ss.26689.1|g.35443	-1.39	6.64	19.70	0.0011	0.0270
7 days	twi_ss.20177.1|g.25924	-1.62	4.90	19.68	0.0011	0.0271
7 days	twi_ss.27840.1|g.37020	1.87	4.17	19.68	0.0011	0.0271
7 days	twi_ss.31865b.1|g.43408	1.09	6.33	19.67	0.0011	0.0271
7 days	twi_ss.20145.1|g.25895	-1.79	5.64	19.66	0.0012	0.0271
7 days	twi_ss.5554.1|g.8429	1.03	5.30	19.66	0.0012	0.0271
7 days	twi_ss.14941.1|g.19485	1.74	4.14	19.63	0.0012	0.0271
7 days	twi_ss.26922.1|g.35773	1.28	6.95	19.63	0.0012	0.0271
7 days	twi_ss.27673.1|g.36873	1.31	6.38	19.63	0.0012	0.0271
7 days	twi_ss.30798.1|g.41762	-1.25	7.01	19.63	0.0012	0.0271
7 days	twi_ss.25320.1|g.33273	1.12	6.76	19.62	0.0012	0.0272
7 days	twi_ss.18881a.2|g.23976	-4.71	5.02	19.60	0.0012	0.0272
7 days	twi_ss.24846a.1|g.32671	-1.23	6.44	19.61	0.0012	0.0272
7 days	twi_ss.9553.1|g.12824	-2.94	3.44	19.60	0.0012	0.0272
7 days	twi_ss.6230.1|g.9268	-1.25	5.74	19.59	0.0012	0.0272
7 days	twi_ss.30985.2|g.42111	-1.65	4.86	19.58	0.0012	0.0272

7 days	twi_ss.16684.1|g.21353	1.00	5.40	19.56	0.0012	0.0273
7 days	twi_ss.20587.1|g.26691	-2.77	4.40	19.57	0.0012	0.0273
7 days	twi_ss.21951.1|g.28458	-1.23	5.20	19.54	0.0012	0.0274
7 days	twi_ss.24283.1|g.31722	0.98	5.70	19.53	0.0012	0.0274
7 days	twi_ss.30978.1|g.41821	0.76	5.98	19.54	0.0012	0.0274
7 days	twi_ss.19741a.2|g.25399	-2.50	3.42	19.52	0.0012	0.0274
7 days	twi_ss.2660.1|g.4835	-5.41	2.87	23.04	0.0012	0.0274
7 days	twi_ss.9212.1|g.12409	-3.38	3.81	19.51	0.0012	0.0274
7 days	twi_ss.18662.1|g.23811	1.96	4.09	19.50	0.0012	0.0274
7 days	twi_ss.2278.1|g.4035	0.90	6.07	19.50	0.0012	0.0274
7 days	twi_ss.26936.1|g.35728	-4.46	3.52	19.50	0.0012	0.0274
7 days	twi_ss.11382.5|g.15021	7.29	3.27	19.48	0.0012	0.0275
7 days	twi_ss.6202.1|g.9125	2.11	4.99	19.48	0.0012	0.0275
7 days	twi_ss.1616.1|g.2829	-1.45	7.25	19.46	0.0012	0.0275
7 days	twi_ss.1801.1|g.3187	1.37	6.16	19.45	0.0012	0.0276
7 days	twi_ss.10968.1|g.14529	-2.50	3.61	19.45	0.0012	0.0276
7 days	twi_ss.2422.2|g.4429	5.42	4.95	19.44	0.0012	0.0276
7 days	twi_ss.6365.1|g.9392	1.32	8.35	19.44	0.0012	0.0276
7 days	twi_ss.12246.2|g.16397	4.42	6.65	19.41	0.0012	0.0277
7 days	twi_ss.1424.2|g.2514	-7.55	3.74	19.39	0.0012	0.0278
7 days	twi_ss.16264.1|g.20846	0.81	6.35	19.36	0.0012	0.0279
7 days	twi_ss.5245.1|g.7897	-3.15	3.55	19.36	0.0012	0.0279
7 days	twi_ss.27464.1|g.36589	-2.07	4.34	19.33	0.0012	0.0280
7 days	twi_ss.28493.1|g.38148	-2.31	4.21	19.33	0.0012	0.0280
7 days	twi_ss.6267.1|g.9312	-6.80	3.22	30.27	0.0012	0.0280
7 days	twi_ss.23266.1|g.29923	1.46	4.22	19.31	0.0012	0.0281
7 days	twi_ss.25939.4|g.34236	-5.97	2.73	22.77	0.0012	0.0281
7 days	twi_ss.27364.1|g.36414	0.62	9.50	19.22	0.0012	0.0285
7 days	twi_ss.5080.1|g.7730	-2.07	4.71	19.22	0.0012	0.0285
7 days	twi_ss.29986.1|g.40498	-4.87	3.65	19.21	0.0012	0.0285
7 days	twi_ss.22743.5|g.29082	-4.59	4.03	19.19	0.0013	0.0286
7 days	twi_ss.12118.1|g.16075	-1.47	8.44	19.18	0.0013	0.0286
7 days	twi_ss.31635.1|g.43104	-2.72	4.12	19.13	0.0013	0.0289

7 days	twi_ss.2254.1|g.3942	-0.62	8.64	19.10	0.0013	0.0290
7 days	twi_ss.24527.1|g.32096	-1.94	6.16	19.08	0.0013	0.0290
7 days	twi_ss.27268a.1|g.36251	-0.99	7.46	19.08	0.0013	0.0290
7 days	twi_ss.10792.1|g.14433	2.38	3.53	19.07	0.0013	0.0291
7 days	twi_ss.22433.4|g.28986	5.84	2.44	19.06	0.0013	0.0291
7 days	twi_ss.6299.1|g.9323	-2.79	6.05	19.05	0.0013	0.0291
7 days	twi_ss.10631.1|g.14271	1.32	4.98	18.99	0.0013	0.0292
7 days	twi_ss.11378.4|g.14981	-5.90	3.57	22.38	0.0013	0.0292
7 days	twi_ss.12133.1|g.16028	-1.65	5.23	19.00	0.0013	0.0292
7 days	twi_ss.12878.1|g.17063	-1.60	6.15	18.99	0.0013	0.0292
7 days	twi_ss.1785.1|g.3176	1.13	5.98	19.02	0.0013	0.0292
7 days	twi_ss.1853b.1|g.3271	1.25	5.30	19.02	0.0013	0.0292
7 days	twi_ss.24837.1|g.32677	1.39	6.50	18.99	0.0013	0.0292
7 days	twi_ss.2594.1|g.4760	0.73	7.28	19.00	0.0013	0.0292
7 days	twi_ss.29938.2|g.40355	-2.48	4.74	19.00	0.0013	0.0292
7 days	twi_ss.3480b.3|g.5862	-7.09	3.31	18.99	0.0013	0.0292
7 days	twi_ss.27291.1|g.36220	-1.53	4.56	18.98	0.0013	0.0293
7 days	twi_ss.30654b.2|g.41481	-5.28	3.55	18.97	0.0013	0.0293
7 days	twi_ss.29121.1|g.39030	1.60	4.59	18.96	0.0013	0.0293
7 days	twi_ss.31490.1|g.42882	-2.60	4.19	18.96	0.0013	0.0293
7 days	twi_ss.7454.1|g.10476	2.29	5.71	18.96	0.0013	0.0293
7 days	twi_ss.8094.1|g.11173	-1.74	5.76	18.95	0.0013	0.0293
7 days	twi_ss.10283.1|g.13790	2.21	6.08	18.94	0.0013	0.0293
7 days	twi_ss.24554.1|g.32097	1.56	4.92	18.94	0.0013	0.0293
7 days	twi_ss.8051.1|g.11103	0.84	6.52	18.94	0.0013	0.0293
7 days	twi_ss.1728.1|g.3067	-2.26	4.41	18.89	0.0013	0.0294
7 days	twi_ss.18805.1|g.24051	-5.65	2.68	22.27	0.0013	0.0294
7 days	twi_ss.20143.1|g.25882	0.79	6.40	18.89	0.0013	0.0294
7 days	twi_ss.20143.2|g.25887	0.79	6.40	18.89	0.0013	0.0294
7 days	twi_ss.20143.3|g.25884	0.79	6.40	18.89	0.0013	0.0294
7 days	twi_ss.20143.4|g.25883	0.79	6.40	18.89	0.0013	0.0294
7 days	twi_ss.26054.4|g.34465	0.74	9.01	18.89	0.0013	0.0294
7 days	twi_ss.28969.1|g.38861	0.97	7.32	18.91	0.0013	0.0294

7 days	twi_ss.31274.1|g.42556	0.95	6.13	18.92	0.0013	0.0294
7 days	twi_ss.6503.1|g.9536	1.60	6.34	18.91	0.0013	0.0294
7 days	twi_ss.6810a.1|g.9870	-1.61	5.85	18.90	0.0013	0.0294
7 days	twi_ss.7683.1|g.10763	1.33	4.60	18.92	0.0013	0.0294
7 days	twi_ss.1489.2|g.2552	-6.85	3.14	18.87	0.0013	0.0294
7 days	twi_ss.30101a.1|g.40628	-2.32	4.00	18.87	0.0013	0.0294
7 days	twi_ss.20778.1|g.26878	-0.84	5.82	18.87	0.0013	0.0294
7 days	twi_ss.7665.9|g.10738	-1.54	6.41	18.84	0.0013	0.0295
7 days	twi_ss.8542.1|g.11697	1.20	5.50	18.84	0.0013	0.0295
7 days	twi_ss.22588b.1|g.29167	-1.29	5.65	18.83	0.0013	0.0295
7 days	twi_ss.25105.5|g.32924	7.13	3.87	18.83	0.0013	0.0295
7 days	twi_ss.6167a.1|g.9147	1.27	4.72	18.83	0.0013	0.0295
7 days	twi_ss.9666b.2|g.12978	1.40	7.66	18.82	0.0013	0.0295
7 days	twi_ss.10520.2|g.14152	-3.78	4.07	18.81	0.0013	0.0296
7 days	twi_ss.5558.1|g.8419	1.64	4.74	18.80	0.0013	0.0296
7 days	twi_ss.4684.1|g.7239	0.89	5.82	18.78	0.0013	0.0297
7 days	twi_ss.26901a.2|g.35658	2.06	5.07	18.77	0.0014	0.0297
7 days	twi_ss.20566.1|g.26524	1.08	5.99	18.76	0.0014	0.0298
7 days	twi_ss.21329.1|g.27617	6.58	4.10	18.75	0.0014	0.0298
7 days	twi_ss.2681.1|g.4899	0.92	7.96	18.75	0.0014	0.0298
7 days	twi_ss.29213.1|g.39249	-1.00	5.88	18.70	0.0014	0.0300
7 days	twi_ss.16136.1|g.20730	1.31	5.17	18.69	0.0014	0.0300
7 days	twi_ss.5074.1|g.7728	-1.04	5.75	18.69	0.0014	0.0300
7 days	twi_ss.20060.1|g.25832	0.73	7.96	18.68	0.0014	0.0301
7 days	twi_ss.168.1|g.248	-2.15	4.44	18.65	0.0014	0.0302
7 days	twi_ss.22218b.1|g.28740	-4.95	3.36	18.63	0.0014	0.0302
7 days	twi_ss.28156b.3|g.37499	-2.32	6.09	18.63	0.0014	0.0302
7 days	twi_ss.291.1|g.638	0.65	7.46	18.63	0.0014	0.0302
7 days	twi_ss.6760.1|g.9845	3.32	3.10	18.63	0.0014	0.0302
7 days	twi_ss.8420.5|g.11577	4.66	1.96	18.63	0.0014	0.0302
7 days	twi_ss.8420.6|g.11578	4.66	1.96	18.63	0.0014	0.0302
7 days	twi_ss.4595.1|g.7163	-1.71	5.45	18.61	0.0014	0.0303
7 days	twi_ss.24034.3|g.31248	7.97	4.07	18.58	0.0014	0.0304

7 days	twi_ss.27883.3|g.37126	1.12	7.04	18.58	0.0014	0.0304
7 days	twi_ss.4854.1|g.7451	-1.76	6.00	18.59	0.0014	0.0304
7 days	twi_ss.6781.2|g.9755	1.44	5.35	18.58	0.0014	0.0304
7 days	twi_ss.8555.1|g.11707	2.01	4.67	18.57	0.0014	0.0304
7 days	twi_ss.4563.1|g.7102	-7.33	3.99	18.57	0.0014	0.0304
7 days	twi_ss.29229.1|g.39186	-3.43	4.00	18.55	0.0014	0.0305
7 days	twi_ss.1818.2|g.3205	1.34	5.72	18.53	0.0014	0.0306
7 days	twi_ss.4977.4|g.7553	-2.28	4.91	18.52	0.0014	0.0306
7 days	twi_ss.4344.1|g.6862	-7.34	3.76	21.73	0.0014	0.0306
7 days	twi_ss.28018.1|g.37302	0.76	9.52	18.50	0.0014	0.0307
7 days	twi_ss.29791.4|g.40179	-1.42	4.76	18.50	0.0014	0.0307
7 days	twi_ss.16483.1|g.21068	-1.61	5.05	18.48	0.0014	0.0308
7 days	twi_ss.18539.3|g.23686	1.92	4.82	18.46	0.0014	0.0309
7 days	twi_ss.31539a.2|g.42932	-4.62	3.37	21.65	0.0014	0.0309
7 days	twi_ss.4977.13|g.7541	1.55	5.56	18.44	0.0014	0.0309
7 days	twi_ss.14643.1|g.19097	-5.25	2.42	28.43	0.0014	0.0310
7 days	twi_ss.1925.1|g.3419	-1.30	5.18	18.42	0.0014	0.0310
7 days	twi_ss.14942.2|g.19462	-4.69	2.85	21.60	0.0014	0.0310
7 days	twi_ss.2886.1|g.5173	-7.28	3.41	28.39	0.0014	0.0311
7 days	twi_ss.18396.1|g.23448	-2.60	5.06	21.56	0.0015	0.0311
7 days	twi_ss.15421.1|g.19963	-3.24	4.00	18.37	0.0015	0.0312
7 days	twi_ss.389.1|g.744	-1.24	6.58	18.36	0.0015	0.0312
7 days	twi_ss.10285c.9|g.13768	-1.53	6.03	18.35	0.0015	0.0313
7 days	twi_ss.17772.1|g.22652	1.08	5.39	18.35	0.0015	0.0313
7 days	twi_ss.5609.1|g.8381	0.44	8.78	18.34	0.0015	0.0313
7 days	twi_ss.15156.3|g.19688	-7.10	3.46	18.33	0.0015	0.0313
7 days	twi_ss.3806.1|g.6237	1.52	4.39	18.30	0.0015	0.0315
7 days	twi_ss.3701.1|g.6078	0.92	5.76	18.29	0.0015	0.0315
7 days	twi_ss.17480.1|g.22291	1.12	5.97	18.27	0.0015	0.0316
7 days	twi_ss.22996.1|g.29640	-3.15	3.73	18.25	0.0015	0.0317
7 days	twi_ss.20501.1|g.26442	1.19	5.64	18.25	0.0015	0.0317
7 days	twi_ss.10211.1|g.13735	-1.34	4.25	18.22	0.0015	0.0318
7 days	twi_ss.12767.1|g.16858	-5.88	3.32	18.21	0.0015	0.0319

7 days	twi_ss.13656.1|g.18009	-4.13	3.52	18.21	0.0015	0.0319
7 days	twi_ss.14987.1|g.19496	3.16	3.91	18.20	0.0015	0.0319
7 days	twi_ss.2753.1|g.5003	-2.04	4.27	18.19	0.0015	0.0319
7 days	twi_ss.31964.1|g.43602	-2.26	4.21	18.18	0.0015	0.0320
7 days	twi_ss.29255.3|g.39220	1.45	7.27	18.17	0.0015	0.0320
7 days	twi_ss.23459.1|g.30108	1.45	6.53	18.17	0.0015	0.0320
7 days	twi_ss.14331.1|g.18637	-3.63	4.93	18.15	0.0015	0.0321
7 days	twi_ss.26824.2|g.35518	1.12	5.69	18.11	0.0015	0.0323
7 days	twi_ss.24113.4|g.31396	-0.50	7.08	18.09	0.0015	0.0324
7 days	twi_ss.30134.4|g.40703	3.06	8.64	18.08	0.0015	0.0325
7 days	twi_ss.3498.1|g.5871	0.70	7.09	18.06	0.0015	0.0325
7 days	twi_ss.29955.1|g.40412	0.85	5.48	18.05	0.0015	0.0326
7 days	twi_ss.27326.1|g.36341	-6.53	4.80	18.05	0.0016	0.0326
7 days	twi_ss.25883.1|g.34164	0.63	8.82	18.03	0.0016	0.0327
7 days	twi_ss.30972.1|g.41798	-1.16	8.77	18.03	0.0016	0.0327
7 days	twi_ss.1959a.1|g.3428	1.74	5.06	18.00	0.0016	0.0328
7 days	twi_ss.20099.1|g.25857	-4.52	2.06	27.59	0.0016	0.0328
7 days	twi_ss.24873.1|g.32725	1.10	6.17	17.99	0.0016	0.0328
7 days	twi_ss.21665.3|g.27902	7.25	4.00	17.97	0.0016	0.0330
7 days	twi_ss.4371.1|g.6877	4.24	2.18	21.02	0.0016	0.0330
7 days	twi_ss.5605.2|g.8341	1.52	4.34	17.94	0.0016	0.0331
7 days	twi_ss.19827.1|g.25491	0.82	6.28	17.94	0.0016	0.0331
7 days	twi_ss.1027.1|g.1857	-1.34	7.81	17.92	0.0016	0.0332
7 days	twi_ss.1027.2|g.1854	-1.34	7.81	17.92	0.0016	0.0332
7 days	twi_ss.29329.1|g.39338	-2.88	4.08	17.89	0.0016	0.0333
7 days	twi_ss.1875.1|g.3340	1.43	7.17	17.84	0.0016	0.0336
7 days	twi_ss.339.1|g.675	3.31	3.79	17.84	0.0016	0.0336
7 days	twi_ss.7559.1|g.10607	-0.55	7.66	17.81	0.0016	0.0338
7 days	twi_ss.28268.1|g.37789	-2.84	5.24	17.80	0.0016	0.0338
7 days	twi_ss.31357.1|g.42605	1.33	5.49	17.80	0.0016	0.0338
7 days	twi_ss.1244.5|g.2216	2.97	4.89	17.79	0.0016	0.0338
7 days	twi_ss.12586.1|g.16702	1.65	6.69	17.78	0.0016	0.0338
7 days	twi_ss.18762.1|g.23959	-1.69	4.79	17.77	0.0016	0.0339

7 days	twi_ss.22501.1|g.29071	-0.64	7.53	17.76	0.0016	0.0339
7 days	twi_ss.26399.1|g.34970	2.02	5.91	17.76	0.0016	0.0339
7 days	twi_ss.16883.1|g.21508	1.37	4.72	17.76	0.0016	0.0339
7 days	twi_ss.25312.4|g.33266	3.13	6.12	17.75	0.0016	0.0339
7 days	twi_ss.13146.5|g.17470	2.44	5.37	17.71	0.0017	0.0342
7 days	twi_ss.13816.1|g.18137	0.84	7.56	17.71	0.0017	0.0342
7 days	twi_ss.10155.1|g.13612	1.30	5.05	17.69	0.0017	0.0342
7 days	twi_ss.2403a.1|g.4147	1.07	7.15	17.69	0.0017	0.0342
7 days	twi_ss.27810.1|g.37027	-3.81	5.08	17.67	0.0017	0.0344
7 days	twi_ss.21665.1|g.27904	3.11	6.25	17.66	0.0017	0.0344
7 days	twi_ss.16839.1|g.21484	-0.86	6.57	17.63	0.0017	0.0346
7 days	twi_ss.17089.5|g.21849	-4.53	4.16	17.62	0.0017	0.0346
7 days	twi_ss.22099.1|g.28606	-1.72	5.22	17.62	0.0017	0.0346
7 days	twi_ss.28101b.2|g.37487	7.17	3.35	17.56	0.0017	0.0350
7 days	twi_ss.31991.1|g.43618	0.88	5.96	17.55	0.0017	0.0350
7 days	twi_ss.13620.1|g.17972	1.85	5.02	17.52	0.0017	0.0352
7 days	twi_ss.28706.1|g.38429	1.03	6.38	17.52	0.0017	0.0352
7 days	twi_ss.29868.1|g.40338	-5.86	3.81	20.44	0.0017	0.0352
7 days	twi_ss.14951.1|g.19385	1.24	6.27	17.50	0.0017	0.0352
7 days	twi_ss.2398a.2|g.4265	1.91	5.28	17.50	0.0017	0.0353
7 days	twi_ss.2690.2|g.4896	1.50	7.59	17.45	0.0017	0.0355
7 days	twi_ss.21143.1|g.27343	6.93	3.14	20.34	0.0017	0.0356
7 days	twi_ss.8125.1|g.11189	1.29	6.58	17.43	0.0017	0.0356
7 days	twi_ss.941.2|g.1597	-2.85	5.40	17.43	0.0017	0.0356
7 days	twi_ss.26608.1|g.35307	-2.69	4.22	17.42	0.0017	0.0356
7 days	twi_ss.29366b.6|g.39462	-7.79	3.96	17.42	0.0018	0.0356
7 days	twi_ss.6137.1|g.9055	0.85	5.52	17.42	0.0018	0.0356
7 days	twi_ss.1553.1|g.2746	-0.97	5.65	17.41	0.0018	0.0357
7 days	twi_ss.4527.1|g.7040	2.51	3.67	17.38	0.0018	0.0358
7 days	twi_ss.25558a.4|g.33612	-1.57	5.00	17.37	0.0018	0.0359
7 days	twi_ss.22261.1|g.28816	-5.00	3.08	20.22	0.0018	0.0361
7 days	twi_ss.26497.1|g.35119	0.61	8.42	17.34	0.0018	0.0361
7 days	twi_ss.13329.1|g.17558	-1.18	6.34	17.33	0.0018	0.0361

7 days	twi_ss.5559.1|g.8412	1.57	5.79	17.33	0.0018	0.0361
7 days	twi_ss.19927.2|g.25679	-5.51	3.16	17.32	0.0018	0.0362
7 days	twi_ss.22611.1|g.29205	-2.49	5.80	17.30	0.0018	0.0363
7 days	twi_ss.31062.2|g.42240	-6.39	2.86	17.29	0.0018	0.0363
7 days	twi_ss.13937.2|g.18266	-4.76	2.20	26.12	0.0018	0.0366
7 days	twi_ss.258.2|g.525	5.16	2.69	17.24	0.0018	0.0366
7 days	twi_ss.26617.1|g.35286	-3.58	3.01	17.24	0.0018	0.0366
7 days	twi_ss.4977.2|g.7539	-5.61	3.31	20.09	0.0018	0.0366
7 days	twi_ss.22200.1|g.28758	-1.56	5.33	17.22	0.0018	0.0367
7 days	twi_ss.17708.1|g.22577	-2.17	4.17	17.21	0.0018	0.0367
7 days	twi_ss.31368.1|g.42696	1.54	4.45	17.20	0.0018	0.0368
7 days	twi_ss.24923.1|g.32789	-2.03	3.83	17.19	0.0018	0.0368
7 days	twi_ss.26310.1|g.34926	1.12	6.37	17.19	0.0018	0.0368
7 days	twi_ss.31816.1|g.43340	-2.91	4.56	17.20	0.0018	0.0368
7 days	twi_ss.18066.1|g.22951	0.69	6.41	17.17	0.0018	0.0369
7 days	twi_ss.25119.3|g.32978	0.55	8.31	17.16	0.0018	0.0370
7 days	twi_ss.1269.1|g.2281	-4.17	3.87	19.94	0.0019	0.0372
7 days	twi_ss.9822.1|g.13153	-1.76	4.90	17.11	0.0019	0.0372
7 days	twi_ss.2900.1|g.5200	-1.03	6.12	17.10	0.0019	0.0373
7 days	twi_ss.11559.1|g.15376	-0.66	7.15	17.06	0.0019	0.0376
7 days	twi_ss.22064.1|g.28563	-1.66	5.07	17.06	0.0019	0.0376
7 days	twi_ss.80.1|g.182	1.10	6.35	17.06	0.0019	0.0376
7 days	twi_ss.29191.5|g.39121	6.76	3.31	17.05	0.0019	0.0376
7 days	twi_ss.20948.1|g.27058	1.62	4.28	17.03	0.0019	0.0377
7 days	twi_ss.31880.2|g.43485	0.64	6.62	16.99	0.0019	0.0380
7 days	twi_ss.6592.1|g.9620	-2.63	5.13	16.99	0.0019	0.0380
7 days	twi_ss.29627.1|g.39917	-1.36	8.50	16.98	0.0019	0.0380
7 days	twi_ss.6146.8|g.9087	5.57	2.47	19.69	0.0019	0.0384
7 days	twi_ss.18562a.1|g.23650	-0.74	7.23	16.93	0.0019	0.0384
7 days	twi_ss.30222a.5|g.40814	-5.51	2.37	16.92	0.0019	0.0384
7 days	twi_ss.31583b.5|g.43005	-1.65	4.85	19.68	0.0019	0.0384
7 days	twi_ss.10667.1|g.14302	-4.62	2.12	19.63	0.0019	0.0386
7 days	twi_ss.15848.1|g.20402	-1.84	3.90	16.88	0.0019	0.0386

7 days	twi_ss.29577.1|g.39830	-5.22	2.43	25.40	0.0019	0.0386
7 days	twi_ss.12746.1|g.16847	5.68	2.42	16.86	0.0020	0.0386
7 days	twi_ss.2185.2|g.3906	4.41	2.21	19.60	0.0020	0.0386
7 days	twi_ss.2677b.2|g.4824	-6.03	2.70	19.60	0.0020	0.0386
7 days	twi_ss.29223.1|g.39193	-4.37	3.95	16.86	0.0020	0.0386
7 days	twi_ss.4312.1|g.6748	9.18	6.38	16.86	0.0020	0.0386
7 days	twi_ss.6498.1|g.9563	1.45	4.74	16.87	0.0020	0.0386
7 days	twi_ss.249.2|g.539	-2.12	5.64	16.84	0.0020	0.0387
7 days	twi_ss.26492.1|g.35141	1.61	4.07	16.84	0.0020	0.0387
7 days	twi_ss.30545.1|g.41375	0.81	6.30	16.80	0.0020	0.0389
7 days	twi_ss.31269.1|g.42501	0.74	7.51	16.81	0.0020	0.0389
7 days	twi_ss.12809.1|g.16919	1.27	5.16	16.80	0.0020	0.0389
7 days	twi_ss.24735.8|g.32337	2.33	6.08	16.80	0.0020	0.0389
7 days	twi_ss.28220a.6|g.37616	-1.96	4.81	16.77	0.0020	0.0391
7 days	twi_ss.207.1|g.332	-1.02	8.02	16.77	0.0020	0.0391
7 days	twi_ss.21908.1|g.28418	1.30	5.05	16.76	0.0020	0.0391
7 days	twi_ss.2298.1|g.4053	-0.80	6.84	16.76	0.0020	0.0391
7 days	twi_ss.9298.1|g.12601	4.68	2.23	19.47	0.0020	0.0391
7 days	twi_ss.26574b.1|g.35220	-2.84	4.43	16.74	0.0020	0.0393
7 days	twi_ss.8565.1|g.11724	-3.36	3.88	16.73	0.0020	0.0393
7 days	twi_ss.8364b.1|g.11477	-1.09	5.59	16.73	0.0020	0.0393
7 days	twi_ss.25318.1|g.33284	1.15	5.11	16.72	0.0020	0.0394
7 days	twi_ss.10044.1|g.13359	-1.15	7.06	16.71	0.0020	0.0394
7 days	twi_ss.16562.1|g.21165	1.05	6.05	16.65	0.0020	0.0398
7 days	twi_ss.4424.1|g.6923	-1.43	4.40	16.64	0.0020	0.0399
7 days	twi_ss.10298c.2|g.13756	0.81	6.61	16.62	0.0021	0.0400
7 days	twi_ss.13102a.1|g.17415	1.07	9.23	16.62	0.0021	0.0400
7 days	twi_ss.1440.1|g.2613	2.12	5.91	16.62	0.0021	0.0400
7 days	twi_ss.9257.1|g.12473	-1.40	5.70	16.62	0.0021	0.0400
7 days	twi_ss.14366.1|g.18716	-3.51	3.91	16.61	0.0021	0.0400
7 days	twi_ss.18204.1|g.23100	0.86	7.83	16.59	0.0021	0.0402
7 days	twi_ss.29406a.2|g.39524	6.01	2.78	24.84	0.0021	0.0402
7 days	twi_ss.27570.2|g.36772	4.24	2.50	16.58	0.0021	0.0402

7 days	twi_ss.6554.1|g.9582	-0.83	6.74	16.58	0.0021	0.0402
7 days	twi_ss.20159.1|g.25916	0.61	6.77	16.55	0.0021	0.0403
7 days	twi_ss.30556.1|g.41363	-6.14	2.91	19.21	0.0021	0.0403
7 days	twi_ss.27913.1|g.37197	-1.63	4.71	16.55	0.0021	0.0403
7 days	twi_ss.3796.3|g.6235	3.95	4.60	16.54	0.0021	0.0404
7 days	twi_ss.18303.1|g.23257	-1.67	6.30	16.45	0.0021	0.0406
7 days	twi_ss.22092.1|g.28591	0.91	5.45	16.46	0.0021	0.0406
7 days	twi_ss.23250.2|g.29901	-1.78	4.65	16.47	0.0021	0.0406
7 days	twi_ss.23946a.1|g.31123	-1.89	3.74	16.46	0.0021	0.0406
7 days	twi_ss.24215.6|g.31582	6.92	3.31	16.45	0.0021	0.0406
7 days	twi_ss.26093.1|g.34588	0.87	7.31	16.44	0.0021	0.0406
7 days	twi_ss.28270a.6|g.37797	-4.50	1.99	24.65	0.0021	0.0406
7 days	twi_ss.30481.2|g.41260	1.34	6.73	16.48	0.0021	0.0406
7 days	twi_ss.30713.1|g.41607	0.99	5.24	16.47	0.0021	0.0406
7 days	twi_ss.31312.1|g.42622	7.29	4.66	16.44	0.0021	0.0406
7 days	twi_ss.31312.12|g.42616	7.29	4.66	16.44	0.0021	0.0406
7 days	twi_ss.31312.2|g.42613	7.29	4.66	16.44	0.0021	0.0406
7 days	twi_ss.31312.3|g.42615	7.29	4.66	16.44	0.0021	0.0406
7 days	twi_ss.31312.4|g.42619	7.29	4.66	16.44	0.0021	0.0406
7 days	twi_ss.31312.6|g.42614	7.29	4.66	16.44	0.0021	0.0406
7 days	twi_ss.31312.7|g.42623	7.29	4.66	16.44	0.0021	0.0406
7 days	twi_ss.3469.1|g.5855	-2.01	4.54	16.49	0.0021	0.0406
7 days	twi_ss.3531.2|g.5898	2.49	6.68	16.50	0.0021	0.0406
7 days	twi_ss.4609.1|g.7115	-1.51	6.44	16.49	0.0021	0.0406
7 days	twi_ss.5041.1|g.7696	-1.56	5.56	16.46	0.0021	0.0406
7 days	twi_ss.5590a.3|g.8362	4.30	3.24	19.08	0.0021	0.0406
7 days	twi_ss.6244b.3|g.9269	-2.14	3.63	16.47	0.0021	0.0406
7 days	twi_ss.6297.1|g.9321	-4.32	3.08	19.11	0.0021	0.0406
7 days	twi_ss.7032.1|g.10090	-2.46	3.47	16.50	0.0021	0.0406
7 days	twi_ss.8426.1|g.11615	-0.64	6.46	16.48	0.0021	0.0406
7 days	twi_ss.6189.1|g.9149	1.12	7.12	16.43	0.0021	0.0406
7 days	twi_ss.19586.1|g.25171	-2.10	3.96	16.43	0.0021	0.0406
7 days	twi_ss.28726.1|g.38471	-0.77	5.53	16.41	0.0021	0.0407

7 days	twi_ss.26087.1|g.34611	1.58	4.34	16.39	0.0021	0.0408
7 days	twi_ss.28887.1|g.38726	0.54	7.78	16.39	0.0021	0.0408
7 days	twi_ss.30208.1|g.40797	5.87	2.88	19.00	0.0022	0.0408
7 days	twi_ss.9415.1|g.12656	-2.06	3.71	16.39	0.0021	0.0408
7 days	twi_ss.12739.1|g.16880	1.64	4.06	16.38	0.0022	0.0408
7 days	twi_ss.10679.3|g.14341	-5.88	2.57	16.36	0.0022	0.0409
7 days	twi_ss.27194.1|g.36139	0.85	6.60	16.36	0.0022	0.0409
7 days	twi_ss.20754.3|g.26862	-7.91	4.08	24.39	0.0022	0.0410
7 days	twi_ss.22284b.2|g.28807	1.13	5.16	16.35	0.0022	0.0410
7 days	twi_ss.16187.1|g.20779	-5.35	2.72	16.34	0.0022	0.0410
7 days	twi_ss.26806.2|g.35580	-4.96	2.15	16.34	0.0022	0.0410
7 days	twi_ss.668.1|g.1194	4.46	5.88	16.34	0.0022	0.0410
7 days	twi_ss.11802b.2|g.15695	-0.46	7.33	16.32	0.0022	0.0411
7 days	twi_ss.27925b.2|g.37193	-1.92	4.45	16.32	0.0022	0.0411
7 days	twi_ss.13931.1|g.18262	1.69	4.82	16.29	0.0022	0.0413
7 days	twi_ss.22735.2|g.29344	-5.49	2.34	16.29	0.0022	0.0413
7 days	twi_ss.4274.1|g.6708	0.73	6.35	16.27	0.0022	0.0415
7 days	twi_ss.12906.1|g.17099	-1.39	5.90	16.26	0.0022	0.0415
7 days	twi_ss.17767.4|g.22645	-2.65	4.18	16.25	0.0022	0.0415
7 days	twi_ss.6132.1|g.8997	-3.69	3.48	16.25	0.0022	0.0415
7 days	twi_ss.7639b.1|g.10712	1.98	4.53	16.24	0.0022	0.0416
7 days	twi_ss.13059.1|g.17305	-2.90	3.99	16.24	0.0022	0.0416
7 days	twi_ss.26485.1|g.35191	1.50	4.12	16.23	0.0022	0.0417
7 days	twi_ss.21006.1|g.27116	-2.36	3.56	16.22	0.0022	0.0417
7 days	twi_ss.28601.1|g.38306	-5.01	2.46	18.78	0.0022	0.0417
7 days	twi_ss.29345.1|g.39441	-1.75	3.93	16.22	0.0022	0.0417
7 days	twi_ss.30173.6|g.40745	-1.19	5.06	16.21	0.0022	0.0417
7 days	twi_ss.30863.2|g.41963	-5.81	2.47	16.20	0.0022	0.0418
7 days	twi_ss.12013.1|g.15876	1.04	6.28	16.18	0.0022	0.0419
7 days	twi_ss.30479.1|g.41273	2.20	4.64	16.17	0.0022	0.0420
7 days	twi_ss.2395.1|g.4284	1.91	3.68	16.15	0.0023	0.0421
7 days	twi_ss.31609.1|g.43064	-2.10	6.29	16.15	0.0023	0.0421
7 days	twi_ss.10009.1|g.13306	0.85	5.38	16.14	0.0023	0.0421

7 days	twi_ss.19345.11|g.24815	5.96	3.23	16.14	0.0023	0.0421
7 days	twi_ss.29768.1|g.40059	-1.09	9.81	16.14	0.0023	0.0421
7 days	twi_ss.4254.1|g.6686	5.06	2.12	16.14	0.0023	0.0421
7 days	twi_ss.25622.1|g.33721	-2.24	4.95	16.13	0.0023	0.0421
7 days	twi_ss.14765.1|g.19181	-0.91	5.81	16.13	0.0023	0.0421
7 days	twi_ss.10313.1|g.13798	0.94	5.10	16.11	0.0023	0.0423
7 days	twi_ss.4972.1|g.7517	1.67	5.14	16.09	0.0023	0.0424
7 days	twi_ss.22218e.5|g.28735	3.68	4.83	16.09	0.0023	0.0424
7 days	twi_ss.556.1|g.1016	-8.31	4.31	18.59	0.0023	0.0426
7 days	twi_ss.14306.1|g.18665	-1.35	5.19	16.05	0.0023	0.0427
7 days	twi_ss.7233.1|g.10275	-2.03	3.74	16.05	0.0023	0.0427
7 days	twi_ss.1651.1|g.2905	-2.40	4.50	16.04	0.0023	0.0427
7 days	twi_ss.23382.1|g.30079	0.64	9.28	16.02	0.0023	0.0429
7 days	twi_ss.8661b.1|g.11804	-1.49	4.34	16.02	0.0023	0.0429
7 days	twi_ss.2269.2|g.4022	-1.76	4.47	16.01	0.0023	0.0429
7 days	twi_ss.27269.2|g.36254	7.14	3.98	16.00	0.0023	0.0430
7 days	twi_ss.30783.1|g.41778	-1.58	6.08	15.99	0.0023	0.0430
7 days	twi_ss.5306.1|g.7938	0.57	7.21	15.99	0.0023	0.0430
7 days	twi_ss.16870.1|g.21502	-1.07	5.69	15.96	0.0023	0.0433
7 days	twi_ss.22100.1|g.28615	-6.26	2.90	15.95	0.0024	0.0433
7 days	twi_ss.10479.1|g.14122	1.82	5.12	15.94	0.0024	0.0433
7 days	twi_ss.1749.2|g.3071	1.48	5.41	15.94	0.0024	0.0433
7 days	twi_ss.17564c.8|g.22378	1.11	5.56	15.94	0.0024	0.0433
7 days	twi_ss.25333.1|g.33360	0.58	8.35	15.94	0.0024	0.0433
7 days	twi_ss.20311b.6|g.26145	-4.35	3.02	15.91	0.0024	0.0435
7 days	twi_ss.20899.1|g.27026	-1.98	5.49	15.90	0.0024	0.0436
7 days	twi_ss.31516.1|g.42907	-1.68	4.78	15.88	0.0024	0.0437
7 days	twi_ss.8442a.1|g.11623	-6.26	2.85	18.35	0.0024	0.0437
7 days	twi_ss.29126.1|g.39032	3.24	4.25	15.86	0.0024	0.0439
7 days	twi_ss.5730.2|g.8594	-4.97	2.14	15.85	0.0024	0.0440
7 days	twi_ss.26988b.1|g.35833	-1.72	5.53	15.84	0.0024	0.0440
7 days	twi_ss.28345b.2|g.37899	-2.47	3.51	15.84	0.0024	0.0440
7 days	twi_ss.969.1|g.1738	-1.77	3.90	15.84	0.0024	0.0440

7 days	twi_ss.18539.1|g.23685	1.22	5.91	15.83	0.0024	0.0440
7 days	twi_ss.16904.1|g.21542	1.06	4.92	15.81	0.0024	0.0442
7 days	twi_ss.22435.1|g.28993	1.21	5.66	15.81	0.0024	0.0442
7 days	twi_ss.23634.1|g.30440	0.94	5.61	15.81	0.0024	0.0442
7 days	twi_ss.2996.1|g.5323	-1.14	5.08	15.80	0.0024	0.0442
7 days	twi_ss.1420.1|g.2668	-1.45	5.37	15.79	0.0024	0.0443
7 days	twi_ss.22977.1|g.29622	-1.69	4.65	15.78	0.0024	0.0443
7 days	twi_ss.9692.2|g.13027	-8.33	4.42	18.21	0.0024	0.0444
7 days	twi_ss.26916.1|g.35674	0.96	7.10	15.76	0.0024	0.0445
7 days	twi_ss.12131.1|g.16090	0.84	7.20	15.72	0.0025	0.0446
7 days	twi_ss.16175b.1|g.20748	1.58	7.85	15.72	0.0025	0.0446
7 days	twi_ss.16912.1|g.21554	-6.04	3.04	23.23	0.0025	0.0446
7 days	twi_ss.19701b.2|g.25349	-0.76	8.39	15.72	0.0025	0.0446
7 days	twi_ss.22469.1|g.29045	-1.20	5.66	15.72	0.0025	0.0446
7 days	twi_ss.23167.1|g.29819	-1.86	4.35	15.72	0.0025	0.0446
7 days	twi_ss.23512.1|g.30174	1.84	5.40	15.73	0.0025	0.0446
7 days	twi_ss.24824.1|g.32616	0.44	8.29	15.72	0.0025	0.0446
7 days	twi_ss.3480b.2|g.5863	-0.71	7.73	15.72	0.0025	0.0446
7 days	twi_ss.21105.11|g.27280	4.26	4.60	15.71	0.0025	0.0446
7 days	twi_ss.8242a.1|g.11314	-6.11	2.69	18.12	0.0025	0.0447
7 days	twi_ss.11143.1|g.14768	-1.33	9.31	15.69	0.0025	0.0447
7 days	twi_ss.82.1|g.30	-1.13	5.75	15.70	0.0025	0.0447
7 days	twi_ss.16552.1|g.21247	-4.41	3.19	15.68	0.0025	0.0448
7 days	twi_ss.18453.1|g.23507	0.91	6.97	15.67	0.0025	0.0449
7 days	twi_ss.8397.1|g.11532	-2.12	3.77	15.63	0.0025	0.0453
7 days	twi_ss.1008a.1|g.1843	1.24	4.98	15.60	0.0025	0.0455
7 days	twi_ss.17902b.1|g.22716	0.79	11.59	15.60	0.0025	0.0455
7 days	twi_ss.1272.1|g.2274	0.91	7.59	15.59	0.0025	0.0456
7 days	twi_ss.21664c.3|g.27917	4.09	5.73	15.58	0.0025	0.0456
7 days	twi_ss.31424b.2|g.42777	-2.77	4.29	15.58	0.0025	0.0456
7 days	twi_ss.31022.2|g.42191	3.63	3.74	15.57	0.0025	0.0456
7 days	twi_ss.22687.1|g.29325	1.08	5.72	15.56	0.0026	0.0457
7 days	twi_ss.24302.1|g.31671	0.64	10.46	15.55	0.0026	0.0458

7 days	twi_ss.25872.13|g.34152	-6.16	3.56	17.92	0.0026	0.0458
7 days	twi_ss.5522.1|g.8292	0.35	8.10	15.54	0.0026	0.0458
7 days	twi_ss.23800.1|g.30819	1.06	5.09	15.53	0.0026	0.0459
7 days	twi_ss.24614.1|g.32256	0.71	5.91	15.53	0.0026	0.0459
7 days	twi_ss.31865a.2|g.43393	1.04	5.70	15.53	0.0026	0.0459
7 days	twi_ss.13698.2|g.18023	1.69	5.46	15.51	0.0026	0.0461
7 days	twi_ss.24063.1|g.31273	-1.24	5.38	15.50	0.0026	0.0461
7 days	twi_ss.8089.1|g.11160	-1.15	5.02	15.49	0.0026	0.0462
7 days	twi_ss.5718.1|g.8555	2.14	3.91	15.48	0.0026	0.0462
7 days	twi_ss.8906.1|g.12066	1.10	4.87	15.46	0.0026	0.0464
7 days	twi_ss.20760.1|g.26839	0.92	7.85	15.44	0.0026	0.0466
7 days	twi_ss.18660b.1|g.23765	2.71	3.70	15.43	0.0026	0.0466
7 days	twi_ss.31247.1|g.42483	5.22	6.49	15.42	0.0026	0.0467
7 days	twi_ss.588.1|g.1087	-5.29	5.48	15.41	0.0026	0.0468
7 days	twi_ss.11444.1|g.15092	2.96	5.71	15.39	0.0026	0.0469
7 days	twi_ss.13635.1|g.17990	1.96	3.68	15.37	0.0027	0.0470
7 days	twi_ss.15599.1|g.20166	-0.56	7.05	15.37	0.0027	0.0470
7 days	twi_ss.28155.1|g.37497	-3.81	3.03	15.37	0.0027	0.0470
7 days	twi_ss.5475.1|g.8261	-1.85	3.78	15.38	0.0026	0.0470
7 days	twi_ss.5805b.1|g.8713	1.87	4.64	15.37	0.0027	0.0470
7 days	twi_ss.13443.1|g.17730	1.05	5.26	15.37	0.0027	0.0470
7 days	twi_ss.19570.12|g.25102	-4.77	4.41	15.35	0.0027	0.0471
7 days	twi_ss.7774.2|g.10873	-1.47	4.60	15.35	0.0027	0.0472
7 days	twi_ss.16945.1|g.21593	1.21	4.68	15.33	0.0027	0.0473
7 days	twi_ss.25210.1|g.33117	-1.44	6.26	15.33	0.0027	0.0473
7 days	twi_ss.2951.1|g.5301	-5.27	2.71	17.65	0.0027	0.0473
7 days	twi_ss.25247.1|g.33252	-1.21	5.31	15.31	0.0027	0.0474
7 days	twi_ss.10157.2|g.13613	6.91	3.65	15.29	0.0027	0.0476
7 days	twi_ss.19188.1|g.24583	-4.92	2.49	17.60	0.0027	0.0476
7 days	twi_ss.2382.1|g.4289	1.11	5.79	15.29	0.0027	0.0476
7 days	twi_ss.30121.1|g.40695	-3.34	3.58	15.28	0.0027	0.0476
7 days	twi_ss.30158.1|g.40726	0.67	7.49	15.28	0.0027	0.0476
7 days	twi_ss.927c.1|g.1620	0.56	6.38	15.26	0.0027	0.0478

7 days	twi_ss.25970.2|g.34285	3.44	4.49	15.23	0.0027	0.0480
7 days	twi_ss.5428.1|g.8196	-4.88	2.29	17.53	0.0027	0.0480
7 days	twi_ss.7066.1|g.10124	-5.57	2.84	17.54	0.0027	0.0480
7 days	twi_ss.11596.1|g.15229	-3.63	3.62	15.23	0.0027	0.0480
7 days	twi_ss.16895.1|g.21531	1.41	6.26	15.23	0.0027	0.0480
7 days	twi_ss.27976.3|g.37224	4.32	1.91	17.52	0.0027	0.0480
7 days	twi_ss.11616.1|g.15418	-0.87	6.39	15.21	0.0027	0.0480
7 days	twi_ss.20271b.1|g.26055	1.09	5.85	15.21	0.0027	0.0480
7 days	twi_ss.20296.3|g.26134	1.38	6.26	15.21	0.0027	0.0480
7 days	twi_ss.30309.1|g.40994	0.79	5.72	15.21	0.0027	0.0480
7 days	twi_ss.7623.1|g.10683	2.03	5.24	15.21	0.0028	0.0480
7 days	twi_ss.23867.1|g.31016	0.61	7.09	15.20	0.0028	0.0481
7 days	twi_ss.22694.1|g.29310	-1.00	5.93	15.19	0.0028	0.0482
7 days	twi_ss.10273a.4|g.13876	1.49	5.60	15.18	0.0028	0.0482
7 days	twi_ss.29491.1|g.39721	-1.52	3.96	15.18	0.0028	0.0482
7 days	twi_ss.28802.1|g.38598	-1.52	4.26	15.15	0.0028	0.0485
7 days	twi_ss.12211.2|g.16254	1.44	5.84	15.11	0.0028	0.0487
7 days	twi_ss.18881a.6|g.24014	-1.98	5.22	17.38	0.0028	0.0487
7 days	twi_ss.21114a.5|g.27293	-7.48	3.83	15.12	0.0028	0.0487
7 days	twi_ss.25328.1|g.33337	0.76	8.81	15.12	0.0028	0.0487
7 days	twi_ss.26920.1|g.35735	0.67	6.17	15.11	0.0028	0.0487
7 days	twi_ss.3615.1|g.5979	0.49	7.01	15.11	0.0028	0.0487
7 days	twi_ss.4871b.1|g.7455	0.70	6.56	15.13	0.0028	0.0487
7 days	twi_ss.18626b.1|g.23809	-2.83	4.14	15.10	0.0028	0.0487
7 days	twi_ss.14702.1|g.19158	3.36	4.08	15.09	0.0028	0.0488
7 days	twi_ss.21192.1|g.27431	0.89	5.38	15.09	0.0028	0.0488
7 days	twi_ss.27680.1|g.36876	1.03	5.35	15.08	0.0028	0.0489
7 days	twi_ss.7075.1|g.10131	-2.48	4.19	15.07	0.0028	0.0490
7 days	twi_ss.1859.1|g.3292	1.60	4.06	15.05	0.0028	0.0491
7 days	twi_ss.9619.1|g.12899	1.58	4.40	15.05	0.0028	0.0492
7 days	twi_ss.18095.1|g.22988	-5.54	2.46	17.29	0.0029	0.0492
7 days	twi_ss.28559.1|g.38253	-3.04	4.09	15.04	0.0028	0.0492
7 days	twi_ss.31412.1|g.42750	-2.93	3.73	15.04	0.0029	0.0492

7 days	twi_ss.13604.2|g.17956	0.66	8.04	15.02	0.0029	0.0493
7 days	twi_ss.12142.1|g.16071	1.09	8.13	14.99	0.0029	0.0494
7 days	twi_ss.12241.1|g.16284	1.83	4.34	15.00	0.0029	0.0494
7 days	twi_ss.12950.1|g.17138	-5.35	2.42	17.24	0.0029	0.0494
7 days	twi_ss.15542.1|g.20120	-1.34	5.03	14.99	0.0029	0.0494
7 days	twi_ss.21978.1|g.28482	-2.82	3.45	14.99	0.0029	0.0494
7 days	twi_ss.2339.1|g.4197	-7.32	4.18	15.00	0.0029	0.0494
7 days	twi_ss.25045a.1|g.32875	-1.09	6.66	14.99	0.0029	0.0494
7 days	twi_ss.814.1|g.1385	-2.23	4.97	15.00	0.0029	0.0494
7 days	twi_ss.9828.1|g.13157	2.46	3.30	15.01	0.0029	0.0494
7 days	twi_ss.3627.1|g.5997	2.73	3.88	14.97	0.0029	0.0495
7 days	twi_ss.23793.1|g.30757	-2.33	4.17	14.93	0.0029	0.0500
7 days	twi_ss.4815a.1|g.7394	0.53	7.58	14.93	0.0029	0.0500
7 days	twi_ss.15223.1|g.19805	-3.96	3.16	14.91	0.0029	0.0501
7 days	twi_ss.18174.1|g.23103	1.36	5.45	14.91	0.0029	0.0501
7 days	twi_ss.2230.1|g.3995	1.12	5.06	14.91	0.0029	0.0501
7 days	twi_ss.6556.1|g.9581	-1.36	4.44	14.90	0.0029	0.0502
7 days	twi_ss.2800b.1|g.5041	0.91	7.73	14.89	0.0029	0.0503
7 days	twi_ss.21306.2|g.27550	5.96	2.45	35.31	0.0029	0.0503
7 days	twi_ss.22044.1|g.28559	-3.00	3.74	14.87	0.0030	0.0503
7 days	twi_ss.24518.1|g.32102	0.99	5.98	14.88	0.0030	0.0503
7 days	twi_ss.2092.1|g.3745	2.00	3.22	14.85	0.0030	0.0506
7 days	twi_ss.25757.7|g.33980	-5.32	3.87	14.85	0.0030	0.0506
21 days	twi_ss.4874.1|g.7384	-6.20	7.60	817.60	3.14E-11	0.0000
21 days	twi_ss.800.2|g.1407	-5.20	6.66	722.55	5.93E-11	0.0000
21 days	twi_ss.20755a.2|g.26865	-3.66	8.81	606.97	1.45E-10	0.0000
21 days	twi_ss.1219.1|g.2104	-4.48	8.02	552.77	2.34E-10	0.0000
21 days	twi_ss.28038b.1|g.37328	3.07	8.70	437.98	7.67E-10	0.0000
21 days	twi_ss.31790b.1|g.43276	-6.62	7.71	450.72	6.63E-10	0.0000
21 days	twi_ss.28936.1|g.38782	-7.19	8.53	408.63	1.09E-09	0.0000
21 days	twi_ss.4874.2|g.7378	-2.70	8.80	415.73	1.00E-09	0.0000
21 days	twi_ss.22567.1|g.29150	-5.43	6.27	388.70	1.41E-09	0.0000
21 days	twi_ss.5201.1|g.7826	-4.18	8.11	395.54	1.29E-09	0.0000

21 days	twi_ss.1226.1|g.2106	-4.32	7.40	372.04	1.76E-09	0.0000
21 days	twi_ss.25299a.1|g.33316	-4.89	7.03	363.09	1.99E-09	0.0000
21 days	twi_ss.4034.1|g.6451	-5.40	6.78	368.25	1.85E-09	0.0000
21 days	twi_ss.25693a.1|g.33896	5.42	11.28	332.17	3.12E-09	0.0000
21 days	twi_ss.11722.2|g.15540	6.97	9.40	322.71	3.61E-09	0.0000
21 days	twi_ss.12318.1|g.16442	-7.28	6.49	323.59	3.56E-09	0.0000
21 days	twi_ss.14278.1|g.18615	6.35	9.20	301.49	5.09E-09	0.0000
21 days	twi_ss.14730.1|g.19254	2.79	6.87	303.12	4.95E-09	0.0000
21 days	twi_ss.20735.1|g.26813	8.87	9.95	302.46	5.01E-09	0.0000
21 days	twi_ss.24166.1|g.31414	3.08	9.99	307.99	4.57E-09	0.0000
21 days	twi_ss.3712.1|g.6096	4.96	10.33	303.57	4.92E-09	0.0000
21 days	twi_ss.5820.1|g.8724	-5.11	7.97	303.79	4.90E-09	0.0000
21 days	twi_ss.2376.1|g.4266	3.61	6.29	296.95	5.49E-09	0.0000
21 days	twi_ss.2517.1|g.4486	-4.63	6.72	289.09	6.29E-09	0.0000
21 days	twi_ss.5643.1|g.8453	5.45	10.41	287.48	6.47E-09	0.0000
21 days	twi_ss.13698.1|g.18022	2.44	7.15	282.65	7.04E-09	0.0000
21 days	twi_ss.12823b.1|g.16930	-8.40	4.65	275.53	8.01E-09	0.0000
21 days	twi_ss.25693b.2|g.33886	4.59	9.40	279.05	7.51E-09	0.0000
21 days	twi_ss.9362.1|g.12634	-9.89	8.80	277.19	7.77E-09	0.0000
21 days	twi_ss.24532a.1|g.32203	-2.73	7.04	272.18	8.52E-09	0.0000
21 days	twi_ss.11312.1|g.14924	3.39	7.86	259.27	1.09E-08	0.0000
21 days	twi_ss.8873.1|g.12071	5.39	9.97	246.12	1.41E-08	0.0000
21 days	twi_ss.22049a.2|g.28532	-3.29	8.29	237.30	1.69E-08	0.0000
21 days	twi_ss.24809.1|g.32606	-6.24	8.19	236.21	1.73E-08	0.0000
21 days	twi_ss.3513.1|g.5884	-2.67	7.03	237.87	1.67E-08	0.0000
21 days	twi_ss.120.2|g.195	2.35	6.95	228.92	2.03E-08	0.0000
21 days	twi_ss.13838.1|g.18145	2.87	9.41	222.61	2.33E-08	0.0000
21 days	twi_ss.19960a.4|g.25706	7.41	4.70	219.63	2.49E-08	0.0000
21 days	twi_ss.7586.1|g.10632	2.27	8.25	220.83	2.43E-08	0.0000
21 days	twi_ss.8875.1|g.12104	4.41	6.38	220.69	2.43E-08	0.0000
21 days	twi_ss.19635.1|g.25202	1.67	8.15	217.77	2.60E-08	0.0000
21 days	twi_ss.20513.1|g.26432	-5.30	4.89	212.68	2.93E-08	0.0000
21 days	twi_ss.25299b.2|g.33311	4.49	9.98	204.55	3.55E-08	0.0000

21 days	twi_ss.4463.1|g.6986	3.35	10.14	200.54	3.92E-08	0.0000
21 days	twi_ss.4881.1|g.7484	-4.65	8.71	197.61	4.22E-08	0.0000
21 days	twi_ss.18478.1|g.23540	-5.13	7.38	195.13	4.49E-08	0.0000
21 days	twi_ss.22010.1|g.28502	2.40	6.59	194.50	4.56E-08	0.0000
21 days	twi_ss.20795.1|g.26925	10.04	10.91	191.15	4.97E-08	0.0000
21 days	twi_ss.2045.1|g.3673	-3.69	5.26	186.71	5.59E-08	0.0000
21 days	twi_ss.10965.1|g.14538	3.50	12.07	183.30	6.12E-08	0.0000
21 days	twi_ss.21114b.1|g.27305	-3.70	6.39	181.67	6.39E-08	0.0000
21 days	twi_ss.23402.1|g.30075	3.20	6.81	181.41	6.44E-08	0.0000
21 days	twi_ss.978.1|g.1784	6.64	10.48	180.35	6.63E-08	0.0000
21 days	twi_ss.11705.1|g.15516	-1.89	8.15	176.71	7.33E-08	0.0000
21 days	twi_ss.3603a.1|g.5981	-3.22	5.70	176.50	7.37E-08	0.0000
21 days	twi_ss.15729.1|g.20309	2.80	9.44	173.14	8.11E-08	0.0000
21 days	twi_ss.3000.1|g.5329	1.61	6.76	174.33	7.84E-08	0.0000
21 days	twi_ss.5069.1|g.7717	-5.29	7.16	173.31	8.07E-08	0.0000
21 days	twi_ss.647.1|g.1197	1.43	7.65	171.84	8.41E-08	0.0000
21 days	twi_ss.31789.1|g.43293	-7.77	7.55	170.13	8.84E-08	0.0000
21 days	twi_ss.19594.1|g.25172	-2.90	6.66	166.02	9.97E-08	0.0000
21 days	twi_ss.4710.1|g.7263	-5.06	5.16	166.45	9.84E-08	0.0000
21 days	twi_ss.19960a.2|g.25708	-6.50	4.09	159.41	1.22E-07	0.0001
21 days	twi_ss.21205.1|g.27437	4.33	6.27	158.74	1.24E-07	0.0001
21 days	twi_ss.16184.1|g.20768	-9.67	5.70	259.26	1.31E-07	0.0001
21 days	twi_ss.28647.1|g.38357	1.82	8.32	157.30	1.30E-07	0.0001
21 days	twi_ss.5609.1|g.8381	1.30	8.78	158.06	1.27E-07	0.0001
21 days	twi_ss.13405.1|g.17801	-6.19	8.97	156.40	1.34E-07	0.0001
21 days	twi_ss.6620.1|g.9636	2.62	9.55	151.27	1.57E-07	0.0001
21 days	twi_ss.13145.1|g.17465	2.81	7.70	149.76	1.65E-07	0.0001
21 days	twi_ss.4286.1|g.6756	2.11	10.01	150.06	1.64E-07	0.0001
21 days	twi_ss.6495.1|g.9528	2.50	6.55	148.64	1.72E-07	0.0001
21 days	twi_ss.9907.1|g.13240	3.13	7.26	146.21	1.86E-07	0.0001
21 days	twi_ss.12661.1|g.16777	-5.09	5.53	145.44	1.91E-07	0.0001
21 days	twi_ss.24296.1|g.31742	1.99	8.01	143.24	2.05E-07	0.0001
21 days	twi_ss.2611.1|g.4635	3.51	8.33	143.16	2.06E-07	0.0001

21 days	twi_ss.7206.1|g.10257	2.83	6.36	143.04	2.07E-07	0.0001
21 days	twi_ss.800.1|g.1410	-2.81	7.58	142.75	2.09E-07	0.0001
21 days	twi_ss.8361b.2|g.11480	2.72	7.66	143.87	2.01E-07	0.0001
21 days	twi_ss.1191.1|g.2086	1.87	6.81	141.60	2.17E-07	0.0001
21 days	twi_ss.8099.3|g.11155	-2.58	6.38	141.96	2.15E-07	0.0001
21 days	twi_ss.22556.1|g.29135	-11.02	7.02	224.24	2.36E-07	0.0001
21 days	twi_ss.26117a.1|g.34555	2.91	5.20	138.98	2.38E-07	0.0001
21 days	twi_ss.27768.1|g.37001	5.25	6.17	138.84	2.39E-07	0.0001
21 days	twi_ss.28657.1|g.38361	2.01	7.33	138.22	2.44E-07	0.0001
21 days	twi_ss.18881a.22|g.23994	-3.40	6.71	136.52	2.60E-07	0.0001
21 days	twi_ss.2510.1|g.4542	-2.17	7.96	137.19	2.54E-07	0.0001
21 days	twi_ss.26616.1|g.35296	-4.79	7.34	137.49	2.51E-07	0.0001
21 days	twi_ss.31453.1|g.42823	-4.85	7.75	136.54	2.59E-07	0.0001
21 days	twi_ss.9313.4|g.12557	-12.32	8.39	218.89	2.60E-07	0.0001
21 days	twi_ss.15378.1|g.19918	-2.38	6.14	135.61	2.68E-07	0.0001
21 days	twi_ss.2969.1|g.5263	-1.99	8.81	133.50	2.90E-07	0.0001
21 days	twi_ss.18952b.3|g.24232	-3.57	5.38	129.42	3.37E-07	0.0001
21 days	twi_ss.29475.1|g.39689	4.34	7.92	130.01	3.29E-07	0.0001
21 days	twi_ss.3533.1|g.5908	6.63	4.04	129.20	3.39E-07	0.0001
21 days	twi_ss.3533.2|g.5904	6.63	4.04	129.20	3.39E-07	0.0001
21 days	twi_ss.3533.3|g.5912	6.63	4.04	129.20	3.39E-07	0.0001
21 days	twi_ss.3533.4|g.5906	6.63	4.04	129.20	3.39E-07	0.0001
21 days	twi_ss.6037.1|g.8896	2.98	5.97	128.37	3.50E-07	0.0001
21 days	twi_ss.21790.1|g.28289	-2.36	6.43	127.75	3.58E-07	0.0001
21 days	twi_ss.25105.9|g.32925	-7.26	3.41	127.83	3.57E-07	0.0001
21 days	twi_ss.3335.2|g.5740	-2.50	7.29	127.39	3.63E-07	0.0001
21 days	twi_ss.24294.1|g.31738	1.84	7.66	126.46	3.76E-07	0.0001
21 days	twi_ss.24697.1|g.32404	2.78	6.36	126.82	3.71E-07	0.0001
21 days	twi_ss.2869.2|g.5156	3.34	7.03	126.71	3.73E-07	0.0001
21 days	twi_ss.2830.1|g.5082	1.57	9.93	124.47	4.06E-07	0.0001
21 days	twi_ss.5288.1|g.7947	-2.24	6.75	124.33	4.09E-07	0.0001
21 days	twi_ss.459c.1|g.826	-1.76	8.81	124.07	4.13E-07	0.0001
21 days	twi_ss.26446.1|g.35014	1.94	9.82	123.73	4.18E-07	0.0001

21 days	twi_ss.1469.1|g.2686	-3.06	4.99	123.38	4.24E-07	0.0001
21 days	twi_ss.16547.1|g.21223	-2.97	6.36	123.23	4.27E-07	0.0001
21 days	twi_ss.26358.1|g.34798	7.30	9.69	122.98	4.31E-07	0.0001
21 days	twi_ss.2375a.2|g.4144	1.78	6.79	122.64	4.37E-07	0.0001
21 days	twi_ss.2067.1|g.3656	-3.39	5.41	121.87	4.50E-07	0.0001
21 days	twi_ss.2869.1|g.5155	5.80	5.94	122.25	4.43E-07	0.0001
21 days	twi_ss.6045.1|g.8903	2.52	7.02	122.07	4.46E-07	0.0001
21 days	twi_ss.556.2|g.1009	-4.94	6.22	121.05	4.65E-07	0.0001
21 days	twi_ss.19329a.2|g.24785	2.43	8.63	120.55	4.74E-07	0.0001
21 days	twi_ss.21060a.1|g.27171	1.47	7.92	120.35	4.78E-07	0.0001
21 days	twi_ss.24106.1|g.31280	4.23	6.28	120.44	4.76E-07	0.0001
21 days	twi_ss.25703.1|g.33871	-7.10	6.17	119.80	4.89E-07	0.0001
21 days	twi_ss.30657b.1|g.41506	-5.26	5.94	119.84	4.88E-07	0.0001
21 days	twi_ss.7998.1|g.11044	-4.57	7.51	119.88	4.87E-07	0.0001
21 days	twi_ss.10882.1|g.14512	6.01	9.11	118.53	5.14E-07	0.0001
21 days	twi_ss.30338.1|g.41031	-2.82	6.34	118.24	5.21E-07	0.0001
21 days	twi_ss.23257.1|g.29910	-4.27	4.82	117.23	5.43E-07	0.0001
21 days	twi_ss.5474.4|g.8258	2.08	8.15	117.15	5.44E-07	0.0001
21 days	twi_ss.28010.1|g.37326	2.82	5.77	116.64	5.56E-07	0.0001
21 days	twi_ss.30724.1|g.41642	4.17	7.69	116.33	5.63E-07	0.0001
21 days	twi_ss.18522.1|g.23566	-11.44	7.52	177.76	6.04E-07	0.0001
21 days	twi_ss.5155.1|g.7788	1.88	6.27	113.40	6.36E-07	0.0001
21 days	twi_ss.27063.1|g.35999	7.75	5.95	175.16	6.41E-07	0.0001
21 days	twi_ss.12226.1|g.16336	4.86	6.55	112.27	6.68E-07	0.0002
21 days	twi_ss.22588b.1|g.29167	-3.76	5.65	112.40	6.64E-07	0.0002
21 days	twi_ss.30512b.1|g.41315	-4.68	5.12	112.37	6.65E-07	0.0002
21 days	twi_ss.12625.1|g.16729	1.19	9.41	111.84	6.80E-07	0.0002
21 days	twi_ss.623.1|g.1129	-5.12	6.36	112.01	6.75E-07	0.0002
21 days	twi_ss.18906.1|g.24170	1.09	7.78	111.67	6.85E-07	0.0002
21 days	twi_ss.25457.1|g.33489	2.71	8.38	110.69	7.15E-07	0.0002
21 days	twi_ss.28616.1|g.38309	2.83	6.73	110.83	7.11E-07	0.0002
21 days	twi_ss.9089.1|g.12267	-7.04	7.43	110.86	7.10E-07	0.0002
21 days	twi_ss.21635b.1|g.28037	1.25	9.47	110.08	7.34E-07	0.0002

21 days	twi_ss.6877.1|g.9936	4.27	5.24	110.08	7.34E-07	0.0002
21 days	twi_ss.16815.1|g.21469	-2.46	5.95	108.30	7.93E-07	0.0002
21 days	twi_ss.2053.1|g.3716	-5.03	4.96	108.38	7.91E-07	0.0002
21 days	twi_ss.26717.1|g.35464	-2.59	5.89	108.52	7.86E-07	0.0002
21 days	twi_ss.12243.1|g.16329	2.67	6.81	107.28	8.30E-07	0.0002
21 days	twi_ss.6091.1|g.8963	1.86	7.70	107.06	8.38E-07	0.0002
21 days	twi_ss.21105.9|g.27276	9.58	6.72	334.88	9.04E-07	0.0002
21 days	twi_ss.26719.1|g.35434	-4.35	8.58	105.43	9.02E-07	0.0002
21 days	twi_ss.19875.1|g.25622	1.60	8.29	104.17	9.55E-07	0.0002
21 days	twi_ss.20391.1|g.26269	-10.37	6.60	157.28	9.89E-07	0.0002
21 days	twi_ss.7208.2|g.10260	4.29	5.93	103.21	9.98E-07	0.0002
21 days	twi_ss.22858.1|g.29499	-4.01	6.39	101.55	1.08E-06	0.0002
21 days	twi_ss.8912.1|g.12098	-2.21	6.22	100.81	1.12E-06	0.0002
21 days	twi_ss.11488c.5|g.15145	1.75	5.74	100.60	1.13E-06	0.0002
21 days	twi_ss.27219.1|g.36158	2.41	10.24	100.40	1.14E-06	0.0002
21 days	twi_ss.9041.1|g.12212	-1.77	8.47	100.32	1.14E-06	0.0002
21 days	twi_ss.10495.1|g.14129	2.04	9.39	98.26	1.26E-06	0.0002
21 days	twi_ss.23557.1|g.30254	-3.15	5.37	98.26	1.26E-06	0.0002
21 days	twi_ss.19570.5|g.25098	7.42	4.87	98.04	1.27E-06	0.0002
21 days	twi_ss.31583b.5|g.43005	-8.36	4.85	147.21	1.29E-06	0.0002
21 days	twi_ss.1977.1|g.3449	1.15	9.41	97.31	1.32E-06	0.0002
21 days	twi_ss.9775.1|g.13150	5.18	5.03	97.22	1.33E-06	0.0002
21 days	twi_ss.10440.1|g.14080	1.12	8.43	97.01	1.34E-06	0.0002
21 days	twi_ss.4179.1|g.6597	4.45	6.34	96.06	1.40E-06	0.0003
21 days	twi_ss.22201a.1|g.28770	-2.43	6.17	95.20	1.47E-06	0.0003
21 days	twi_ss.28736.1|g.38496	-1.53	6.41	95.18	1.47E-06	0.0003
21 days	twi_ss.25566.1|g.33668	-4.65	4.98	94.20	1.54E-06	0.0003
21 days	twi_ss.15379.1|g.19919	-3.35	5.86	93.92	1.56E-06	0.0003
21 days	twi_ss.20450.1|g.26352	1.48	7.40	93.88	1.57E-06	0.0003
21 days	twi_ss.22907.1|g.29571	-2.21	8.99	93.59	1.59E-06	0.0003
21 days	twi_ss.26686.1|g.35380	-2.26	5.54	93.74	1.58E-06	0.0003
21 days	twi_ss.28760.1|g.38522	1.95	7.34	93.70	1.58E-06	0.0003
21 days	twi_ss.3782.2|g.6191	1.42	7.62	93.39	1.60E-06	0.0003

21 days	twi_ss.9393.1|g.12653	1.81	6.60	93.41	1.60E-06	0.0003
21 days	twi_ss.10559.1|g.14213	-3.74	6.75	93.27	1.61E-06	0.0003
21 days	twi_ss.30972.1|g.41798	-2.85	8.77	92.90	1.64E-06	0.0003
21 days	twi_ss.10250.1|g.13755	1.93	6.42	92.78	1.65E-06	0.0003
21 days	twi_ss.28295a.1|g.37846	-1.60	7.50	92.34	1.69E-06	0.0003
21 days	twi_ss.29768.1|g.40059	-2.78	9.81	92.35	1.69E-06	0.0003
21 days	twi_ss.21214.1|g.27436	2.21	7.28	92.11	1.71E-06	0.0003
21 days	twi_ss.31673.1|g.43122	2.48	8.01	92.20	1.70E-06	0.0003
21 days	twi_ss.22947.1|g.29606	-3.61	6.59	91.42	1.77E-06	0.0003
21 days	twi_ss.13619b.2|g.17975	1.48	6.81	90.76	1.84E-06	0.0003
21 days	twi_ss.160.1|g.288	-3.78	5.76	89.98	1.91E-06	0.0003
21 days	twi_ss.20282.1|g.26105	1.48	7.34	89.92	1.92E-06	0.0003
21 days	twi_ss.28922.1|g.38780	-9.37	5.38	132.38	1.97E-06	0.0003
21 days	twi_ss.10837.3|g.14465	2.63	6.52	89.29	1.98E-06	0.0003
21 days	twi_ss.30222b.8|g.40809	-5.63	4.72	88.29	2.09E-06	0.0003
21 days	twi_ss.28816.2|g.38575	3.53	5.27	87.93	2.13E-06	0.0003
21 days	twi_ss.30705.1|g.41592	-6.09	4.80	87.96	2.13E-06	0.0003
21 days	twi_ss.2312b.3|g.4065	-6.44	3.27	87.82	2.14E-06	0.0003
21 days	twi_ss.28308.1|g.37864	-2.85	5.79	87.67	2.16E-06	0.0003
21 days	twi_ss.13409.1|g.17726	-3.52	5.79	87.01	2.24E-06	0.0003
21 days	twi_ss.13842.1|g.18147	-4.63	10.84	87.31	2.20E-06	0.0003
21 days	twi_ss.16353.1|g.20942	-11.02	6.87	128.82	2.19E-06	0.0003
21 days	twi_ss.25372.1|g.33400	-3.90	6.66	86.99	2.24E-06	0.0003
21 days	twi_ss.26069.1|g.34483	-3.09	5.50	87.20	2.22E-06	0.0003
21 days	twi_ss.29765.1|g.40103	-2.05	10.04	86.96	2.24E-06	0.0003
21 days	twi_ss.297.1|g.596	0.92	9.12	86.86	2.26E-06	0.0003
21 days	twi_ss.4017.1|g.6439	-4.21	5.71	86.65	2.28E-06	0.0003
21 days	twi_ss.30312.8|g.40958	1.80	6.50	86.14	2.35E-06	0.0004
21 days	twi_ss.22497.1|g.29072	1.93	9.61	86.00	2.36E-06	0.0004
21 days	twi_ss.1765.1|g.3114	1.80	9.24	84.80	2.52E-06	0.0004
21 days	twi_ss.17845.1|g.22746	-3.50	7.98	84.89	2.51E-06	0.0004
21 days	twi_ss.21316b.3|g.27576	2.06	7.41	84.62	2.55E-06	0.0004
21 days	twi_ss.21610.1|g.28014	2.89	5.82	84.71	2.54E-06	0.0004

21 days	twi_ss.22743.11|g.29088	-3.25	5.56	85.03	2.49E-06	0.0004
21 days	twi_ss.2321.1|g.4060	-2.72	5.52	84.91	2.51E-06	0.0004
21 days	twi_ss.24236.1|g.31566	-11.02	11.20	84.67	2.54E-06	0.0004
21 days	twi_ss.30935.1|g.41933	0.94	8.71	85.22	2.47E-06	0.0004
21 days	twi_ss.16484.1|g.21069	-2.02	6.53	84.32	2.59E-06	0.0004
21 days	twi_ss.26297.1|g.34808	-6.93	5.79	84.32	2.59E-06	0.0004
21 days	twi_ss.27364.1|g.36414	1.30	9.50	84.17	2.62E-06	0.0004
21 days	twi_ss.10136.2|g.13581	-2.69	7.86	83.95	2.65E-06	0.0004
21 days	twi_ss.7122a.1|g.10138	1.76	7.65	84.03	2.64E-06	0.0004
21 days	twi_ss.724b.1|g.1263	1.06	9.03	83.91	2.65E-06	0.0004
21 days	twi_ss.6452.1|g.9489	2.95	9.20	83.17	2.76E-06	0.0004
21 days	twi_ss.20951.1|g.27056	1.74	5.77	82.98	2.79E-06	0.0004
21 days	twi_ss.7010.1|g.10061	-3.47	5.61	82.83	2.82E-06	0.0004
21 days	twi_ss.9465.1|g.12697	-3.67	4.45	82.90	2.81E-06	0.0004
21 days	twi_ss.29232.1|g.39206	-3.53	5.59	82.59	2.86E-06	0.0004
21 days	twi_ss.17187a.1|g.21951	-3.51	5.34	82.31	2.90E-06	0.0004
21 days	twi_ss.28414.1|g.38016	2.97	6.71	82.41	2.89E-06	0.0004
21 days	twi_ss.31729.1|g.43227	3.95	4.47	82.24	2.91E-06	0.0004
21 days	twi_ss.9647.1|g.12933	1.39	7.92	82.26	2.91E-06	0.0004
21 days	twi_ss.11366.1|g.15001	2.17	9.43	81.74	3.00E-06	0.0004
21 days	twi_ss.1152.1|g.2029	1.23	7.01	81.82	2.98E-06	0.0004
21 days	twi_ss.13436.1|g.17773	2.29	7.23	81.79	2.99E-06	0.0004
21 days	twi_ss.24847.1|g.32681	1.89	5.83	81.71	3.00E-06	0.0004
21 days	twi_ss.19933a.2|g.25657	-2.81	6.53	81.40	3.06E-06	0.0004
21 days	twi_ss.31184.1|g.42387	1.01	8.98	81.01	3.13E-06	0.0004
21 days	twi_ss.4163.1|g.6586	-4.07	4.87	80.89	3.15E-06	0.0004
21 days	twi_ss.19023.1|g.24422	3.22	6.51	80.80	3.16E-06	0.0004
21 days	twi_ss.6040.1|g.8889	1.83	9.34	80.60	3.20E-06	0.0004
21 days	twi_ss.3793.1|g.6196	-3.49	4.89	80.48	3.22E-06	0.0004
21 days	twi_ss.17561.1|g.22399	-1.15	9.15	80.29	3.26E-06	0.0004
21 days	twi_ss.16258.1|g.20833	-7.10	5.05	79.84	3.34E-06	0.0004
21 days	twi_ss.4654.1|g.7188	3.55	6.57	79.81	3.35E-06	0.0004
21 days	twi_ss.30706.1|g.41609	-5.03	5.78	79.36	3.44E-06	0.0004

21 days	twi_ss.19895.1|g.25639	1.55	8.07	79.23	3.47E-06	0.0004
21 days	twi_ss.670.1|g.1159	-2.43	5.90	79.12	3.49E-06	0.0004
21 days	twi_ss.11664.1|g.15469	2.99	6.76	78.72	3.57E-06	0.0004
21 days	twi_ss.25312.3|g.33264	5.82	7.88	78.69	3.58E-06	0.0004
21 days	twi_ss.431.1|g.791	-2.53	5.28	78.53	3.61E-06	0.0004
21 days	twi_ss.3922a.1|g.6353	1.56	6.88	78.29	3.66E-06	0.0005
21 days	twi_ss.12047.1|g.15946	1.97	6.60	78.16	3.69E-06	0.0005
21 days	twi_ss.27046.1|g.35942	1.26	6.75	78.05	3.72E-06	0.0005
21 days	twi_ss.3152.1|g.5557	2.71	7.62	77.96	3.74E-06	0.0005
21 days	twi_ss.13601.1|g.17943	-3.05	6.48	77.59	3.82E-06	0.0005
21 days	twi_ss.2764.1|g.4998	1.15	8.88	77.34	3.88E-06	0.0005
21 days	twi_ss.10945.1|g.14555	5.13	6.62	77.20	3.91E-06	0.0005
21 days	twi_ss.29759b.1|g.40113	3.17	6.10	77.19	3.91E-06	0.0005
21 days	twi_ss.25312.2|g.33268	-5.50	4.57	77.02	3.95E-06	0.0005
21 days	twi_ss.4716a.1|g.7265	1.15	6.46	76.65	4.04E-06	0.0005
21 days	twi_ss.7080.1|g.10166	2.09	6.64	76.62	4.05E-06	0.0005
21 days	twi_ss.8570.1|g.11715	-1.57	7.71	76.49	4.08E-06	0.0005
21 days	twi_ss.12889.1|g.17077	-4.11	4.65	76.38	4.11E-06	0.0005
21 days	twi_ss.2678.1|g.4844	1.10	9.62	76.16	4.16E-06	0.0005
21 days	twi_ss.28991.1|g.38906	-7.19	3.31	76.21	4.15E-06	0.0005
21 days	twi_ss.11279.1|g.14907	-5.25	4.16	75.96	4.21E-06	0.0005
21 days	twi_ss.824.2|g.1435	-2.77	5.36	76.00	4.20E-06	0.0005
21 days	twi_ss.17802.1|g.22700	7.03	10.31	75.80	4.26E-06	0.0005
21 days	twi_ss.12162.1|g.16141	1.09	7.74	75.55	4.32E-06	0.0005
21 days	twi_ss.22219.1|g.28723	2.22	6.13	75.56	4.32E-06	0.0005
21 days	twi_ss.23514.1|g.30170	4.10	6.85	75.43	4.35E-06	0.0005
21 days	twi_ss.26160.1|g.34649	-3.94	6.24	75.36	4.37E-06	0.0005
21 days	twi_ss.7665.9|g.10738	-3.54	6.41	75.35	4.37E-06	0.0005
21 days	twi_ss.1871.1|g.3293	2.81	8.47	74.81	4.52E-06	0.0005
21 days	twi_ss.8788.1|g.11924	1.60	6.58	74.53	4.60E-06	0.0005
21 days	twi_ss.9756.1|g.13098	-4.31	4.88	74.39	4.64E-06	0.0005
21 days	twi_ss.31795.1|g.43313	-5.21	4.62	74.28	4.67E-06	0.0005
21 days	twi_ss.13863.1|g.18169	1.53	8.89	73.92	4.78E-06	0.0005

21 days	twi_ss.31059.1|g.42230	-5.35	5.50	73.88	4.79E-06	0.0005
21 days	twi_ss.19882.1|g.25643	2.31	6.84	73.52	4.90E-06	0.0005
21 days	twi_ss.25079.1|g.32949	-5.89	5.84	73.35	4.95E-06	0.0005
21 days	twi_ss.23516.1|g.30163	2.54	7.31	73.18	5.00E-06	0.0005
21 days	twi_ss.17733.1|g.22605	-4.51	4.23	72.98	5.07E-06	0.0006
21 days	twi_ss.22264.1|g.28821	-5.92	5.59	72.90	5.10E-06	0.0006
21 days	twi_ss.1444.1|g.2597	1.21	8.64	72.80	5.13E-06	0.0006
21 days	twi_ss.20094.1|g.25861	-7.74	5.74	72.74	5.15E-06	0.0006
21 days	twi_ss.21611.1|g.28009	1.44	7.71	72.66	5.17E-06	0.0006
21 days	twi_ss.478.1|g.870	-5.84	4.63	72.30	5.29E-06	0.0006
21 days	twi_ss.58.1|g.80	-2.79	7.27	72.07	5.37E-06	0.0006
21 days	twi_ss.1579a.1|g.2780	2.98	6.85	71.90	5.43E-06	0.0006
21 days	twi_ss.25128.1|g.33004	-2.25	5.38	71.52	5.56E-06	0.0006
21 days	twi_ss.8806.1|g.11996	-4.03	7.04	71.50	5.57E-06	0.0006
21 days	twi_ss.2019.2|g.3587	1.03	9.70	71.39	5.61E-06	0.0006
21 days	twi_ss.28156a.2|g.37510	-8.90	9.33	71.40	5.61E-06	0.0006
21 days	twi_ss.14752.1|g.19217	1.55	6.03	71.13	5.70E-06	0.0006
21 days	twi_ss.10122.1|g.13573	-11.50	11.62	71.06	5.73E-06	0.0006
21 days	twi_ss.20961a.1|g.27085	2.18	6.27	70.84	5.81E-06	0.0006
21 days	twi_ss.21211.1|g.27438	2.67	5.60	70.68	5.87E-06	0.0006
21 days	twi_ss.235.6|g.443	2.40	6.70	70.62	5.90E-06	0.0006
21 days	twi_ss.1027.1|g.1857	-2.91	7.81	70.16	6.07E-06	0.0006
21 days	twi_ss.1027.2|g.1854	-2.91	7.81	70.16	6.07E-06	0.0006
21 days	twi_ss.1625.1|g.2826	1.46	7.16	70.13	6.09E-06	0.0006
21 days	twi_ss.28938.1|g.38803	-4.85	4.93	70.19	6.06E-06	0.0006
21 days	twi_ss.29603.1|g.39875	0.93	9.46	70.11	6.10E-06	0.0006
21 days	twi_ss.6192.1|g.9196	-4.79	9.99	70.03	6.13E-06	0.0006
21 days	twi_ss.1968.1|g.3462	1.46	11.14	69.82	6.21E-06	0.0006
21 days	twi_ss.2594.1|g.4760	1.40	7.28	69.83	6.21E-06	0.0006
21 days	twi_ss.1972.1|g.3478	1.46	10.23	69.70	6.26E-06	0.0006
21 days	twi_ss.8953.1|g.12138	-3.00	5.87	69.63	6.29E-06	0.0006
21 days	twi_ss.24245.1|g.31625	-9.89	10.63	69.42	6.38E-06	0.0006
21 days	twi_ss.24378.1|g.31976	-2.73	6.72	69.42	6.38E-06	0.0006

21 days	twi_ss.6198.1|g.9204	-5.56	4.72	69.51	6.34E-06	0.0006
21 days	twi_ss.21348.1|g.27573	-2.83	5.71	69.23	6.46E-06	0.0006
21 days	twi_ss.11864.1|g.15756	1.84	6.14	69.02	6.55E-06	0.0006
21 days	twi_ss.19774.1|g.25443	1.03	8.09	69.01	6.55E-06	0.0006
21 days	twi_ss.21292.1|g.27529	-5.81	5.83	69.07	6.53E-06	0.0006
21 days	twi_ss.6621.1|g.9671	-3.28	5.37	69.06	6.53E-06	0.0006
21 days	twi_ss.26983.1|g.35830	-2.00	6.04	68.90	6.60E-06	0.0006
21 days	twi_ss.9661.1|g.12991	1.58	6.09	68.66	6.71E-06	0.0006
21 days	twi_ss.11143.1|g.14768	-3.02	9.31	68.24	6.90E-06	0.0007
21 days	twi_ss.27056.1|g.35956	0.94	9.37	68.23	6.90E-06	0.0007
21 days	twi_ss.11893a.1|g.15773	1.00	8.89	68.02	7.00E-06	0.0007
21 days	twi_ss.14780.1|g.19240	2.31	7.81	67.98	7.02E-06	0.0007
21 days	twi_ss.17091.1|g.21872	-2.07	5.36	67.93	7.04E-06	0.0007
21 days	twi_ss.2619.3|g.4738	1.77	7.74	67.92	7.05E-06	0.0007
21 days	twi_ss.27268a.1|g.36251	-1.96	7.46	67.90	7.06E-06	0.0007
21 days	twi_ss.2232.1|g.3943	1.82	6.78	67.81	7.10E-06	0.0007
21 days	twi_ss.13311.1|g.17670	-1.25	7.23	67.68	7.16E-06	0.0007
21 days	twi_ss.28666.1|g.38373	-5.26	5.56	67.66	7.17E-06	0.0007
21 days	twi_ss.11060.1|g.14695	1.45	7.03	67.40	7.30E-06	0.0007
21 days	twi_ss.18580.1|g.23702	7.12	7.68	94.91	7.29E-06	0.0007
21 days	twi_ss.329.1|g.608	1.51	8.45	67.20	7.40E-06	0.0007
21 days	twi_ss.4609.1|g.7115	-3.49	6.44	67.13	7.43E-06	0.0007
21 days	twi_ss.16612.1|g.21263	-1.92	6.64	66.79	7.61E-06	0.0007
21 days	twi_ss.18990a.1|g.24426	-1.18	7.72	66.75	7.63E-06	0.0007
21 days	twi_ss.23845.1|g.30845	-8.94	7.37	66.59	7.71E-06	0.0007
21 days	twi_ss.17303.1|g.22085	2.71	11.19	66.50	7.76E-06	0.0007
21 days	twi_ss.18881a.6|g.24014	-8.55	5.22	93.34	7.78E-06	0.0007
21 days	twi_ss.14116b.2|g.18421	3.28	4.82	66.39	7.82E-06	0.0007
21 days	twi_ss.3049.4|g.5406	-2.39	7.94	66.38	7.83E-06	0.0007
21 days	twi_ss.31289.1|g.42555	-5.03	6.26	66.02	8.02E-06	0.0007
21 days	twi_ss.5199.1|g.7825	-4.85	4.95	65.99	8.04E-06	0.0007
21 days	twi_ss.9920.1|g.13258	-6.07	2.70	66.03	8.02E-06	0.0007
21 days	twi_ss.8351.1|g.11463	1.38	6.89	65.69	8.20E-06	0.0007

21 days	twi_ss.9862.1|g.13198	1.03	7.60	65.71	8.19E-06	0.0007
21 days	twi_ss.1947.1|g.3447	-1.33	7.28	65.45	8.34E-06	0.0007
21 days	twi_ss.14325.1|g.18631	1.68	7.48	65.21	8.48E-06	0.0008
21 days	twi_ss.19469.1|g.24973	1.57	7.59	65.10	8.55E-06	0.0008
21 days	twi_ss.20287b.1|g.26122	1.17	7.52	64.75	8.76E-06	0.0008
21 days	twi_ss.27170b.2|g.36114	1.87	6.83	64.57	8.87E-06	0.0008
21 days	twi_ss.30117.2|g.40715	-2.60	4.87	64.60	8.85E-06	0.0008
21 days	twi_ss.17799.1|g.22676	-4.48	10.10	64.48	8.93E-06	0.0008
21 days	twi_ss.25737.1|g.33901	-1.49	6.95	64.36	9.00E-06	0.0008
21 days	twi_ss.2298.1|g.4053	-1.64	6.84	64.28	9.05E-06	0.0008
21 days	twi_ss.5306.1|g.7938	1.14	7.21	64.21	9.10E-06	0.0008
21 days	twi_ss.16604.1|g.21254	-3.31	7.50	64.00	9.24E-06	0.0008
21 days	twi_ss.5590a.7|g.8350	-8.89	5.11	157.92	9.46E-06	0.0008
21 days	twi_ss.21059.1|g.27172	1.43	7.39	63.53	9.55E-06	0.0008
21 days	twi_ss.28158a.3|g.37550	-6.43	2.83	63.55	9.54E-06	0.0008
21 days	twi_ss.291.1|g.638	1.19	7.46	63.53	9.55E-06	0.0008
21 days	twi_ss.29381.1|g.39493	1.00	6.98	63.35	9.67E-06	0.0008
21 days	twi_ss.70.1|g.178	0.99	9.04	63.37	9.66E-06	0.0008
21 days	twi_ss.28878.1|g.38683	-3.87	3.89	63.28	9.72E-06	0.0008
21 days	twi_ss.8930b.1|g.12126	-2.99	5.19	63.25	9.74E-06	0.0008
21 days	twi_ss.21105.1|g.27279	5.15	9.10	63.13	9.82E-06	0.0008
21 days	twi_ss.30255.1|g.40883	1.07	8.47	62.83	1.00E-05	0.0008
21 days	twi_ss.12669.1|g.16768	-3.27	4.72	62.76	1.01E-05	0.0008
21 days	twi_ss.26161.1|g.34639	-3.73	6.66	62.53	1.03E-05	0.0009
21 days	twi_ss.30691.1|g.41555	0.78	7.45	62.53	1.03E-05	0.0009
21 days	twi_ss.3382.1|g.5769	1.87	7.96	62.57	1.02E-05	0.0009
21 days	twi_ss.30108.1|g.40611	1.80	9.08	62.37	1.04E-05	0.0009
21 days	twi_ss.8457.1|g.11649	-4.02	5.52	62.36	1.04E-05	0.0009
21 days	twi_ss.6750.1|g.9832	2.13	8.24	62.23	1.05E-05	0.0009
21 days	twi_ss.30219.1|g.40817	-1.79	6.67	62.14	1.06E-05	0.0009
21 days	twi_ss.511.1|g.948	-3.70	6.64	62.04	1.06E-05	0.0009
21 days	twi_ss.16506.1|g.20593	-4.88	10.69	61.99	1.07E-05	0.0009
21 days	twi_ss.21231.1|g.27456	1.38	6.70	61.93	1.07E-05	0.0009

21 days	twi_ss.30418.1|g.41146	0.93	9.03	61.92	1.07E-05	0.0009
21 days	twi_ss.31144.1|g.42355	-2.82	5.39	61.55	1.10E-05	0.0009
21 days	twi_ss.30982.1|g.42019	0.99	9.70	61.45	1.11E-05	0.0009
21 days	twi_ss.503.1|g.907	-2.08	5.54	61.43	1.11E-05	0.0009
21 days	twi_ss.8805.1|g.11998	-3.66	5.31	61.32	1.12E-05	0.0009
21 days	twi_ss.14795.1|g.19270	-2.66	6.34	60.94	1.15E-05	0.0009
21 days	twi_ss.19570.3|g.25107	1.65	6.27	60.91	1.15E-05	0.0009
21 days	twi_ss.21393.1|g.27680	-3.50	6.75	60.91	1.16E-05	0.0009
21 days	twi_ss.26689.1|g.35443	-2.65	6.64	61.00	1.15E-05	0.0009
21 days	twi_ss.30222b.6|g.40808	-4.53	5.47	61.04	1.14E-05	0.0009
21 days	twi_ss.6094.1|g.8967	-4.56	4.30	60.99	1.15E-05	0.0009
21 days	twi_ss.23644.1|g.30455	1.28	7.35	60.62	1.18E-05	0.0009
21 days	twi_ss.21105.2|g.27285	6.17	7.96	60.54	1.19E-05	0.0009
21 days	twi_ss.2312b.1|g.4062	5.11	2.45	60.48	1.19E-05	0.0009
21 days	twi_ss.26746.1|g.35393	1.18	10.41	60.45	1.19E-05	0.0009
21 days	twi_ss.15060.1|g.19559	1.40	6.84	60.38	1.20E-05	0.0009
21 days	twi_ss.16656.2|g.21332	10.28	7.44	60.33	1.21E-05	0.0009
21 days	twi_ss.23722.1|g.30715	1.94	7.62	60.38	1.20E-05	0.0009
21 days	twi_ss.6907b.2|g.9981	-1.13	8.85	60.31	1.21E-05	0.0009
21 days	twi_ss.14446.1|g.18840	-2.69	5.53	60.20	1.22E-05	0.0009
21 days	twi_ss.5433b.1|g.8210	1.96	6.33	60.14	1.22E-05	0.0009
21 days	twi_ss.22436.1|g.28973	-2.57	7.76	60.02	1.23E-05	0.0009
21 days	twi_ss.7626.1|g.10685	1.84	7.31	60.02	1.23E-05	0.0009
21 days	twi_ss.5207.1|g.7832	-3.31	5.44	59.90	1.25E-05	0.0010
21 days	twi_ss.2064.1|g.3690	0.93	9.57	59.76	1.26E-05	0.0010
21 days	twi_ss.386.1|g.750	1.14	9.20	59.68	1.27E-05	0.0010
21 days	twi_ss.4223.1|g.6649	0.90	9.19	59.51	1.28E-05	0.0010
21 days	twi_ss.1173.1|g.2039	1.17	8.97	59.43	1.29E-05	0.0010
21 days	twi_ss.25132.1|g.33040	-2.75	7.05	59.38	1.29E-05	0.0010
21 days	twi_ss.4977.9|g.7534	9.50	6.96	59.41	1.29E-05	0.0010
21 days	twi_ss.8401.1|g.11548	1.44	6.15	59.35	1.30E-05	0.0010
21 days	twi_ss.14306.1|g.18665	-2.94	5.19	59.31	1.30E-05	0.0010
21 days	twi_ss.19934.1|g.25674	1.47	6.77	59.16	1.32E-05	0.0010

21 days	twi_ss.4307.1|g.6721	0.85	8.99	59.03	1.33E-05	0.0010
21 days	twi_ss.17485.1|g.22307	1.68	6.02	58.93	1.34E-05	0.0010
21 days	twi_ss.6096.1|g.8972	-1.63	6.44	58.92	1.34E-05	0.0010
21 days	twi_ss.6884.1|g.9949	2.70	6.45	58.96	1.34E-05	0.0010
21 days	twi_ss.18595.1|g.23781	5.11	5.80	58.87	1.35E-05	0.0010
21 days	twi_ss.18535.1|g.23608	-1.95	7.40	58.77	1.36E-05	0.0010
21 days	twi_ss.26480.1|g.35123	2.99	5.79	58.69	1.36E-05	0.0010
21 days	twi_ss.5201.2|g.7830	-2.41	6.34	58.68	1.37E-05	0.0010
21 days	twi_ss.1242.1|g.2217	-1.71	6.84	58.48	1.39E-05	0.0010
21 days	twi_ss.17801.1|g.22698	2.93	8.98	58.50	1.38E-05	0.0010
21 days	twi_ss.19030.11|g.24317	-4.17	11.19	58.54	1.38E-05	0.0010
21 days	twi_ss.26516.1|g.35125	1.48	6.15	58.55	1.38E-05	0.0010
21 days	twi_ss.3609.1|g.5991	-3.52	5.90	58.47	1.39E-05	0.0010
21 days	twi_ss.2017b.1|g.3603	-3.80	4.86	58.23	1.41E-05	0.0010
21 days	twi_ss.2512.1|g.4495	-2.78	5.15	58.09	1.43E-05	0.0010
21 days	twi_ss.26622.1|g.35284	1.56	7.49	58.13	1.42E-05	0.0010
21 days	twi_ss.2800a.3|g.5024	0.97	8.99	58.13	1.42E-05	0.0010
21 days	twi_ss.31777.1|g.43288	4.31	6.49	58.10	1.43E-05	0.0010
21 days	twi_ss.6541.1|g.9566	0.98	9.15	58.13	1.42E-05	0.0010
21 days	twi_ss.8770.1|g.11923	-2.98	7.09	58.25	1.41E-05	0.0010
21 days	twi_ss.18299.1|g.23330	-1.65	7.94	58.05	1.43E-05	0.0010
21 days	twi_ss.28761.2|g.38490	-2.44	5.07	57.96	1.44E-05	0.0010
21 days	twi_ss.31068.1|g.42246	0.92	8.50	57.94	1.45E-05	0.0010
21 days	twi_ss.9963.1|g.13288	2.72	6.05	57.93	1.45E-05	0.0010
21 days	twi_ss.14577.1|g.19060	-2.71	5.49	57.89	1.45E-05	0.0010
21 days	twi_ss.7980.1|g.11036	-3.05	5.39	57.70	1.47E-05	0.0010
21 days	twi_ss.24299b.2|g.31693	1.12	6.91	57.45	1.50E-05	0.0011
21 days	twi_ss.14250.1|g.18561	1.55	5.91	57.23	1.53E-05	0.0011
21 days	twi_ss.6558.5|g.9595	3.33	4.82	78.31	1.53E-05	0.0011
21 days	twi_ss.28429.1|g.38122	4.04	5.57	56.95	1.56E-05	0.0011
21 days	twi_ss.5279.3|g.7978	-7.21	3.31	77.86	1.57E-05	0.0011
21 days	twi_ss.9201.1|g.12410	-2.23	6.34	56.93	1.56E-05	0.0011
21 days	twi_ss.11367.1|g.14997	1.60	7.72	56.66	1.60E-05	0.0011

21 days	twi_ss.24063.1|g.31273	-2.60	5.38	56.64	1.60E-05	0.0011
21 days	twi_ss.24776.1|g.32540	-3.45	6.84	56.68	1.59E-05	0.0011
21 days	twi_ss.4484.1|g.6998	-3.46	6.38	56.70	1.59E-05	0.0011
21 days	twi_ss.12872.3|g.17048	4.11	6.27	56.36	1.63E-05	0.0011
21 days	twi_ss.13445.1|g.17741	1.56	6.40	56.38	1.63E-05	0.0011
21 days	twi_ss.29546.1|g.39653	5.49	5.60	56.36	1.63E-05	0.0011
21 days	twi_ss.1218.1|g.2194	-4.66	4.38	56.22	1.65E-05	0.0011
21 days	twi_ss.12538.1|g.16681	-2.99	4.16	56.22	1.65E-05	0.0011
21 days	twi_ss.26361b.4|g.34823	-9.56	7.24	56.22	1.65E-05	0.0011
21 days	twi_ss.20695.1|g.26791	1.78	6.71	56.12	1.67E-05	0.0011
21 days	twi_ss.2565.1|g.4595	-4.13	5.04	56.10	1.67E-05	0.0011
21 days	twi_ss.25745.1|g.33928	1.81	6.35	56.15	1.66E-05	0.0011
21 days	twi_ss.24587a.1|g.32232	-4.93	4.61	55.94	1.69E-05	0.0011
21 days	twi_ss.1117.1|g.1988	-2.91	6.37	55.87	1.70E-05	0.0011
21 days	twi_ss.21437.1|g.27736	1.25	8.44	55.75	1.72E-05	0.0011
21 days	twi_ss.22780.1|g.29402	-2.02	5.65	55.76	1.71E-05	0.0011
21 days	twi_ss.12986.1|g.17245	-2.80	6.05	55.69	1.73E-05	0.0011
21 days	twi_ss.21579.1|g.27977	-4.24	4.77	55.70	1.72E-05	0.0011
21 days	twi_ss.8767.1|g.11983	-3.83	5.66	55.64	1.73E-05	0.0012
21 days	twi_ss.10270.2|g.13868	6.64	2.84	55.58	1.74E-05	0.0012
21 days	twi_ss.6953.1|g.10022	-2.99	6.19	55.58	1.74E-05	0.0012
21 days	twi_ss.11861.1|g.15753	-2.43	6.78	55.51	1.75E-05	0.0012
21 days	twi_ss.15395.2|g.19950	1.18	7.88	55.44	1.76E-05	0.0012
21 days	twi_ss.2531.2|g.4548	6.35	3.08	75.49	1.76E-05	0.0012
21 days	twi_ss.29749.1|g.40117	1.49	6.32	55.41	1.76E-05	0.0012
21 days	twi_ss.384.1|g.691	-2.16	5.01	55.45	1.76E-05	0.0012
21 days	twi_ss.16476.1|g.21059	-2.56	6.38	55.30	1.78E-05	0.0012
21 days	twi_ss.23599.1|g.30281	-1.25	8.42	55.32	1.78E-05	0.0012
21 days	twi_ss.481.1|g.863	0.79	8.63	55.18	1.80E-05	0.0012
21 days	twi_ss.14825.1|g.19285	-2.60	4.19	55.14	1.80E-05	0.0012
21 days	twi_ss.11541a.1|g.15331	1.02	6.61	55.07	1.81E-05	0.0012
21 days	twi_ss.17094.1|g.21879	-5.41	4.78	54.93	1.83E-05	0.0012
21 days	twi_ss.20715.1|g.26800	-3.60	5.16	54.90	1.84E-05	0.0012

21 days	twi_ss.8887.1|g.12076	0.90	9.62	54.87	1.84E-05	0.0012
21 days	twi_ss.12814.1|g.16931	-2.87	5.64	54.79	1.85E-05	0.0012
21 days	twi_ss.22194.1|g.28748	-2.81	6.75	54.78	1.86E-05	0.0012
21 days	twi_ss.5986.1|g.8855	-2.74	6.81	54.81	1.85E-05	0.0012
21 days	twi_ss.27247.1|g.36184	-5.29	6.96	54.70	1.87E-05	0.0012
21 days	twi_ss.21349.1|g.27560	-10.69	8.36	54.62	1.88E-05	0.0012
21 days	twi_ss.27689.1|g.36875	1.03	7.68	54.56	1.89E-05	0.0012
21 days	twi_ss.3562.1|g.5942	-2.15	5.20	54.55	1.89E-05	0.0012
21 days	twi_ss.926.1|g.1644	1.02	8.05	54.53	1.89E-05	0.0012
21 days	twi_ss.23853b.2|g.30943	2.36	6.69	54.36	1.92E-05	0.0012
21 days	twi_ss.13406b.1|g.17721	0.98	8.53	54.15	1.95E-05	0.0012
21 days	twi_ss.2981.1|g.5305	-3.36	5.12	54.17	1.95E-05	0.0012
21 days	twi_ss.4178.1|g.6595	-3.48	5.24	54.20	1.94E-05	0.0012
21 days	twi_ss.21800.1|g.28295	1.96	5.80	53.96	1.98E-05	0.0012
21 days	twi_ss.3533.5|g.5910	-2.28	5.35	53.95	1.99E-05	0.0012
21 days	twi_ss.7623.1|g.10683	3.83	5.24	53.94	1.99E-05	0.0012
21 days	twi_ss.5416.1|g.8182	2.65	6.06	53.92	1.99E-05	0.0012
21 days	twi_ss.1442.2|g.2639	3.27	8.14	53.87	2.00E-05	0.0012
21 days	twi_ss.29980.2|g.40459	-3.50	5.07	53.82	2.01E-05	0.0012
21 days	twi_ss.30546.1|g.41379	4.61	7.52	53.79	2.01E-05	0.0012
21 days	twi_ss.1944.1|g.3472	-1.15	6.95	53.74	2.02E-05	0.0012
21 days	twi_ss.4467.1|g.6978	0.87	9.42	53.69	2.03E-05	0.0012
21 days	twi_ss.108.1|g.188	3.88	5.72	53.58	2.05E-05	0.0013
21 days	twi_ss.13249.1|g.17548	-2.29	6.44	53.48	2.06E-05	0.0013
21 days	twi_ss.21610.6|g.28011	2.72	6.06	53.46	2.07E-05	0.0013
21 days	twi_ss.26633b.1|g.35303	-4.23	10.12	53.49	2.06E-05	0.0013
21 days	twi_ss.30096b.2|g.40711	1.78	9.17	53.49	2.06E-05	0.0013
21 days	twi_ss.32100.1|g.43763	-4.90	4.96	53.44	2.07E-05	0.0013
21 days	twi_ss.7400.1|g.10411	-3.84	6.64	53.37	2.08E-05	0.0013
21 days	twi_ss.30208.2|g.40799	-4.32	4.65	53.33	2.09E-05	0.0013
21 days	twi_ss.28014.2|g.37321	-6.03	2.61	53.25	2.10E-05	0.0013
21 days	twi_ss.10752.1|g.14417	3.22	6.96	53.00	2.15E-05	0.0013
21 days	twi_ss.17127a.2|g.21899	1.04	8.76	53.00	2.15E-05	0.0013

21 days	twi_ss.23581.1|g.30352	-2.93	9.70	53.01	2.15E-05	0.0013
21 days	twi_ss.24955.1|g.32806	2.29	5.88	53.03	2.14E-05	0.0013
21 days	twi_ss.28487.3|g.38053	2.64	6.91	52.96	2.15E-05	0.0013
21 days	twi_ss.12920.1|g.17109	3.18	5.05	52.90	2.17E-05	0.0013
21 days	twi_ss.27510b.1|g.36650	-2.75	6.61	52.91	2.16E-05	0.0013
21 days	twi_ss.3473.1|g.5859	-4.55	4.12	52.87	2.17E-05	0.0013
21 days	twi_ss.15994.1|g.20599	-2.85	9.86	52.75	2.19E-05	0.0013
21 days	twi_ss.20778.1|g.26878	-1.44	5.82	52.78	2.19E-05	0.0013
21 days	twi_ss.30631.1|g.41471	1.10	9.37	52.73	2.20E-05	0.0013
21 days	twi_ss.9822.1|g.13153	-3.49	4.90	52.77	2.19E-05	0.0013
21 days	twi_ss.1034.1|g.1887	-10.38	8.05	52.55	2.23E-05	0.0013
21 days	twi_ss.23514.2|g.30168	4.43	5.15	52.55	2.23E-05	0.0013
21 days	twi_ss.29491.1|g.39721	-3.25	3.96	52.51	2.24E-05	0.0013
21 days	twi_ss.26111.1|g.34564	2.65	6.64	52.41	2.26E-05	0.0013
21 days	twi_ss.30985.2|g.42111	-2.96	4.86	52.42	2.26E-05	0.0013
21 days	twi_ss.14300.1|g.18619	-6.85	3.13	70.64	2.27E-05	0.0013
21 days	twi_ss.16288.1|g.20863	3.63	4.77	70.61	2.27E-05	0.0013
21 days	twi_ss.19155.1|g.24571	2.35	5.32	52.33	2.27E-05	0.0013
21 days	twi_ss.6359.1|g.9419	-2.51	8.55	52.27	2.28E-05	0.0013
21 days	twi_ss.15538.1|g.20108	-3.39	6.20	52.21	2.29E-05	0.0013
21 days	twi_ss.26260.1|g.34793	-1.32	6.42	52.17	2.30E-05	0.0013
21 days	twi_ss.16774.1|g.21438	0.87	9.29	51.88	2.36E-05	0.0014
21 days	twi_ss.24269.1|g.31712	2.50	6.62	51.91	2.35E-05	0.0014
21 days	twi_ss.6374.1|g.9426	-1.73	6.80	51.88	2.36E-05	0.0014
21 days	twi_ss.11802b.2|g.15695	-0.83	7.33	51.77	2.38E-05	0.0014
21 days	twi_ss.18396.1|g.23448	-8.17	5.06	69.77	2.38E-05	0.0014
21 days	twi_ss.20001.1|g.25769	-3.01	6.01	51.76	2.38E-05	0.0014
21 days	twi_ss.4127.1|g.6552	-3.20	5.25	51.74	2.39E-05	0.0014
21 days	twi_ss.12922.1|g.17106	-2.37	7.39	51.66	2.40E-05	0.0014
21 days	twi_ss.13265.1|g.17605	-2.13	4.93	51.66	2.40E-05	0.0014
21 days	twi_ss.5295.1|g.8034	-3.12	6.25	51.61	2.42E-05	0.0014
21 days	twi_ss.10168.1|g.13644	-3.75	4.87	51.43	2.45E-05	0.0014
21 days	twi_ss.26283.1|g.34925	-2.67	4.95	51.44	2.45E-05	0.0014

21 days	twi_ss.2690.3|g.4893	1.91	7.26	51.45	2.45E-05	0.0014
21 days	twi_ss.20132.2|g.25898	-3.13	5.27	51.37	2.46E-05	0.0014
21 days	twi_ss.14244.1|g.18538	0.91	8.45	51.16	2.51E-05	0.0014
21 days	twi_ss.16506.4|g.20590	-2.15	6.18	51.19	2.50E-05	0.0014
21 days	twi_ss.28413.1|g.37989	-3.69	4.52	51.17	2.51E-05	0.0014
21 days	twi_ss.28187.1|g.37571	-8.00	5.82	51.08	2.53E-05	0.0014
21 days	twi_ss.27090.4|g.36004	-5.54	4.50	51.04	2.54E-05	0.0014
21 days	twi_ss.29081.3|g.38992	-2.62	6.63	51.00	2.54E-05	0.0014
21 days	twi_ss.22636.1|g.29230	-2.42	6.48	50.96	2.55E-05	0.0014
21 days	twi_ss.6682.1|g.9749	1.63	7.81	50.91	2.56E-05	0.0014
21 days	twi_ss.7409.1|g.10417	3.62	4.68	50.87	2.57E-05	0.0014
21 days	twi_ss.6575.1|g.9608	-4.96	4.06	50.79	2.59E-05	0.0014
21 days	twi_ss.26920.1|g.35735	1.22	6.17	50.74	2.60E-05	0.0014
21 days	twi_ss.18303.1|g.23257	-3.28	6.30	50.71	2.61E-05	0.0014
21 days	twi_ss.25143.1|g.33053	-3.24	4.85	50.63	2.63E-05	0.0014
21 days	twi_ss.6472.1|g.9500	1.17	9.46	50.65	2.62E-05	0.0014
21 days	twi_ss.9086.2|g.12276	1.19	7.83	50.62	2.63E-05	0.0014
21 days	twi_ss.30351.1|g.41066	2.01	6.73	50.59	2.63E-05	0.0014
21 days	twi_ss.9484.1|g.12696	-3.52	4.92	50.51	2.65E-05	0.0015
21 days	twi_ss.16158.1|g.20747	1.55	6.81	50.43	2.67E-05	0.0015
21 days	twi_ss.17937.1|g.22815	-5.37	5.29	50.44	2.67E-05	0.0015
21 days	twi_ss.25703.4|g.33870	-5.17	4.92	50.34	2.69E-05	0.0015
21 days	twi_ss.1051.1|g.1890	1.83	4.96	50.28	2.71E-05	0.0015
21 days	twi_ss.22668.4|g.29253	-6.13	3.14	50.32	2.70E-05	0.0015
21 days	twi_ss.23480b.3|g.30142	-7.06	4.42	67.45	2.71E-05	0.0015
21 days	twi_ss.19956b.2|g.25691	1.39	6.65	50.24	2.72E-05	0.0015
21 days	twi_ss.23133.1|g.29738	1.87	7.15	50.02	2.77E-05	0.0015
21 days	twi_ss.908.1|g.1722	2.09	5.39	50.00	2.78E-05	0.0015
21 days	twi_ss.11082.1|g.14675	-2.48	4.86	49.95	2.79E-05	0.0015
21 days	twi_ss.12098.1|g.16040	0.85	9.26	49.86	2.81E-05	0.0015
21 days	twi_ss.2593.1|g.4662	1.08	7.08	49.82	2.82E-05	0.0015
21 days	twi_ss.31321a.2|g.42685	-3.84	4.83	49.83	2.82E-05	0.0015
21 days	twi_ss.5273.1|g.7993	-14.72	10.33	66.55	2.85E-05	0.0015

21 days	twi_ss.23297.1|g.29984	1.90	6.26	49.64	2.86E-05	0.0015
21 days	twi_ss.14612.3|g.19088	2.28	6.41	49.39	2.93E-05	0.0015
21 days	twi_ss.18540b.1|g.23638	1.63	6.83	49.45	2.91E-05	0.0015
21 days	twi_ss.21197a.1|g.27420	0.76	8.02	49.40	2.92E-05	0.0015
21 days	twi_ss.23142.2|g.29796	-2.07	6.61	49.50	2.90E-05	0.0015
21 days	twi_ss.26276.1|g.34766	0.79	7.97	49.40	2.93E-05	0.0015
21 days	twi_ss.28424.1|g.38003	2.36	6.85	49.38	2.93E-05	0.0015
21 days	twi_ss.4579.1|g.7109	-3.24	5.37	49.45	2.91E-05	0.0015
21 days	twi_ss.1515.1|g.2702	-2.11	6.42	49.28	2.96E-05	0.0016
21 days	twi_ss.5419.1|g.8184	-2.03	5.37	49.21	2.97E-05	0.0016
21 days	twi_ss.9186.1|g.12364	-1.37	6.36	49.22	2.97E-05	0.0016
21 days	twi_ss.6881.1|g.9943	3.84	5.46	49.19	2.98E-05	0.0016
21 days	twi_ss.6345.1|g.9359	0.86	7.13	49.16	2.99E-05	0.0016
21 days	twi_ss.10044.1|g.13359	-2.06	7.06	49.07	3.01E-05	0.0016
21 days	twi_ss.661.1|g.1181	-1.45	5.95	49.03	3.02E-05	0.0016
21 days	twi_ss.28687.1|g.38392	-3.76	4.72	49.00	3.03E-05	0.0016
21 days	twi_ss.7331.1|g.10349	1.47	6.55	48.90	3.06E-05	0.0016
21 days	twi_ss.4014.3|g.6432	-1.56	6.61	48.76	3.10E-05	0.0016
21 days	twi_ss.25248.1|g.33251	1.49	7.85	48.70	3.11E-05	0.0016
21 days	twi_ss.25248.2|g.33248	1.49	7.85	48.70	3.11E-05	0.0016
21 days	twi_ss.9105.1|g.12289	-1.26	6.59	48.70	3.11E-05	0.0016
21 days	twi_ss.18583.2|g.23692	-7.64	4.80	64.93	3.12E-05	0.0016
21 days	twi_ss.2516.1|g.4498	-3.01	6.22	48.53	3.16E-05	0.0016
21 days	twi_ss.2518.1|g.4528	2.52	5.44	48.48	3.17E-05	0.0016
21 days	twi_ss.25893.1|g.34159	-2.30	5.79	48.46	3.18E-05	0.0016
21 days	twi_ss.29206.1|g.39146	-1.91	6.90	48.44	3.19E-05	0.0016
21 days	twi_ss.9475.1|g.12659	-6.94	3.79	64.55	3.19E-05	0.0016
21 days	twi_ss.1294.1|g.2337	-1.96	5.82	48.37	3.21E-05	0.0016
21 days	twi_ss.5271.1|g.7892	-1.09	6.87	48.38	3.20E-05	0.0016
21 days	twi_ss.25233.1|g.33148	-1.79	6.15	48.29	3.23E-05	0.0016
21 days	twi_ss.30001b.1|g.40489	2.11	5.32	48.30	3.23E-05	0.0016
21 days	twi_ss.29246.1|g.39251	-2.01	6.12	48.25	3.24E-05	0.0016
21 days	twi_ss.18186.1|g.23109	-1.68	6.47	48.17	3.27E-05	0.0016

21 days	twi_ss.18751.1|g.23960	-5.37	3.34	48.15	3.27E-05	0.0016
21 days	twi_ss.19070a.4|g.24462	1.76	6.00	48.17	3.27E-05	0.0016
21 days	twi_ss.30242b.2|g.40842	8.25	5.72	48.20	3.26E-05	0.0016
21 days	twi_ss.25939.3|g.34238	-3.80	4.95	48.10	3.29E-05	0.0016
21 days	twi_ss.28494.1|g.38157	1.73	6.61	48.08	3.29E-05	0.0016
21 days	twi_ss.4972.1|g.7517	2.87	5.14	47.87	3.36E-05	0.0017
21 days	twi_ss.29224.1|g.39184	-1.90	6.29	47.81	3.37E-05	0.0017
21 days	twi_ss.29777.1|g.40102	-5.19	2.32	47.80	3.38E-05	0.0017
21 days	twi_ss.31358.1|g.42626	2.14	5.99	47.79	3.38E-05	0.0017
21 days	twi_ss.24443a.2|g.32034	-3.88	4.93	47.70	3.41E-05	0.0017
21 days	twi_ss.31159.1|g.42402	0.90	7.87	47.71	3.41E-05	0.0017
21 days	twi_ss.23382.1|g.30079	1.10	9.28	47.52	3.47E-05	0.0017
21 days	twi_ss.15435.1|g.19991	2.50	5.31	47.49	3.47E-05	0.0017
21 days	twi_ss.20264c.8|g.26050	8.18	4.91	62.65	3.57E-05	0.0018
21 days	twi_ss.5555.1|g.8428	-2.62	9.74	47.11	3.60E-05	0.0018
21 days	twi_ss.27332.1|g.36359	-1.76	6.28	47.07	3.61E-05	0.0018
21 days	twi_ss.6931.1|g.9997	-2.97	5.58	47.05	3.62E-05	0.0018
21 days	twi_ss.27247.2|g.36186	-3.53	7.65	46.96	3.65E-05	0.0018
21 days	twi_ss.21105.3|g.27275	5.22	6.10	46.91	3.66E-05	0.0018
21 days	twi_ss.22950.1|g.29594	-4.23	6.51	46.75	3.72E-05	0.0018
21 days	twi_ss.16245.2|g.20824	-1.95	5.60	46.60	3.77E-05	0.0018
21 days	twi_ss.17967b.1|g.22841	-2.03	6.05	46.64	3.76E-05	0.0018
21 days	twi_ss.21664c.4|g.27926	1.78	10.23	46.60	3.77E-05	0.0018
21 days	twi_ss.247.1|g.483	1.36	7.87	46.58	3.78E-05	0.0018
21 days	twi_ss.24880.1|g.32703	0.94	9.54	46.58	3.78E-05	0.0018
21 days	twi_ss.13440.1|g.17763	0.96	7.10	46.44	3.83E-05	0.0018
21 days	twi_ss.21114a.2|g.27303	-3.09	5.50	46.42	3.83E-05	0.0018
21 days	twi_ss.25132.2|g.33043	-2.19	5.65	46.36	3.86E-05	0.0019
21 days	twi_ss.32045.1|g.43715	1.02	8.88	46.27	3.89E-05	0.0019
21 days	twi_ss.26771.1|g.35501	1.92	5.31	46.23	3.90E-05	0.0019
21 days	twi_ss.16506.2|g.20591	-2.81	8.21	46.20	3.91E-05	0.0019
21 days	twi_ss.28384.1|g.37949	-2.50	4.75	46.16	3.93E-05	0.0019
21 days	twi_ss.30114.1|g.40707	-2.11	6.08	46.08	3.96E-05	0.0019

21 days	twi_ss.5815.1|g.8723	-3.24	6.92	45.87	4.04E-05	0.0019
21 days	twi_ss.8397.1|g.11532	-4.41	3.77	45.88	4.03E-05	0.0019
21 days	twi_ss.1956.1|g.3454	-1.48	5.91	45.81	4.06E-05	0.0019
21 days	twi_ss.12222.1|g.16260	1.61	7.14	45.77	4.08E-05	0.0019
21 days	twi_ss.18869.1|g.24133	-1.71	6.98	45.64	4.12E-05	0.0020
21 days	twi_ss.4977.8|g.7536	-4.13	5.84	45.63	4.13E-05	0.0020
21 days	twi_ss.1660.1|g.2916	-4.22	5.19	45.53	4.17E-05	0.0020
21 days	twi_ss.14969.1|g.19471	1.34	7.00	45.46	4.20E-05	0.0020
21 days	twi_ss.4259.1|g.6680	-2.99	7.61	45.47	4.19E-05	0.0020
21 days	twi_ss.16506.3|g.20586	-3.05	9.24	45.33	4.25E-05	0.0020
21 days	twi_ss.21799.1|g.28294	1.07	7.61	45.34	4.24E-05	0.0020
21 days	twi_ss.27407.1|g.36535	-4.71	3.87	45.34	4.24E-05	0.0020
21 days	twi_ss.29547.1|g.39665	6.33	3.61	59.83	4.24E-05	0.0020
21 days	twi_ss.6506.1|g.9518	1.48	6.73	45.36	4.24E-05	0.0020
21 days	twi_ss.4989.1|g.7605	1.51	6.67	45.26	4.28E-05	0.0020
21 days	twi_ss.21473.1|g.27813	1.91	5.92	45.07	4.35E-05	0.0020
21 days	twi_ss.8749.1|g.11986	-3.36	5.66	45.05	4.36E-05	0.0020
21 days	twi_ss.4014.5|g.6429	-2.34	8.86	45.01	4.38E-05	0.0020
21 days	twi_ss.17104.1|g.21883	-5.86	4.23	44.96	4.40E-05	0.0020
21 days	twi_ss.7349.1|g.10353	-4.50	6.25	44.94	4.41E-05	0.0020
21 days	twi_ss.21065.1|g.27251	-3.10	4.63	44.73	4.50E-05	0.0021
21 days	twi_ss.5716.1|g.8554	3.49	5.71	44.51	4.59E-05	0.0021
21 days	twi_ss.27617.1|g.36652	1.67	6.02	44.47	4.61E-05	0.0021
21 days	twi_ss.14677.1|g.19126	1.11	9.07	44.45	4.62E-05	0.0021
21 days	twi_ss.16059.1|g.20655	-1.24	6.89	44.43	4.63E-05	0.0021
21 days	twi_ss.18744.1|g.23953	-2.82	5.05	44.36	4.66E-05	0.0021
21 days	twi_ss.19831.1|g.25472	-2.30	6.83	44.32	4.68E-05	0.0021
21 days	twi_ss.19831.8|g.25466	-2.30	6.83	44.32	4.68E-05	0.0021
21 days	twi_ss.30320.1|g.41003	-2.92	4.91	44.32	4.68E-05	0.0021
21 days	twi_ss.10185.1|g.13679	1.22	8.05	44.20	4.74E-05	0.0022
21 days	twi_ss.13330.1|g.17615	-2.34	5.33	44.16	4.75E-05	0.0022
21 days	twi_ss.3365c.1|g.5761	-4.07	4.81	44.12	4.77E-05	0.0022
21 days	twi_ss.1994.1|g.3586	2.17	5.57	44.07	4.80E-05	0.0022

21 days	twi_ss.25247.1|g.33252	-2.17	5.31	43.95	4.85E-05	0.0022
21 days	twi_ss.26671.1|g.35345	0.89	8.71	43.90	4.88E-05	0.0022
21 days	twi_ss.27979b.1|g.37212	0.81	9.27	43.94	4.86E-05	0.0022
21 days	twi_ss.28724.1|g.38426	-2.15	8.98	43.90	4.88E-05	0.0022
21 days	twi_ss.31444.1|g.42805	-2.61	9.81	43.95	4.85E-05	0.0022
21 days	twi_ss.31635.1|g.43104	-5.05	4.12	43.91	4.87E-05	0.0022
21 days	twi_ss.59.1|g.18	-3.28	4.80	43.96	4.84E-05	0.0022
21 days	twi_ss.11570.1|g.15365	-0.89	10.07	43.82	4.91E-05	0.0022
21 days	twi_ss.22200.1|g.28758	-2.68	5.33	43.76	4.94E-05	0.0022
21 days	twi_ss.27261.1|g.36228	-1.93	6.63	43.72	4.96E-05	0.0022
21 days	twi_ss.23836a.1|g.30868	-2.10	7.46	43.69	4.98E-05	0.0022
21 days	twi_ss.7665.1|g.10740	-3.38	5.62	43.63	5.01E-05	0.0022
21 days	twi_ss.115.1|g.206	-1.41	7.10	43.57	5.04E-05	0.0022
21 days	twi_ss.30248.1|g.40878	1.20	6.70	43.55	5.04E-05	0.0022
21 days	twi_ss.25142.1|g.33049	-4.12	3.78	43.50	5.07E-05	0.0023
21 days	twi_ss.19481.1|g.25027	-4.85	4.64	43.45	5.09E-05	0.0023
21 days	twi_ss.25212.1|g.33076	-1.66	6.13	43.46	5.09E-05	0.0023
21 days	twi_ss.29902.4|g.40370	5.23	2.15	56.97	5.09E-05	0.0023
21 days	twi_ss.18605.1|g.23719	-6.01	3.01	56.91	5.11E-05	0.0023
21 days	twi_ss.2900.1|g.5200	-1.70	6.12	43.43	5.11E-05	0.0023
21 days	twi_ss.12665.1|g.16747	-2.80	4.68	43.39	5.13E-05	0.0023
21 days	twi_ss.18360.1|g.23367	-2.62	8.00	43.37	5.13E-05	0.0023
21 days	twi_ss.1937.1|g.3477	-1.99	5.00	43.36	5.14E-05	0.0023
21 days	twi_ss.30590.1|g.41391	-1.51	5.45	43.26	5.19E-05	0.0023
21 days	twi_ss.14603.1|g.19056	0.78	7.43	43.19	5.23E-05	0.0023
21 days	twi_ss.25472.1|g.33509	-2.27	4.13	43.14	5.25E-05	0.0023
21 days	twi_ss.30136b.2|g.40637	-2.33	5.65	43.13	5.26E-05	0.0023
21 days	twi_ss.10775.1|g.14409	2.36	6.71	43.05	5.30E-05	0.0023
21 days	twi_ss.26278.1|g.34948	-1.33	7.91	43.06	5.29E-05	0.0023
21 days	twi_ss.27326.2|g.36338	-3.91	6.71	43.08	5.28E-05	0.0023
21 days	twi_ss.3519.1|g.5890	-2.65	4.34	43.05	5.30E-05	0.0023
21 days	twi_ss.15550.1|g.20114	3.24	4.41	42.92	5.37E-05	0.0023
21 days	twi_ss.4595.1|g.7163	-2.79	5.45	42.91	5.37E-05	0.0023

21 days	twi_ss.8094.1|g.11173	-2.81	5.76	42.76	5.46E-05	0.0024
21 days	twi_ss.10005.1|g.13311	-3.43	3.84	42.63	5.52E-05	0.0024
21 days	twi_ss.16421.1|g.21003	-3.49	5.22	42.65	5.52E-05	0.0024
21 days	twi_ss.17392.1|g.22172	-2.42	6.82	42.57	5.56E-05	0.0024
21 days	twi_ss.28921.1|g.38793	-8.14	4.19	88.38	5.57E-05	0.0024
21 days	twi_ss.7769.1|g.10845	-1.81	6.58	42.53	5.58E-05	0.0024
21 days	twi_ss.21485.1|g.27864	0.93	9.85	42.46	5.62E-05	0.0024
21 days	twi_ss.7142.1|g.10189	0.76	7.28	42.46	5.62E-05	0.0024
21 days	twi_ss.21577.1|g.27967	-4.31	4.21	42.34	5.69E-05	0.0024
21 days	twi_ss.5818.1|g.8727	-2.49	6.56	42.31	5.71E-05	0.0024
21 days	twi_ss.524.1|g.947	-3.46	4.40	42.28	5.73E-05	0.0024
21 days	twi_ss.1966.2|g.3482	1.97	6.10	42.23	5.75E-05	0.0024
21 days	twi_ss.4424.1|g.6923	-2.45	4.40	42.21	5.77E-05	0.0025
21 days	twi_ss.27623.4|g.36634	-2.25	6.29	42.11	5.82E-05	0.0025
21 days	twi_ss.19960b.5|g.25659	-7.21	5.06	54.91	5.84E-05	0.0025
21 days	twi_ss.32072.1|g.43738	-2.85	3.91	42.05	5.86E-05	0.0025
21 days	twi_ss.12025.1|g.15916	1.85	7.28	42.01	5.88E-05	0.0025
21 days	twi_ss.14119.1|g.18425	3.55	4.54	41.85	5.98E-05	0.0025
21 days	twi_ss.30156.5|g.40644	1.05	7.34	41.81	6.00E-05	0.0025
21 days	twi_ss.3408.1|g.5790	-4.08	4.19	41.82	6.00E-05	0.0025
21 days	twi_ss.6603b.1|g.9643	2.94	8.46	41.80	6.01E-05	0.0025
21 days	twi_ss.26676.1|g.35344	-6.63	3.58	54.43	6.03E-05	0.0025
21 days	twi_ss.31675.1|g.43127	-5.13	4.52	41.73	6.05E-05	0.0025
21 days	twi_ss.11794.1|g.15702	0.90	7.47	41.67	6.09E-05	0.0025
21 days	twi_ss.12150.1|g.16083	-3.46	6.77	41.68	6.08E-05	0.0025
21 days	twi_ss.5427.2|g.8207	-3.38	3.69	41.60	6.13E-05	0.0026
21 days	twi_ss.18303.5|g.23253	-3.15	9.07	41.50	6.19E-05	0.0026
21 days	twi_ss.7485.2|g.10485	1.10	9.24	41.50	6.20E-05	0.0026
21 days	twi_ss.29296.1|g.39294	1.07	9.97	41.42	6.25E-05	0.0026
21 days	twi_ss.10271a.1|g.13759	1.18	7.13	41.37	6.28E-05	0.0026
21 days	twi_ss.23785b.1|g.30813	-1.75	7.64	41.36	6.28E-05	0.0026
21 days	twi_ss.30027b.2|g.40527	2.54	8.55	41.35	6.29E-05	0.0026
21 days	twi_ss.10588.1|g.14230	-5.91	6.24	41.27	6.34E-05	0.0026

21 days	twi_ss.10632.1|g.14268	-2.32	6.68	41.32	6.31E-05	0.0026
21 days	twi_ss.21865.1|g.28349	-2.13	7.57	41.26	6.35E-05	0.0026
21 days	twi_ss.25720.1|g.33885	-3.99	4.65	41.25	6.36E-05	0.0026
21 days	twi_ss.2715.1|g.4854	4.17	4.83	41.25	6.35E-05	0.0026
21 days	twi_ss.27783b.1|g.36950	-4.55	6.65	41.29	6.33E-05	0.0026
21 days	twi_ss.6810a.1|g.9870	-2.52	5.85	41.27	6.34E-05	0.0026
21 days	twi_ss.22686.1|g.29319	-1.31	5.75	41.16	6.42E-05	0.0026
21 days	twi_ss.21631b.4|g.28006	4.11	5.43	41.14	6.43E-05	0.0026
21 days	twi_ss.10112.1|g.13569	0.99	8.60	41.03	6.50E-05	0.0026
21 days	twi_ss.13327.1|g.17560	2.98	5.01	41.11	6.45E-05	0.0026
21 days	twi_ss.25629.1|g.33825	0.89	8.20	41.06	6.48E-05	0.0026
21 days	twi_ss.26988b.1|g.35833	-3.03	5.53	41.06	6.48E-05	0.0026
21 days	twi_ss.2757.1|g.5039	-1.14	6.62	41.03	6.50E-05	0.0026
21 days	twi_ss.28839.1|g.38609	-3.42	4.86	41.07	6.47E-05	0.0026
21 days	twi_ss.30386.1|g.41117	-1.53	5.36	41.07	6.48E-05	0.0026
21 days	twi_ss.7359.1|g.10368	-3.36	5.18	41.02	6.51E-05	0.0026
21 days	twi_ss.25969.1|g.34284	-2.01	5.36	41.00	6.52E-05	0.0026
21 days	twi_ss.16912.2|g.21548	-5.84	3.67	40.79	6.66E-05	0.0027
21 days	twi_ss.2778.1|g.5047	-0.87	8.33	40.66	6.75E-05	0.0027
21 days	twi_ss.8361a.1|g.11511	0.81	7.31	40.65	6.76E-05	0.0027
21 days	twi_ss.4860.1|g.7466	-1.76	6.00	40.62	6.79E-05	0.0027
21 days	twi_ss.23819.1|g.30881	2.71	4.36	40.57	6.82E-05	0.0027
21 days	twi_ss.656.1|g.1168	1.05	6.58	40.48	6.88E-05	0.0028
21 days	twi_ss.8434.1|g.11622	-2.74	6.27	40.44	6.91E-05	0.0028
21 days	twi_ss.17370.2|g.22147	0.82	9.05	40.40	6.94E-05	0.0028
21 days	twi_ss.2836.1|g.5119	-2.37	5.20	40.40	6.94E-05	0.0028
21 days	twi_ss.3117.1|g.5501	2.16	5.94	40.39	6.95E-05	0.0028
21 days	twi_ss.19607.1|g.25175	0.75	7.80	40.32	7.00E-05	0.0028
21 days	twi_ss.20143.1|g.25882	1.14	6.40	40.33	6.99E-05	0.0028
21 days	twi_ss.20143.2|g.25887	1.14	6.40	40.33	6.99E-05	0.0028
21 days	twi_ss.20143.3|g.25884	1.14	6.40	40.33	6.99E-05	0.0028
21 days	twi_ss.20143.4|g.25883	1.14	6.40	40.33	6.99E-05	0.0028
21 days	twi_ss.8661b.1|g.11804	-2.54	4.34	40.31	7.01E-05	0.0028

21 days	twi_ss.8200.1|g.11287	2.11	5.60	40.10	7.16E-05	0.0028
21 days	twi_ss.28547.1|g.38243	-2.31	7.24	40.02	7.22E-05	0.0028
21 days	twi_ss.30156.1|g.40647	-4.15	6.39	40.02	7.22E-05	0.0028
21 days	twi_ss.8321.1|g.11424	1.10	6.79	39.97	7.26E-05	0.0029
21 days	twi_ss.20694.1|g.26746	-5.95	4.34	39.94	7.28E-05	0.0029
21 days	twi_ss.11056b.2|g.14704	1.00	6.52	39.88	7.33E-05	0.0029
21 days	twi_ss.12230.1|g.16255	2.04	6.91	39.88	7.33E-05	0.0029
21 days	twi_ss.24387d.5|g.31908	-3.07	4.68	39.84	7.36E-05	0.0029
21 days	twi_ss.2662.1|g.4821	1.27	6.95	39.85	7.36E-05	0.0029
21 days	twi_ss.31822.1|g.43347	1.28	6.20	39.84	7.36E-05	0.0029
21 days	twi_ss.14951.1|g.19385	1.88	6.27	39.82	7.38E-05	0.0029
21 days	twi_ss.25852.1|g.34092	-2.16	4.62	39.79	7.40E-05	0.0029
21 days	twi_ss.26691.1|g.35424	-2.69	8.22	39.75	7.43E-05	0.0029
21 days	twi_ss.13791.2|g.18112	2.20	5.47	39.60	7.55E-05	0.0029
21 days	twi_ss.16882.1|g.21509	2.42	5.32	39.61	7.54E-05	0.0029
21 days	twi_ss.2604.1|g.4680	2.50	6.16	39.59	7.56E-05	0.0029
21 days	twi_ss.57.2|g.130	0.90	8.52	39.58	7.56E-05	0.0029
21 days	twi_ss.1014.1|g.1834	-2.32	4.93	39.54	7.60E-05	0.0029
21 days	twi_ss.29469.1|g.39702	-2.88	5.46	39.55	7.59E-05	0.0029
21 days	twi_ss.21105.6|g.27278	3.93	5.41	39.46	7.66E-05	0.0030
21 days	twi_ss.31219.1|g.42419	1.84	6.87	39.45	7.67E-05	0.0030
21 days	twi_ss.14962.1|g.19413	1.15	6.30	39.44	7.68E-05	0.0030
21 days	twi_ss.22789.1|g.29433	-3.19	5.87	39.38	7.73E-05	0.0030
21 days	twi_ss.30565a.6|g.41425	-2.53	8.28	39.36	7.74E-05	0.0030
21 days	twi_ss.9015.1|g.12194	0.84	8.42	39.25	7.83E-05	0.0030
21 days	twi_ss.20396.1|g.26276	1.33	5.90	39.19	7.89E-05	0.0030
21 days	twi_ss.16983.1|g.21687	-1.47	6.15	39.10	7.96E-05	0.0030
21 days	twi_ss.23691.1|g.30491	1.06	11.80	39.09	7.97E-05	0.0030
21 days	twi_ss.2663.1|g.4820	1.35	6.80	39.09	7.97E-05	0.0030
21 days	twi_ss.6923.2|g.10001	-2.88	7.64	39.11	7.95E-05	0.0030
21 days	twi_ss.2273.1|g.4015	-2.17	5.60	39.06	7.99E-05	0.0030
21 days	twi_ss.5143.1|g.7782	-2.23	5.55	39.00	8.05E-05	0.0031
21 days	twi_ss.1999.3|g.3592	-7.01	3.58	50.29	8.06E-05	0.0031

21 days	twi_ss.23740.1|g.30719	0.93	10.16	38.96	8.09E-05	0.0031
21 days	twi_ss.8263.1|g.11334	-1.80	5.43	38.92	8.12E-05	0.0031
21 days	twi_ss.15436.1|g.19994	2.93	5.71	38.89	8.15E-05	0.0031
21 days	twi_ss.19701b.2|g.25349	-1.21	8.39	38.82	8.21E-05	0.0031
21 days	twi_ss.10262.1|g.13762	-2.46	4.65	38.77	8.25E-05	0.0031
21 days	twi_ss.26361a.1|g.34928	-10.47	6.15	49.91	8.29E-05	0.0031
21 days	twi_ss.17309.1|g.22092	1.14	6.81	38.68	8.34E-05	0.0031
21 days	twi_ss.17490.1|g.22321	-2.86	4.37	38.67	8.34E-05	0.0031
21 days	twi_ss.1269.2|g.2279	-4.95	4.46	38.65	8.36E-05	0.0031
21 days	twi_ss.12283.1|g.16263	-1.82	5.76	38.52	8.48E-05	0.0032
21 days	twi_ss.25806.1|g.34080	-5.22	4.73	38.52	8.48E-05	0.0032
21 days	twi_ss.20452.1|g.26367	1.90	9.11	38.45	8.54E-05	0.0032
21 days	twi_ss.718.2|g.1251	-12.05	7.70	49.47	8.57E-05	0.0032
21 days	twi_ss.21339.5|g.27594	-4.72	2.19	42.98	8.65E-05	0.0032
21 days	twi_ss.26110.1|g.34567	3.99	4.03	38.32	8.67E-05	0.0032
21 days	twi_ss.19671.1|g.25299	0.73	8.98	38.30	8.68E-05	0.0032
21 days	twi_ss.21575.1|g.27963	-2.53	4.48	38.22	8.76E-05	0.0032
21 days	twi_ss.24909.1|g.32761	-1.93	6.32	38.23	8.75E-05	0.0032
21 days	twi_ss.818.1|g.1403	-1.27	5.51	38.24	8.74E-05	0.0032
21 days	twi_ss.459b.3|g.814	-1.73	6.78	38.21	8.77E-05	0.0032
21 days	twi_ss.13431.1|g.17740	0.88	8.69	38.17	8.81E-05	0.0033
21 days	twi_ss.16483.1|g.21068	-2.46	5.05	38.09	8.89E-05	0.0033
21 days	twi_ss.1881.1|g.3310	-2.39	5.98	38.10	8.88E-05	0.0033
21 days	twi_ss.30137.1|g.40684	2.55	4.89	38.09	8.89E-05	0.0033
21 days	twi_ss.12268.1|g.16337	-2.45	4.83	37.96	9.02E-05	0.0033
21 days	twi_ss.26708.1|g.35428	2.13	6.20	37.94	9.04E-05	0.0033
21 days	twi_ss.29239.1|g.39216	-7.10	3.77	48.76	9.03E-05	0.0033
21 days	twi_ss.30116.1|g.40638	-1.88	6.04	37.94	9.03E-05	0.0033
21 days	twi_ss.2383.1|g.4297	-5.95	4.09	37.89	9.09E-05	0.0033
21 days	twi_ss.7858.6|g.10905	1.33	6.84	37.87	9.10E-05	0.0033
21 days	twi_ss.22383.1|g.28898	-3.86	4.26	37.83	9.15E-05	0.0033
21 days	twi_ss.10279.1|g.13918	5.73	3.45	48.47	9.23E-05	0.0034
21 days	twi_ss.23880.1|g.31029	4.35	11.32	37.73	9.25E-05	0.0034

21 days	twi_ss.14760.1|g.19255	-5.40	3.35	48.38	9.29E-05	0.0034
21 days	twi_ss.23575.1|g.30271	2.53	6.28	37.70	9.27E-05	0.0034
21 days	twi_ss.24527.1|g.32096	-2.91	6.16	37.68	9.30E-05	0.0034
21 days	twi_ss.28969.1|g.38861	1.36	7.32	37.67	9.31E-05	0.0034
21 days	twi_ss.29220a.1|g.39210	-3.18	4.34	37.67	9.31E-05	0.0034
21 days	twi_ss.27700.1|g.36906	0.96	6.19	37.64	9.34E-05	0.0034
21 days	twi_ss.28048.1|g.37371	2.72	7.95	37.54	9.44E-05	0.0034
21 days	twi_ss.7208.1|g.10259	2.14	5.49	37.54	9.44E-05	0.0034
21 days	twi_ss.398b.4|g.760	-1.93	7.62	37.47	9.51E-05	0.0034
21 days	twi_ss.3211.1|g.5629	0.81	9.18	37.43	9.56E-05	0.0034
21 days	twi_ss.7069.1|g.10128	-1.13	6.06	37.42	9.56E-05	0.0034
21 days	twi_ss.28726.1|g.38471	-1.18	5.53	37.40	9.58E-05	0.0034
21 days	twi_ss.13937.1|g.18267	-4.50	3.87	37.36	9.63E-05	0.0035
21 days	twi_ss.1877.1|g.3338	-2.30	6.06	37.36	9.63E-05	0.0035
21 days	twi_ss.24804.1|g.32562	0.81	7.86	37.33	9.66E-05	0.0035
21 days	twi_ss.27783a.4|g.36937	-7.24	5.28	37.34	9.65E-05	0.0035
21 days	twi_ss.15151.1|g.19718	-5.65	3.50	37.27	9.73E-05	0.0035
21 days	twi_ss.17576.2|g.22474	-5.37	3.39	37.27	9.73E-05	0.0035
21 days	twi_ss.20469.1|g.26384	-6.04	4.98	37.24	9.76E-05	0.0035
21 days	twi_ss.30027a.5|g.40531	7.61	4.96	37.22	9.78E-05	0.0035
21 days	twi_ss.27868.1|g.37038	-1.39	8.63	37.14	9.87E-05	0.0035
21 days	twi_ss.4368.1|g.6882	-7.33	3.86	47.45	9.97E-05	0.0035
21 days	twi_ss.5304a.1|g.8061	-2.84	7.92	37.04	9.98E-05	0.0035
21 days	twi_ss.7133a.1|g.10161	-1.25	6.91	37.05	9.97E-05	0.0035
21 days	twi_ss.6013.1|g.8882	-2.07	5.38	37.03	9.99E-05	0.0035
21 days	twi_ss.20285.1|g.26104	-3.42	3.65	37.01	0.0001	0.0035
21 days	twi_ss.21016.1|g.27185	1.36	6.25	36.95	0.0001	0.0036
21 days	twi_ss.29791.4|g.40179	-2.11	4.76	36.95	0.0001	0.0036
21 days	twi_ss.13003.1|g.17229	0.58	7.60	36.93	0.0001	0.0036
21 days	twi_ss.9174.1|g.12374	-3.79	5.25	36.89	0.0001	0.0036
21 days	twi_ss.11539a.1|g.15322	4.02	7.86	36.83	0.0001	0.0036
21 days	twi_ss.19925.1|g.25689	1.64	5.31	36.84	0.0001	0.0036
21 days	twi_ss.27582c.1|g.36775	1.27	7.64	36.84	0.0001	0.0036

21 days	twi_ss.5415.4|g.8178	3.50	5.56	36.85	0.0001	0.0036
21 days	twi_ss.669.1|g.1200	-2.58	4.91	36.82	0.0001	0.0036
21 days	twi_ss.9501.1|g.12770	2.53	4.76	36.87	0.0001	0.0036
21 days	twi_ss.26301.1|g.34878	0.94	7.18	36.71	0.0001	0.0036
21 days	twi_ss.20330.1|g.26166	1.04	6.18	36.69	0.0001	0.0036
21 days	twi_ss.295.1|g.572	-2.26	4.82	36.66	0.0001	0.0036
21 days	twi_ss.25236.3|g.33194	-0.98	5.89	36.59	0.0001	0.0037
21 days	twi_ss.13326.1|g.17604	3.66	3.87	36.50	0.0001	0.0037
21 days	twi_ss.17095.1|g.21886	-4.93	4.67	36.50	0.0001	0.0037
21 days	twi_ss.26694b.3|g.35409	1.60	4.37	36.38	0.0001	0.0037
21 days	twi_ss.28487.2|g.38056	2.17	7.83	36.34	0.0001	0.0037
21 days	twi_ss.16652.3|g.21312	2.02	6.61	36.31	0.0001	0.0038
21 days	twi_ss.23181.1|g.29805	-1.75	6.61	36.27	0.0001	0.0038
21 days	twi_ss.29566.1|g.39761	1.50	7.88	36.27	0.0001	0.0038
21 days	twi_ss.16229.2|g.20811	1.33	6.55	36.26	0.0001	0.0038
21 days	twi_ss.5518.1|g.8303	-3.45	4.49	36.25	0.0001	0.0038
21 days	twi_ss.31892a.1|g.43457	-1.15	7.45	36.20	0.0001	0.0038
21 days	twi_ss.25634b.10|g.33766	-7.07	3.42	46.18	0.0001	0.0038
21 days	twi_ss.23162.1|g.29804	-0.94	6.15	36.18	0.0001	0.0038
21 days	twi_ss.8118.1|g.11181	0.86	7.87	36.13	0.0001	0.0038
21 days	twi_ss.3007.1|g.5346	2.10	5.33	36.11	0.0001	0.0038
21 days	twi_ss.31805.1|g.43321	1.18	7.21	36.04	0.0001	0.0038
21 days	twi_ss.20410.1|g.26292	-7.32	3.46	45.89	0.0001	0.0038
21 days	twi_ss.8568.1|g.11723	-3.83	4.28	35.97	0.0001	0.0038
21 days	twi_ss.20345.1|g.26181	-1.11	5.89	35.94	0.0001	0.0039
21 days	twi_ss.27737.2|g.36921	-1.04	7.98	35.93	0.0001	0.0039
21 days	twi_ss.10271b.2|g.13825	1.34	8.06	35.91	0.0001	0.0039
21 days	twi_ss.25210.1|g.33117	-2.33	6.26	35.88	0.0001	0.0039
21 days	twi_ss.26382.1|g.34889	0.93	6.31	35.87	0.0001	0.0039
21 days	twi_ss.12029.1|g.15878	0.76	8.35	35.85	0.0001	0.0039
21 days	twi_ss.5227.1|g.7855	0.95	8.20	35.83	0.0001	0.0039
21 days	twi_ss.10511.1|g.14144	0.64	7.98	35.82	0.0001	0.0039
21 days	twi_ss.5279.2|g.7975	-6.96	3.87	45.61	0.0001	0.0039

21 days	twi_ss.14940.1|g.19451	2.05	6.20	35.75	0.0001	0.0039
21 days	twi_ss.2996.1|g.5323	-1.78	5.08	35.72	0.0001	0.0039
21 days	twi_ss.1037.1|g.1879	-2.33	3.93	35.70	0.0001	0.0039
21 days	twi_ss.10103.1|g.13571	-2.81	4.93	35.67	0.0001	0.0039
21 days	twi_ss.16555.3|g.21235	-4.66	5.47	35.63	0.0001	0.0039
21 days	twi_ss.22998.1|g.29645	3.34	4.20	35.61	0.0001	0.0039
21 days	twi_ss.2398a.2|g.4265	2.74	5.28	35.62	0.0001	0.0039
21 days	twi_ss.3088.1|g.5481	-3.62	4.60	35.64	0.0001	0.0039
21 days	twi_ss.852.1|g.1428	-0.92	9.60	35.61	0.0001	0.0039
21 days	twi_ss.19471.1|g.24990	1.35	5.77	35.58	0.0001	0.0039
21 days	twi_ss.28950.1|g.38870	2.26	5.28	35.56	0.0001	0.0039
21 days	twi_ss.28950.2|g.38871	2.26	5.28	35.56	0.0001	0.0039
21 days	twi_ss.168.1|g.248	-3.22	4.44	35.52	0.0001	0.0040
21 days	twi_ss.20032.1|g.25789	-2.36	6.07	35.44	0.0001	0.0040
21 days	twi_ss.3149.1|g.5588	1.14	7.11	35.44	0.0001	0.0040
21 days	twi_ss.6809.1|g.9871	-1.31	8.99	35.45	0.0001	0.0040
21 days	twi_ss.31879.2|g.43447	1.95	7.02	35.41	0.0001	0.0040
21 days	twi_ss.27731.1|g.36953	2.53	5.07	35.34	0.0001	0.0040
21 days	twi_ss.15987.1|g.20595	-2.75	5.94	35.32	0.0001	0.0040
21 days	twi_ss.16520.1|g.21215	0.86	6.89	35.25	0.0001	0.0040
21 days	twi_ss.4535.2|g.7041	0.84	7.87	35.24	0.0001	0.0040
21 days	twi_ss.811.1|g.1442	-3.31	4.06	35.22	0.0001	0.0041
21 days	twi_ss.10092.1|g.13514	-1.08	6.86	35.20	0.0001	0.0041
21 days	twi_ss.20648.1|g.26650	1.04	6.64	35.21	0.0001	0.0041
21 days	twi_ss.20029.1|g.25796	-2.02	4.78	35.14	0.0001	0.0041
21 days	twi_ss.3349.1|g.5752	-3.32	4.43	35.15	0.0001	0.0041
21 days	twi_ss.12104.1|g.16038	-2.67	3.98	35.12	0.0001	0.0041
21 days	twi_ss.25141.1|g.33048	-6.14	3.05	44.63	0.0001	0.0041
21 days	twi_ss.16953.1|g.21649	0.89	7.83	35.05	0.0001	0.0041
21 days	twi_ss.25826.1|g.34060	1.76	4.87	35.00	0.0001	0.0041
21 days	twi_ss.31413.1|g.42762	1.91	5.05	35.00	0.0001	0.0041
21 days	twi_ss.4014.1|g.6434	-2.32	5.37	35.01	0.0001	0.0041
21 days	twi_ss.18742.1|g.23938	-3.19	5.27	34.96	0.0001	0.0041

21 days	twi_ss.20449b.1|g.26353	-5.15	4.07	34.96	0.0001	0.0041
21 days	twi_ss.30481.1|g.41261	0.83	9.16	34.95	0.0001	0.0041
21 days	twi_ss.7028.1|g.10085	-2.27	6.14	34.95	0.0001	0.0041
21 days	twi_ss.4558.2|g.7085	-2.52	5.72	34.88	0.0001	0.0042
21 days	twi_ss.29085.1|g.38997	-6.80	4.79	34.79	0.0001	0.0042
21 days	twi_ss.29236.1|g.39201	1.07	7.03	34.80	0.0001	0.0042
21 days	twi_ss.27623.6|g.36625	1.25	8.21	34.77	0.0001	0.0042
21 days	twi_ss.26025.1|g.34427	-1.51	5.64	34.72	0.0001	0.0042
21 days	twi_ss.9076.1|g.12263	-2.50	7.12	34.73	0.0001	0.0042
21 days	twi_ss.14576.1|g.19038	2.74	6.88	34.68	0.0001	0.0042
21 days	twi_ss.28002.1|g.37275	1.86	6.21	34.64	0.0001	0.0042
21 days	twi_ss.2284a.1|g.4027	0.74	8.85	34.60	0.0001	0.0043
21 days	twi_ss.18583.1|g.23690	-4.02	5.85	34.56	0.0001	0.0043
21 days	twi_ss.27814.1|g.37026	-1.75	5.37	34.56	0.0001	0.0043
21 days	twi_ss.31424b.2|g.42777	-5.13	4.29	34.54	0.0001	0.0043
21 days	twi_ss.21074.1|g.27242	1.12	8.17	34.45	0.0001	0.0043
21 days	twi_ss.24962.1|g.32827	1.36	5.87	34.45	0.0001	0.0043
21 days	twi_ss.20998.1|g.27114	-2.13	4.34	34.32	0.0001	0.0044
21 days	twi_ss.24136.1|g.31452	1.73	6.50	34.32	0.0001	0.0044
21 days	twi_ss.22466.3|g.29033	1.25	9.03	34.28	0.0001	0.0044
21 days	twi_ss.9415.1|g.12656	-3.29	3.71	34.26	0.0001	0.0044
21 days	twi_ss.2269.2|g.4022	-2.76	4.47	34.24	0.0001	0.0044
21 days	twi_ss.25312.1|g.33265	3.09	5.02	34.23	0.0001	0.0044
21 days	twi_ss.29085.2|g.39000	-1.71	6.81	34.21	0.0001	0.0044
21 days	twi_ss.16055.1|g.20656	-4.15	4.03	34.17	0.0001	0.0044
21 days	twi_ss.23264.1|g.29908	-1.10	6.66	34.17	0.0001	0.0044
21 days	twi_ss.15686.1|g.20257	-1.92	4.88	34.14	0.0001	0.0044
21 days	twi_ss.19899.1|g.25651	1.54	6.74	34.11	0.0001	0.0044
21 days	twi_ss.11539b.4|g.15321	4.29	8.46	34.03	0.0001	0.0045
21 days	twi_ss.21054.1|g.27163	-2.03	5.81	33.89	0.0001	0.0045
21 days	twi_ss.7858.1|g.10912	0.91	8.17	33.89	0.0001	0.0045
21 days	twi_ss.23961.1|g.31180	1.53	6.32	33.86	0.0001	0.0045
21 days	twi_ss.22606.1|g.29188	-3.74	5.73	33.81	0.0001	0.0046

21 days	twi_ss.27012.1|g.35895	3.13	5.07	33.82	0.0001	0.0046
21 days	twi_ss.21060b.2|g.27174	-1.77	5.07	33.76	0.0001	0.0046
21 days	twi_ss.19853.1|g.25516	0.89	7.65	33.75	0.0001	0.0046
21 days	twi_ss.25742.1|g.33929	1.92	4.74	33.71	0.0001	0.0046
21 days	twi_ss.7489.1|g.10522	-4.17	5.65	33.69	0.0001	0.0046
21 days	twi_ss.25733.1|g.33931	-4.04	4.16	33.58	0.0001	0.0047
21 days	twi_ss.718.1|g.1252	-11.55	7.23	42.40	0.0001	0.0047
21 days	twi_ss.24851.1|g.32717	-5.16	4.92	33.53	0.0002	0.0047
21 days	twi_ss.31796.1|g.43314	-2.85	8.77	33.54	0.0002	0.0047
21 days	twi_ss.3631.1|g.6004	-2.71	4.60	33.53	0.0002	0.0047
21 days	twi_ss.30366.1|g.41072	-4.15	3.77	33.49	0.0002	0.0047
21 days	twi_ss.12248a.3|g.16318	1.39	7.10	33.47	0.0002	0.0047
21 days	twi_ss.18321.1|g.23341	-4.73	4.48	33.45	0.0002	0.0047
21 days	twi_ss.14884.1|g.19318	1.23	6.30	33.43	0.0002	0.0047
21 days	twi_ss.28459.2|g.38108	-6.38	3.65	33.43	0.0002	0.0047
21 days	twi_ss.16144.1|g.20741	-1.37	8.99	33.40	0.0002	0.0047
21 days	twi_ss.30978.1|g.41821	0.99	5.98	33.40	0.0002	0.0047
21 days	twi_ss.1785.1|g.3176	1.50	5.98	33.36	0.0002	0.0047
21 days	twi_ss.30372.1|g.41048	-3.13	4.59	33.35	0.0002	0.0047
21 days	twi_ss.24430.1|g.32014	-4.47	5.56	33.31	0.0002	0.0048
21 days	twi_ss.28686.1|g.38419	-2.66	4.71	33.31	0.0002	0.0048
21 days	twi_ss.3274.1|g.5683	1.12	6.69	33.32	0.0002	0.0048
21 days	twi_ss.6031.1|g.8907	-1.60	7.22	33.29	0.0002	0.0048
21 days	twi_ss.6365.1|g.9392	1.74	8.35	33.28	0.0002	0.0048
21 days	twi_ss.25926.1|g.34242	-1.87	5.49	33.25	0.0002	0.0048
21 days	twi_ss.11938.1|g.15800	-2.30	5.45	33.20	0.0002	0.0048
21 days	twi_ss.14354.1|g.18661	2.12	8.80	33.20	0.0002	0.0048
21 days	twi_ss.26533.1|g.35199	1.71	5.82	33.14	0.0002	0.0048
21 days	twi_ss.20341.1|g.26189	-4.15	4.63	33.11	0.0002	0.0048
21 days	twi_ss.7858.3|g.10907	2.22	6.05	33.12	0.0002	0.0048
21 days	twi_ss.6933.1|g.9993	1.33	5.35	33.06	0.0002	0.0049
21 days	twi_ss.17841.1|g.22744	-1.88	5.74	33.04	0.0002	0.0049
21 days	twi_ss.27783a.2|g.36942	-1.29	7.56	33.04	0.0002	0.0049

21 days	twi_ss.6365.3|g.9393	1.33	5.63	33.01	0.0002	0.0049
21 days	twi_ss.29994.1|g.40475	2.16	7.77	32.98	0.0002	0.0049
21 days	twi_ss.19415.1|g.24943	4.62	4.02	32.86	0.0002	0.0049
21 days	twi_ss.14288.1|g.18566	0.84	9.63	32.81	0.0002	0.0050
21 days	twi_ss.27291.1|g.36220	-2.08	4.56	32.78	0.0002	0.0050
21 days	twi_ss.11428.1|g.15070	1.06	6.23	32.67	0.0002	0.0050
21 days	twi_ss.3292.1|g.5696	-0.90	7.35	32.65	0.0002	0.0051
21 days	twi_ss.5404.1|g.8149	-1.18	5.66	32.62	0.0002	0.0051
21 days	twi_ss.30342.1|g.41041	-1.90	6.54	32.57	0.0002	0.0051
21 days	twi_ss.32065.2|g.43721	4.86	6.06	32.58	0.0002	0.0051
21 days	twi_ss.29027.1|g.38937	1.88	5.34	32.54	0.0002	0.0051
21 days	twi_ss.31441.1|g.42801	-2.89	6.16	32.54	0.0002	0.0051
21 days	twi_ss.1470.1|g.2606	-2.91	4.38	32.52	0.0002	0.0051
21 days	twi_ss.971b.5|g.1737	-7.06	3.84	40.85	0.0002	0.0051
21 days	twi_ss.9652.1|g.12945	-5.41	4.17	32.46	0.0002	0.0051
21 days	twi_ss.24463.1|g.32052	-2.38	5.90	32.39	0.0002	0.0052
21 days	twi_ss.22271.1|g.28809	1.17	6.95	32.37	0.0002	0.0052
21 days	twi_ss.4279.1|g.6783	1.91	6.30	32.38	0.0002	0.0052
21 days	twi_ss.24497.2|g.32088	3.52	5.08	32.35	0.0002	0.0052
21 days	twi_ss.117.1|g.209	0.82	6.91	32.33	0.0002	0.0052
21 days	twi_ss.14178.1|g.18455	-1.64	8.45	32.33	0.0002	0.0052
21 days	twi_ss.13807.1|g.18125	2.57	7.05	32.31	0.0002	0.0052
21 days	twi_ss.9085.1|g.12246	-5.85	3.93	40.60	0.0002	0.0052
21 days	twi_ss.10335.1|g.13873	-2.25	3.95	32.25	0.0002	0.0052
21 days	twi_ss.25771b.1|g.33558	-2.51	8.46	32.25	0.0002	0.0052
21 days	twi_ss.28025.1|g.37315	-1.76	5.73	32.24	0.0002	0.0052
21 days	twi_ss.31636.1|g.43103	1.50	6.90	32.26	0.0002	0.0052
21 days	twi_ss.5045.1|g.7648	-4.51	4.81	32.25	0.0002	0.0052
21 days	twi_ss.24846a.1|g.32671	-1.60	6.44	32.21	0.0002	0.0052
21 days	twi_ss.5147.1|g.7787	-2.56	4.51	32.19	0.0002	0.0052
21 days	twi_ss.9193.1|g.12381	-3.57	4.31	32.18	0.0002	0.0052
21 days	twi_ss.19741a.2|g.25399	-3.47	3.42	32.15	0.0002	0.0052
21 days	twi_ss.22941.1|g.29608	-4.04	3.75	32.15	0.0002	0.0052

21 days	twi_ss.9407.1|g.12694	-1.84	4.42	32.13	0.0002	0.0053
21 days	twi_ss.31893.1|g.43466	-3.93	4.83	32.12	0.0002	0.0053
21 days	twi_ss.29526.1|g.39822	-1.57	5.99	31.92	0.0002	0.0054
21 days	twi_ss.30229.1|g.40845	0.78	9.20	31.92	0.0002	0.0054
21 days	twi_ss.21748.1|g.28212	-2.96	4.40	31.90	0.0002	0.0054
21 days	twi_ss.28866.1|g.38753	4.01	4.51	31.87	0.0002	0.0054
21 days	twi_ss.3244.1|g.5651	-1.72	5.73	31.87	0.0002	0.0054
21 days	twi_ss.12099.1|g.16051	0.62	9.07	31.79	0.0002	0.0054
21 days	twi_ss.29133.1|g.39099	1.07	5.51	31.79	0.0002	0.0054
21 days	twi_ss.31288.1|g.42543	-5.82	4.01	39.86	0.0002	0.0054
21 days	twi_ss.26704b.1|g.35420	1.14	10.59	31.77	0.0002	0.0054
21 days	twi_ss.9529.1|g.12779	4.99	2.47	39.82	0.0002	0.0054
21 days	twi_ss.1474a.1|g.2550	0.96	5.86	31.76	0.0002	0.0054
21 days	twi_ss.21289.1|g.27542	0.91	7.64	31.73	0.0002	0.0055
21 days	twi_ss.29443.2|g.39650	2.13	6.06	31.65	0.0002	0.0055
21 days	twi_ss.308a.2|g.609	-1.36	5.46	31.63	0.0002	0.0055
21 days	twi_ss.29623.1|g.39903	-3.06	5.62	31.61	0.0002	0.0055
21 days	twi_ss.28156a.1|g.37511	-9.27	6.91	39.57	0.0002	0.0055
21 days	twi_ss.10196.1|g.13703	0.87	7.16	31.58	0.0002	0.0055
21 days	twi_ss.16264.1|g.20846	1.03	6.35	31.42	0.0002	0.0056
21 days	twi_ss.12733.1|g.16821	2.61	4.41	31.40	0.0002	0.0057
21 days	twi_ss.20751.1|g.26836	0.97	7.11	31.39	0.0002	0.0057
21 days	twi_ss.27071b.2|g.35979	-1.11	8.03	31.37	0.0002	0.0057
21 days	twi_ss.14947.1|g.19373	-1.95	5.13	31.31	0.0002	0.0057
21 days	twi_ss.8364b.1|g.11477	-1.52	5.59	31.27	0.0002	0.0057
21 days	twi_ss.1798a.1|g.3194	-3.77	5.15	31.24	0.0002	0.0057
21 days	twi_ss.13566.1|g.17913	2.37	8.08	31.19	0.0002	0.0058
21 days	twi_ss.22791.1|g.29420	-3.12	4.45	31.11	0.0002	0.0058
21 days	twi_ss.24823.1|g.32552	2.54	5.26	31.11	0.0002	0.0058
21 days	twi_ss.5080.1|g.7730	-2.77	4.71	31.03	0.0002	0.0059
21 days	twi_ss.15849.1|g.20416	2.45	5.05	30.95	0.0002	0.0059
21 days	twi_ss.4992.1|g.7612	2.27	4.84	30.93	0.0002	0.0059
21 days	twi_ss.1632.1|g.2874	-2.28	4.52	30.92	0.0002	0.0060

21 days	twi_ss.9412.1|g.12748	-5.26	4.23	30.90	0.0002	0.0060
21 days	twi_ss.6577.1|g.9613	-5.91	3.72	38.56	0.0002	0.0060
21 days	twi_ss.27614.1|g.36797	-3.85	5.18	30.87	0.0002	0.0060
21 days	twi_ss.25669.1|g.33849	-2.28	4.53	30.84	0.0002	0.0060
21 days	twi_ss.2009.1|g.3633	0.97	7.43	30.82	0.0002	0.0060
21 days	twi_ss.28123.1|g.37442	-1.91	5.07	30.81	0.0002	0.0060
21 days	twi_ss.25813.1|g.34079	2.60	4.67	30.77	0.0002	0.0060
21 days	twi_ss.12038.1|g.15928	0.85	8.37	30.76	0.0002	0.0060
21 days	twi_ss.28605.1|g.38302	-5.73	4.94	30.72	0.0002	0.0061
21 days	twi_ss.14945a.1|g.19407	-1.91	5.11	30.68	0.0002	0.0061
21 days	twi_ss.13791.1|g.18111	2.10	6.00	30.67	0.0002	0.0061
21 days	twi_ss.15425.1|g.19949	-1.91	5.26	30.65	0.0002	0.0061
21 days	twi_ss.22712.1|g.29343	-3.68	8.18	30.65	0.0002	0.0061
21 days	twi_ss.6182.1|g.9161	-2.77	8.14	30.64	0.0002	0.0061
21 days	twi_ss.29544b.2|g.39679	1.41	6.83	30.56	0.0002	0.0061
21 days	twi_ss.25621.4|g.33682	-1.05	6.33	30.53	0.0002	0.0062
21 days	twi_ss.26999.1|g.35881	-1.15	6.93	30.50	0.0002	0.0062
21 days	twi_ss.31432.1|g.42807	-2.45	10.68	30.50	0.0002	0.0062
21 days	twi_ss.24183.1|g.31560	-6.09	5.27	30.49	0.0002	0.0062
21 days	twi_ss.24546.1|g.32194	-1.86	6.35	30.44	0.0002	0.0062
21 days	twi_ss.28476.1|g.38101	1.18	6.68	30.45	0.0002	0.0062
21 days	twi_ss.15332.1|g.19854	-0.67	9.12	30.44	0.0002	0.0062
21 days	twi_ss.10479.1|g.14122	2.51	5.12	30.42	0.0002	0.0062
21 days	twi_ss.7966.1|g.11006	-2.25	5.06	30.42	0.0002	0.0062
21 days	twi_ss.24497.5|g.32090	6.38	3.34	30.41	0.0002	0.0062
21 days	twi_ss.25307.1|g.33261	0.88	6.28	30.39	0.0002	0.0062
21 days	twi_ss.25520.1|g.33549	-4.14	3.71	30.37	0.0002	0.0062
21 days	twi_ss.1075b.3|g.1936	0.83	6.62	30.36	0.0002	0.0062
21 days	twi_ss.1493b.1|g.2649	0.97	7.26	30.35	0.0002	0.0062
21 days	twi_ss.12094a.1|g.16001	-3.36	7.55	30.30	0.0002	0.0063
21 days	twi_ss.17576.1|g.22475	-2.91	5.59	30.28	0.0002	0.0063
21 days	twi_ss.2005.1|g.3565	1.34	5.20	30.26	0.0002	0.0063
21 days	twi_ss.2005.2|g.3573	1.34	5.20	30.26	0.0002	0.0063

21 days	twi_ss.2005.3|g.3577	1.34	5.20	30.26	0.0002	0.0063
21 days	twi_ss.24586.1|g.32228	-1.40	5.32	30.21	0.0002	0.0063
21 days	twi_ss.30173.6|g.40745	-1.67	5.06	30.18	0.0002	0.0063
21 days	twi_ss.6592.1|g.9620	-3.87	5.13	30.18	0.0002	0.0063
21 days	twi_ss.1119.1|g.1990	0.87	7.53	30.15	0.0002	0.0064
21 days	twi_ss.28038b.2|g.37332	4.60	4.50	30.12	0.0002	0.0064
21 days	twi_ss.16821.1|g.21482	-4.29	4.87	30.08	0.0002	0.0064
21 days	twi_ss.28804.1|g.38536	2.01	5.54	30.05	0.0002	0.0064
21 days	twi_ss.21742.1|g.28168	0.79	7.82	30.04	0.0002	0.0064
21 days	twi_ss.23801.2|g.30746	1.31	7.41	30.00	0.0002	0.0064
21 days	twi_ss.19577.1|g.25087	0.90	8.80	29.97	0.0002	0.0065
21 days	twi_ss.1904.3|g.3390	1.18	6.35	29.92	0.0002	0.0065
21 days	twi_ss.29675.1|g.39998	-2.63	6.80	29.93	0.0002	0.0065
21 days	twi_ss.29908.1|g.40336	-1.60	4.69	29.81	0.0002	0.0066
21 days	twi_ss.21950.1|g.28445	0.72	7.32	29.73	0.0002	0.0067
21 days	twi_ss.30095.1|g.40635	1.35	6.19	29.65	0.0002	0.0067
21 days	twi_ss.21653.1|g.28054	-3.12	4.61	29.64	0.0002	0.0067
21 days	twi_ss.18548.1|g.23653	-2.31	5.37	29.60	0.0002	0.0068
21 days	twi_ss.20349.1|g.26257	-5.85	3.07	36.59	0.0003	0.0068
21 days	twi_ss.2820.1|g.5093	-1.37	5.23	29.49	0.0003	0.0068
21 days	twi_ss.8220.1|g.11303	-2.49	4.38	29.43	0.0003	0.0069
21 days	twi_ss.12133.1|g.16028	-2.11	5.23	29.41	0.0003	0.0069
21 days	twi_ss.17531a.2|g.22385	0.96	5.96	29.42	0.0003	0.0069
21 days	twi_ss.26323.1|g.34866	1.50	6.74	29.40	0.0003	0.0069
21 days	twi_ss.28262.1|g.37804	-2.72	4.07	29.36	0.0003	0.0069
21 days	twi_ss.8565.1|g.11724	-5.40	3.88	29.36	0.0003	0.0069
21 days	twi_ss.14231.1|g.18498	-1.77	5.07	29.29	0.0003	0.0070
21 days	twi_ss.17370.1|g.22146	1.50	6.10	29.29	0.0003	0.0070
21 days	twi_ss.6415.1|g.9461	-1.22	8.11	29.27	0.0003	0.0070
21 days	twi_ss.8188b.1|g.11272	-2.84	3.50	29.27	0.0003	0.0070
21 days	twi_ss.29617.1|g.39880	2.75	9.59	29.25	0.0003	0.0070
21 days	twi_ss.27623.5|g.36631	-2.04	5.99	29.24	0.0003	0.0070
21 days	twi_ss.12389.1|g.16515	-3.94	3.47	29.21	0.0003	0.0070

21 days	twi_ss.18468.1|g.23509	-0.90	6.98	29.21	0.0003	0.0070
21 days	twi_ss.22999.1|g.29646	3.33	3.97	29.21	0.0003	0.0070
21 days	twi_ss.26266.1|g.34794	0.71	7.08	29.22	0.0003	0.0070
21 days	twi_ss.923.1|g.1720	-2.83	6.81	29.20	0.0003	0.0070
21 days	twi_ss.7040.1|g.10101	-5.06	3.19	36.09	0.0003	0.0071
21 days	twi_ss.18762.1|g.23959	-2.24	4.79	29.12	0.0003	0.0071
21 days	twi_ss.15844b.3|g.20393	6.42	3.96	29.04	0.0003	0.0071
21 days	twi_ss.29997.1|g.40469	-1.95	5.99	29.04	0.0003	0.0071
21 days	twi_ss.1801.1|g.3187	1.67	6.16	29.01	0.0003	0.0072
21 days	twi_ss.17219a.12|g.21775	0.56	9.25	29.00	0.0003	0.0072
21 days	twi_ss.19210.1|g.24594	0.62	8.35	28.98	0.0003	0.0072
21 days	twi_ss.3516.1|g.5892	-2.75	3.99	28.96	0.0003	0.0072
21 days	twi_ss.24550.1|g.32081	-2.39	8.64	28.88	0.0003	0.0073
21 days	twi_ss.29330.1|g.39379	0.98	7.58	28.86	0.0003	0.0073
21 days	twi_ss.8537.1|g.11696	-1.20	6.90	28.86	0.0003	0.0073
21 days	twi_ss.24393.1|g.31956	1.30	5.67	28.82	0.0003	0.0073
21 days	twi_ss.28580.1|g.38266	0.90	7.17	28.82	0.0003	0.0073
21 days	twi_ss.9553.1|g.12824	-3.85	3.44	28.82	0.0003	0.0073
21 days	twi_ss.16037.1|g.20641	-2.35	5.23	28.81	0.0003	0.0073
21 days	twi_ss.2240.1|g.3947	2.48	5.35	28.80	0.0003	0.0073
21 days	twi_ss.30491.1|g.41266	1.58	5.67	28.79	0.0003	0.0073
21 days	twi_ss.4825.1|g.7403	-2.10	4.72	28.77	0.0003	0.0073
21 days	twi_ss.30423.1|g.41204	-1.61	7.12	28.76	0.0003	0.0073
21 days	twi_ss.22326.1|g.28866	-4.27	3.60	28.75	0.0003	0.0073
21 days	twi_ss.23705.1|g.30520	-1.39	6.01	28.74	0.0003	0.0073
21 days	twi_ss.32112.1|g.43770	-1.30	5.79	28.73	0.0003	0.0073
21 days	twi_ss.22572.1|g.29155	-3.50	5.01	28.72	0.0003	0.0073
21 days	twi_ss.27729.1|g.36923	2.69	5.81	28.71	0.0003	0.0073
21 days	twi_ss.4660.1|g.7190	0.99	7.39	28.71	0.0003	0.0073
21 days	twi_ss.18852.1|g.24126	-6.27	5.40	28.70	0.0003	0.0073
21 days	twi_ss.4854.1|g.7451	-2.26	6.00	28.62	0.0003	0.0074
21 days	twi_ss.29223.2|g.39194	-5.78	3.92	28.57	0.0003	0.0074
21 days	twi_ss.173.1|g.271	0.81	8.46	28.54	0.0003	0.0075

21 days	twi_ss.30101a.1|g.40628	-3.03	4.00	28.52	0.0003	0.0075
21 days	twi_ss.28940.1|g.38796	-7.09	3.43	35.23	0.0003	0.0075
21 days	twi_ss.10227.1|g.13742	1.23	5.40	28.44	0.0003	0.0075
21 days	twi_ss.26.1|g.114	1.39	5.65	28.44	0.0003	0.0075
21 days	twi_ss.31558.1|g.42985	-1.45	7.76	28.44	0.0003	0.0075
21 days	twi_ss.2194.1|g.3904	1.22	6.38	28.42	0.0003	0.0076
21 days	twi_ss.32065.1|g.43717	4.48	6.08	28.41	0.0003	0.0076
21 days	twi_ss.161.1|g.272	-4.61	4.56	28.40	0.0003	0.0076
21 days	twi_ss.19158.1|g.24558	-5.10	7.36	28.39	0.0003	0.0076
21 days	twi_ss.20446.1|g.26335	-0.91	6.20	28.39	0.0003	0.0076
21 days	twi_ss.1346.1|g.2430	-2.87	4.25	28.37	0.0003	0.0076
21 days	twi_ss.14580.1|g.19046	1.46	5.46	28.36	0.0003	0.0076
21 days	twi_ss.21150.1|g.27373	-1.72	6.07	28.32	0.0003	0.0076
21 days	twi_ss.29213.1|g.39249	-1.25	5.88	28.32	0.0003	0.0076
21 days	twi_ss.30768.1|g.41766	1.51	4.82	28.32	0.0003	0.0076
21 days	twi_ss.5298a.2|g.8044	0.98	6.43	28.33	0.0003	0.0076
21 days	twi_ss.18417.1|g.23456	-5.81	3.57	34.90	0.0003	0.0076
21 days	twi_ss.18417.2|g.23458	-5.81	3.57	34.90	0.0003	0.0076
21 days	twi_ss.7711.1|g.10773	1.70	5.80	28.26	0.0003	0.0076
21 days	twi_ss.1343.1|g.2422	0.49	8.24	28.24	0.0003	0.0076
21 days	twi_ss.4928.2|g.7570	1.21	6.58	28.24	0.0003	0.0076
21 days	twi_ss.9256b.1|g.12481	1.34	5.82	28.24	0.0003	0.0076
21 days	twi_ss.31037.1|g.42133	1.25	5.86	28.22	0.0003	0.0077
21 days	twi_ss.17708.1|g.22577	-2.97	4.17	28.20	0.0003	0.0077
21 days	twi_ss.30160.1|g.40720	0.85	7.65	28.21	0.0003	0.0077
21 days	twi_ss.14928.1|g.19441	-5.53	2.98	34.76	0.0003	0.0077
21 days	twi_ss.10102.1|g.13565	-2.86	4.21	28.12	0.0003	0.0077
21 days	twi_ss.13162.3|g.17442	1.11	6.18	28.13	0.0003	0.0077
21 days	twi_ss.1957a.1|g.3427	0.78	7.70	28.10	0.0003	0.0077
21 days	twi_ss.2422.1|g.4431	3.31	5.64	28.08	0.0003	0.0077
21 days	twi_ss.24554.1|g.32097	1.90	4.92	28.09	0.0003	0.0077
21 days	twi_ss.25650.1|g.33834	-1.44	4.70	28.08	0.0003	0.0077
21 days	twi_ss.27562.1|g.36720	-2.90	6.47	28.12	0.0003	0.0077

21 days	twi_ss.28802.1|g.38598	-2.17	4.26	28.14	0.0003	0.0077
21 days	twi_ss.31443.1|g.42802	-1.98	5.02	28.09	0.0003	0.0077
21 days	twi_ss.6244a.2|g.9240	-3.46	4.39	28.09	0.0003	0.0077
21 days	twi_ss.6438.1|g.9482	-2.15	5.03	28.11	0.0003	0.0077
21 days	twi_ss.19688.1|g.25275	-1.75	4.75	28.05	0.0003	0.0077
21 days	twi_ss.8573.1|g.11722	3.51	3.60	28.05	0.0003	0.0077
21 days	twi_ss.17390.1|g.22174	-2.88	7.61	28.02	0.0003	0.0078
21 days	twi_ss.21600.1|g.27981	3.65	7.04	27.97	0.0003	0.0078
21 days	twi_ss.31964.1|g.43602	-2.95	4.21	27.97	0.0003	0.0078
21 days	twi_ss.14254.1|g.18567	-2.86	4.76	27.95	0.0003	0.0078
21 days	twi_ss.30153.2|g.40650	1.31	5.85	27.94	0.0003	0.0078
21 days	twi_ss.6573b.1|g.9605	-2.72	4.79	27.93	0.0003	0.0078
21 days	twi_ss.10376.1|g.14023	1.57	5.44	27.92	0.0003	0.0078
21 days	twi_ss.28187.2|g.37570	-8.08	4.09	27.92	0.0003	0.0078
21 days	twi_ss.16738.1|g.21427	1.11	6.91	27.90	0.0003	0.0078
21 days	twi_ss.1686.1|g.2967	-1.93	4.59	27.88	0.0003	0.0079
21 days	twi_ss.32017.1|g.43662	-7.65	3.74	34.34	0.0003	0.0079
21 days	twi_ss.15647.3|g.20218	6.23	2.76	27.86	0.0003	0.0079
21 days	twi_ss.4555.1|g.7078	-2.18	6.05	27.84	0.0003	0.0079
21 days	twi_ss.981.1|g.1783	4.65	5.46	27.84	0.0003	0.0079
21 days	twi_ss.7791.1|g.10881	0.92	7.26	27.83	0.0003	0.0079
21 days	twi_ss.14116a.1|g.18422	1.58	6.53	27.81	0.0003	0.0079
21 days	twi_ss.9953.1|g.13282	-2.23	4.17	27.81	0.0003	0.0079
21 days	twi_ss.9457.1|g.12743	0.95	6.72	27.80	0.0003	0.0079
21 days	twi_ss.24700.1|g.32390	1.01	6.15	27.74	0.0003	0.0080
21 days	twi_ss.3491.1|g.5867	1.72	4.48	27.73	0.0003	0.0080
21 days	twi_ss.10068.1|g.13381	9.01	4.93	27.69	0.0003	0.0080
21 days	twi_ss.11909.1|g.15789	2.09	5.69	27.65	0.0003	0.0080
21 days	twi_ss.16157.1|g.20755	-5.84	3.98	27.65	0.0003	0.0080
21 days	twi_ss.19499.1|g.25032	1.31	5.25	27.67	0.0003	0.0080
21 days	twi_ss.26477.1|g.35088	-3.29	3.96	27.67	0.0003	0.0080
21 days	twi_ss.27810.1|g.37027	-5.66	5.08	27.65	0.0003	0.0080
21 days	twi_ss.27827b.1|g.37022	-1.78	6.12	27.65	0.0003	0.0080

21 days	twi_ss.29852.1|g.40264	-3.73	4.32	27.68	0.0003	0.0080
21 days	twi_ss.9109.1|g.12292	0.93	6.01	27.64	0.0003	0.0080
21 days	twi_ss.24967.1|g.32796	4.04	7.66	27.62	0.0003	0.0080
21 days	twi_ss.26672b.1|g.35349	-2.48	4.89	27.62	0.0003	0.0080
21 days	twi_ss.24043.1|g.31303	-3.21	4.50	27.61	0.0003	0.0080
21 days	twi_ss.9430.1|g.12699	3.22	4.18	27.59	0.0003	0.0080
21 days	twi_ss.21606.1|g.28005	1.14	6.83	27.56	0.0003	0.0081
21 days	twi_ss.30292.1|g.40927	-4.04	3.81	27.56	0.0003	0.0081
21 days	twi_ss.18124.1|g.23044	-4.43	3.79	27.54	0.0003	0.0081
21 days	twi_ss.28295b.2|g.37937	-4.10	4.73	27.52	0.0003	0.0081
21 days	twi_ss.20162.1|g.25923	-6.88	3.23	33.80	0.0003	0.0081
21 days	twi_ss.26937.1|g.35757	-1.66	4.63	27.48	0.0003	0.0081
21 days	twi_ss.8660.1|g.11799	-1.35	6.16	27.50	0.0003	0.0081
21 days	twi_ss.9709.1|g.13070	6.28	3.43	27.48	0.0003	0.0081
21 days	twi_ss.168.2|g.251	-4.82	4.44	27.47	0.0003	0.0081
21 days	twi_ss.974.3|g.1751	6.72	2.98	27.41	0.0003	0.0082
21 days	twi_ss.8930a.2|g.12125	-5.69	2.87	33.66	0.0003	0.0082
21 days	twi_ss.24627.2|g.32262	6.90	3.56	27.37	0.0003	0.0082
21 days	twi_ss.29715.1|g.40145	-1.13	5.92	27.34	0.0003	0.0082
21 days	twi_ss.3976.1|g.6397	-2.52	4.71	27.34	0.0003	0.0082
21 days	twi_ss.17786.1|g.22684	-4.25	4.35	27.33	0.0003	0.0082
21 days	twi_ss.20955.1|g.27087	1.61	5.35	27.32	0.0003	0.0082
21 days	twi_ss.17946.1|g.22825	-2.67	4.21	27.32	0.0003	0.0082
21 days	twi_ss.15844b.4|g.20394	-8.01	4.59	27.28	0.0003	0.0083
21 days	twi_ss.19345.12|g.24807	-6.59	2.90	47.77	0.0003	0.0083
21 days	twi_ss.27413.1|g.36501	-3.37	4.31	27.27	0.0003	0.0083
21 days	twi_ss.29853.1|g.40256	-2.51	5.96	27.28	0.0003	0.0083
21 days	twi_ss.5329.1|g.7937	-1.26	7.79	27.26	0.0003	0.0083
21 days	twi_ss.28752.1|g.38495	1.33	6.53	27.21	0.0003	0.0083
21 days	twi_ss.30965.1|g.41816	-1.32	5.51	27.18	0.0003	0.0083
21 days	twi_ss.480.3|g.867	-4.03	5.01	27.19	0.0003	0.0083
21 days	twi_ss.19231.1|g.24664	-2.80	4.52	27.18	0.0003	0.0083
21 days	twi_ss.7122b.2|g.10135	-1.54	7.59	27.15	0.0003	0.0084

21 days	twi_ss.12505a.1|g.16597	0.91	6.85	27.12	0.0003	0.0084
21 days	twi_ss.25649.1|g.33858	-4.43	3.80	27.12	0.0003	0.0084
21 days	twi_ss.9786.1|g.13142	-1.62	5.71	27.09	0.0004	0.0084
21 days	twi_ss.2348.1|g.4257	-4.14	4.12	27.06	0.0004	0.0084
21 days	twi_ss.31915.5|g.43431	0.99	8.46	27.01	0.0004	0.0085
21 days	twi_ss.8150.1|g.11239	1.30	6.70	27.00	0.0004	0.0085
21 days	twi_ss.12972.1|g.17144	2.14	4.99	26.99	0.0004	0.0085
21 days	twi_ss.18250.1|g.23153	2.80	7.02	26.98	0.0004	0.0085
21 days	twi_ss.2483.2|g.4532	7.15	3.31	26.96	0.0004	0.0085
21 days	twi_ss.1269.1|g.2281	-7.36	3.87	33.04	0.0004	0.0086
21 days	twi_ss.12013.1|g.15876	1.34	6.28	26.90	0.0004	0.0086
21 days	twi_ss.18066.1|g.22951	0.86	6.41	26.89	0.0004	0.0086
21 days	twi_ss.22201b.2|g.28772	-6.06	3.01	32.97	0.0004	0.0086
21 days	twi_ss.17159.1|g.21925	2.25	4.06	26.88	0.0004	0.0086
21 days	twi_ss.30966.1|g.42009	-3.31	5.45	26.86	0.0004	0.0086
21 days	twi_ss.29504.3|g.39728	5.26	2.72	32.92	0.0004	0.0086
21 days	twi_ss.30809.1|g.41716	1.93	5.84	26.82	0.0004	0.0087
21 days	twi_ss.30031.1|g.40544	-1.57	5.60	26.81	0.0004	0.0087
21 days	twi_ss.21312.1|g.27547	0.92	8.48	26.78	0.0004	0.0087
21 days	twi_ss.22308.1|g.28854	-6.35	3.23	46.58	0.0004	0.0087
21 days	twi_ss.26264.1|g.34774	1.21	5.52	26.77	0.0004	0.0087
21 days	twi_ss.18485.1|g.23547	-5.76	3.06	32.76	0.0004	0.0087
21 days	twi_ss.8878b.2|g.12094	-4.41	3.67	26.74	0.0004	0.0087
21 days	twi_ss.10365.1|g.13990	7.93	4.46	26.71	0.0004	0.0087
21 days	twi_ss.14938.1|g.19454	-6.85	3.30	32.71	0.0004	0.0088
21 days	twi_ss.1430.1|g.2572	0.62	8.32	26.67	0.0004	0.0088
21 days	twi_ss.7443.1|g.10451	2.26	4.01	26.67	0.0004	0.0088
21 days	twi_ss.3881.1|g.6304	1.23	5.65	26.62	0.0004	0.0088
21 days	twi_ss.10285c.9|g.13768	-1.88	6.03	26.58	0.0004	0.0089
21 days	twi_ss.19488.1|g.25029	-1.40	7.09	26.55	0.0004	0.0089
21 days	twi_ss.23102.1|g.29733	-1.42	5.77	26.55	0.0004	0.0089
21 days	twi_ss.22992.1|g.29637	-2.41	5.79	26.53	0.0004	0.0089
21 days	twi_ss.9821.1|g.13111	0.84	6.82	26.51	0.0004	0.0089

21 days	twi_ss.32070.1|g.43737	-0.90	7.43	26.49	0.0004	0.0090
21 days	twi_ss.26090a.1|g.34602	-5.26	5.73	26.48	0.0004	0.0090
21 days	twi_ss.28335.1|g.37855	-2.31	4.86	26.47	0.0004	0.0090
21 days	twi_ss.11112.1|g.14736	-3.72	4.76	26.45	0.0004	0.0090
21 days	twi_ss.28292.4|g.37668	-3.32	5.17	26.46	0.0004	0.0090
21 days	twi_ss.7793.1|g.10869	0.85	8.07	26.44	0.0004	0.0090
21 days	twi_ss.4477.1|g.6994	4.94	5.40	26.39	0.0004	0.0090
21 days	twi_ss.18006.1|g.22906	1.59	4.73	26.38	0.0004	0.0090
21 days	twi_ss.23617.1|g.30394	-4.17	4.50	26.38	0.0004	0.0090
21 days	twi_ss.982c.1|g.1756	1.82	6.42	26.38	0.0004	0.0090
21 days	twi_ss.15762.1|g.20339	-5.60	3.87	26.36	0.0004	0.0091
21 days	twi_ss.31609.1|g.43064	-2.86	6.29	26.33	0.0004	0.0091
21 days	twi_ss.31663.1|g.43109	-1.39	9.16	26.32	0.0004	0.0091
21 days	twi_ss.2395.1|g.4284	2.43	3.68	26.32	0.0004	0.0091
21 days	twi_ss.30226.1|g.40823	0.60	7.99	26.28	0.0004	0.0091
21 days	twi_ss.6155.1|g.9054	-1.53	4.86	26.24	0.0004	0.0092
21 days	twi_ss.16455.1|g.21022	-2.65	5.07	26.20	0.0004	0.0092
21 days	twi_ss.23585.1|g.30340	0.73	6.16	26.20	0.0004	0.0092
21 days	twi_ss.31287.1|g.42554	-5.73	3.96	26.20	0.0004	0.0092
21 days	twi_ss.31539a.2|g.42932	-7.02	3.37	32.02	0.0004	0.0092
21 days	twi_ss.22044.1|g.28559	-4.59	3.74	26.16	0.0004	0.0093
21 days	twi_ss.18363.1|g.23409	1.07	7.05	26.14	0.0004	0.0093
21 days	twi_ss.25151.1|g.33065	-3.75	3.84	26.13	0.0004	0.0093
21 days	twi_ss.4337.1|g.6840	0.98	5.94	26.11	0.0004	0.0093
21 days	twi_ss.7858.12|g.10915	4.38	6.25	26.08	0.0004	0.0093
21 days	twi_ss.19199b.2|g.24597	0.78	6.83	26.07	0.0004	0.0093
21 days	twi_ss.24078.1|g.31307	0.53	7.63	26.04	0.0004	0.0094
21 days	twi_ss.31260.1|g.42493	-1.55	5.11	26.04	0.0004	0.0094
21 days	twi_ss.11848b.2|g.15737	-5.83	3.02	31.78	0.0004	0.0094
21 days	twi_ss.18781.1|g.23962	-0.81	7.23	26.01	0.0004	0.0094
21 days	twi_ss.12272.1|g.16279	-2.64	5.31	26.00	0.0004	0.0094
21 days	twi_ss.22034.1|g.28521	-0.87	6.93	25.99	0.0004	0.0094
21 days	twi_ss.28144.1|g.37496	-1.42	5.42	26.00	0.0004	0.0094

21 days	twi_ss.556.4|g.1001	-4.38	4.89	26.00	0.0004	0.0094
21 days	twi_ss.30416.1|g.41149	-2.75	4.52	25.97	0.0004	0.0094
21 days	twi_ss.1121.1|g.1995	-4.77	2.80	31.70	0.0004	0.0094
21 days	twi_ss.5509.1|g.8289	-4.32	5.30	25.94	0.0004	0.0094
21 days	twi_ss.23142.1|g.29795	-3.20	3.94	25.93	0.0004	0.0095
21 days	twi_ss.7929.1|g.10983	0.84	6.20	25.92	0.0004	0.0095
21 days	twi_ss.18290.1|g.23321	-5.28	2.86	25.88	0.0004	0.0095
21 days	twi_ss.389.1|g.744	-1.49	6.58	25.88	0.0004	0.0095
21 days	twi_ss.31218.1|g.42418	1.67	5.58	25.84	0.0004	0.0095
21 days	twi_ss.1701.1|g.3004	1.04	6.22	25.83	0.0004	0.0096
21 days	twi_ss.19751.1|g.25411	0.48	7.66	25.83	0.0004	0.0096
21 days	twi_ss.2026.1|g.3585	0.59	7.72	25.82	0.0004	0.0096
21 days	twi_ss.23612.1|g.30359	-2.44	7.62	25.79	0.0004	0.0096
21 days	twi_ss.27883.3|g.37126	-1.44	7.04	25.77	0.0004	0.0096
21 days	twi_ss.31831.1|g.43350	-1.69	7.30	25.76	0.0004	0.0096
21 days	twi_ss.469.1|g.851	1.40	5.67	25.74	0.0004	0.0096
21 days	twi_ss.14522.1|g.18995	-2.55	5.24	25.68	0.0004	0.0097
21 days	twi_ss.16921.2|g.21563	-1.10	6.49	25.68	0.0004	0.0097
21 days	twi_ss.19202.1|g.24617	0.72	6.38	25.70	0.0004	0.0097
21 days	twi_ss.6230.1|g.9268	-1.45	5.74	25.68	0.0004	0.0097
21 days	twi_ss.6784.1|g.9724	-1.32	6.73	25.69	0.0004	0.0097
21 days	twi_ss.15520.1|g.20104	-1.89	4.74	25.66	0.0004	0.0097
21 days	twi_ss.29887b.1|g.40353	-2.50	4.73	25.66	0.0004	0.0097
21 days	twi_ss.21288a.1|g.27523	2.37	3.84	25.62	0.0004	0.0098
21 days	twi_ss.29422.1|g.39591	-1.02	6.65	25.61	0.0004	0.0098
21 days	twi_ss.28282.1|g.37778	0.80	6.72	25.58	0.0004	0.0098
21 days	twi_ss.1087.9|g.1904	-1.58	8.01	25.52	0.0004	0.0099
21 days	twi_ss.13046.1|g.17280	-1.47	6.07	25.52	0.0004	0.0099
21 days	twi_ss.14946a.1|g.19352	0.85	6.81	25.52	0.0004	0.0099
21 days	twi_ss.27162.1|g.36069	-1.62	5.86	25.52	0.0004	0.0099
21 days	twi_ss.1007.1|g.1828	-2.67	3.89	25.51	0.0004	0.0099
21 days	twi_ss.2085.1|g.3724	-5.02	4.10	25.50	0.0004	0.0099
21 days	twi_ss.3722.1|g.6107	3.04	3.59	25.50	0.0004	0.0099

21 days	twi_ss.30158.1|g.40726	0.86	7.49	25.48	0.0004	0.0099
21 days	twi_ss.25871.1|g.34134	0.60	7.99	25.43	0.0004	0.0100
21 days	twi_ss.20132.1|g.25897	-2.54	5.94	25.38	0.0004	0.0100
21 days	twi_ss.16705.3|g.21383	-6.57	2.97	25.36	0.0005	0.0100
21 days	twi_ss.26494.2|g.35156	-4.74	3.22	25.36	0.0005	0.0100
21 days	twi_ss.23554b.4|g.30231	-3.11	9.77	25.34	0.0005	0.0101
21 days	twi_ss.23569.1|g.30338	-2.25	5.55	25.34	0.0005	0.0101
21 days	twi_ss.20312.1|g.26146	1.00	5.49	25.33	0.0005	0.0101
21 days	twi_ss.25370.1|g.33401	-4.19	4.50	25.32	0.0005	0.0101
21 days	twi_ss.22743.10|g.29084	-2.63	4.68	25.31	0.0005	0.0101
21 days	twi_ss.31013.1|g.42205	-3.63	4.95	25.30	0.0005	0.0101
21 days	twi_ss.2291.1|g.4028	-1.78	6.45	25.28	0.0005	0.0101
21 days	twi_ss.4192.1|g.6605	-3.33	5.26	25.29	0.0005	0.0101
21 days	twi_ss.21425.2|g.27686	4.32	4.31	25.26	0.0005	0.0101
21 days	twi_ss.5245.1|g.7897	-3.80	3.55	25.24	0.0005	0.0102
21 days	twi_ss.21655.5|g.28069	-4.14	4.90	25.22	0.0005	0.0102
21 days	twi_ss.29446.3|g.39630	1.60	6.30	25.22	0.0005	0.0102
21 days	twi_ss.12132.1|g.16041	-1.34	4.89	25.20	0.0005	0.0102
21 days	twi_ss.30378.1|g.41083	-0.80	9.87	25.20	0.0005	0.0102
21 days	twi_ss.28055.1|g.37390	0.92	7.97	25.19	0.0005	0.0102
21 days	twi_ss.18795.4|g.24047	2.63	7.57	25.18	0.0005	0.0102
21 days	twi_ss.10017.1|g.13314	4.50	2.25	30.93	0.0005	0.0102
21 days	twi_ss.30481.2|g.41260	1.66	6.73	25.15	0.0005	0.0102
21 days	twi_ss.22400.1|g.28915	-1.13	6.33	25.13	0.0005	0.0102
21 days	twi_ss.30134.5|g.40699	1.52	8.79	25.13	0.0005	0.0102
21 days	twi_ss.8324b.1|g.11420	0.81	6.13	25.13	0.0005	0.0102
21 days	twi_ss.25263.1|g.33243	-2.72	3.45	25.12	0.0005	0.0103
21 days	twi_ss.261.1|g.548	-1.42	5.84	25.07	0.0005	0.0103
21 days	twi_ss.2564.1|g.4613	-6.27	3.53	30.45	0.0005	0.0103
21 days	twi_ss.12986.3|g.17244	-2.34	6.40	25.05	0.0005	0.0103
21 days	twi_ss.10597.1|g.14226	1.05	6.18	25.04	0.0005	0.0103
21 days	twi_ss.24015.2|g.31231	-1.88	4.39	25.03	0.0005	0.0103
21 days	twi_ss.30355.1|g.41052	-1.97	5.61	25.03	0.0005	0.0103

21 days	twi_ss.19570.1|g.25103	-2.82	5.06	25.01	0.0005	0.0104
21 days	twi_ss.6320.1|g.9344	-0.77	6.57	25.01	0.0005	0.0104
21 days	twi_ss.13485.1|g.17844	0.71	7.68	24.97	0.0005	0.0104
21 days	twi_ss.22373.1|g.28900	-1.40	4.78	24.97	0.0005	0.0104
21 days	twi_ss.25939.1|g.34234	-5.40	3.64	24.94	0.0005	0.0104
21 days	twi_ss.249.2|g.539	-2.67	5.64	24.93	0.0005	0.0105
21 days	twi_ss.10999.1|g.14629	-1.45	5.19	24.88	0.0005	0.0105
21 days	twi_ss.22588a.2|g.29168	-1.59	5.11	24.86	0.0005	0.0106
21 days	twi_ss.31044.2|g.42225	-9.71	5.93	24.85	0.0005	0.0106
21 days	twi_ss.18899.1|g.24174	1.23	7.54	24.79	0.0005	0.0106
21 days	twi_ss.10437.2|g.14071	1.55	5.77	24.75	0.0005	0.0107
21 days	twi_ss.23113.2|g.29758	-1.53	6.76	24.74	0.0005	0.0107
21 days	twi_ss.9570.1|g.12838	-2.25	5.37	24.71	0.0005	0.0108
21 days	twi_ss.6679.1|g.9780	1.00	7.29	24.70	0.0005	0.0108
21 days	twi_ss.12175.1|g.16235	-2.37	5.06	24.67	0.0005	0.0108
21 days	twi_ss.12822.1|g.16932	-1.85	4.94	24.68	0.0005	0.0108
21 days	twi_ss.2199.1|g.3915	1.74	5.50	24.69	0.0005	0.0108
21 days	twi_ss.7242.1|g.10281	-1.95	5.41	24.68	0.0005	0.0108
21 days	twi_ss.22374.1|g.28899	-4.98	3.57	24.66	0.0005	0.0108
21 days	twi_ss.11466.1|g.15114	-1.90	3.88	24.60	0.0005	0.0109
21 days	twi_ss.17620.1|g.22508	-5.36	2.65	29.85	0.0005	0.0109
21 days	twi_ss.6818.1|g.9874	-0.97	6.84	24.61	0.0005	0.0109
21 days	twi_ss.25120.1|g.32969	1.21	6.53	24.57	0.0005	0.0109
21 days	twi_ss.891a.3|g.1588	1.42	6.45	24.58	0.0005	0.0109
21 days	twi_ss.7283.1|g.10307	-0.80	7.82	24.54	0.0005	0.0110
21 days	twi_ss.11609.1|g.15428	-1.50	5.92	24.51	0.0005	0.0110
21 days	twi_ss.19005.1|g.24288	-3.10	4.86	24.49	0.0005	0.0110
21 days	twi_ss.29442.1|g.39647	-1.92	5.56	24.49	0.0005	0.0110
21 days	twi_ss.6833.1|g.9896	-4.26	3.79	24.48	0.0005	0.0110
21 days	twi_ss.12876.1|g.17052	-1.28	6.47	24.48	0.0005	0.0110
21 days	twi_ss.25751.1|g.33988	-0.68	7.05	24.46	0.0005	0.0110
21 days	twi_ss.18626b.1|g.23809	-3.95	4.14	24.45	0.0005	0.0110
21 days	twi_ss.2254.1|g.3942	-0.71	8.64	24.45	0.0005	0.0110

21 days	twi_ss.24745.1|g.32452	1.30	4.94	24.44	0.0005	0.0110
21 days	twi_ss.28186a.1|g.37576	-5.05	5.36	24.44	0.0005	0.0111
21 days	twi_ss.13778.1|g.18098	1.75	7.32	24.42	0.0005	0.0111
21 days	twi_ss.27028.1|g.35919	0.76	7.57	24.41	0.0005	0.0111
21 days	twi_ss.495.1|g.897	1.20	4.84	24.40	0.0005	0.0111
21 days	twi_ss.20281.1|g.26123	1.15	6.05	24.39	0.0005	0.0111
21 days	twi_ss.24154.1|g.31475	0.89	6.63	24.36	0.0005	0.0111
21 days	twi_ss.26824.2|g.35518	1.30	5.69	24.36	0.0005	0.0111
21 days	twi_ss.22611.1|g.29205	-3.08	5.80	24.34	0.0005	0.0112
21 days	twi_ss.12999.5|g.17151	-1.11	7.16	24.33	0.0005	0.0112
21 days	twi_ss.13654.1|g.18011	-2.99	4.27	24.32	0.0005	0.0112
21 days	twi_ss.18881b.27|g.23963	-2.26	4.05	24.31	0.0005	0.0112
21 days	twi_ss.26361a.2|g.34929	-1.29	6.90	24.30	0.0005	0.0112
21 days	twi_ss.4059.1|g.6476	-4.63	4.73	24.29	0.0005	0.0112
21 days	twi_ss.18640.1|g.23785	-2.02	4.48	24.28	0.0005	0.0112
21 days	twi_ss.10273b.1|g.13796	0.80	9.05	24.27	0.0005	0.0112
21 days	twi_ss.28510a.24|g.38060	-6.93	3.78	29.38	0.0005	0.0112
21 days	twi_ss.11064.1|g.14683	0.62	9.52	24.25	0.0005	0.0112
21 days	twi_ss.18879.2|g.24150	-5.22	4.99	24.25	0.0005	0.0112
21 days	twi_ss.1818.1|g.3203	-6.89	3.34	40.76	0.0005	0.0113
21 days	twi_ss.15124.1|g.19641	1.78	7.05	24.20	0.0005	0.0113
21 days	twi_ss.25342.1|g.33330	-2.07	4.76	24.19	0.0005	0.0113
21 days	twi_ss.26608.1|g.35307	-3.33	4.22	24.18	0.0005	0.0113
21 days	twi_ss.6762.1|g.9844	-2.88	3.91	24.16	0.0005	0.0114
21 days	twi_ss.23266.1|g.29923	1.62	4.22	24.11	0.0005	0.0114
21 days	twi_ss.26399.1|g.34970	2.36	5.91	24.10	0.0005	0.0114
21 days	twi_ss.8754.1|g.11980	0.83	7.04	24.00	0.0006	0.0116
21 days	twi_ss.18618.1|g.23814	-2.84	5.20	23.98	0.0006	0.0116
21 days	twi_ss.16497.1|g.21089	-2.46	4.01	23.96	0.0006	0.0117
21 days	twi_ss.28926.1|g.38849	-1.75	5.16	23.93	0.0006	0.0117
21 days	twi_ss.26924.1|g.35731	0.98	6.17	23.92	0.0006	0.0117
21 days	twi_ss.30551.1|g.41380	4.31	5.98	23.87	0.0006	0.0118
21 days	twi_ss.4756.2|g.7341	1.81	5.15	23.87	0.0006	0.0118

21 days	twi_ss.12049.2|g.15954	1.63	5.01	23.84	0.0006	0.0118
21 days	twi_ss.13146.9|g.17473	4.13	5.22	23.84	0.0006	0.0118
21 days	twi_ss.16136.1|g.20730	1.47	5.17	23.85	0.0006	0.0118
21 days	twi_ss.24873.1|g.32725	1.27	6.17	23.85	0.0006	0.0118
21 days	twi_ss.31959.1|g.43577	-1.30	5.96	23.84	0.0006	0.0118
21 days	twi_ss.23867.1|g.31016	0.76	7.09	23.83	0.0006	0.0118
21 days	twi_ss.27217.1|g.36145	-2.50	4.57	23.83	0.0006	0.0118
21 days	twi_ss.28259.1|g.37792	0.67	7.59	23.81	0.0006	0.0119
21 days	twi_ss.2681.1|g.4899	1.03	7.96	23.80	0.0006	0.0119
21 days	twi_ss.2692.1|g.4845	-4.90	2.80	28.74	0.0006	0.0119
21 days	twi_ss.24638.1|g.32344	-6.65	3.75	28.73	0.0006	0.0119
21 days	twi_ss.3480b.2|g.5863	-0.88	7.73	23.71	0.0006	0.0120
21 days	twi_ss.26334.1|g.34890	-1.69	4.85	23.70	0.0006	0.0120
21 days	twi_ss.22218e.7|g.28734	6.89	4.53	28.60	0.0006	0.0120
21 days	twi_ss.2362.1|g.4274	-1.94	5.35	23.69	0.0006	0.0120
21 days	twi_ss.31790b.2|g.43275	-2.39	4.36	23.69	0.0006	0.0120
21 days	twi_ss.4103.1|g.6462	1.59	9.01	23.68	0.0006	0.0120
21 days	twi_ss.21197a.2|g.27421	2.46	4.81	23.65	0.0006	0.0121
21 days	twi_ss.19926.1|g.25684	0.76	9.57	23.64	0.0006	0.0121
21 days	twi_ss.30544.1|g.41374	-2.30	3.75	23.64	0.0006	0.0121
21 days	twi_ss.6297.1|g.9321	-6.32	3.08	28.49	0.0006	0.0121
21 days	twi_ss.16945.1|g.21593	1.50	4.68	23.60	0.0006	0.0121
21 days	twi_ss.21635a.2|g.28002	0.84	7.15	23.60	0.0006	0.0121
21 days	twi_ss.31616.1|g.43015	-1.34	5.20	23.59	0.0006	0.0121
21 days	twi_ss.1003.1|g.1833	0.95	6.19	23.58	0.0006	0.0121
21 days	twi_ss.3943.1|g.6365	-2.18	4.73	23.57	0.0006	0.0122
21 days	twi_ss.9313.5|g.12551	6.80	3.18	23.56	0.0006	0.0122
21 days	twi_ss.30919.1|g.41867	-2.30	4.63	23.53	0.0006	0.0122
21 days	twi_ss.5108.1|g.7749	-5.82	2.65	39.19	0.0006	0.0122
21 days	twi_ss.24923.1|g.32789	-2.45	3.83	23.52	0.0006	0.0122
21 days	twi_ss.31568.1|g.42983	-6.21	3.09	28.36	0.0006	0.0122
21 days	twi_ss.25732.1|g.33930	-3.16	4.28	23.50	0.0006	0.0122
21 days	twi_ss.27620.1|g.36669	0.76	8.29	23.50	0.0006	0.0122

21 days	twi_ss.23785a.2|g.30828	1.72	5.38	23.48	0.0006	0.0123
21 days	twi_ss.29406a.1|g.39525	2.16	5.48	23.48	0.0006	0.0123
21 days	twi_ss.4549.1|g.7066	-2.52	4.38	23.49	0.0006	0.0123
21 days	twi_ss.5041.1|g.7696	-1.90	5.56	23.45	0.0006	0.0123
21 days	twi_ss.2788.1|g.5068	0.95	6.98	23.41	0.0006	0.0124
21 days	twi_ss.5653.1|g.8500	1.04	5.87	23.41	0.0006	0.0124
21 days	twi_ss.20562.1|g.26577	-4.05	4.07	23.39	0.0006	0.0124
21 days	twi_ss.27819.3|g.37033	-2.56	3.68	23.39	0.0006	0.0124
21 days	twi_ss.3614.1|g.5977	-0.72	6.64	23.38	0.0006	0.0124
21 days	twi_ss.1584.2|g.2844	1.00	6.16	23.38	0.0006	0.0124
21 days	twi_ss.21746.3|g.28220	-6.99	3.37	28.17	0.0006	0.0124
21 days	twi_ss.4707.1|g.7260	-0.60	6.83	23.34	0.0006	0.0124
21 days	twi_ss.6247.1|g.9241	0.97	6.92	23.34	0.0006	0.0124
21 days	twi_ss.8230.1|g.11305	0.94	7.01	23.34	0.0006	0.0124
21 days	twi_ss.30657a.2|g.41503	-7.49	4.22	23.32	0.0006	0.0125
21 days	twi_ss.25333.1|g.33360	0.70	8.35	23.29	0.0006	0.0125
21 days	twi_ss.24491.1|g.32093	-1.32	6.00	23.28	0.0006	0.0125
21 days	twi_ss.31618.1|g.43019	0.96	7.34	23.24	0.0006	0.0126
21 days	twi_ss.1893.1|g.3341	0.91	6.83	23.22	0.0006	0.0126
21 days	twi_ss.20963.1|g.27081	1.24	4.56	23.21	0.0006	0.0126
21 days	twi_ss.25883.1|g.34164	0.72	8.82	23.22	0.0006	0.0126
21 days	twi_ss.30540.1|g.41328	0.49	9.47	23.22	0.0006	0.0126
21 days	twi_ss.31816.1|g.43340	-3.57	4.56	23.23	0.0006	0.0126
21 days	twi_ss.3498.1|g.5871	0.79	7.09	23.22	0.0006	0.0126
21 days	twi_ss.24627.1|g.32261	-7.30	4.14	23.19	0.0006	0.0127
21 days	twi_ss.28220a.3|g.37612	-2.02	4.44	23.17	0.0006	0.0127
21 days	twi_ss.10058.1|g.13415	-5.40	3.75	23.15	0.0006	0.0127
21 days	twi_ss.1616.1|g.2829	-1.59	7.25	23.15	0.0006	0.0127
21 days	twi_ss.17480.1|g.22291	1.25	5.97	23.15	0.0006	0.0127
21 days	twi_ss.29885.1|g.40327	-4.30	4.93	23.14	0.0006	0.0127
21 days	twi_ss.447.1|g.847	-1.86	5.50	23.16	0.0006	0.0127
21 days	twi_ss.8443.1|g.11629	-1.37	6.09	23.14	0.0006	0.0127
21 days	twi_ss.17793a.3|g.22692	-0.85	6.38	23.14	0.0006	0.0127

21 days	twi_ss.5493.1|g.8278	-1.85	4.65	23.13	0.0006	0.0127
21 days	twi_ss.14843.1|g.19305	-5.06	3.07	27.84	0.0006	0.0127
21 days	twi_ss.28457.1|g.38034	-3.42	5.19	23.09	0.0006	0.0128
21 days	twi_ss.29473.1|g.39694	-4.22	3.10	23.07	0.0006	0.0128
21 days	twi_ss.984.1|g.1782	4.07	4.43	23.07	0.0006	0.0128
21 days	twi_ss.1905.1|g.3389	1.14	5.71	23.05	0.0006	0.0128
21 days	twi_ss.26050.1|g.34513	-1.15	4.82	23.01	0.0007	0.0129
21 days	twi_ss.17767.4|g.22645	-3.34	4.18	22.98	0.0007	0.0129
21 days	twi_ss.23881.2|g.31020	1.31	4.95	22.98	0.0007	0.0129
21 days	twi_ss.30862.1|g.42102	-1.21	5.47	22.98	0.0007	0.0129
21 days	twi_ss.4148.1|g.6569	-1.97	4.51	22.98	0.0007	0.0129
21 days	twi_ss.476.1|g.879	1.43	4.84	22.97	0.0007	0.0129
21 days	twi_ss.630.1|g.1146	-1.99	5.06	22.95	0.0007	0.0130
21 days	twi_ss.11553.1|g.15368	1.11	6.71	22.92	0.0007	0.0130
21 days	twi_ss.21453b.5|g.27717	6.11	2.72	27.55	0.0007	0.0130
21 days	twi_ss.6204.1|g.9043	1.18	8.39	22.91	0.0007	0.0130
21 days	twi_ss.26816.1|g.35581	-5.07	2.67	27.52	0.0007	0.0131
21 days	twi_ss.16407.1|g.20962	1.22	9.11	22.85	0.0007	0.0131
21 days	twi_ss.1809.2|g.3201	1.02	7.68	22.83	0.0007	0.0132
21 days	twi_ss.1234.3|g.2201	-7.12	3.58	22.82	0.0007	0.0132
21 days	twi_ss.31536.1|g.42950	-3.59	4.21	22.82	0.0007	0.0132
21 days	twi_ss.23011.1|g.29652	-2.93	5.36	22.80	0.0007	0.0132
21 days	twi_ss.11526.15|g.15221	3.08	3.74	22.79	0.0007	0.0132
21 days	twi_ss.12535.1|g.16682	-3.79	4.06	22.79	0.0007	0.0132
21 days	twi_ss.2790.1|g.5020	0.78	6.34	22.77	0.0007	0.0132
21 days	twi_ss.5694.1|g.8531	-3.09	3.98	22.77	0.0007	0.0132
21 days	twi_ss.2754.1|g.4996	1.27	5.70	22.76	0.0007	0.0132
21 days	twi_ss.12878.1|g.17063	-1.77	6.15	22.72	0.0007	0.0133
21 days	twi_ss.18979.1|g.24245	6.78	3.17	22.71	0.0007	0.0133
21 days	twi_ss.1959a.1|g.3428	1.95	5.06	22.72	0.0007	0.0133
21 days	twi_ss.22469.1|g.29045	-1.47	5.66	22.63	0.0007	0.0135
21 days	twi_ss.27004.2|g.35871	0.55	6.84	22.61	0.0007	0.0135
21 days	twi_ss.21375b.1|g.27632	1.26	6.28	22.59	0.0007	0.0136

21 days	twi_ss.14291a.1|g.18602	-1.90	4.24	22.56	0.0007	0.0136
21 days	twi_ss.11378.4|g.14981	-7.55	3.57	27.07	0.0007	0.0136
21 days	twi_ss.15760.1|g.20329	-4.40	4.25	22.56	0.0007	0.0136
21 days	twi_ss.5474.2|g.8254	3.62	5.45	22.53	0.0007	0.0137
21 days	twi_ss.28714.2|g.38497	-1.65	5.04	22.52	0.0007	0.0137
21 days	twi_ss.9215.3|g.12424	-1.69	4.82	22.51	0.0007	0.0137
21 days	twi_ss.9215.4|g.12420	-1.69	4.82	22.51	0.0007	0.0137
21 days	twi_ss.5007.2|g.7625	-6.10	2.81	22.48	0.0007	0.0137
21 days	twi_ss.18318.2|g.23343	1.42	4.97	22.47	0.0007	0.0138
21 days	twi_ss.19831.9|g.25475	5.41	2.22	36.84	0.0007	0.0138
21 days	twi_ss.8303a.1|g.11337	0.60	11.05	22.46	0.0007	0.0138
21 days	twi_ss.12009.1|g.15919	0.95	7.46	22.44	0.0007	0.0138
21 days	twi_ss.6079.1|g.8932	1.01	7.81	22.44	0.0007	0.0138
21 days	twi_ss.21602.1|g.27974	5.16	3.14	26.90	0.0007	0.0138
21 days	twi_ss.28493.1|g.38148	-2.53	4.21	22.42	0.0007	0.0138
21 days	twi_ss.15552a.1|g.20112	-3.85	4.02	22.42	0.0007	0.0138
21 days	twi_ss.24487.1|g.32104	-3.64	3.69	22.39	0.0007	0.0139
21 days	twi_ss.9920.2|g.13257	4.46	2.76	22.38	0.0007	0.0139
21 days	twi_ss.6662.1|g.9728	0.78	6.14	22.34	0.0007	0.0140
21 days	twi_ss.18630.1|g.23736	0.83	9.37	22.30	0.0007	0.0140
21 days	twi_ss.2655.1|g.4837	1.41	5.39	22.30	0.0007	0.0140
21 days	twi_ss.18394.1|g.23443	-6.82	3.65	26.71	0.0007	0.0141
21 days	twi_ss.814.1|g.1385	-2.83	4.97	22.27	0.0007	0.0141
21 days	twi_ss.11707.2|g.15505	-7.32	3.62	22.26	0.0007	0.0141
21 days	twi_ss.13154.1|g.17467	0.97	5.31	22.26	0.0007	0.0141
21 days	twi_ss.25558a.4|g.33612	-1.80	5.00	22.26	0.0007	0.0141
21 days	twi_ss.3226.1|g.5656	2.72	3.24	22.25	0.0007	0.0141
21 days	twi_ss.7777.1|g.10852	3.24	4.97	22.22	0.0007	0.0142
21 days	twi_ss.5387.1|g.8143	-0.76	7.38	22.21	0.0007	0.0142
21 days	twi_ss.22274.1|g.28804	1.11	6.72	22.20	0.0007	0.0142
21 days	twi_ss.4343.1|g.6846	-1.53	5.09	22.18	0.0007	0.0142
21 days	twi_ss.29508.1|g.39754	0.91	6.20	22.16	0.0007	0.0143
21 days	twi_ss.12319.1|g.16441	-3.88	5.15	22.16	0.0007	0.0143

21 days	twi_ss.5898.1|g.8780	-2.42	4.71	22.15	0.0007	0.0143
21 days	twi_ss.27318.1|g.36310	6.58	2.82	22.14	0.0007	0.0143
21 days	twi_ss.7075.1|g.10131	-3.18	4.19	22.14	0.0008	0.0143
21 days	twi_ss.18983.1|g.24272	1.65	4.63	22.10	0.0008	0.0144
21 days	twi_ss.35b.1|g.142	1.26	7.54	22.09	0.0008	0.0144
21 days	twi_ss.440.1|g.827	1.02	6.27	22.07	0.0008	0.0144
21 days	twi_ss.23517.1|g.30164	0.99	5.92	22.07	0.0008	0.0144
21 days	twi_ss.10747a.2|g.14392	-1.90	5.71	22.04	0.0008	0.0145
21 days	twi_ss.9231.1|g.12468	-1.74	5.37	22.04	0.0008	0.0145
21 days	twi_ss.31830.1|g.43358	0.72	7.46	22.01	0.0008	0.0145
21 days	twi_ss.1114.1|g.1985	1.53	4.44	21.99	0.0008	0.0146
21 days	twi_ss.22388.1|g.28909	-6.69	3.13	35.81	0.0008	0.0146
21 days	twi_ss.4635.1|g.7183	-2.47	4.96	21.98	0.0008	0.0146
21 days	twi_ss.11691.1|g.15523	1.03	5.69	21.97	0.0008	0.0146
21 days	twi_ss.14208.1|g.18481	1.34	5.71	21.94	0.0008	0.0147
21 days	twi_ss.18602.1|g.23723	-3.45	4.91	21.94	0.0008	0.0147
21 days	twi_ss.2961.1|g.5302	-2.73	4.00	21.93	0.0008	0.0147
21 days	twi_ss.11315.1|g.14927	1.56	4.77	21.92	0.0008	0.0147
21 days	twi_ss.12695.1|g.16773	-3.39	4.19	21.91	0.0008	0.0147
21 days	twi_ss.22989.1|g.29643	0.99	5.58	21.91	0.0008	0.0147
21 days	twi_ss.3981.1|g.6392	-1.86	4.33	21.92	0.0008	0.0147
21 days	twi_ss.25499.1|g.33535	-2.36	3.93	21.89	0.0008	0.0147
21 days	twi_ss.25499.2|g.33537	-2.36	3.93	21.89	0.0008	0.0147
21 days	twi_ss.25499.4|g.33534	-2.36	3.93	21.89	0.0008	0.0147
21 days	twi_ss.30112a.1|g.40710	0.86	7.02	21.89	0.0008	0.0147
21 days	twi_ss.8413.1|g.11540	-1.14	7.70	21.89	0.0008	0.0147
21 days	twi_ss.20053.1|g.25830	1.13	6.00	21.89	0.0008	0.0147
21 days	twi_ss.27005.1|g.35887	-1.31	5.08	21.87	0.0008	0.0147
21 days	twi_ss.27925b.2|g.37193	-2.28	4.45	21.86	0.0008	0.0147
21 days	twi_ss.26710.2|g.35422	-7.20	3.57	21.86	0.0008	0.0147
21 days	twi_ss.15925d.9|g.20473	-1.44	5.84	21.85	0.0008	0.0147
21 days	twi_ss.15661.1|g.20226	0.66	7.51	21.85	0.0008	0.0147
21 days	twi_ss.29914.1|g.40391	-2.64	3.59	21.83	0.0008	0.0148

21 days	twi_ss.24081.1|g.31319	0.58	7.87	21.81	0.0008	0.0148
21 days	twi_ss.25552.1|g.33606	-1.81	5.53	21.79	0.0008	0.0148
21 days	twi_ss.30429.1|g.41205	-2.12	6.15	21.80	0.0008	0.0148
21 days	twi_ss.3505.1|g.5882	-4.91	4.55	21.76	0.0008	0.0149
21 days	twi_ss.30867.2|g.41899	-0.98	5.78	21.73	0.0008	0.0150
21 days	twi_ss.30121.1|g.40695	-4.43	3.58	21.73	0.0008	0.0150
21 days	twi_ss.1272.1|g.2274	1.07	7.59	21.72	0.0008	0.0150
21 days	twi_ss.5087.1|g.7753	-5.63	4.31	25.94	0.0008	0.0150
21 days	twi_ss.7889.1|g.10952	-4.76	3.35	21.71	0.0008	0.0150
21 days	twi_ss.19359.1|g.24865	0.72	6.55	21.68	0.0008	0.0150
21 days	twi_ss.2485.1|g.4550	1.99	4.35	21.69	0.0008	0.0150
21 days	twi_ss.25424.2|g.33443	0.83	7.11	21.68	0.0008	0.0150
21 days	twi_ss.3076.1|g.5470	2.53	4.69	21.68	0.0008	0.0150
21 days	twi_ss.5863.1|g.8715	-0.82	9.63	21.68	0.0008	0.0150
21 days	twi_ss.28007.1|g.37283	2.04	8.60	21.67	0.0008	0.0150
21 days	twi_ss.21532.6|g.27875	1.54	5.01	21.66	0.0008	0.0150
21 days	twi_ss.20991.1|g.27100	-3.18	5.78	21.64	0.0008	0.0151
21 days	twi_ss.25146.1|g.33000	-2.08	7.65	21.60	0.0008	0.0152
21 days	twi_ss.574.2|g.1054	1.63	7.11	21.60	0.0008	0.0152
21 days	twi_ss.21610.5|g.28012	2.65	6.22	21.58	0.0008	0.0152
21 days	twi_ss.23502.1|g.30094	-6.06	2.80	25.78	0.0008	0.0152
21 days	twi_ss.28506.9|g.38184	-6.14	2.65	34.95	0.0008	0.0152
21 days	twi_ss.7151.1|g.10200	-6.33	2.94	34.96	0.0008	0.0152
21 days	twi_ss.20081.1|g.25853	0.81	6.31	21.54	0.0008	0.0153
21 days	twi_ss.24028.2|g.31262	2.27	5.27	21.51	0.0008	0.0153
21 days	twi_ss.6695.1|g.9740	0.79	7.16	21.49	0.0008	0.0154
21 days	twi_ss.6172.1|g.9048	0.92	7.49	21.48	0.0008	0.0154
21 days	twi_ss.29118.1|g.39040	-1.26	5.97	21.47	0.0008	0.0154
21 days	twi_ss.7687.1|g.10769	-4.80	3.38	21.46	0.0008	0.0154
21 days	twi_ss.26489.1|g.35100	-1.64	6.01	21.45	0.0008	0.0154
21 days	twi_ss.4977.4|g.7553	-2.48	4.91	21.45	0.0008	0.0155
21 days	twi_ss.21412b.2|g.27660	1.58	4.73	21.44	0.0008	0.0155
21 days	twi_ss.27089.2|g.35960	-3.85	4.44	21.44	0.0008	0.0155

21 days	twi_ss.6728.1|g.9797	-1.93	4.37	21.43	0.0008	0.0155
21 days	twi_ss.22501.1|g.29071	-0.70	7.53	21.41	0.0008	0.0155
21 days	twi_ss.29585.1|g.39874	1.00	6.80	21.41	0.0008	0.0155
21 days	twi_ss.21610.4|g.28010	2.18	6.64	21.38	0.0009	0.0156
21 days	twi_ss.22261.1|g.28816	-6.30	3.08	25.50	0.0009	0.0156
21 days	twi_ss.28718.1|g.38438	6.33	2.67	21.36	0.0009	0.0156
21 days	twi_ss.28718.3|g.38437	6.33	2.67	21.36	0.0009	0.0156
21 days	twi_ss.30543.1|g.41378	-1.79	4.85	21.36	0.0009	0.0156
21 days	twi_ss.3249.1|g.5670	-1.65	4.98	21.36	0.0009	0.0156
21 days	twi_ss.19279.1|g.24687	-1.72	5.14	21.30	0.0009	0.0157
21 days	twi_ss.14339b.2|g.18702	-6.96	3.29	34.34	0.0009	0.0157
21 days	twi_ss.558.1|g.1024	1.73	4.96	21.28	0.0009	0.0158
21 days	twi_ss.26281.1|g.34909	-3.44	3.65	21.26	0.0009	0.0158
21 days	twi_ss.19776.2|g.25452	0.75	7.26	21.24	0.0009	0.0158
21 days	twi_ss.12155.1|g.16039	0.81	6.61	21.23	0.0009	0.0159
21 days	twi_ss.16726.1|g.21410	-3.42	3.80	21.22	0.0009	0.0159
21 days	twi_ss.22247.1|g.28780	0.56	10.05	21.23	0.0009	0.0159
21 days	twi_ss.24622.1|g.32277	-2.83	3.97	21.22	0.0009	0.0159
21 days	twi_ss.24741.1|g.32467	0.82	7.25	21.22	0.0009	0.0159
21 days	twi_ss.10584.1|g.14227	-2.92	4.66	21.17	0.0009	0.0159
21 days	twi_ss.18476.1|g.23508	-5.55	3.68	21.17	0.0009	0.0159
21 days	twi_ss.24052.1|g.31321	3.56	3.46	21.17	0.0009	0.0159
21 days	twi_ss.24052.2|g.31320	3.56	3.46	21.17	0.0009	0.0159
21 days	twi_ss.24234.1|g.31692	0.66	7.93	21.18	0.0009	0.0159
21 days	twi_ss.26938.1|g.35766	-4.50	3.65	21.16	0.0009	0.0159
21 days	twi_ss.6610.1|g.9638	2.21	5.00	21.17	0.0009	0.0159
21 days	twi_ss.28037.1|g.37356	2.41	4.00	21.15	0.0009	0.0160
21 days	twi_ss.18428.1|g.23474	1.50	5.33	21.13	0.0009	0.0160
21 days	twi_ss.4306.2|g.6752	-3.00	6.42	21.14	0.0009	0.0160
21 days	twi_ss.12118.1|g.16075	-1.55	8.44	21.12	0.0009	0.0160
21 days	twi_ss.6700.1|g.9783	-2.02	5.53	21.11	0.0009	0.0160
21 days	twi_ss.25312.4|g.33266	3.42	6.12	21.10	0.0009	0.0161
21 days	twi_ss.2174.1|g.3848	0.70	7.66	21.09	0.0009	0.0161

21 days	twi_ss.27928.4|g.37169	6.76	2.92	21.07	0.0009	0.0161
21 days	twi_ss.26015c.3|g.34432	-1.87	4.92	21.06	0.0009	0.0162
21 days	twi_ss.4284.1|g.6781	-1.32	5.39	21.04	0.0009	0.0162
21 days	twi_ss.4977.2|g.7539	-7.18	3.31	25.05	0.0009	0.0162
21 days	twi_ss.2734.1|g.4902	1.31	5.41	21.03	0.0009	0.0162
21 days	twi_ss.23645.1|g.30478	1.14	5.18	21.02	0.0009	0.0162
21 days	twi_ss.22293.1|g.28839	-4.86	3.50	21.01	0.0009	0.0162
21 days	twi_ss.8427.1|g.11612	-2.36	4.67	21.01	0.0009	0.0162
21 days	twi_ss.26855.1|g.35618	-2.54	4.45	20.97	0.0009	0.0163
21 days	twi_ss.2310.1|g.4082	-1.91	4.97	20.93	0.0009	0.0164
21 days	twi_ss.20209.1|g.26003	1.90	5.42	20.93	0.0009	0.0164
21 days	twi_ss.29294.1|g.39307	0.82	6.93	20.92	0.0009	0.0165
21 days	twi_ss.4483.1|g.7000	0.79	6.01	20.90	0.0009	0.0165
21 days	twi_ss.27031.1|g.35920	1.16	7.00	20.88	0.0009	0.0165
21 days	twi_ss.19378.2|g.24898	3.82	2.72	20.87	0.0009	0.0166
21 days	twi_ss.5372.1|g.8137	-1.54	5.65	20.84	0.0009	0.0166
21 days	twi_ss.19562.1|g.25130	0.80	7.69	20.84	0.0009	0.0166
21 days	twi_ss.3789a.2|g.6222	-2.16	5.96	20.84	0.0009	0.0166
21 days	twi_ss.28187.4|g.37572	-5.89	5.11	20.81	0.0009	0.0167
21 days	twi_ss.24090.1|g.31393	2.03	4.59	20.81	0.0009	0.0167
21 days	twi_ss.6207.1|g.9205	0.78	7.63	20.80	0.0009	0.0167
21 days	twi_ss.18038.2|g.22931	-3.03	4.81	20.77	0.0009	0.0168
21 days	twi_ss.15395.1|g.19951	1.00	7.90	20.76	0.0009	0.0168
21 days	twi_ss.13681.2|g.18027	-5.56	2.47	20.74	0.0010	0.0168
21 days	twi_ss.20550.1|g.26467	1.58	6.79	20.74	0.0010	0.0168
21 days	twi_ss.2198.1|g.3886	1.01	8.50	20.73	0.0010	0.0168
21 days	twi_ss.29806.1|g.40219	-5.44	3.70	20.73	0.0010	0.0168
21 days	twi_ss.27392.1|g.36463	-1.72	4.60	20.71	0.0010	0.0169
21 days	twi_ss.31306.1|g.42632	1.02	7.27	20.71	0.0010	0.0169
21 days	twi_ss.3837.1|g.6289	-1.27	6.03	20.72	0.0010	0.0169
21 days	twi_ss.9215.2|g.12418	-2.77	5.43	20.70	0.0010	0.0169
21 days	twi_ss.14526.1|g.18906	-2.25	5.15	20.68	0.0010	0.0169
21 days	twi_ss.29195.1|g.39113	1.18	4.88	20.68	0.0010	0.0169

21 days	twi_ss.26953.1|g.35739	0.57	8.16	20.63	0.0010	0.0171
21 days	twi_ss.16791.1|g.21460	-3.22	3.44	20.61	0.0010	0.0171
21 days	twi_ss.28559.1|g.38253	-3.81	4.09	20.60	0.0010	0.0171
21 days	twi_ss.844.1|g.1489	-2.91	6.22	20.60	0.0010	0.0171
21 days	twi_ss.2601.1|g.4625	0.85	6.38	20.58	0.0010	0.0172
21 days	twi_ss.26824.1|g.35514	1.13	6.60	20.57	0.0010	0.0172
21 days	twi_ss.6181b.2|g.9066	-0.99	5.76	20.57	0.0010	0.0172
21 days	twi_ss.27316.1|g.36336	-1.78	4.84	20.56	0.0010	0.0172
21 days	twi_ss.7326.1|g.10332	1.13	7.18	20.54	0.0010	0.0173
21 days	twi_ss.10283.1|g.13790	2.30	6.08	20.53	0.0010	0.0173
21 days	twi_ss.27195.1|g.36148	-3.45	3.67	20.51	0.0010	0.0173
21 days	twi_ss.16934.1|g.21585	-1.88	5.48	20.51	0.0010	0.0173
21 days	twi_ss.17959.1|g.22843	-1.90	5.07	20.50	0.0010	0.0173
21 days	twi_ss.20867.1|g.26998	-2.94	4.96	20.47	0.0010	0.0174
21 days	twi_ss.10746.3|g.14382	1.22	6.87	20.47	0.0010	0.0174
21 days	twi_ss.29971.1|g.40449	1.89	6.18	20.46	0.0010	0.0174
21 days	twi_ss.8324a.2|g.11405	0.54	6.97	20.43	0.0010	0.0175
21 days	twi_ss.2632.2|g.4692	6.47	3.04	20.42	0.0010	0.0175
21 days	twi_ss.21458.1|g.27724	-2.12	5.53	20.42	0.0010	0.0175
21 days	twi_ss.22673.2|g.29281	-2.48	5.04	20.41	0.0010	0.0176
21 days	twi_ss.9180.1|g.12368	-5.05	4.49	20.40	0.0010	0.0176
21 days	twi_ss.24905.1|g.32762	-3.77	3.63	20.40	0.0010	0.0176
21 days	twi_ss.24382.1|g.31995	-1.70	5.24	20.38	0.0010	0.0176
21 days	twi_ss.30700b.6|g.41568	6.70	2.92	20.37	0.0010	0.0176
21 days	twi_ss.14987.1|g.19496	3.35	3.91	20.37	0.0010	0.0176
21 days	twi_ss.8474.1|g.11541	-1.65	4.83	20.37	0.0010	0.0176
21 days	twi_ss.437.1|g.823	0.75	9.22	20.35	0.0010	0.0177
21 days	twi_ss.24515.2|g.32164	0.81	5.90	20.34	0.0010	0.0177
21 days	twi_ss.23684.2|g.30599	-6.40	3.73	20.33	0.0010	0.0177
21 days	twi_ss.18398.1|g.23447	-2.04	3.80	20.33	0.0010	0.0177
21 days	twi_ss.4244.1|g.6679	-4.49	2.44	26.89	0.0010	0.0177
21 days	twi_ss.19458.1|g.25023	1.01	6.19	20.32	0.0010	0.0177
21 days	twi_ss.1961.2|g.3438	-0.94	5.64	20.30	0.0010	0.0177

21 days	twi_ss.30594.1|g.41437	-2.31	6.48	20.30	0.0010	0.0177
21 days	twi_ss.30983b.3|g.41861	-4.96	4.02	20.30	0.0010	0.0178
21 days	twi_ss.22038.1|g.28557	0.82	7.05	20.29	0.0010	0.0178
21 days	twi_ss.19836.2|g.25503	5.45	2.25	20.26	0.0010	0.0178
21 days	twi_ss.2549.1|g.4559	0.73	8.20	20.25	0.0010	0.0179
21 days	twi_ss.26694a.1|g.35459	1.50	7.42	20.25	0.0010	0.0179
21 days	twi_ss.28819.1|g.38592	-2.55	6.57	20.25	0.0010	0.0179
21 days	twi_ss.17053.1|g.21823	0.52	8.77	20.23	0.0010	0.0179
21 days	twi_ss.836.3|g.1475	-2.98	6.16	20.21	0.0010	0.0180
21 days	twi_ss.20345.2|g.26180	-3.15	4.93	20.19	0.0010	0.0180
21 days	twi_ss.21280.1|g.27544	-3.18	4.47	20.19	0.0010	0.0180
21 days	twi_ss.3231.1|g.5660	-1.12	4.79	24.56	0.0010	0.0180
21 days	twi_ss.5530.1|g.8370	-1.75	5.16	20.19	0.0010	0.0180
21 days	twi_ss.17829.1|g.22743	1.85	5.13	20.18	0.0010	0.0180
21 days	twi_ss.24526c.3|g.32158	-3.03	5.14	20.17	0.0010	0.0180
21 days	twi_ss.29855.1|g.40270	-2.28	4.66	20.17	0.0010	0.0180
21 days	twi_ss.13078.1|g.17398	0.78	6.80	20.16	0.0011	0.0180
21 days	twi_ss.21329.1|g.27617	6.79	4.10	20.16	0.0011	0.0180
21 days	twi_ss.24824.1|g.32616	0.49	8.29	20.16	0.0011	0.0180
21 days	twi_ss.25939.4|g.34236	-6.19	2.73	23.87	0.0011	0.0180
21 days	twi_ss.29949.1|g.40411	1.32	5.40	20.14	0.0011	0.0180
21 days	twi_ss.4477.2|g.6993	4.50	3.83	23.85	0.0011	0.0181
21 days	twi_ss.21299.1|g.27525	-2.18	3.92	20.12	0.0011	0.0181
21 days	twi_ss.8141.1|g.11183	-3.12	3.41	20.12	0.0011	0.0181
21 days	twi_ss.20609.1|g.26641	1.75	5.60	20.09	0.0011	0.0182
21 days	twi_ss.23834.1|g.30873	1.26	7.71	20.08	0.0011	0.0182
21 days	twi_ss.19532.1|g.25054	-1.48	4.90	20.07	0.0011	0.0182
21 days	twi_ss.26082.1|g.34548	4.96	2.11	20.07	0.0011	0.0182
21 days	twi_ss.31956.1|g.43530	0.51	7.53	20.05	0.0011	0.0182
21 days	twi_ss.8490.1|g.11663	1.10	5.06	20.05	0.0011	0.0182
21 days	twi_ss.25341a.2|g.33365	-2.08	4.60	20.03	0.0011	0.0183
21 days	twi_ss.29230.1|g.39192	-2.26	3.63	20.02	0.0011	0.0183
21 days	twi_ss.10630.1|g.14270	-3.19	3.38	20.00	0.0011	0.0184

21 days	twi_ss.20175.1|g.25982	0.79	6.26	20.01	0.0011	0.0184
21 days	twi_ss.22393a.1|g.28911	-1.12	6.33	20.00	0.0011	0.0184
21 days	twi_ss.30118.1|g.40686	-1.90	5.85	20.00	0.0011	0.0184
21 days	twi_ss.10852.1|g.14478	-2.00	3.90	19.99	0.0011	0.0184
21 days	twi_ss.3404.1|g.5787	-6.07	2.77	31.58	0.0011	0.0184
21 days	twi_ss.17576.3|g.22477	-3.08	3.91	19.97	0.0011	0.0184
21 days	twi_ss.25504.1|g.33563	-2.83	5.57	19.96	0.0011	0.0184
21 days	twi_ss.22634.1|g.29237	-1.11	5.85	19.93	0.0011	0.0185
21 days	twi_ss.22899.1|g.29570	-2.06	5.52	19.93	0.0011	0.0185
21 days	twi_ss.23041.1|g.29689	0.67	6.56	19.91	0.0011	0.0186
21 days	twi_ss.31973.1|g.43597	-1.44	5.16	19.90	0.0011	0.0186
21 days	twi_ss.1441.1|g.2632	1.00	5.51	19.89	0.0011	0.0186
21 days	twi_ss.19129.1|g.24538	-5.64	2.63	23.50	0.0011	0.0187
21 days	twi_ss.20681.1|g.26792	1.16	6.36	19.86	0.0011	0.0187
21 days	twi_ss.17964.1|g.22828	2.39	8.67	19.85	0.0011	0.0187
21 days	twi_ss.22336.1|g.28863	1.97	4.10	19.85	0.0011	0.0187
21 days	twi_ss.4264.1|g.6704	3.01	4.78	19.84	0.0011	0.0187
21 days	twi_ss.6317.1|g.9345	-1.46	7.42	19.84	0.0011	0.0187
21 days	twi_ss.7420.6|g.10428	1.18	4.58	19.83	0.0011	0.0187
21 days	twi_ss.946.10|g.1716	5.86	2.46	19.83	0.0011	0.0187
21 days	twi_ss.946.3|g.1704	5.86	2.46	19.83	0.0011	0.0187
21 days	twi_ss.30079.1|g.40602	-1.90	4.16	19.81	0.0011	0.0188
21 days	twi_ss.30743.1|g.41663	-2.34	4.03	19.80	0.0011	0.0188
21 days	twi_ss.24658.1|g.32366	-1.11	5.87	19.79	0.0011	0.0188
21 days	twi_ss.22776.1|g.29409	0.92	6.33	19.78	0.0011	0.0188
21 days	twi_ss.5340.1|g.8047	0.73	6.93	19.78	0.0011	0.0188
21 days	twi_ss.9198.3|g.12456	1.15	4.88	19.75	0.0011	0.0189
21 days	twi_ss.11751.1|g.15579	-2.75	4.15	19.74	0.0011	0.0189
21 days	twi_ss.13475.1|g.17845	-2.44	3.36	19.75	0.0011	0.0189
21 days	twi_ss.17494.1|g.22269	1.13	6.54	19.71	0.0011	0.0190
21 days	twi_ss.10631.1|g.14271	1.34	4.98	19.68	0.0011	0.0191
21 days	twi_ss.14077.4|g.18364	-5.34	2.63	19.68	0.0011	0.0191
21 days	twi_ss.10136.1|g.13580	7.19	3.19	56.11	0.0012	0.0192

21 days	twi_ss.27594.1|g.36809	-1.70	4.41	19.65	0.0012	0.0192
21 days	twi_ss.23136.1|g.29794	-1.65	5.74	19.65	0.0012	0.0192
21 days	twi_ss.22925.1|g.29592	-3.50	3.64	19.64	0.0012	0.0192
21 days	twi_ss.25757.7|g.33980	-7.55	3.87	19.63	0.0012	0.0192
21 days	twi_ss.4577.1|g.7107	-3.33	3.66	19.62	0.0012	0.0193
21 days	twi_ss.27510a.2|g.36667	-2.36	6.04	19.61	0.0012	0.0193
21 days	twi_ss.1020.1|g.1845	0.88	6.23	19.61	0.0012	0.0193
21 days	twi_ss.27290.1|g.36273	-1.96	4.87	19.60	0.0012	0.0193
21 days	twi_ss.29846.1|g.40284	1.37	6.44	19.60	0.0012	0.0193
21 days	twi_ss.19285.1|g.24699	-2.45	4.80	19.59	0.0012	0.0193
21 days	twi_ss.1728.1|g.3067	-2.30	4.41	19.57	0.0012	0.0194
21 days	twi_ss.26694a.2|g.35456	0.81	7.37	19.57	0.0012	0.0194
21 days	twi_ss.188.1|g.303	-0.99	8.65	19.57	0.0012	0.0194
21 days	twi_ss.11619.2|g.15422	5.11	2.37	19.55	0.0012	0.0194
21 days	twi_ss.1168.1|g.2027	1.33	6.63	19.55	0.0012	0.0194
21 days	twi_ss.17008.1|g.21730	1.59	5.75	19.56	0.0012	0.0194
21 days	twi_ss.20577.1|g.26632	1.04	6.44	19.55	0.0012	0.0194
21 days	twi_ss.23041.3|g.29688	1.06	5.67	19.55	0.0012	0.0194
21 days	twi_ss.29277.1|g.39272	-1.32	6.48	19.55	0.0012	0.0194
21 days	twi_ss.21978.1|g.28482	-3.36	3.45	19.53	0.0012	0.0194
21 days	twi_ss.31407.1|g.42758	-1.57	5.35	19.53	0.0012	0.0194
21 days	twi_ss.13437.1|g.17802	1.45	6.04	19.50	0.0012	0.0195
21 days	twi_ss.28459.1|g.38109	6.09	2.95	19.48	0.0012	0.0196
21 days	twi_ss.30746.2|g.41665	-1.39	5.36	19.48	0.0012	0.0196
21 days	twi_ss.30424.1|g.41215	-1.83	6.48	19.46	0.0012	0.0196
21 days	twi_ss.23529a.1|g.30186	-2.44	3.73	19.45	0.0012	0.0196
21 days	twi_ss.28912.1|g.38856	4.27	2.81	25.43	0.0012	0.0196
21 days	twi_ss.13077.1|g.17423	-2.66	3.25	19.43	0.0012	0.0197
21 days	twi_ss.10516.1|g.14150	3.48	5.54	19.42	0.0012	0.0197
21 days	twi_ss.24844.1|g.32682	0.76	8.39	19.42	0.0012	0.0197
21 days	twi_ss.1264.1|g.2230	-2.27	6.14	19.41	0.0012	0.0197
21 days	twi_ss.16351.1|g.20945	1.24	5.12	19.41	0.0012	0.0197
21 days	twi_ss.2230.1|g.3995	1.28	5.06	19.40	0.0012	0.0197

21 days	twi_ss.12144.1|g.16027	0.57	7.87	19.40	0.0012	0.0197
21 days	twi_ss.1880.1|g.3316	-3.93	3.93	19.40	0.0012	0.0197
21 days	twi_ss.366.1|g.666	3.27	3.92	19.39	0.0012	0.0197
21 days	twi_ss.19501.1|g.25037	1.16	5.66	19.39	0.0012	0.0197
21 days	twi_ss.20099.1|g.25857	-4.76	2.06	30.32	0.0012	0.0198
21 days	twi_ss.2705.3|g.4930	3.70	4.39	19.34	0.0012	0.0199
21 days	twi_ss.14683.1|g.19128	1.40	5.12	19.33	0.0012	0.0199
21 days	twi_ss.30590.3|g.41404	7.24	3.84	19.32	0.0012	0.0199
21 days	twi_ss.6267.1|g.9312	-6.80	3.22	30.20	0.0012	0.0200
21 days	twi_ss.26435.1|g.35043	5.55	4.03	19.29	0.0012	0.0200
21 days	twi_ss.28809.1|g.38538	2.45	4.74	19.29	0.0012	0.0200
21 days	twi_ss.27170b.1|g.36115	0.98	7.08	19.28	0.0012	0.0200
21 days	twi_ss.4335.1|g.6842	2.77	6.39	19.28	0.0012	0.0200
21 days	twi_ss.4684.1|g.7239	0.91	5.82	19.28	0.0012	0.0200
21 days	twi_ss.6820.2|g.9882	1.38	5.46	19.28	0.0012	0.0200
21 days	twi_ss.22743.5|g.29082	-4.61	4.03	19.27	0.0012	0.0200
21 days	twi_ss.31368.1|g.42696	1.62	4.45	19.25	0.0012	0.0201
21 days	twi_ss.5881.1|g.8746	0.56	7.72	19.25	0.0012	0.0201
21 days	twi_ss.10276.1|g.13757	1.04	6.35	19.24	0.0012	0.0201
21 days	twi_ss.12345.1|g.16456	0.83	5.87	19.24	0.0012	0.0201
21 days	twi_ss.15008.1|g.19493	0.64	8.16	19.23	0.0012	0.0201
21 days	twi_ss.20948.1|g.27058	1.72	4.28	19.23	0.0012	0.0201
21 days	twi_ss.23229.1|g.29893	-7.49	3.70	22.65	0.0012	0.0202
21 days	twi_ss.15148.1|g.19668	-2.52	4.28	19.21	0.0012	0.0202
21 days	twi_ss.28214.1|g.37656	1.82	5.22	19.21	0.0012	0.0202
21 days	twi_ss.31391.1|g.42759	-1.99	4.41	19.20	0.0012	0.0202
21 days	twi_ss.8695.1|g.11850	-3.23	3.83	19.20	0.0013	0.0202
21 days	twi_ss.29153.1|g.39101	0.88	6.85	19.19	0.0013	0.0202
21 days	twi_ss.25841.1|g.34037	1.00	8.05	19.18	0.0013	0.0203
21 days	twi_ss.13318.1|g.17677	0.70	6.64	19.15	0.0013	0.0203
21 days	twi_ss.18431.1|g.23473	1.30	4.86	19.15	0.0013	0.0203
21 days	twi_ss.15568.1|g.20148	-5.91	2.76	22.55	0.0013	0.0204
21 days	twi_ss.24188.1|g.31533	-3.60	3.67	19.10	0.0013	0.0205

21 days	twi_ss.941.2|g.1597	-3.04	5.40	19.08	0.0013	0.0206
21 days	twi_ss.2154.1|g.3839	-0.88	6.47	19.07	0.0013	0.0206
21 days	twi_ss.13146.5|g.17470	2.53	5.37	19.06	0.0013	0.0206
21 days	twi_ss.18204.1|g.23100	0.92	7.83	19.06	0.0013	0.0206
21 days	twi_ss.30890.1|g.41957	0.93	7.10	19.06	0.0013	0.0206
21 days	twi_ss.24119.1|g.31413	-3.17	3.81	19.05	0.0013	0.0206
21 days	twi_ss.29623.4|g.39905	-3.61	5.64	19.05	0.0013	0.0206
21 days	twi_ss.28439.1|g.38039	0.74	8.53	19.04	0.0013	0.0207
21 days	twi_ss.24651.1|g.32283	-0.83	8.08	19.03	0.0013	0.0207
21 days	twi_ss.28220a.6|g.37616	-2.11	4.81	18.96	0.0013	0.0209
21 days	twi_ss.29581.3|g.39841	4.20	1.88	18.96	0.0013	0.0209
21 days	twi_ss.3480b.3|g.5862	-7.09	3.31	18.96	0.0013	0.0209
21 days	twi_ss.24232.1|g.31682	-1.49	4.68	18.95	0.0013	0.0209
21 days	twi_ss.1434.1|g.2549	3.64	3.43	18.94	0.0013	0.0209
21 days	twi_ss.2277.1|g.4031	1.10	5.56	18.94	0.0013	0.0209
21 days	twi_ss.25690.1|g.33874	-3.69	3.85	18.91	0.0013	0.0211
21 days	twi_ss.26087.1|g.34611	1.70	4.34	18.91	0.0013	0.0211
21 days	twi_ss.18704.1|g.23877	0.43	9.08	18.87	0.0013	0.0212
21 days	twi_ss.1238.1|g.2207	-5.05	2.74	22.19	0.0013	0.0212
21 days	twi_ss.18405.1|g.23467	-2.75	5.33	18.86	0.0013	0.0212
21 days	twi_ss.31439.1|g.42811	-2.74	5.14	18.86	0.0013	0.0212
21 days	twi_ss.4304a.1|g.6726	2.01	5.36	18.83	0.0013	0.0213
21 days	twi_ss.17563.1|g.22348	-1.45	6.92	18.82	0.0013	0.0213
21 days	twi_ss.21234.1|g.27455	1.57	4.54	18.81	0.0013	0.0213
21 days	twi_ss.2690.2|g.4896	1.56	7.59	18.82	0.0013	0.0213
21 days	twi_ss.7761.1|g.10814	1.26	6.04	18.80	0.0013	0.0214
21 days	twi_ss.805.1|g.1398	0.83	7.93	18.79	0.0013	0.0214
21 days	twi_ss.1489.2|g.2552	-6.83	3.14	18.77	0.0014	0.0214
21 days	twi_ss.15156.3|g.19688	-7.33	3.46	18.76	0.0014	0.0214
21 days	twi_ss.22774.1|g.29404	-2.38	3.32	18.76	0.0014	0.0214
21 days	twi_ss.23138.1|g.29797	-1.52	6.55	18.76	0.0014	0.0214
21 days	twi_ss.27995.1|g.37282	-1.37	5.30	18.77	0.0014	0.0214
21 days	twi_ss.6167a.1|g.9147	1.27	4.72	18.77	0.0014	0.0214

21 days	twi_ss.16822.1|g.21483	3.04	2.82	18.74	0.0014	0.0215
21 days	twi_ss.21303.1|g.27511	-1.71	4.63	18.72	0.0014	0.0216
21 days	twi_ss.21675.1|g.28101	-2.36	7.11	18.72	0.0014	0.0216
21 days	twi_ss.26048.1|g.34534	-5.78	2.62	22.00	0.0014	0.0216
21 days	twi_ss.17195.1|g.21952	-1.96	5.10	18.71	0.0014	0.0216
21 days	twi_ss.888.2|g.1581	-4.85	2.60	21.99	0.0014	0.0216
21 days	twi_ss.28611.1|g.38310	-1.54	4.62	18.70	0.0014	0.0216
21 days	twi_ss.4370.1|g.6876	2.29	6.49	18.70	0.0014	0.0216
21 days	twi_ss.8465.1|g.11549	0.89	6.65	18.69	0.0014	0.0216
21 days	twi_ss.19744.1|g.25408	-1.85	3.80	18.69	0.0014	0.0216
21 days	twi_ss.6201b.2|g.9010	-3.62	3.94	18.69	0.0014	0.0216
21 days	twi_ss.23542.1|g.30223	0.93	6.25	18.67	0.0014	0.0216
21 days	twi_ss.28008.1|g.37358	0.63	7.20	18.68	0.0014	0.0216
21 days	twi_ss.29015.1|g.38926	0.95	6.18	18.67	0.0014	0.0216
21 days	twi_ss.11555.4|g.15340	-2.84	3.71	18.67	0.0014	0.0217
21 days	twi_ss.4180.1|g.6599	3.06	3.75	18.66	0.0014	0.0217
21 days	twi_ss.1522.1|g.2716	-1.67	6.77	18.63	0.0014	0.0218
21 days	twi_ss.25749.1|g.34010	-1.97	3.92	18.62	0.0014	0.0218
21 days	twi_ss.4097.1|g.6512	-1.26	5.99	18.63	0.0014	0.0218
21 days	twi_ss.11614.1|g.15414	0.88	8.25	18.61	0.0014	0.0218
21 days	twi_ss.11948.1|g.15813	-1.07	8.07	18.62	0.0014	0.0218
21 days	twi_ss.21999.1|g.28496	-2.28	5.43	18.61	0.0014	0.0218
21 days	twi_ss.29504.1|g.39730	1.43	5.26	18.62	0.0014	0.0218
21 days	twi_ss.20220.1|g.26001	1.17	5.36	18.59	0.0014	0.0219
21 days	twi_ss.14245.1|g.18537	0.92	6.54	18.59	0.0014	0.0219
21 days	twi_ss.16733.1|g.21393	0.74	7.00	18.57	0.0014	0.0219
21 days	twi_ss.17345.1|g.22145	-3.04	4.64	18.58	0.0014	0.0219
21 days	twi_ss.27361.1|g.36419	2.07	3.74	18.57	0.0014	0.0219
21 days	twi_ss.5054.1|g.7704	1.57	4.74	18.57	0.0014	0.0219
21 days	twi_ss.11098.1|g.14735	-2.16	3.91	18.56	0.0014	0.0219
21 days	twi_ss.2525.1|g.4549	1.18	5.92	18.56	0.0014	0.0219
21 days	twi_ss.12990.2|g.17197	6.01	3.59	18.53	0.0014	0.0220
21 days	twi_ss.12990.3|g.17194	6.01	3.59	18.53	0.0014	0.0220

21 days	twi_ss.15132.1|g.19660	3.05	2.93	18.52	0.0014	0.0221
21 days	twi_ss.21625.1|g.28032	1.26	6.44	18.51	0.0014	0.0221
21 days	twi_ss.12247.1|g.16395	3.75	3.98	18.50	0.0014	0.0221
21 days	twi_ss.14536.1|g.18999	-4.82	2.45	21.70	0.0014	0.0221
21 days	twi_ss.23954.1|g.31137	-1.61	4.59	18.48	0.0014	0.0221
21 days	twi_ss.31322.1|g.42624	-1.39	5.66	18.49	0.0014	0.0221
21 days	twi_ss.23359.1|g.30032	2.06	5.02	18.47	0.0014	0.0222
21 days	twi_ss.27783b.3|g.36949	-5.25	5.40	18.48	0.0014	0.0222
21 days	twi_ss.10431.1|g.14069	-1.73	5.13	18.47	0.0014	0.0222
21 days	twi_ss.31529.1|g.42917	0.72	6.47	18.47	0.0014	0.0222
21 days	twi_ss.1156.2|g.2061	-1.27	5.84	18.46	0.0014	0.0222
21 days	twi_ss.18318.1|g.23342	1.01	5.69	18.46	0.0014	0.0222
21 days	twi_ss.25244.4|g.33174	0.99	7.51	18.46	0.0014	0.0222
21 days	twi_ss.8331.2|g.11450	-3.14	7.29	18.46	0.0014	0.0222
21 days	twi_ss.14343.4|g.18656	-1.25	5.33	18.45	0.0014	0.0222
21 days	twi_ss.3645.1|g.6020	0.59	7.42	18.45	0.0014	0.0222
21 days	twi_ss.13574.1|g.17954	-3.82	3.49	18.44	0.0014	0.0222
21 days	twi_ss.14215.1|g.18531	-2.67	4.50	18.44	0.0014	0.0222
21 days	twi_ss.22349.1|g.28871	1.07	6.87	18.40	0.0014	0.0223
21 days	twi_ss.14643.1|g.19097	-5.25	2.42	28.36	0.0015	0.0224
21 days	twi_ss.1679.1|g.2993	1.08	5.15	18.39	0.0015	0.0224
21 days	twi_ss.6681.1|g.9714	-1.34	6.70	18.39	0.0015	0.0224
21 days	twi_ss.10703.10|g.14318	-1.12	5.29	18.38	0.0015	0.0224
21 days	twi_ss.19620.1|g.25207	1.20	5.49	18.37	0.0015	0.0224
21 days	twi_ss.19627.1|g.25179	0.95	10.03	18.37	0.0015	0.0224
21 days	twi_ss.2886.1|g.5173	-7.28	3.41	28.33	0.0015	0.0224
21 days	twi_ss.16684.1|g.21353	0.97	5.40	18.36	0.0015	0.0224
21 days	twi_ss.6316.1|g.9338	0.79	8.96	18.35	0.0015	0.0224
21 days	twi_ss.18353.1|g.23364	1.44	4.44	18.35	0.0015	0.0224
21 days	twi_ss.28509a.5|g.38119	-2.06	4.64	18.35	0.0015	0.0224
21 days	twi_ss.25478.1|g.33559	-5.19	3.05	21.46	0.0015	0.0226
21 days	twi_ss.28801.1|g.38539	-1.46	5.09	18.30	0.0015	0.0226
21 days	twi_ss.202.1|g.416	0.67	5.91	18.29	0.0015	0.0226

21 days	twi_ss.7685.1|g.10766	-1.89	4.01	18.30	0.0015	0.0226
21 days	twi_ss.18795.3|g.24038	3.06	8.19	18.29	0.0015	0.0226
21 days	twi_ss.14290.1|g.18558	0.69	5.96	18.27	0.0015	0.0226
21 days	twi_ss.14839.1|g.19294	5.48	3.62	18.28	0.0015	0.0226
21 days	twi_ss.1766.1|g.3105	1.41	6.37	18.28	0.0015	0.0226
21 days	twi_ss.25600.1|g.33726	0.84	5.41	18.28	0.0015	0.0226
21 days	twi_ss.26979b.2|g.35827	0.87	6.44	18.27	0.0015	0.0226
21 days	twi_ss.7115.1|g.10182	-2.02	5.46	18.28	0.0015	0.0226
21 days	twi_ss.849.1|g.1397	-0.94	5.99	18.26	0.0015	0.0227
21 days	twi_ss.13862.1|g.18177	1.32	4.54	18.25	0.0015	0.0227
21 days	twi_ss.20616.1|g.26644	-4.54	2.31	21.37	0.0015	0.0228
21 days	twi_ss.28292.9|g.37672	-3.52	4.11	18.23	0.0015	0.0228
21 days	twi_ss.18919.1|g.24236	1.03	5.48	18.23	0.0015	0.0228
21 days	twi_ss.14316.1|g.18666	0.86	7.01	18.21	0.0015	0.0228
21 days	twi_ss.25028.1|g.32853	-3.30	4.20	18.20	0.0015	0.0229
21 days	twi_ss.3010.1|g.5362	0.72	7.77	18.20	0.0015	0.0229
21 days	twi_ss.10703.2|g.14314	5.25	2.25	18.15	0.0015	0.0230
21 days	twi_ss.1676.1|g.2968	-2.06	4.87	18.15	0.0015	0.0230
21 days	twi_ss.3531.2|g.5898	2.61	6.68	18.15	0.0015	0.0230
21 days	twi_ss.5357.1|g.8107	0.93	6.95	18.15	0.0015	0.0230
21 days	twi_ss.16870.1|g.21502	-1.14	5.69	18.12	0.0015	0.0231
21 days	twi_ss.23994b.2|g.31227	-3.27	3.27	18.12	0.0015	0.0231
21 days	twi_ss.4630.1|g.7168	-1.90	4.86	18.13	0.0015	0.0231
21 days	twi_ss.19678.1|g.25351	-3.02	3.77	18.11	0.0015	0.0232
21 days	twi_ss.8262.1|g.11331	-5.49	2.65	21.21	0.0015	0.0232
21 days	twi_ss.4253.1|g.6682	-1.96	4.31	18.10	0.0015	0.0232
21 days	twi_ss.1582.1|g.2828	1.08	5.25	18.10	0.0015	0.0232
21 days	twi_ss.8411.3|g.11569	-0.94	5.09	18.09	0.0015	0.0232
21 days	twi_ss.14840.1|g.19297	2.12	3.90	18.07	0.0015	0.0233
21 days	twi_ss.10034.1|g.13353	-7.57	4.09	18.04	0.0016	0.0234
21 days	twi_ss.19030.10|g.24349	-10.12	6.39	18.02	0.0016	0.0235
21 days	twi_ss.17695.1|g.22566	-1.99	3.55	18.01	0.0016	0.0235
21 days	twi_ss.234.1|g.476	-1.68	5.43	18.01	0.0016	0.0235

21 days	twi_ss.26324.1|g.34849	-0.82	5.66	18.01	0.0016	0.0235
21 days	twi_ss.9396.1|g.12708	-1.82	5.19	18.01	0.0016	0.0235
21 days	twi_ss.16056.1|g.20654	-3.37	4.00	17.97	0.0016	0.0236
21 days	twi_ss.31434.1|g.42809	-2.11	4.74	17.97	0.0016	0.0236
21 days	twi_ss.6264.1|g.9307	0.78	7.31	17.95	0.0016	0.0237
21 days	twi_ss.24282.1|g.31707	-3.26	4.53	17.94	0.0016	0.0237
21 days	twi_ss.2468b.1|g.4472	0.58	7.06	17.94	0.0016	0.0237
21 days	twi_ss.3025.1|g.5363	1.30	5.03	17.94	0.0016	0.0237
21 days	twi_ss.2847.2|g.5127	-2.31	4.01	17.94	0.0016	0.0237
21 days	twi_ss.24815.1|g.32609	1.42	5.55	17.92	0.0016	0.0238
21 days	twi_ss.28839.2|g.38607	-1.78	4.20	17.91	0.0016	0.0238
21 days	twi_ss.28569.1|g.38231	5.55	4.01	20.95	0.0016	0.0238
21 days	twi_ss.31915.4|g.43425	7.66	4.01	17.89	0.0016	0.0239
21 days	twi_ss.14134.1|g.18436	-4.48	3.44	17.88	0.0016	0.0239
21 days	twi_ss.3499.1|g.5870	-1.70	4.10	17.86	0.0016	0.0240
21 days	twi_ss.9313.1|g.12565	-2.48	7.04	17.86	0.0016	0.0240
21 days	twi_ss.27952.1|g.37207	-3.84	4.99	17.85	0.0016	0.0240
21 days	twi_ss.25032.1|g.32863	-4.07	3.23	17.84	0.0016	0.0241
21 days	twi_ss.25125.1|g.32989	0.88	7.64	17.83	0.0016	0.0241
21 days	twi_ss.3916.1|g.6338	-2.78	3.44	17.83	0.0016	0.0241
21 days	twi_ss.7233.1|g.10275	-2.17	3.74	17.83	0.0016	0.0241
21 days	twi_ss.26267.1|g.34786	0.97	5.96	17.82	0.0016	0.0241
21 days	twi_ss.30581.1|g.41429	-1.46	5.11	17.81	0.0016	0.0241
21 days	twi_ss.30956.1|g.42004	-0.85	7.64	17.80	0.0016	0.0242
21 days	twi_ss.8241.1|g.11315	-4.36	2.95	17.80	0.0016	0.0242
21 days	twi_ss.17097.1|g.21887	-4.47	4.33	17.75	0.0016	0.0244
21 days	twi_ss.28341.1|g.37847	0.76	6.15	17.75	0.0016	0.0244
21 days	twi_ss.17134.1|g.21914	-1.29	5.21	17.73	0.0016	0.0245
21 days	twi_ss.18451.1|g.23500	0.89	6.31	17.71	0.0017	0.0245
21 days	twi_ss.13292.1|g.17628	-6.37	3.12	17.70	0.0017	0.0246
21 days	twi_ss.17531b.1|g.22398	1.43	7.62	17.69	0.0017	0.0246
21 days	twi_ss.31088b.4|g.42266	-1.36	4.74	17.69	0.0017	0.0246
21 days	twi_ss.12246.2|g.16397	4.20	6.65	17.68	0.0017	0.0246

21 days	twi_ss.8819.1|g.12011	5.19	3.28	17.68	0.0017	0.0246
21 days	twi_ss.12008.1|g.15934	0.84	6.44	17.66	0.0017	0.0246
21 days	twi_ss.1702.1|g.3002	-4.38	3.50	17.67	0.0017	0.0246
21 days	twi_ss.4149.1|g.6565	0.58	9.21	17.66	0.0017	0.0246
21 days	twi_ss.5623.1|g.8372	-3.15	2.93	17.67	0.0017	0.0246
21 days	twi_ss.810.1|g.1384	1.18	6.41	17.66	0.0017	0.0246
21 days	twi_ss.20177.1|g.25924	-1.52	4.90	17.64	0.0017	0.0247
21 days	twi_ss.26905.1|g.35685	-1.47	5.60	17.64	0.0017	0.0247
21 days	twi_ss.4816.1|g.7395	5.35	2.64	20.60	0.0017	0.0247
21 days	twi_ss.8513.1|g.11673	1.16	6.48	17.64	0.0017	0.0247
21 days	twi_ss.23606.1|g.30403	0.83	7.00	17.62	0.0017	0.0248
21 days	twi_ss.30545.1|g.41375	0.83	6.30	17.62	0.0017	0.0248
21 days	twi_ss.16975.5|g.21623	1.33	6.45	17.62	0.0017	0.0248
21 days	twi_ss.4582a.1|g.7108	-3.34	3.53	17.62	0.0017	0.0248
21 days	twi_ss.10467.1|g.14103	0.97	5.97	17.60	0.0017	0.0248
21 days	twi_ss.10667.1|g.14302	-4.73	2.12	20.55	0.0017	0.0248
21 days	twi_ss.13620.1|g.17972	1.86	5.02	17.60	0.0017	0.0248
21 days	twi_ss.28338.2|g.37861	4.68	2.22	18.85	0.0017	0.0248
21 days	twi_ss.31134.2|g.42327	-6.23	3.92	17.59	0.0017	0.0248
21 days	twi_ss.49a.1|g.160	-5.93	2.62	20.54	0.0017	0.0248
21 days	twi_ss.2942.1|g.5261	-0.89	8.77	17.58	0.0017	0.0249
21 days	twi_ss.23992.1|g.31228	0.76	6.01	17.54	0.0017	0.0250
21 days	twi_ss.24737.1|g.32313	0.73	7.85	17.54	0.0017	0.0250
21 days	twi_ss.3847.1|g.6276	5.36	3.33	17.53	0.0017	0.0251
21 days	twi_ss.24834.1|g.32673	0.85	6.39	17.51	0.0017	0.0252
21 days	twi_ss.4291.1|g.6725	0.83	7.51	17.51	0.0017	0.0252
21 days	twi_ss.27680.1|g.36876	1.11	5.35	17.49	0.0017	0.0252
21 days	twi_ss.31951.1|g.43532	1.80	4.14	17.49	0.0017	0.0252
21 days	twi_ss.31583b.6|g.43008	-4.00	3.97	17.45	0.0017	0.0254
21 days	twi_ss.684.1|g.1227	2.58	3.42	17.45	0.0017	0.0254
21 days	twi_ss.30671.1|g.41559	-1.23	5.17	17.44	0.0017	0.0254
21 days	twi_ss.21657.1|g.28088	1.76	5.80	17.44	0.0017	0.0254
21 days	twi_ss.6274b.5|g.9297	0.43	7.53	17.44	0.0017	0.0254

21 days	twi_ss.11257.1|g.14893	-1.42	5.36	17.43	0.0017	0.0254
21 days	twi_ss.19822b.1|g.25495	-2.65	5.27	17.43	0.0017	0.0254
21 days	twi_ss.20376.3|g.26244	-3.18	7.97	17.42	0.0017	0.0254
21 days	twi_ss.23459.1|g.30108	1.42	6.53	17.42	0.0017	0.0254
21 days	twi_ss.19345.11|g.24815	6.18	3.23	17.41	0.0018	0.0255
21 days	twi_ss.6158.1|g.9045	-2.98	4.05	17.40	0.0018	0.0255
21 days	twi_ss.27688.1|g.36899	0.87	5.45	17.38	0.0018	0.0256
21 days	twi_ss.20484.1|g.26433	0.78	6.43	17.38	0.0018	0.0256
21 days	twi_ss.8827.1|g.12015	-1.05	5.27	17.35	0.0018	0.0257
21 days	twi_ss.26090b.4|g.34552	5.21	2.72	17.35	0.0018	0.0257
21 days	twi_ss.10324.1|g.13924	-2.09	4.33	17.34	0.0018	0.0258
21 days	twi_ss.19030.3|g.24370	-1.96	9.66	17.33	0.0018	0.0258
21 days	twi_ss.20172.1|g.25984	1.27	5.59	17.33	0.0018	0.0258
21 days	twi_ss.2278.1|g.4035	0.85	6.07	17.32	0.0018	0.0258
21 days	twi_ss.6793.2|g.9851	-1.66	4.13	17.31	0.0018	0.0258
21 days	twi_ss.19030.2|g.24404	-10.72	7.13	20.18	0.0018	0.0259
21 days	twi_ss.29822.1|g.40226	0.98	8.42	17.28	0.0018	0.0260
21 days	twi_ss.11840.1|g.15729	-2.16	4.07	17.28	0.0018	0.0260
21 days	twi_ss.11765.1|g.15581	-2.13	4.15	17.26	0.0018	0.0261
21 days	twi_ss.13698.2|g.18023	1.78	5.46	17.26	0.0018	0.0261
21 days	twi_ss.2800a.2|g.5025	1.27	9.06	17.25	0.0018	0.0261
21 days	twi_ss.492a.1|g.889	0.68	6.99	17.25	0.0018	0.0261
21 days	twi_ss.31062.2|g.42240	-6.38	2.86	17.23	0.0018	0.0262
21 days	twi_ss.29590a.5|g.39853	3.21	6.74	17.22	0.0018	0.0262
21 days	twi_ss.13045.2|g.17295	-0.83	7.30	17.21	0.0018	0.0262
21 days	twi_ss.13937.2|g.18266	-4.76	2.20	26.04	0.0018	0.0262
21 days	twi_ss.441.1|g.824	-2.02	3.82	17.21	0.0018	0.0262
21 days	twi_ss.6244b.3|g.9269	-2.19	3.63	17.20	0.0018	0.0263
21 days	twi_ss.12094b.2|g.15993	-1.94	4.10	17.20	0.0018	0.0263
21 days	twi_ss.10600.1|g.14246	-4.37	2.41	25.10	0.0018	0.0263
21 days	twi_ss.21076.1|g.27226	-0.71	6.23	17.18	0.0018	0.0264
21 days	twi_ss.7777.2|g.10855	-1.42	6.31	17.17	0.0018	0.0264
21 days	twi_ss.9882.1|g.13208	0.85	7.18	17.17	0.0018	0.0264

21 days	twi_ss.20680.1|g.26793	-5.35	3.33	19.99	0.0018	0.0264
21 days	twi_ss.15834.1|g.20405	-2.27	4.04	17.14	0.0019	0.0265
21 days	twi_ss.1103.1|g.1963	-1.98	5.34	17.13	0.0019	0.0265
21 days	twi_ss.8555.1|g.11707	1.93	4.67	17.13	0.0019	0.0265
21 days	twi_ss.30027c.4|g.40535	2.62	6.28	17.12	0.0019	0.0266
21 days	twi_ss.90.1|g.115	0.69	7.95	17.11	0.0019	0.0266
21 days	twi_ss.28917.1|g.38791	-2.04	3.65	17.10	0.0019	0.0267
21 days	twi_ss.227.1|g.434	0.91	5.08	17.09	0.0019	0.0267
21 days	twi_ss.29074.1|g.38985	1.97	3.63	17.08	0.0019	0.0267
21 days	twi_ss.2039.1|g.3559	-0.72	6.12	17.08	0.0019	0.0267
21 days	twi_ss.30694.1|g.41598	0.98	7.56	17.07	0.0019	0.0267
21 days	twi_ss.24130.1|g.31519	-2.38	4.98	17.07	0.0019	0.0267
21 days	twi_ss.18252.3|g.23229	-4.84	4.43	17.06	0.0019	0.0268
21 days	twi_ss.18736.1|g.23916	0.84	7.13	17.06	0.0019	0.0268
21 days	twi_ss.30747.1|g.41683	-4.07	3.30	17.00	0.0019	0.0270
21 days	twi_ss.31626.2|g.43067	-1.17	7.15	17.00	0.0019	0.0270
21 days	twi_ss.12311.1|g.16436	1.07	6.07	17.00	0.0019	0.0270
21 days	twi_ss.29431.1|g.39588	0.63	6.81	17.00	0.0019	0.0271
21 days	twi_ss.4602.1|g.7125	-1.77	4.30	16.99	0.0019	0.0271
21 days	twi_ss.6255.1|g.9309	0.64	7.04	16.98	0.0019	0.0271
21 days	twi_ss.23655.1|g.30479	-2.08	5.97	16.96	0.0019	0.0272
21 days	twi_ss.10110.1|g.13599	0.78	7.03	16.95	0.0019	0.0272
21 days	twi_ss.10473.1|g.14116	0.78	7.77	16.95	0.0019	0.0273
21 days	twi_ss.25992.1|g.34326	-1.83	4.74	16.94	0.0019	0.0273
21 days	twi_ss.182.1|g.283	-7.05	3.45	19.69	0.0019	0.0273
21 days	twi_ss.15261.1|g.19837	-2.52	4.21	16.93	0.0019	0.0273
21 days	twi_ss.15708.1|g.20284	1.42	5.13	16.88	0.0019	0.0276
21 days	twi_ss.25767.1|g.34012	0.75	8.60	16.88	0.0019	0.0276
21 days	twi_ss.9052.1|g.12228	-1.51	4.70	16.87	0.0020	0.0276
21 days	twi_ss.12297.1|g.16283	0.60	6.88	16.84	0.0020	0.0278
21 days	twi_ss.29577.1|g.39830	-5.22	2.43	25.33	0.0020	0.0278
21 days	twi_ss.9992.1|g.13302	-0.88	5.45	16.83	0.0020	0.0278
21 days	twi_ss.9797.1|g.13128	-3.06	4.44	16.81	0.0020	0.0279

21 days	twi_ss.9479.1|g.12731	-2.00	4.06	16.81	0.0020	0.0279
21 days	twi_ss.2426.1|g.4306	-2.21	4.34	16.80	0.0020	0.0279
21 days	twi_ss.30222a.5|g.40814	-5.50	2.37	16.80	0.0020	0.0279
21 days	twi_ss.16070.1|g.20685	-3.72	3.21	16.80	0.0020	0.0279
21 days	twi_ss.5718.1|g.8555	2.23	3.91	16.79	0.0020	0.0279
21 days	twi_ss.20445.7|g.26343	2.05	4.20	16.78	0.0020	0.0280
21 days	twi_ss.7624.1|g.10679	0.76	7.52	16.78	0.0020	0.0280
21 days	twi_ss.29623.7|g.39906	-3.63	3.41	16.78	0.0020	0.0280
21 days	twi_ss.17564a.2|g.22358	0.76	6.57	16.76	0.0020	0.0280
21 days	twi_ss.5603.1|g.8310	-3.51	3.80	16.74	0.0020	0.0282
21 days	twi_ss.7928.1|g.10984	0.94	5.54	16.74	0.0020	0.0282
21 days	twi_ss.16979.1|g.21658	1.02	5.43	16.73	0.0020	0.0282
21 days	twi_ss.13146.1|g.17471	2.22	4.88	16.71	0.0020	0.0282
21 days	twi_ss.14941.1|g.19485	1.61	4.14	16.71	0.0020	0.0282
21 days	twi_ss.17415.1|g.22224	1.71	4.60	16.72	0.0020	0.0282
21 days	twi_ss.20183.1|g.25921	0.85	6.44	16.71	0.0020	0.0282
21 days	twi_ss.31962.2|g.43579	-4.57	3.57	16.71	0.0020	0.0282
21 days	twi_ss.10990b.2|g.14585	0.92	6.89	16.70	0.0020	0.0283
21 days	twi_ss.11062.1|g.14674	0.57	8.48	16.70	0.0020	0.0283
21 days	twi_ss.12285.1|g.16340	-2.00	4.47	16.70	0.0020	0.0283
21 days	twi_ss.12609.2|g.16717	-5.75	2.72	16.69	0.0020	0.0283
21 days	twi_ss.10705.1|g.14361	-7.22	4.48	16.69	0.0020	0.0283
21 days	twi_ss.8746.1|g.11956	-1.41	4.66	16.68	0.0020	0.0283
21 days	twi_ss.29169b.4|g.39088	0.70	9.29	16.67	0.0020	0.0284
21 days	twi_ss.25419.1|g.33440	-3.62	3.22	16.66	0.0020	0.0284
21 days	twi_ss.30409.1|g.41239	-1.07	6.28	16.64	0.0020	0.0285
21 days	twi_ss.2758.1|g.5058	0.79	6.78	16.64	0.0020	0.0285
21 days	twi_ss.25677.1|g.33857	-1.63	6.35	16.63	0.0020	0.0285
21 days	twi_ss.26713.1|g.35408	-1.33	6.14	16.63	0.0020	0.0285
21 days	twi_ss.28992.1|g.38909	-3.07	4.50	16.62	0.0020	0.0286
21 days	twi_ss.30418.3|g.41143	0.88	6.18	16.58	0.0021	0.0288
21 days	twi_ss.18252.4|g.23231	-4.02	4.47	16.58	0.0021	0.0288
21 days	twi_ss.5738.2|g.8559	1.98	7.96	16.55	0.0021	0.0289

21 days	twi_ss.13944a.4|g.18232	0.87	8.29	16.54	0.0021	0.0290
21 days	twi_ss.1369.1|g.2498	-1.27	4.67	16.53	0.0021	0.0290
21 days	twi_ss.19093.2|g.24483	-1.28	5.74	16.53	0.0021	0.0290
21 days	twi_ss.25214.1|g.33129	-0.90	5.59	16.52	0.0021	0.0291
21 days	twi_ss.28749.1|g.38494	-3.78	3.37	16.51	0.0021	0.0291
21 days	twi_ss.26404.1|g.34972	2.60	3.19	16.50	0.0021	0.0291
21 days	twi_ss.6299.1|g.9323	-2.54	6.05	16.50	0.0021	0.0291
21 days	twi_ss.23103.1|g.29736	0.73	6.44	16.50	0.0021	0.0292
21 days	twi_ss.15005a.1|g.19501	1.70	5.67	16.49	0.0021	0.0292
21 days	twi_ss.12294.1|g.16393	0.72	6.99	16.49	0.0021	0.0292
21 days	twi_ss.30766.1|g.41759	4.64	2.12	16.49	0.0021	0.0292
21 days	twi_ss.21664c.11|g.27914	2.36	8.69	16.48	0.0021	0.0292
21 days	twi_ss.2617.2|g.4710	-1.39	4.73	16.48	0.0021	0.0292
21 days	twi_ss.446.1|g.844	-2.55	4.16	16.48	0.0021	0.0292
21 days	twi_ss.21156.2|g.27391	-4.42	2.66	19.11	0.0021	0.0292
21 days	twi_ss.7286.1|g.10310	-0.69	5.48	16.47	0.0021	0.0292
21 days	twi_ss.24240.1|g.31631	2.70	4.74	16.44	0.0021	0.0293
21 days	twi_ss.17394.1|g.22186	-1.92	5.16	16.43	0.0021	0.0294
21 days	twi_ss.25105.5|g.32924	6.71	3.87	16.43	0.0021	0.0294
21 days	twi_ss.30850.1|g.42013	0.84	5.54	16.43	0.0021	0.0294
21 days	twi_ss.29229.1|g.39186	-3.17	4.00	16.42	0.0021	0.0294
21 days	twi_ss.6499.1|g.9526	2.60	3.73	16.41	0.0021	0.0294
21 days	twi_ss.6713.1|g.9785	-0.99	7.12	16.41	0.0021	0.0294
21 days	twi_ss.15985.1|g.20601	-2.63	4.86	16.40	0.0021	0.0295
21 days	twi_ss.6594.1|g.9621	0.75	5.54	16.40	0.0021	0.0295
21 days	twi_ss.339.1|g.675	3.18	3.79	16.39	0.0021	0.0295
21 days	twi_ss.28981.1|g.38896	-1.39	5.37	16.39	0.0022	0.0296
21 days	twi_ss.12960.1|g.16989	0.73	6.25	16.38	0.0022	0.0296
21 days	twi_ss.27854.5|g.37041	-1.14	7.13	16.38	0.0022	0.0296
21 days	twi_ss.28592.1|g.38300	1.86	4.04	16.37	0.0022	0.0296
21 days	twi_ss.10679.3|g.14341	-5.89	2.57	16.36	0.0022	0.0296
21 days	twi_ss.20047b.1|g.25828	1.10	5.89	16.36	0.0022	0.0296
21 days	twi_ss.5314b.1|g.7997	-3.73	6.29	16.36	0.0022	0.0296

21 days	twi_ss.946.4|g.1710	-4.30	7.05	16.36	0.0022	0.0296
21 days	twi_ss.6152b.1|g.9127	0.46	8.40	16.35	0.0022	0.0297
21 days	twi_ss.13045.1|g.17294	-4.69	4.00	16.35	0.0022	0.0297
21 days	twi_ss.30032.1|g.40525	-1.08	7.40	16.34	0.0022	0.0297
21 days	twi_ss.25618.1|g.33704	-2.12	4.05	16.34	0.0022	0.0297
21 days	twi_ss.24588.1|g.32236	-3.72	2.91	16.34	0.0022	0.0297
21 days	twi_ss.8089.1|g.11160	-1.18	5.02	16.33	0.0022	0.0297
21 days	twi_ss.20754.3|g.26862	-7.91	4.08	24.34	0.0022	0.0297
21 days	twi_ss.11460.1|g.15113	0.99	5.17	16.31	0.0022	0.0298
21 days	twi_ss.26609.10|g.35265	-0.71	7.74	16.28	0.0022	0.0300
21 days	twi_ss.10714.1|g.14363	-1.08	5.37	16.26	0.0022	0.0301
21 days	twi_ss.22735.2|g.29344	-5.49	2.34	16.25	0.0022	0.0301
21 days	twi_ss.26806.2|g.35580	-4.96	2.15	16.25	0.0022	0.0301
21 days	twi_ss.7573.1|g.10627	-2.15	4.32	16.25	0.0022	0.0301
21 days	twi_ss.22211.1|g.28781	-3.13	4.08	16.25	0.0022	0.0301
21 days	twi_ss.6051.1|g.8922	0.72	8.42	16.24	0.0022	0.0301
21 days	twi_ss.19827.1|g.25491	0.79	6.28	16.23	0.0022	0.0302
21 days	twi_ss.16993.1|g.21703	1.92	4.56	16.22	0.0022	0.0303
21 days	twi_ss.4977.3|g.7556	-4.72	4.56	16.21	0.0022	0.0303
21 days	twi_ss.10259.1|g.13908	-1.23	4.95	16.20	0.0022	0.0303
21 days	twi_ss.26448.1|g.35056	0.61	8.38	16.20	0.0022	0.0303
21 days	twi_ss.5977.2|g.8846	-2.02	3.79	16.20	0.0022	0.0303
21 days	twi_ss.2268.1|g.4010	-1.36	5.81	16.19	0.0022	0.0304
21 days	twi_ss.31343.1|g.42625	0.55	6.55	16.19	0.0022	0.0304
21 days	twi_ss.22524.1|g.29120	2.01	3.92	16.17	0.0022	0.0305
21 days	twi_ss.32019.1|g.43673	-5.33	2.40	16.16	0.0023	0.0305
21 days	twi_ss.1400a.2|g.2530	-1.42	6.23	16.15	0.0023	0.0305
21 days	twi_ss.19586.1|g.25171	-2.08	3.96	16.15	0.0023	0.0305
21 days	twi_ss.3131.1|g.5523	2.22	4.78	16.15	0.0023	0.0305
21 days	twi_ss.16707.1|g.21394	-3.22	4.42	16.14	0.0023	0.0306
21 days	twi_ss.27159a.4|g.36070	1.17	5.89	16.12	0.0023	0.0307
21 days	twi_ss.14765.1|g.19181	-0.91	5.81	16.11	0.0023	0.0307
21 days	twi_ss.28543.2|g.38250	-4.48	4.99	16.11	0.0023	0.0307

21 days	twi_ss.17488.1|g.22320	0.76	6.36	16.10	0.0023	0.0308
21 days	twi_ss.28510a.18|g.38068	-3.09	3.40	16.09	0.0023	0.0308
21 days	twi_ss.556.1|g.1016	-8.34	4.31	18.60	0.0023	0.0309
21 days	twi_ss.19561.1|g.25114	1.38	4.70	16.07	0.0023	0.0310
21 days	twi_ss.29416.1|g.39552	1.68	4.33	16.06	0.0023	0.0310
21 days	twi_ss.10273a.4|g.13876	1.53	5.60	16.05	0.0023	0.0310
21 days	twi_ss.28331.1|g.37892	1.00	7.32	16.04	0.0023	0.0311
21 days	twi_ss.29858.1|g.40290	6.28	3.25	16.03	0.0023	0.0311
21 days	twi_ss.6152b.2|g.9126	-1.13	6.76	16.03	0.0023	0.0311
21 days	twi_ss.4105.1|g.6453	-1.09	8.70	16.03	0.0023	0.0311
21 days	twi_ss.22288.1|g.28828	0.92	5.66	16.02	0.0023	0.0312
21 days	twi_ss.13190.1|g.17502	2.89	5.06	16.01	0.0023	0.0312
21 days	twi_ss.16552.1|g.21247	-4.46	3.19	16.01	0.0023	0.0312
21 days	twi_ss.17093.1|g.21889	-3.67	2.89	16.00	0.0023	0.0313
21 days	twi_ss.28038b.4|g.37330	2.27	5.47	15.99	0.0023	0.0313
21 days	twi_ss.30831.1|g.41849	-1.92	3.65	15.99	0.0023	0.0313
21 days	twi_ss.4535.1|g.7042	0.59	8.35	15.98	0.0023	0.0313
21 days	twi_ss.16730.1|g.21388	0.81	8.49	15.98	0.0023	0.0313
21 days	twi_ss.12421.2|g.16532	-1.91	5.22	15.97	0.0023	0.0313
21 days	twi_ss.9228.1|g.12463	-1.13	5.35	15.98	0.0023	0.0313
21 days	twi_ss.10802.1|g.14436	-1.56	4.66	15.97	0.0023	0.0313
21 days	twi_ss.5417.1|g.8185	-1.12	4.60	15.97	0.0023	0.0313
21 days	twi_ss.9995.1|g.13309	-5.58	2.85	18.46	0.0023	0.0313
21 days	twi_ss.1087.4|g.1915	-1.69	6.75	15.96	0.0023	0.0314
21 days	twi_ss.25295.1|g.33312	-3.69	2.44	19.47	0.0023	0.0314
21 days	twi_ss.24819a.9|g.32637	1.63	5.63	15.94	0.0024	0.0314
21 days	twi_ss.796.1|g.1394	-2.05	3.75	15.94	0.0024	0.0314
21 days	twi_ss.1217.1|g.2100	-4.31	3.36	18.42	0.0024	0.0314
21 days	twi_ss.15647.1|g.20216	-1.23	5.70	15.94	0.0024	0.0314
21 days	twi_ss.30863.2|g.41963	-5.76	2.47	15.94	0.0024	0.0314
21 days	twi_ss.4881.2|g.7483	-5.80	4.82	15.92	0.0024	0.0315
21 days	twi_ss.5603.2|g.8313	-5.66	2.70	18.40	0.0024	0.0315
21 days	twi_ss.16352.1|g.20943	0.86	5.20	15.90	0.0024	0.0316

21 days	twi_ss.19091.1|g.24504	2.07	3.28	15.91	0.0024	0.0316
21 days	twi_ss.29937.2|g.40387	-3.65	3.98	15.91	0.0024	0.0316
21 days	twi_ss.5730.2|g.8594	-4.98	2.14	15.90	0.0024	0.0316
21 days	twi_ss.1087.6|g.1920	-1.45	8.01	15.90	0.0024	0.0316
21 days	twi_ss.21063.1|g.27188	-0.89	5.61	15.90	0.0024	0.0316
21 days	twi_ss.21509.2|g.27865	-3.35	3.73	15.89	0.0024	0.0316
21 days	twi_ss.25550.1|g.33585	-3.45	3.51	15.89	0.0024	0.0316
21 days	twi_ss.10211.1|g.13735	-1.25	4.25	15.88	0.0024	0.0317
21 days	twi_ss.22996.1|g.29640	-2.86	3.73	15.87	0.0024	0.0317
21 days	twi_ss.27749.1|g.36955	-2.42	4.00	15.87	0.0024	0.0317
21 days	twi_ss.11050.1|g.14677	-1.94	4.10	15.86	0.0024	0.0318
21 days	twi_ss.26941.1|g.35762	-2.59	4.33	15.85	0.0024	0.0318
21 days	twi_ss.4312.1|g.6748	8.96	6.38	15.85	0.0024	0.0318
21 days	twi_ss.5558.1|g.8419	1.51	4.74	15.84	0.0024	0.0318
21 days	twi_ss.11017.1|g.14636	0.97	5.30	15.84	0.0024	0.0318
21 days	twi_ss.21120.1|g.27345	1.58	3.97	15.84	0.0024	0.0318
21 days	twi_ss.4126.1|g.6550	-2.60	2.97	15.84	0.0024	0.0318
21 days	twi_ss.30279.1|g.40923	1.21	6.88	15.83	0.0024	0.0319
21 days	twi_ss.16186.2|g.20767	-3.75	3.08	15.82	0.0024	0.0319
21 days	twi_ss.5522.1|g.8292	0.36	8.10	15.81	0.0024	0.0319
21 days	twi_ss.14799.3|g.19267	-4.55	3.57	15.80	0.0024	0.0320
21 days	twi_ss.29336.1|g.39439	-1.27	5.09	15.80	0.0024	0.0320
21 days	twi_ss.12479a.1|g.16643	-2.36	5.34	15.78	0.0024	0.0321
21 days	twi_ss.26344.1|g.34801	-3.29	3.39	15.78	0.0024	0.0321
21 days	twi_ss.627.1|g.1124	-4.73	2.50	15.77	0.0024	0.0322
21 days	twi_ss.10383.1|g.13962	-2.89	4.27	15.76	0.0024	0.0322
21 days	twi_ss.17899.1|g.22790	2.07	5.10	15.76	0.0024	0.0322
21 days	twi_ss.21739.1|g.28151	-3.67	3.42	15.75	0.0025	0.0323
21 days	twi_ss.507.4|g.922	-6.36	2.96	15.74	0.0025	0.0323
21 days	twi_ss.5399.1|g.8148	-1.33	4.06	15.74	0.0025	0.0323
21 days	twi_ss.31583a.3|g.42978	-1.67	5.19	15.74	0.0025	0.0323
21 days	twi_ss.14865.1|g.19308	1.35	5.02	15.74	0.0025	0.0323
21 days	twi_ss.30654b.2|g.41481	-4.45	3.55	15.74	0.0025	0.0323

21 days	twi_ss.5458.1|g.8213	0.67	6.34	15.73	0.0025	0.0323
21 days	twi_ss.22100.1|g.28615	-6.20	2.90	15.72	0.0025	0.0323
21 days	twi_ss.21769.1|g.28239	-2.25	5.83	15.72	0.0025	0.0324
21 days	twi_ss.16912.1|g.21554	-6.04	3.04	23.17	0.0025	0.0324
21 days	twi_ss.22651.1|g.29247	1.75	6.33	15.70	0.0025	0.0324
21 days	twi_ss.26574b.1|g.35220	-2.73	4.43	15.70	0.0025	0.0324
21 days	twi_ss.28510a.21|g.38091	-6.01	2.88	18.12	0.0025	0.0324
21 days	twi_ss.3009.2|g.5339	-2.54	5.25	15.70	0.0025	0.0324
21 days	twi_ss.3701.1|g.6078	0.85	5.76	15.71	0.0025	0.0324
21 days	twi_ss.10615.1|g.14264	-4.68	2.40	18.11	0.0025	0.0324
21 days	twi_ss.26863.1|g.35634	-1.37	4.55	15.69	0.0025	0.0324
21 days	twi_ss.5342.1|g.7953	-2.21	4.39	15.69	0.0025	0.0324
21 days	twi_ss.12694.1|g.16740	-1.93	4.01	15.68	0.0025	0.0325
21 days	twi_ss.13735.1|g.18068	-6.01	2.87	18.09	0.0025	0.0325
21 days	twi_ss.6432.1|g.9479	1.02	5.42	15.67	0.0025	0.0325
21 days	twi_ss.3755.1|g.6155	-1.15	6.29	15.67	0.0025	0.0325
21 days	twi_ss.8576.1|g.11730	1.58	4.75	15.67	0.0025	0.0325
21 days	twi_ss.21863.1|g.28360	-3.74	3.37	15.66	0.0025	0.0326
21 days	twi_ss.29624.3|g.39911	-5.70	2.84	15.65	0.0025	0.0326
21 days	twi_ss.228.1|g.435	1.11	5.10	15.64	0.0025	0.0327
21 days	twi_ss.2795.1|g.5006	0.83	6.49	15.62	0.0025	0.0328
21 days	twi_ss.13859.1|g.18173	-3.67	2.86	15.61	0.0025	0.0328
21 days	twi_ss.5805a.2|g.8720	3.35	4.40	15.60	0.0025	0.0328
21 days	twi_ss.16705.1|g.21381	-3.86	5.42	15.60	0.0025	0.0329
21 days	twi_ss.29986.1|g.40498	-4.05	3.65	15.59	0.0025	0.0329
21 days	twi_ss.22261.2|g.28815	-7.15	3.31	17.98	0.0025	0.0329
21 days	twi_ss.14260.2|g.18549	1.91	4.23	15.58	0.0025	0.0329
21 days	twi_ss.25647.1|g.33854	-5.73	2.82	17.97	0.0025	0.0329
21 days	twi_ss.27673.1|g.36873	1.17	6.38	15.57	0.0025	0.0329
21 days	twi_ss.28361.1|g.37927	0.89	6.65	15.58	0.0025	0.0329
21 days	twi_ss.8331.4|g.11453	-1.61	7.60	15.57	0.0025	0.0329
21 days	twi_ss.21289.2|g.27541	0.69	7.49	15.57	0.0025	0.0330
21 days	twi_ss.17571.5|g.22467	7.95	4.66	15.56	0.0026	0.0330

21 days	twi_ss.21079.1|g.27264	-2.08	3.99	15.56	0.0026	0.0330
21 days	twi_ss.30590.6|g.41396	6.48	3.05	17.94	0.0026	0.0330
21 days	twi_ss.26922.1|g.35773	1.14	6.95	15.55	0.0026	0.0330
21 days	twi_ss.11549.1|g.15354	1.05	5.74	15.55	0.0026	0.0330
21 days	twi_ss.27678.1|g.36848	-2.46	4.06	15.55	0.0026	0.0330
21 days	twi_ss.21301.1|g.27540	-1.48	4.58	15.54	0.0026	0.0331
21 days	twi_ss.19879.2|g.25605	-5.64	6.58	15.52	0.0026	0.0331
21 days	twi_ss.31873.1|g.43483	-0.86	5.96	15.53	0.0026	0.0331
21 days	twi_ss.31164.1|g.42386	1.36	5.51	15.52	0.0026	0.0332
21 days	twi_ss.29328.1|g.39445	-2.91	3.97	15.51	0.0026	0.0332
21 days	twi_ss.9768.1|g.13110	-2.54	5.68	15.51	0.0026	0.0332
21 days	twi_ss.20386.1|g.26193	1.14	5.34	15.49	0.0026	0.0333
21 days	twi_ss.23636.4|g.30421	4.95	4.19	15.49	0.0026	0.0333
21 days	twi_ss.31790a.4|g.43290	-6.37	3.19	17.86	0.0026	0.0333
21 days	twi_ss.21566.1|g.27966	-1.52	5.35	15.48	0.0026	0.0333
21 days	twi_ss.28792.1|g.38574	-2.00	4.43	15.48	0.0026	0.0333
21 days	twi_ss.727.1|g.1267	-1.46	4.86	15.48	0.0026	0.0334
21 days	twi_ss.14818.1|g.19280	-1.95	5.62	15.47	0.0026	0.0334
21 days	twi_ss.25500a.3|g.33569	-2.25	4.53	15.45	0.0026	0.0335
21 days	twi_ss.23109.2|g.29748	-6.24	3.11	15.45	0.0026	0.0335
21 days	twi_ss.11893b.2|g.15771	-2.21	4.72	15.44	0.0026	0.0336
21 days	twi_ss.1416.1|g.2651	1.02	4.84	15.42	0.0026	0.0337
21 days	twi_ss.24392.1|g.31991	1.29	7.98	15.42	0.0026	0.0337
21 days	twi_ss.11444.1|g.15092	2.96	5.71	15.41	0.0026	0.0337
21 days	twi_ss.30655.1|g.41500	-4.69	3.18	15.41	0.0026	0.0337
21 days	twi_ss.25707.1|g.33916	-0.83	6.19	15.40	0.0026	0.0338
21 days	twi_ss.4501.1|g.7007	1.06	5.26	15.40	0.0026	0.0338
21 days	twi_ss.30194.1|g.40782	-1.72	5.19	15.40	0.0026	0.0338
21 days	twi_ss.6235.1|g.9210	-2.59	4.50	15.39	0.0026	0.0338
21 days	twi_ss.28270a.6|g.37797	-4.32	1.99	22.58	0.0026	0.0338
21 days	twi_ss.29624.1|g.39910	-2.58	5.87	15.38	0.0027	0.0339
21 days	twi_ss.2495.1|g.4501	-1.81	3.99	15.37	0.0027	0.0339
21 days	twi_ss.30848.1|g.41916	-1.22	4.87	15.36	0.0027	0.0339

21 days	twi_ss.23187.1|g.29844	-1.00	6.23	15.36	0.0027	0.0340
21 days	twi_ss.21665.3|g.27902	6.77	4.00	15.35	0.0027	0.0340
21 days	twi_ss.26274.1|g.34778	-2.83	4.35	15.35	0.0027	0.0340
21 days	twi_ss.18462.1|g.23515	1.66	4.17	15.33	0.0027	0.0341
21 days	twi_ss.10090.1|g.13392	-4.37	3.83	15.32	0.0027	0.0341
21 days	twi_ss.15325.1|g.19889	-2.73	4.46	15.32	0.0027	0.0341
21 days	twi_ss.11378.1|g.14977	-3.16	6.04	15.32	0.0027	0.0342
21 days	twi_ss.21645.2|g.28051	-3.15	3.53	15.31	0.0027	0.0342
21 days	twi_ss.23067.1|g.29709	-1.50	4.80	15.30	0.0027	0.0342
21 days	twi_ss.19965.1|g.25712	-1.38	4.98	15.29	0.0027	0.0343
21 days	twi_ss.26155.1|g.34631	1.27	5.12	15.29	0.0027	0.0343
21 days	twi_ss.330.1|g.606	1.52	4.18	15.29	0.0027	0.0343
21 days	twi_ss.4865.5|g.7448	5.94	2.76	15.27	0.0027	0.0344
21 days	twi_ss.2374b.1|g.4228	1.07	5.66	15.27	0.0027	0.0344
21 days	twi_ss.16847.1|g.21490	-2.90	3.62	15.26	0.0027	0.0344
21 days	twi_ss.22663.1|g.29264	-1.58	4.76	15.25	0.0027	0.0345
21 days	twi_ss.1175.1|g.2087	-6.67	3.25	17.54	0.0027	0.0346
21 days	twi_ss.31179.1|g.42417	-2.24	4.38	15.24	0.0027	0.0346
21 days	twi_ss.15848.1|g.20402	-1.73	3.90	15.23	0.0027	0.0346
21 days	twi_ss.29868.1|g.40338	-5.02	3.81	17.53	0.0027	0.0346
21 days	twi_ss.30708.1|g.41619	1.35	4.36	15.23	0.0027	0.0346
21 days	twi_ss.6134.2|g.9005	0.98	5.85	15.22	0.0027	0.0347
21 days	twi_ss.855.1|g.1417	1.29	4.47	15.21	0.0027	0.0347
21 days	twi_ss.21114a.5|g.27293	-7.54	3.83	15.21	0.0027	0.0347
21 days	twi_ss.9103.1|g.12286	-1.50	4.88	15.17	0.0028	0.0350
21 days	twi_ss.19505.1|g.25044	-4.86	2.30	22.17	0.0028	0.0350
21 days	twi_ss.15787.1|g.20355	1.26	6.60	15.15	0.0028	0.0351
21 days	twi_ss.31045.7|g.42169	-1.06	5.80	15.15	0.0028	0.0351
21 days	twi_ss.12271.2|g.16355	-4.20	4.58	15.15	0.0028	0.0351
21 days	twi_ss.16289.1|g.20877	5.34	2.39	17.42	0.0028	0.0351
21 days	twi_ss.25131.1|g.33044	1.14	6.56	15.14	0.0028	0.0351
21 days	twi_ss.29124.1|g.39039	2.85	3.32	15.14	0.0028	0.0351
21 days	twi_ss.30856.1|g.41914	-1.16	6.19	15.14	0.0028	0.0351

21 days	twi_ss.14153.1|g.18446	-3.16	3.30	15.11	0.0028	0.0353
21 days	twi_ss.4260.1|g.6701	3.73	3.93	15.11	0.0028	0.0353
21 days	twi_ss.21430.1|g.27694	0.86	5.75	15.10	0.0028	0.0353
21 days	twi_ss.29566.2|g.39762	2.17	6.66	15.10	0.0028	0.0353
21 days	twi_ss.9050.1|g.12231	-3.71	4.10	15.10	0.0028	0.0353
21 days	twi_ss.12434.1|g.16558	1.07	5.36	15.09	0.0028	0.0354
21 days	twi_ss.11898.1|g.15785	-2.96	3.79	15.09	0.0028	0.0354
21 days	twi_ss.29746.1|g.40137	-4.60	2.96	15.08	0.0028	0.0354
21 days	twi_ss.7904.1|g.10967	1.36	5.18	15.08	0.0028	0.0354
21 days	twi_ss.20992.1|g.27093	-3.08	3.01	15.07	0.0028	0.0355
21 days	twi_ss.25139a.2|g.33022	1.07	6.17	15.07	0.0028	0.0355
21 days	twi_ss.4270.1|g.6705	-2.34	4.14	15.07	0.0028	0.0355
21 days	twi_ss.5268.1|g.7889	-1.24	4.92	15.07	0.0028	0.0355
21 days	twi_ss.8193.1|g.11281	0.75	5.72	15.07	0.0028	0.0355
21 days	twi_ss.2798.1|g.5037	0.97	5.03	15.06	0.0028	0.0355
21 days	twi_ss.2798.2|g.5032	0.97	5.03	15.06	0.0028	0.0355
21 days	twi_ss.31584b.2|g.43036	0.72	6.71	15.05	0.0028	0.0356
21 days	twi_ss.9482a.1|g.12749	-3.88	3.10	15.04	0.0028	0.0356
21 days	twi_ss.10323.1|g.13907	0.92	6.39	15.04	0.0029	0.0356
21 days	twi_ss.14610.1|g.19082	-1.44	5.14	15.03	0.0029	0.0356
21 days	twi_ss.1960.1|g.3429	1.14	4.81	15.03	0.0029	0.0356
21 days	twi_ss.19645.1|g.25220	-3.02	3.84	15.03	0.0029	0.0356
21 days	twi_ss.24662.1|g.32365	0.53	8.22	15.03	0.0029	0.0356
21 days	twi_ss.6252.3|g.9287	0.80	6.97	15.02	0.0029	0.0357
21 days	twi_ss.2346.1|g.4164	1.28	5.51	15.02	0.0029	0.0357
21 days	twi_ss.2412.1|g.4290	0.74	6.36	15.01	0.0029	0.0357
21 days	twi_ss.15596.1|g.20167	0.63	6.38	15.01	0.0029	0.0357
21 days	twi_ss.15781.1|g.20345	1.53	5.60	14.99	0.0029	0.0357
21 days	twi_ss.15986.1|g.20602	-2.27	5.94	15.01	0.0029	0.0357
21 days	twi_ss.15988.1|g.20594	-1.84	4.62	15.01	0.0029	0.0357
21 days	twi_ss.17427.1|g.22241	-1.52	4.27	14.99	0.0029	0.0357
21 days	twi_ss.18486.1|g.23548	-3.27	3.92	15.00	0.0029	0.0357
21 days	twi_ss.21663.2|g.27929	6.59	4.05	17.23	0.0029	0.0357

21 days	twi_ss.28268.1|g.37789	-2.56	5.24	15.00	0.0029	0.0357
21 days	twi_ss.29168.2|g.39066	0.87	6.71	14.99	0.0029	0.0357
21 days	twi_ss.29329.1|g.39338	-2.56	4.08	15.00	0.0029	0.0357
21 days	twi_ss.6778a.3|g.9735	1.16	4.99	15.00	0.0029	0.0357
21 days	twi_ss.21035.1|g.27197	1.15	4.69	14.99	0.0029	0.0357
21 days	twi_ss.13656.1|g.18009	-3.56	3.52	14.98	0.0029	0.0358
21 days	twi_ss.13349.2|g.17697	-1.92	4.66	14.97	0.0029	0.0358
21 days	twi_ss.2409a.1|g.4153	0.53	8.17	14.97	0.0029	0.0358
21 days	twi_ss.3154.1|g.5546	1.30	6.80	14.97	0.0029	0.0358
21 days	twi_ss.7494.4|g.10534	6.03	3.06	14.96	0.0029	0.0359
21 days	twi_ss.4294.1|g.6780	0.68	7.53	14.95	0.0029	0.0359
21 days	twi_ss.20572.1|g.26615	0.61	6.39	14.94	0.0029	0.0360
21 days	twi_ss.14366.1|g.18716	-3.24	3.91	14.93	0.0029	0.0360
21 days	twi_ss.1583.1|g.2827	1.05	5.46	14.93	0.0029	0.0360
21 days	twi_ss.28031b.1|g.37305	-0.87	7.52	14.94	0.0029	0.0360
21 days	twi_ss.2827b.3|g.5071	1.46	5.48	14.93	0.0029	0.0360
21 days	twi_ss.5604.3|g.8404	-6.28	4.27	14.91	0.0029	0.0361
21 days	twi_ss.6388.2|g.9411	-0.81	6.78	14.90	0.0029	0.0362
21 days	twi_ss.11596.1|g.15229	-3.58	3.62	14.89	0.0029	0.0362
21 days	twi_ss.21287.1|g.27512	1.00	6.01	14.89	0.0029	0.0362
21 days	twi_ss.30803a.1|g.41752	2.08	6.26	14.89	0.0029	0.0362
21 days	twi_ss.946.8|g.1700	5.96	2.46	17.10	0.0029	0.0362
21 days	twi_ss.31958.1|g.43542	1.38	4.51	14.89	0.0029	0.0362
21 days	twi_ss.19447.1|g.24993	0.76	6.32	14.88	0.0029	0.0362
21 days	twi_ss.2112b.1|g.3797	-1.00	5.68	14.88	0.0029	0.0362
21 days	twi_ss.11534.7|g.15250	6.48	2.74	35.26	0.0030	0.0363
21 days	twi_ss.24489.1|g.32068	-4.42	3.14	14.86	0.0030	0.0364
21 days	twi_ss.28345a.1|g.37870	-3.07	3.62	14.83	0.0030	0.0366
21 days	twi_ss.29769.1|g.40156	1.30	4.40	14.83	0.0030	0.0366
21 days	twi_ss.30556.2|g.41340	-2.78	3.93	14.83	0.0030	0.0366
21 days	twi_ss.30665.1|g.41512	-1.34	5.11	14.83	0.0030	0.0366
21 days	twi_ss.3849.2|g.6272	0.97	6.82	14.83	0.0030	0.0366
21 days	twi_ss.18721.1|g.23894	2.36	4.14	14.81	0.0030	0.0367

21 days	twi_ss.1428.1|g.2644	-3.38	3.15	14.81	0.0030	0.0367
21 days	twi_ss.29366b.6|g.39462	-6.14	3.96	14.80	0.0030	0.0367
21 days	twi_ss.13443.1|g.17730	1.03	5.26	14.79	0.0030	0.0368
21 days	twi_ss.31331.1|g.42606	-4.44	3.86	14.79	0.0030	0.0368
21 days	twi_ss.9437.1|g.12744	-4.05	3.92	14.78	0.0030	0.0368
21 days	twi_ss.29142.1|g.39100	-2.68	4.50	14.78	0.0030	0.0369
21 days	twi_ss.29504.8|g.39731	3.11	4.04	14.77	0.0030	0.0369
21 days	twi_ss.18881a.2|g.23976	-3.70	5.02	14.77	0.0030	0.0369
21 days	twi_ss.5146.1|g.7786	-1.89	4.07	14.76	0.0030	0.0370
21 days	twi_ss.30135.1|g.40626	0.77	6.23	14.75	0.0030	0.0370
21 days	twi_ss.16558a.2|g.21136	0.52	9.85	14.75	0.0030	0.0370
21 days	twi_ss.25853.1|g.34153	-2.82	4.57	14.74	0.0030	0.0371
21 days	twi_ss.17775.1|g.22682	-5.67	2.50	16.87	0.0031	0.0374
21 days	twi_ss.1571.1|g.2733	-1.08	6.10	14.69	0.0031	0.0375
21 days	twi_ss.2827a.1|g.5073	-1.13	5.49	14.69	0.0031	0.0375
21 days	twi_ss.23783.1|g.30811	-3.40	3.49	14.68	0.0031	0.0375
21 days	twi_ss.6144a.3|g.9157	1.64	3.84	14.66	0.0031	0.0376
21 days	twi_ss.28573.1|g.38252	1.02	6.36	14.66	0.0031	0.0377
21 days	twi_ss.4371.1|g.6877	4.03	2.18	19.15	0.0031	0.0377
21 days	twi_ss.2729.1|g.4936	-0.77	6.41	14.64	0.0031	0.0377
21 days	twi_ss.2729.2|g.4934	-0.77	6.41	14.64	0.0031	0.0377
21 days	twi_ss.4661.2|g.7185	0.68	6.38	14.64	0.0031	0.0377
21 days	twi_ss.25007.2|g.32832	-4.38	1.96	14.63	0.0031	0.0378
21 days	twi_ss.14557.1|g.18980	0.80	6.51	14.63	0.0031	0.0378
21 days	twi_ss.9257.1|g.12473	-1.31	5.70	14.62	0.0031	0.0379
21 days	twi_ss.17504.1|g.22273	0.70	7.19	14.61	0.0031	0.0379
21 days	twi_ss.24683.1|g.32384	-1.95	3.73	14.61	0.0031	0.0379
21 days	twi_ss.24683.2|g.32385	-1.95	3.73	14.61	0.0031	0.0379
21 days	twi_ss.799a.3|g.1393	3.41	5.83	14.61	0.0031	0.0379
21 days	twi_ss.3095.1|g.5484	-4.47	4.38	14.61	0.0031	0.0379
21 days	twi_ss.13379.1|g.17690	-2.08	4.15	14.59	0.0031	0.0380
21 days	twi_ss.4828.1|g.7416	3.05	4.40	14.59	0.0031	0.0380
21 days	twi_ss.11616.1|g.15418	-0.85	6.39	14.59	0.0031	0.0380

21 days	twi_ss.15555.1|g.20126	-2.06	3.49	14.59	0.0031	0.0380
21 days	twi_ss.23697.1|g.30680	-2.53	3.53	14.59	0.0031	0.0380
21 days	twi_ss.11488b.2|g.15166	1.32	5.52	14.58	0.0032	0.0381
21 days	twi_ss.13032.1|g.17262	-1.01	5.69	14.57	0.0032	0.0381
21 days	twi_ss.4355.1|g.6871	1.49	5.77	14.57	0.0032	0.0381
21 days	twi_ss.20145.1|g.25895	-1.52	5.64	14.56	0.0032	0.0381
21 days	twi_ss.17277a.13|g.22019	5.00	2.56	16.68	0.0032	0.0382
21 days	twi_ss.2920.1|g.5215	-3.68	3.56	14.55	0.0032	0.0382
21 days	twi_ss.4425.1|g.6924	1.85	4.35	14.55	0.0032	0.0382
21 days	twi_ss.8507.1|g.11674	2.44	4.21	14.55	0.0032	0.0382
21 days	twi_ss.27623.2|g.36632	-0.61	7.22	14.55	0.0032	0.0382
21 days	twi_ss.3336.1|g.5735	-7.57	3.56	34.02	0.0032	0.0382
21 days	twi_ss.12114.1|g.16084	0.87	7.05	14.53	0.0032	0.0383
21 days	twi_ss.28156b.3|g.37499	-2.01	6.09	14.52	0.0032	0.0383
21 days	twi_ss.6166a.1|g.9002	1.66	4.28	14.52	0.0032	0.0384
21 days	twi_ss.3933.1|g.6366	-2.16	3.20	14.51	0.0032	0.0384
21 days	twi_ss.12241.1|g.16284	1.80	4.34	14.50	0.0032	0.0384
21 days	twi_ss.2434.1|g.4300	0.59	7.06	14.50	0.0032	0.0385
21 days	twi_ss.14337.1|g.18618	1.03	5.27	14.50	0.0032	0.0385
21 days	twi_ss.2373.3|g.4234	0.93	5.74	14.49	0.0032	0.0385
21 days	twi_ss.15059.1|g.19552	-3.68	3.51	14.47	0.0032	0.0386
21 days	twi_ss.2092.1|g.3745	1.97	3.22	14.47	0.0032	0.0386
21 days	twi_ss.25754.2|g.33965	-5.54	2.66	16.57	0.0032	0.0386
21 days	twi_ss.26685.1|g.35461	-2.74	5.20	14.48	0.0032	0.0386
21 days	twi_ss.22017.1|g.28515	-4.37	2.20	16.56	0.0032	0.0387
21 days	twi_ss.30904.1|g.42100	1.02	6.07	14.44	0.0032	0.0389
21 days	twi_ss.10507.1|g.14141	0.60	6.10	14.42	0.0033	0.0389
21 days	twi_ss.10507.2|g.14143	0.60	6.10	14.42	0.0033	0.0389
21 days	twi_ss.10507.3|g.14140	0.60	6.10	14.42	0.0033	0.0389
21 days	twi_ss.10507.4|g.14139	0.60	6.10	14.42	0.0033	0.0389
21 days	twi_ss.10507.5|g.14142	0.60	6.10	14.42	0.0033	0.0389
21 days	twi_ss.28174.1|g.37582	-1.00	6.64	14.42	0.0033	0.0389
21 days	twi_ss.17936.2|g.22817	-6.83	3.53	14.41	0.0033	0.0390

21 days	twi_ss.21215.1|g.27452	1.02	6.07	14.40	0.0033	0.0390
21 days	twi_ss.10306.10|g.13837	6.49	3.20	14.40	0.0033	0.0390
21 days	twi_ss.23465.1|g.30092	-0.78	5.75	14.40	0.0033	0.0390
21 days	twi_ss.3540.1|g.5916	1.85	4.25	14.40	0.0033	0.0390
21 days	twi_ss.19350.2|g.24861	-1.14	5.47	14.40	0.0033	0.0391
21 days	twi_ss.1420.1|g.2668	-1.38	5.37	14.38	0.0033	0.0392
21 days	twi_ss.11378.2|g.14991	-3.00	7.34	14.37	0.0033	0.0392
21 days	twi_ss.17481b.2|g.22286	0.72	7.48	14.36	0.0033	0.0393
21 days	twi_ss.1696.1|g.3023	-0.86	5.10	14.35	0.0033	0.0394
21 days	twi_ss.11197.1|g.14847	-1.14	5.94	14.34	0.0033	0.0394
21 days	twi_ss.24286.1|g.31737	-6.75	3.06	14.34	0.0033	0.0394
21 days	twi_ss.9224.1|g.12466	-1.17	5.33	14.34	0.0033	0.0394
21 days	twi_ss.7525.1|g.10578	-4.23	2.05	17.14	0.0033	0.0394
21 days	twi_ss.902.1|g.1591	-4.07	3.42	14.33	0.0033	0.0395
21 days	twi_ss.10707b.1|g.14373	0.95	6.48	14.32	0.0033	0.0396
21 days	twi_ss.16630.1|g.21259	-0.78	9.51	14.31	0.0033	0.0396
21 days	twi_ss.25565.1|g.33670	-2.48	4.08	14.31	0.0033	0.0396
21 days	twi_ss.1000.1|g.1831	-2.20	3.98	14.31	0.0033	0.0396
21 days	twi_ss.18886.1|g.24141	1.04	5.26	14.30	0.0034	0.0397
21 days	twi_ss.5112.1|g.7738	1.95	5.43	14.30	0.0034	0.0397
21 days	twi_ss.11610.1|g.15425	0.53	8.85	14.29	0.0034	0.0397
21 days	twi_ss.10160.1|g.13616	-4.13	1.90	15.03	0.0034	0.0397
21 days	twi_ss.17908.1|g.22797	2.35	4.70	14.29	0.0034	0.0397
21 days	twi_ss.16840.1|g.21488	0.73	5.95	14.28	0.0034	0.0398
21 days	twi_ss.3096.1|g.5486	1.99	4.37	14.28	0.0034	0.0398
21 days	twi_ss.11250.1|g.14896	-0.66	7.40	14.26	0.0034	0.0398
21 days	twi_ss.19931.1|g.25709	-1.34	4.60	14.26	0.0034	0.0398
21 days	twi_ss.2665.8|g.4812	5.09	2.10	16.31	0.0034	0.0398
21 days	twi_ss.30834b.4|g.42026	-5.59	3.02	14.27	0.0034	0.0398
21 days	twi_ss.7015.1|g.10082	-2.90	3.75	14.27	0.0034	0.0398
21 days	twi_ss.17038.1|g.21810	1.16	5.69	14.26	0.0034	0.0399
21 days	twi_ss.27229.1|g.36173	1.18	5.88	14.25	0.0034	0.0399
21 days	twi_ss.28087.1|g.37427	-0.83	6.02	14.26	0.0034	0.0399

21 days	twi_ss.3684.1|g.6062	1.37	5.00	14.24	0.0034	0.0400
21 days	twi_ss.16883.1|g.21508	1.22	4.72	14.22	0.0034	0.0401
21 days	twi_ss.21760.1|g.28222	0.68	7.59	14.22	0.0034	0.0401
21 days	twi_ss.23718.1|g.30710	-5.76	2.62	16.26	0.0034	0.0401
21 days	twi_ss.28557.1|g.38278	-1.75	4.71	14.22	0.0034	0.0401
21 days	twi_ss.29717.1|g.40099	-4.01	4.13	14.22	0.0034	0.0401
21 days	twi_ss.3197.4|g.5619	1.34	6.42	14.22	0.0034	0.0401
21 days	twi_ss.3796.3|g.6235	3.66	4.60	14.22	0.0034	0.0401
21 days	twi_ss.7100.1|g.10177	-2.83	5.19	14.22	0.0034	0.0401
21 days	twi_ss.9250.1|g.12472	0.90	5.71	14.22	0.0034	0.0401
21 days	twi_ss.1749.3|g.3079	7.28	3.41	16.25	0.0034	0.0401
21 days	twi_ss.26363.1|g.34804	-0.52	6.52	14.21	0.0034	0.0401
21 days	twi_ss.7665.8|g.10743	-4.44	5.06	14.21	0.0034	0.0401
21 days	twi_ss.18560.1|g.23679	-2.61	5.22	14.20	0.0034	0.0401
21 days	twi_ss.27345a.1|g.36417	-0.53	9.34	14.19	0.0034	0.0402
21 days	twi_ss.30208.1|g.40797	5.41	2.88	16.23	0.0034	0.0402
21 days	twi_ss.22661.1|g.29266	-0.90	6.04	14.19	0.0034	0.0402
21 days	twi_ss.27981.1|g.37260	-3.97	3.08	14.18	0.0034	0.0402
21 days	twi_ss.30486.1|g.41283	1.48	6.06	14.18	0.0034	0.0402
21 days	twi_ss.23535.5|g.30208	4.68	2.51	14.18	0.0034	0.0402
21 days	twi_ss.31953.1|g.43541	1.22	6.10	14.18	0.0034	0.0402
21 days	twi_ss.20311b.6|g.26145	-3.93	3.02	14.14	0.0035	0.0405
21 days	twi_ss.1663.1|g.2901	-3.17	3.42	14.14	0.0035	0.0406
21 days	twi_ss.5449.1|g.8242	0.62	7.42	14.13	0.0035	0.0406
21 days	twi_ss.8624.1|g.11776	-1.31	4.87	14.12	0.0035	0.0406
21 days	twi_ss.26388.1|g.34959	-3.18	3.28	14.11	0.0035	0.0408
21 days	twi_ss.420.1|g.804	-0.91	4.81	14.11	0.0035	0.0408
21 days	twi_ss.4486.1|g.7001	-1.37	4.53	14.10	0.0035	0.0408
21 days	twi_ss.16608.1|g.21270	-1.95	5.01	14.08	0.0035	0.0410
21 days	twi_ss.23086.1|g.29718	-5.44	2.95	16.09	0.0035	0.0410
21 days	twi_ss.5247.1|g.7904	-5.78	2.76	16.08	0.0035	0.0410
21 days	twi_ss.9212.1|g.12409	-2.72	3.81	14.08	0.0035	0.0410
21 days	twi_ss.27452.1|g.36369	0.84	5.88	14.07	0.0035	0.0410

21 days	twi_ss.10319.1|g.13828	0.94	5.88	14.05	0.0035	0.0412
21 days	twi_ss.12970.1|g.17215	0.97	6.09	14.05	0.0035	0.0412
21 days	twi_ss.7858.13|g.10902	1.52	6.11	14.04	0.0035	0.0412
21 days	twi_ss.31044.1|g.42224	-4.36	4.84	14.04	0.0036	0.0413
21 days	twi_ss.18500.1|g.23592	-0.94	7.56	14.02	0.0036	0.0414
21 days	twi_ss.2115.1|g.3816	-3.00	4.98	14.02	0.0036	0.0414
21 days	twi_ss.8139.1|g.11191	-5.49	2.67	16.00	0.0036	0.0414
21 days	twi_ss.6132.1|g.8997	-3.31	3.48	14.00	0.0036	0.0415
21 days	twi_ss.25718.1|g.33921	-3.38	3.90	14.00	0.0036	0.0415
21 days	twi_ss.10199.1|g.13714	1.43	4.62	13.99	0.0036	0.0416
21 days	twi_ss.7013.1|g.10084	-6.71	3.05	15.96	0.0036	0.0417
21 days	twi_ss.4508.1|g.7016	-0.55	9.11	13.98	0.0036	0.0417
21 days	twi_ss.28712.2|g.38478	4.27	1.87	15.95	0.0036	0.0417
21 days	twi_ss.28412.1|g.37988	-2.20	4.17	13.97	0.0036	0.0417
21 days	twi_ss.25088.1|g.32922	-1.69	4.80	13.97	0.0036	0.0417
21 days	twi_ss.16904.1|g.21542	1.00	4.92	13.95	0.0036	0.0418
21 days	twi_ss.25245.1|g.33240	-2.79	4.98	13.95	0.0036	0.0418
21 days	twi_ss.28977.1|g.38899	4.72	2.60	13.95	0.0036	0.0418
21 days	twi_ss.31790a.6|g.43289	-4.33	2.41	15.93	0.0036	0.0418
21 days	twi_ss.3627.1|g.5997	2.64	3.88	13.95	0.0036	0.0418
21 days	twi_ss.26812.1|g.35547	-1.01	6.03	13.95	0.0036	0.0418
21 days	twi_ss.5400.1|g.8145	-1.73	5.66	13.94	0.0036	0.0419
21 days	twi_ss.2415.2|g.4288	0.53	7.75	13.94	0.0036	0.0419
21 days	twi_ss.27269.2|g.36254	6.72	3.98	13.93	0.0036	0.0419
21 days	twi_ss.28111.1|g.37440	-5.53	2.45	15.90	0.0036	0.0419
21 days	twi_ss.30592.1|g.41428	-1.65	4.65	13.93	0.0036	0.0419
21 days	twi_ss.25519.1|g.33528	1.10	4.96	13.91	0.0037	0.0421
21 days	twi_ss.29486.2|g.39693	-1.74	4.38	13.91	0.0037	0.0421
21 days	twi_ss.26888.1|g.35668	-1.46	5.51	13.90	0.0037	0.0422
21 days	twi_ss.13480.1|g.17843	-1.74	4.35	13.89	0.0037	0.0423
21 days	twi_ss.5607.1|g.8346	1.82	3.75	13.89	0.0037	0.0423
21 days	twi_ss.17350.1|g.22149	0.40	8.64	13.88	0.0037	0.0423
21 days	twi_ss.22694.1|g.29310	-0.95	5.93	13.87	0.0037	0.0423

21 days	twi_ss.11522.1|g.15189	2.05	3.18	13.87	0.0037	0.0424
21 days	twi_ss.29937.5|g.40383	-4.46	2.14	19.81	0.0037	0.0424
21 days	twi_ss.24144.1|g.31407	-5.32	2.74	15.82	0.0037	0.0424
21 days	twi_ss.23836a.2|g.30870	-0.80	7.43	13.86	0.0037	0.0424
21 days	twi_ss.31344.3|g.42641	-0.95	5.18	13.85	0.0037	0.0424
21 days	twi_ss.24783.1|g.32483	-3.41	2.93	13.85	0.0037	0.0424
21 days	twi_ss.17103.1|g.21882	-4.13	3.53	13.84	0.0037	0.0426
21 days	twi_ss.8252.1|g.11322	-3.08	3.70	13.84	0.0037	0.0426
21 days	twi_ss.5444.1|g.8241	-4.43	3.20	13.83	0.0037	0.0426
21 days	twi_ss.31032.3|g.42136	5.55	2.58	15.76	0.0037	0.0427
21 days	twi_ss.27661.1|g.36870	-3.09	3.70	13.81	0.0037	0.0428
21 days	twi_ss.28446.1|g.38057	-0.89	7.08	13.81	0.0037	0.0428
21 days	twi_ss.3576.3|g.5947	-5.48	2.81	13.81	0.0037	0.0428
21 days	twi_ss.17967a.3|g.22837	-1.86	4.92	13.80	0.0037	0.0428
21 days	twi_ss.5304a.2|g.8063	1.46	4.22	13.80	0.0037	0.0428
21 days	twi_ss.4572.1|g.7092	-1.76	6.31	13.80	0.0037	0.0428
21 days	twi_ss.12990.4|g.17199	-6.70	3.58	15.73	0.0038	0.0428
21 days	twi_ss.20557.7|g.26555	-2.85	5.79	13.80	0.0038	0.0428
21 days	twi_ss.21665.1|g.27904	2.74	6.25	13.79	0.0038	0.0428
21 days	twi_ss.23748.1|g.30645	-4.82	3.30	13.79	0.0038	0.0428
21 days	twi_ss.30630.1|g.41472	-0.54	6.70	13.78	0.0038	0.0429
21 days	twi_ss.9083.1|g.12240	1.28	4.24	13.78	0.0038	0.0429
21 days	twi_ss.26468.1|g.35099	0.78	5.46	13.77	0.0038	0.0429
21 days	twi_ss.1574.1|g.2757	0.74	6.68	13.75	0.0038	0.0432
21 days	twi_ss.28280.1|g.37812	0.55	8.58	13.74	0.0038	0.0432
21 days	twi_ss.10321.1|g.13814	0.73	6.05	13.74	0.0038	0.0432
21 days	twi_ss.9923.1|g.13253	0.51	7.65	13.73	0.0038	0.0433
21 days	twi_ss.29011.1|g.38931	-1.45	5.02	13.73	0.0038	0.0433
21 days	twi_ss.6948.1|g.10020	-3.62	3.72	13.73	0.0038	0.0433
21 days	twi_ss.8703.1|g.11841	1.55	4.90	13.72	0.0038	0.0433
21 days	twi_ss.31909.2|g.43516	4.08	6.09	13.72	0.0038	0.0434
21 days	twi_ss.2139.1|g.3798	-2.79	3.85	13.71	0.0038	0.0434
21 days	twi_ss.2387a.1|g.4247	-3.22	3.26	13.70	0.0038	0.0435

21 days	twi_ss.9747.1|g.13086	0.52	6.43	13.69	0.0038	0.0435
21 days	twi_ss.5283.3|g.8002	7.31	4.69	13.68	0.0039	0.0437
21 days	twi_ss.28507.1|g.38036	-4.95	2.59	15.58	0.0039	0.0437
21 days	twi_ss.32054.1|g.43704	-0.69	6.34	13.66	0.0039	0.0438
21 days	twi_ss.3373.2|g.5768	-4.52	2.01	13.66	0.0039	0.0438
21 days	twi_ss.12712.1|g.16771	1.01	5.52	13.65	0.0039	0.0439
21 days	twi_ss.14216.1|g.18528	-7.29	3.70	13.64	0.0039	0.0440
21 days	twi_ss.18778.1|g.23944	-1.23	5.48	13.62	0.0039	0.0440
21 days	twi_ss.22954b.1|g.29602	0.67	6.47	13.63	0.0039	0.0440
21 days	twi_ss.24806.1|g.32569	-0.74	5.57	13.63	0.0039	0.0440
21 days	twi_ss.29730.1|g.40162	0.96	6.29	13.62	0.0039	0.0440
21 days	twi_ss.3095.2|g.5485	-3.03	3.83	13.63	0.0039	0.0440
21 days	twi_ss.6228.2|g.9233	-4.60	2.01	13.63	0.0039	0.0440
21 days	twi_ss.15904.2|g.20518	-6.54	3.75	14.42	0.0039	0.0441
21 days	twi_ss.2227.1|g.4003	-0.99	5.40	13.61	0.0039	0.0442
21 days	twi_ss.6046.1|g.8890	1.32	6.14	13.61	0.0039	0.0442
21 days	twi_ss.11020.1|g.14643	-3.68	2.79	13.60	0.0039	0.0442
21 days	twi_ss.20792.1|g.26912	1.11	4.76	13.59	0.0039	0.0443
21 days	twi_ss.20900.2|g.27016	-7.75	4.31	13.59	0.0039	0.0443
21 days	twi_ss.26497.1|g.35119	0.54	8.42	13.59	0.0039	0.0443
21 days	twi_ss.4596.1|g.7149	0.81	7.84	13.58	0.0039	0.0444
21 days	twi_ss.16895.1|g.21531	1.33	6.26	13.57	0.0039	0.0444
21 days	twi_ss.9787.1|g.13143	-3.08	3.23	13.56	0.0040	0.0445
21 days	twi_ss.30318.1|g.41001	-5.80	2.82	15.43	0.0040	0.0445
21 days	twi_ss.24322.1|g.31835	0.83	4.93	14.17	0.0040	0.0446
21 days	twi_ss.8950.1|g.12141	-2.78	4.17	13.54	0.0040	0.0447
21 days	twi_ss.27674.1|g.36836	-0.71	5.99	13.53	0.0040	0.0447
21 days	twi_ss.11338.1|g.14959	-4.73	2.78	15.40	0.0040	0.0447
21 days	twi_ss.4540.1|g.7056	-5.11	2.25	19.21	0.0040	0.0447
21 days	twi_ss.17709.1|g.22580	-3.52	3.12	13.53	0.0040	0.0448
21 days	twi_ss.31562.1|g.42975	0.71	5.52	13.52	0.0040	0.0448
21 days	twi_ss.24352c.4|g.31774	0.95	7.43	13.52	0.0040	0.0448
21 days	twi_ss.31752.1|g.43239	-3.15	3.91	13.52	0.0040	0.0448

21 days	twi_ss.7858.17|g.10906	1.65	5.47	13.51	0.0040	0.0448
21 days	twi_ss.28579.4|g.38269	1.12	7.55	13.50	0.0040	0.0449
21 days	twi_ss.969.1|g.1738	-1.62	3.90	13.50	0.0040	0.0449
21 days	twi_ss.14331.1|g.18637	-2.96	4.93	13.49	0.0040	0.0449
21 days	twi_ss.19570.12|g.25102	-4.29	4.41	13.49	0.0040	0.0449
21 days	twi_ss.21399.2|g.27678	-5.44	2.64	15.36	0.0040	0.0449
21 days	twi_ss.25545.1|g.33590	1.60	4.60	13.50	0.0040	0.0449
21 days	twi_ss.27000.1|g.35874	1.44	6.94	13.48	0.0040	0.0450
21 days	twi_ss.14756.4|g.19195	1.99	4.93	13.48	0.0040	0.0450
21 days	twi_ss.18300a.1|g.23261	-5.92	3.20	13.48	0.0040	0.0450
21 days	twi_ss.25977.1|g.34277	5.73	2.77	13.47	0.0040	0.0450
21 days	twi_ss.29828.1|g.40233	-1.59	4.10	13.48	0.0040	0.0450
21 days	twi_ss.31494.4|g.42901	-4.69	2.05	13.48	0.0040	0.0450
21 days	twi_ss.22431.1|g.28977	-2.10	3.16	13.47	0.0040	0.0451
21 days	twi_ss.17952.1|g.22827	0.98	5.38	13.46	0.0040	0.0451
21 days	twi_ss.31108.5|g.42313	5.11	2.10	15.32	0.0040	0.0451
21 days	twi_ss.13116a.2|g.17395	0.95	5.89	13.45	0.0041	0.0452
21 days	twi_ss.21964.1|g.28464	2.42	4.38	13.44	0.0041	0.0453
21 days	twi_ss.22411.1|g.28955	1.41	4.24	13.44	0.0041	0.0453
21 days	twi_ss.7276.1|g.10305	-0.81	5.47	13.44	0.0041	0.0453
21 days	twi_ss.18361.2|g.23429	-4.89	2.49	13.43	0.0041	0.0453
21 days	twi_ss.16232.2|g.20802	6.20	3.53	13.43	0.0041	0.0453
21 days	twi_ss.18881a.24|g.24022	-4.04	3.60	13.43	0.0041	0.0453
21 days	twi_ss.20935.1|g.27048	-1.66	5.89	13.43	0.0041	0.0453
21 days	twi_ss.13261.1|g.17623	1.06	4.42	13.41	0.0041	0.0454
21 days	twi_ss.23593.1|g.30335	1.50	4.94	13.41	0.0041	0.0454
21 days	twi_ss.2953.1|g.5269	-6.20	2.71	19.00	0.0041	0.0454
21 days	twi_ss.28981.7|g.38890	4.28	1.95	13.40	0.0041	0.0455
21 days	twi_ss.23711.13|g.30557	-7.31	3.94	13.40	0.0041	0.0456
21 days	twi_ss.31563a.1|g.42998	1.19	4.69	13.39	0.0041	0.0457
21 days	twi_ss.3638a.1|g.6014	-2.17	4.28	13.39	0.0041	0.0457
21 days	twi_ss.11249.1|g.14908	1.12	4.36	15.70	0.0041	0.0457
21 days	twi_ss.20264a.1|g.26041	0.98	5.48	13.38	0.0041	0.0457

21 days	twi_ss.23409.1|g.30076	-1.25	5.76	13.37	0.0041	0.0458
21 days	twi_ss.9116.1|g.12305	-1.77	4.11	13.36	0.0041	0.0458
21 days	twi_ss.686.1|g.1225	-1.61	4.34	13.35	0.0042	0.0459
21 days	twi_ss.2403a.1|g.4147	0.93	7.15	13.35	0.0042	0.0459
21 days	twi_ss.4929.1|g.7529	0.83	6.55	13.34	0.0042	0.0460
21 days	twi_ss.1038.2|g.1886	-0.98	6.20	13.33	0.0042	0.0461
21 days	twi_ss.10693.1|g.14355	-3.02	3.60	13.33	0.0042	0.0461
21 days	twi_ss.2301.1|g.4043	0.99	6.49	13.33	0.0042	0.0461
21 days	twi_ss.23183.1|g.29846	0.69	6.26	13.32	0.0042	0.0462
21 days	twi_ss.30928.1|g.41907	-7.92	4.92	13.32	0.0042	0.0462
21 days	twi_ss.31278.5|g.42559	-6.76	3.05	13.32	0.0042	0.0462
21 days	twi_ss.8099.7|g.11141	-4.31	4.68	13.32	0.0042	0.0462
21 days	twi_ss.16186.1|g.20766	-4.46	3.04	13.31	0.0042	0.0462
21 days	twi_ss.10837.5|g.14461	2.07	5.41	13.31	0.0042	0.0462
21 days	twi_ss.31878a.2|g.43481	-2.90	3.54	13.31	0.0042	0.0462
21 days	twi_ss.1669.1|g.2983	1.13	4.89	13.30	0.0042	0.0462
21 days	twi_ss.28344.1|g.37843	-1.60	4.20	13.30	0.0042	0.0462
21 days	twi_ss.2342.1|g.4244	-1.93	3.99	13.30	0.0042	0.0463
21 days	twi_ss.19213.1|g.24609	0.78	6.18	13.28	0.0042	0.0464
21 days	twi_ss.498.1|g.892	-0.56	8.16	13.28	0.0042	0.0464
21 days	twi_ss.29671.1|g.39997	-3.57	2.94	13.28	0.0042	0.0464
21 days	twi_ss.3735.1|g.6113	-1.23	5.71	13.27	0.0042	0.0465
21 days	twi_ss.17442.1|g.22250	0.76	7.69	13.26	0.0042	0.0466
21 days	twi_ss.1805.1|g.3179	0.82	5.55	13.25	0.0043	0.0467
21 days	twi_ss.3520.1|g.5891	1.36	5.16	13.25	0.0043	0.0467
21 days	twi_ss.26140b.3|g.34607	0.62	8.94	13.25	0.0043	0.0467
21 days	twi_ss.16562.1|g.21165	0.94	6.05	13.24	0.0043	0.0467
21 days	twi_ss.24837.2|g.32675	-1.51	6.19	13.24	0.0043	0.0467
21 days	twi_ss.27913.1|g.37197	-1.44	4.71	13.24	0.0043	0.0467
21 days	twi_ss.20048.1|g.25829	1.05	5.61	13.23	0.0043	0.0467
21 days	twi_ss.20599.2|g.26613	-1.01	5.40	13.23	0.0043	0.0467
21 days	twi_ss.20647.1|g.26637	-1.35	4.80	13.22	0.0043	0.0468
21 days	twi_ss.10430.1|g.14064	-1.26	4.44	13.22	0.0043	0.0469

21 days	twi_ss.26238b.2|g.34744	1.45	4.71	13.22	0.0043	0.0469
21 days	twi_ss.3906.1|g.6319	1.17	4.55	13.22	0.0043	0.0469
21 days	twi_ss.12035.1|g.15933	0.71	5.37	13.19	0.0043	0.0471
21 days	twi_ss.4282.1|g.6722	-1.66	4.61	13.16	0.0043	0.0475
21 days	twi_ss.12473.1|g.16571	0.65	9.58	13.15	0.0044	0.0475
21 days	twi_ss.21314.1|g.27582	1.02	5.25	13.15	0.0044	0.0475
21 days	twi_ss.25243.1|g.33160	4.48	2.06	13.14	0.0044	0.0476
21 days	twi_ss.28712.1|g.38476	-4.03	3.71	13.13	0.0044	0.0477
21 days	twi_ss.27964.1|g.37238	1.11	6.59	13.13	0.0044	0.0477
21 days	twi_ss.12698.1|g.16804	0.97	4.80	13.11	0.0044	0.0479
21 days	twi_ss.27993.1|g.37281	1.08	5.08	13.11	0.0044	0.0479
21 days	twi_ss.31626.1|g.43069	-1.61	7.10	13.11	0.0044	0.0479
21 days	twi_ss.19384.1|g.24874	7.36	6.19	14.87	0.0044	0.0479
21 days	twi_ss.27089.1|g.35962	-1.94	5.21	13.10	0.0044	0.0479
21 days	twi_ss.28018.1|g.37302	0.64	9.52	13.10	0.0044	0.0479
21 days	twi_ss.2917.1|g.5212	-1.14	5.84	13.10	0.0044	0.0479
21 days	twi_ss.2166.6|g.3829	5.75	2.59	14.86	0.0044	0.0479
21 days	twi_ss.19898.1|g.25626	1.42	3.89	13.09	0.0044	0.0480
21 days	twi_ss.9232b.3|g.12510	-5.12	2.33	14.84	0.0044	0.0481
21 days	twi_ss.14132.1|g.18428	-1.68	4.21	13.07	0.0044	0.0481
21 days	twi_ss.7757.3|g.10826	3.33	7.05	13.06	0.0044	0.0482
21 days	twi_ss.18453.1|g.23507	0.83	6.97	13.06	0.0044	0.0483
21 days	twi_ss.26716.1|g.35367	0.56	7.78	13.05	0.0045	0.0483
21 days	twi_ss.25463b.1|g.33527	0.70	6.24	13.05	0.0045	0.0484
21 days	twi_ss.21051.1|g.27249	2.90	3.47	13.04	0.0045	0.0484
21 days	twi_ss.31419.1|g.42791	1.54	4.02	13.04	0.0045	0.0484
21 days	twi_ss.9735b.8|g.13056	-5.45	2.32	28.60	0.0045	0.0484
21 days	twi_ss.20004.1|g.25770	7.92	3.75	28.57	0.0045	0.0485
21 days	twi_ss.24380.1|g.31949	-1.57	4.57	13.02	0.0045	0.0486
21 days	twi_ss.4477.3|g.6995	7.23	3.97	14.75	0.0045	0.0487
21 days	twi_ss.31753.1|g.43255	-3.96	5.26	13.00	0.0045	0.0488
21 days	twi_ss.1636.1|g.2868	-2.46	3.74	12.98	0.0045	0.0489
21 days	twi_ss.17297.2|g.22079	-2.39	5.38	12.99	0.0045	0.0489

21 days	twi_ss.182.3|g.285	0.59	6.60	12.99	0.0045	0.0489
21 days	twi_ss.26147.1|g.34632	-1.67	4.34	12.98	0.0045	0.0489
21 days	twi_ss.29120.4|g.39036	-4.61	2.65	13.73	0.0045	0.0489
21 days	twi_ss.9511.1|g.12775	-3.55	2.82	12.99	0.0045	0.0489
21 days	twi_ss.24316.1|g.31764	0.56	7.27	12.98	0.0045	0.0489
21 days	twi_ss.4815a.1|g.7394	0.49	7.58	12.98	0.0045	0.0489
21 days	twi_ss.16834.1|g.21486	-1.80	3.73	12.97	0.0045	0.0490
21 days	twi_ss.24309.1|g.31756	-2.86	3.07	12.96	0.0046	0.0491
21 days	twi_ss.7449.1|g.10452	1.11	4.37	12.96	0.0046	0.0491
21 days	twi_ss.10729.1|g.14395	-4.92	3.00	14.69	0.0046	0.0491
21 days	twi_ss.18434.1|g.23478	-4.16	2.30	14.69	0.0046	0.0491
21 days	twi_ss.5608.1|g.8343	1.31	4.84	12.95	0.0046	0.0491
21 days	twi_ss.21510.1|g.27779	6.10	3.82	12.93	0.0046	0.0494
21 days	twi_ss.668.1|g.1194	3.94	5.88	12.92	0.0046	0.0495
21 days	twi_ss.3977.3|g.6382	-0.60	6.55	12.91	0.0046	0.0495
21 days	twi_ss.2364.1|g.4172	1.22	5.46	12.90	0.0046	0.0496
21 days	twi_ss.15552b.2|g.20117	-3.06	3.54	12.90	0.0046	0.0496
21 days	twi_ss.28639.1|g.38344	1.11	5.84	12.89	0.0046	0.0497
21 days	twi_ss.31911.2|g.43522	-2.97	2.77	12.89	0.0046	0.0497
21 days	twi_ss.29511.2|g.39821	5.61	3.25	12.88	0.0046	0.0498
21 days	twi_ss.3426.1|g.5812	1.64	3.68	12.88	0.0046	0.0498
21 days	twi_ss.4713.1|g.7267	-8.08	4.68	12.87	0.0047	0.0499
21 days	twi_ss.6683.1|g.9710	-1.89	4.27	12.87	0.0047	0.0499
21 days	twi_ss.3494.1|g.5872	1.61	4.38	12.86	0.0047	0.0499
21 days	twi_ss.15218.1|g.19806	0.69	6.49	12.86	0.0047	0.0500
21 days	twi_ss.10023.1|g.13343	0.85	5.13	12.85	0.0047	0.0500
21 days	twi_ss.16563.1|g.21179	0.75	7.37	12.85	0.0047	0.0500
21 days	twi_ss.20387.1|g.26195	0.53	7.49	12.85	0.0047	0.0501
21 days	twi_ss.31297.1|g.42567	-2.41	6.10	12.84	0.0047	0.0501
21 days	twi_ss.2739a.4|g.4903	-1.00	5.74	12.84	0.0047	0.0501
21 days	twi_ss.30794.1|g.41767	1.15	4.45	12.84	0.0047	0.0501
21 days	twi_ss.6412.1|g.9463	-2.72	3.79	12.84	0.0047	0.0501
21 days	twi_ss.15927.1|g.20467	1.02	5.36	12.82	0.0047	0.0503

21 days	twi_ss.1818.2|g.3205	1.11	5.72	12.81	0.0047	0.0504
21 days	twi_ss.2892b.2|g.5187	-1.27	4.12	12.81	0.0047	0.0504

Table S3: List of genes overexpressed 24 hours, 7 and 21 days after X-ray exposure.

The table reports at least 2 log2-fold differentially expressed and statistically significant genes after multiple comparison correction (FDR<0.05) and their human homolog genes.

Gene/Transcript ID	24H logFC	7D logFC	21D logFC	Human Gene Homolog	Homolog Description
twi_ss.10017.1			4.5	NA	NA
twi_ss.10068.1			9	**PARP3**	poly(ADP-ribose) polymerase family member 3
twi_ss.10136.1			7.2	NA	NA
twi_ss.10157.2		6.9		NA	NA
twi_ss.10270.2			6.6	**EPHA4**	EPH receptor A4
twi_ss.10279.1	6	6.1	5.7	NA	NA
twi_ss.10283.1		2.2	2.3	**SCARB2**	scavenger receptor class B member 2
twi_ss.10306.10			6.5	**DPH6**	diphthamine biosynthesis 6
twi_ss.1035.1	4.3	3.9		NA	NA
twi_ss.1036.1	4.4	4.4		NA	NA
twi_ss.10365.1			7.9	**DUS2**	dihydrouridine synthase 2
twi_ss.10479.1			2.5	**GABBR2**	gamma-aminobutyric acid type B receptor subunit 2
twi_ss.10495.1		2.1	2	NA	NA
twi_ss.10516.1	4.9	4.8	3.5	NA	NA
twi_ss.10703.2			5.3	**ROBO2**	roundabout guidance receptor 2
twi_ss.10752.1	3.2	4	3.2	**ASPN**	asporin
twi_ss.10775.1		2.1	2.4	NA	NA
twi_ss.10792.1		2.4		NA	NA
twi_ss.108.1		3	3.9	NA	NA
twi_ss.10837.3		2.6	2.6	NA	NA
twi_ss.10837.5			2.1	NA	NA
twi_ss.10838.1		2.1		NA	NA
twi_ss.10882.1	2.4	6	6	**MPEG1**	macrophage expressed 1
twi_ss.10945.1		5.3	5.1	NA	NA
twi_ss.10965.1		3.7	3.5	NA	NA
twi_ss.110a.2	5	4.3		NA	NA
twi_ss.110b.1		2.9		NA	NA
twi_ss.11312.1		2.9	3.4	NA	NA
twi_ss.11366.1		2.1	2.2	**MYOM3**	myomesin 3
twi_ss.11375.1	2.6			**PCNA**	proliferating cell nuclear antigen
twi_ss.11382.5		7.3		**PITRM1**	pitrilysin metallopeptidase 1
twi_ss.11444.1		3	3	**EPHA2**	EPH receptor A2
twi_ss.11522.1			2.1	**TYW3**	tRNA-yW synthesizing protein 3 homolog

twi_ss.11526.15			3.1	**MYOM1**	myomesin 1
twi_ss.11534.7			6.5	NA	NA
twi_ss.11539a.1		3.9	4	NA	NA
twi_ss.11539b.4		4.1	4.3	NA	NA
twi_ss.11619.2			5.1	NA	NA
twi_ss.11664.1	2.5	3.2	3	**HHIPL1**	HHIP like 1
twi_ss.1168.1		2.7		**BMP6**	bone morphogenetic protein 6
twi_ss.11722.2		6.1	7	**TNR**	tenascin R
twi_ss.11864.1		2		**RIPK4**	receptor interacting serine/threonine kinase 4
twi_ss.11909.1		3.1	2.1	NA	NA
twi_ss.120.2		2.3	2.4	NA	NA
twi_ss.12025.1		2.4		**CERT1**	ceramide transporter 1
twi_ss.12047.1		2.3		**ANKRD50**	ankyrin repeat domain 50
twi_ss.12129.2		5.2		**EXOSC2**	exosome component 2
twi_ss.12222.1		2		**CEACAM20**	CEA cell adhesion molecule 20
twi_ss.12226.1	2.3	5.3	4.9	NA	NA
twi_ss.12230.1			2	**DDAH1**	dimethylarginine dimethylaminohydrolase 1
twi_ss.12243.1		2.4	2.7	**VWA3A**	von Willebrand factor A domain containing 3A
twi_ss.12246.2		4.4	4.2	NA	NA
twi_ss.12247.1			3.8	**HOMEZ**	homeobox and leucine zipper encoding
twi_ss.12272.1	2.9			**CLEC4C**	C-type lectin domain family 4 member C
twi_ss.12332.9	7.5	8.2		**CACNA1B**	calcium voltage-gated channel subunit alpha1 B
twi_ss.12377.1		2.1		NA	NA
twi_ss.1244.5		3		**ADGRE5**	adhesion G protein-coupled receptor E5
twi_ss.12447.4	5.3			NA	NA
twi_ss.12458.1	9.3	6.8		**TTN**	titin
twi_ss.12733.1		2.3	2.6	NA	NA
twi_ss.12746.1		5.7		**CCDC84**	coiled-coil domain containing 84
twi_ss.12866.1	2.3			**MSRA**	methionine sulfoxide reductase A
twi_ss.12872.3		2.7	4.1	**COL11A2**	collagen type XI alpha 2 chain
twi_ss.12920.1		2.8	3.2	NA	NA
twi_ss.1295.2	4.1			NA	NA
twi_ss.12972.1	2.2	3.2	2.1	NA	NA
twi_ss.12990.2	5.9		6	NA	NA
twi_ss.12990.3	5.9		6	**WDR75**	WD repeat domain 75
twi_ss.13145.1			2.8	NA	NA
twi_ss.13146.1		2.6	2.2	NA	NA

twi_ss.13146.5		2.4	2.5	NA	NA
twi_ss.13146.9		4.4	4.1	NA	NA
twi_ss.13190.1			2.9	NA	NA
twi_ss.13326.1			3.7	NA	NA
twi_ss.13327.1			3	NA	NA
twi_ss.13436.1		2.6	2.3	NA	NA
twi_ss.13566.1	3.9	4	2.4	**CD163L1**	CD163 molecule like 1
twi_ss.13698.1		2.4	2.4	**FAT3**	FAT atypical cadherin 3
twi_ss.13791.1			2.1	NA	NA
twi_ss.13791.2			2.2	NA	NA
twi_ss.13807.1		3.1	2.6	**MOXD1**	monooxygenase DBH like 1
twi_ss.13838.1	3	3.6	2.9	**PXDN**	peroxidasin
twi_ss.13856.1		2.3		NA	NA
twi_ss.1403.2	8.6	7.5		NA	NA
twi_ss.14090.1	2.5			**WDR83OS**	WD repeat domain 83 opposite strand
twi_ss.14116a.1		2.1		NA	NA
twi_ss.14116b.2		3.2	3.3	NA	NA
twi_ss.14119.1	2.7	3.8	3.6	NA	NA
twi_ss.14260.2		2.2		NA	NA
twi_ss.14278.1	7.8	6.7	6.4	NA	NA
twi_ss.1434.1			3.6	**UGT2B7**	UDP glucuronosyltransferase family 2 member B7
twi_ss.14354.1	2.5	3.2	2.1	**SOX14**	SRY-box transcription factor 14
twi_ss.1440.1		2.1		**UGT2B7**	UDP glucuronosyltransferase family 2 member B7
twi_ss.1442.2		3.6	3.3	NA	NA
twi_ss.14541.1	5.5			**SHFL**	shiftless antiviral inhibitor of ribosomal frameshifting
twi_ss.14576.1		2.7	2.7	**PCSK9**	proprotein convertase subtilisin/kexin type 9
twi_ss.14612.3		3	2.3	**NOTCH2**	notch receptor 2
twi_ss.14702.1		3.4		NA	NA
twi_ss.14730.1		2.2	2.8	NA	NA
twi_ss.14756.4	3.7	3.3		NA	NA
twi_ss.14780.1	2.3	2.6	2.3	NA	NA
twi_ss.14839.1	6	6.7	5.5	NA	NA
twi_ss.14840.1			2.1	**OTUD1**	OTU deubiquitinase 1
twi_ss.14851.1	2.5			NA	NA
twi_ss.1490.1	3.1			**TLX2**	T cell leukemia homeobox 2
twi_ss.14940.1			2.1	**ASTL**	astacin like metalloendopeptidase
twi_ss.14987.1		3.2	3.4	NA	NA

twi_ss.15124.1		2.1		**GABBR2**	gamma-aminobutyric acid type B receptor subunit 2
twi_ss.15132.1			3.1	NA	NA
twi_ss.1543.4	7	7.1		NA	NA
twi_ss.15435.1			2.5	NA	NA
twi_ss.15436.1			2.9	NA	NA
twi_ss.15550.1			3.2	NA	NA
twi_ss.15647.3			6.2	**SACS**	sacsin molecular chaperone
twi_ss.15661.2		2.3		NA	NA
twi_ss.15729.1		3.1	2.8	NA	NA
twi_ss.15781.1	2.1	2.4		NA	NA
twi_ss.15787.1		2.3		NA	NA
twi_ss.1579a.1		3	3	NA	NA
twi_ss.15844b.3	5.8		6.4	NA	NA
twi_ss.15849.1		2.4	2.5	**PTPRD**	protein tyrosine phosphatase receptor type D
twi_ss.16049.1		3.4		NA	NA
twi_ss.16232.2			6.2	**UTP15**	UTP15 small subunit processome component
twi_ss.16285.1		2.5		**MYO15A**	myosin XVA
twi_ss.16288.1		2.8	3.6	**CDIP1**	cell death inducing p53 target 1
twi_ss.16289.1			5.3	**MRPL27**	mitochondrial ribosomal protein L27
twi_ss.16652.3			2	**SCUBE3**	signal peptide, CUB domain and EGF like domain containing 3
twi_ss.16656.2	7.3	10.4	10.3	**PHF8**	PHD finger protein 8
twi_ss.16738.1		2.4		NA	NA
twi_ss.16822.1			3	NA	NA
twi_ss.16882.1		2	2.4	NA	NA
twi_ss.17159.1			2.3	**GNPNAT1**	glucosamine-phosphate N-acetyltransferase 1
twi_ss.17277a.13			5	**PTPRA**	protein tyrosine phosphatase receptor type A
twi_ss.17303.1			2.7	NA	NA
twi_ss.17326.6	8.2			**CUBN**	cubilin
twi_ss.17326.7	8.6			**CUBN**	cubilin
twi_ss.17473.4		5.5		**ARMC3**	armadillo repeat containing 3
twi_ss.17485.1		2		NA	NA
twi_ss.1749.3			7.3	NA	NA
twi_ss.17531b.1	2.3			**CRYM**	crystallin mu
twi_ss.17571.5			8	NA	NA
twi_ss.1765.1	3	2.5		**GSTM5**	glutathione S-transferase mu 5
twi_ss.1766.1	2.2			**TRMU**	tRNA 5-methylaminomethyl-2-thiouridylate methyltransferase
twi_ss.17801.1		3.5	2.9	**SNED1**	sushi, nidogen and EGF like domains 1

twi_ss.17802.1	6.3	8.1	7	**SUSD2**	sushi domain containing 2
twi_ss.17899.1	3.5	3.3	2.1	NA	NA
twi_ss.17908.1	2.6	3.5	2.4	NA	NA
twi_ss.17964.1		2.8	2.4	**LARP6**	La ribonucleoprotein 6, translational regulator
twi_ss.18100.4		5		NA	NA
twi_ss.18212.3		6.4		**ANKRD29**	ankyrin repeat domain 29
twi_ss.18250.1		2.6	2.8	NA	NA
twi_ss.18368.5	6.3			NA	NA
twi_ss.18580.1			7.1	NA	NA
twi_ss.18584.1	7.9			**DHX33**	DEAH-box helicase 33
twi_ss.18595.1		4.5	5.1	NA	NA
twi_ss.18660b.1		2.7		NA	NA
twi_ss.18687.1		3.4		NA	NA
twi_ss.1871.1		3	2.8	NA	NA
twi_ss.18721.1			2.4	NA	NA
twi_ss.18795.3		3.4	3.1	NA	NA
twi_ss.18795.4		3	2.6	**PRSS12**	serine protease 12
twi_ss.18979.1			6.8	NA	NA
twi_ss.19023.1		2.6	3.2	NA	NA
twi_ss.19091.1			2.1	**FAM207A**	family with sequence similarity 207 member A
twi_ss.19155.1			2.4	NA	NA
twi_ss.19282.2	5.6			**PTCD1**	pentatricopeptide repeat domain 1
twi_ss.19329a.2		2.2	2.4	**RO60**	Ro60, Y RNA binding protein
twi_ss.19345.11		6	6.2	**NEO1**	neogenin 1
twi_ss.19378.2	4.5		3.8	**ERCC1**	ERCC Excision Repair 1, Endonuclease Non-Catalytic Subunit
twi_ss.19384.1	8.7	10	7.4	**PRRT1**	proline rich transmembrane protein 1
twi_ss.19415.1	4	3.9	4.6	NA	NA
twi_ss.19449.1	4.2			NA	NA
twi_ss.19570.5	6.7	8.3	7.4	NA	NA
twi_ss.19625.1		2.2		NA	NA
twi_ss.19635.1		2.1		**TRIP12**	thyroid hormone receptor interactor 12
twi_ss.19831.9			5.4	**DNAH3**	dynein axonemal heavy chain 3
twi_ss.19836.2			5.5	**TERT**	telomerase reverse transcriptase
twi_ss.19882.1		2.3	2.3	NA	NA
twi_ss.19885.1		3		NA	NA
twi_ss.1994.1	3	2.7	2.2	**FBXL15**	F-box and leucine rich repeat protein 15
twi_ss.19960a.4	7.1	7.3	7.4	NA	NA

twi_ss.20004.1			7.9	NA	NA
twi_ss.20209.2		7.3		NA	NA
twi_ss.20264c.8	7.2	7.5	8.2	NA	NA
twi_ss.20282.1	3.3	2.4		**PPM1L**	protein phosphatase, Mg2+/Mn2+ dependent 1L
twi_ss.20445.7			2.1	**ANK2**	ankyrin 2
twi_ss.20452.1	2	2.2		**SOX14**	SRY-box transcription factor 14
twi_ss.20491.1	2.3			NA	NA
twi_ss.20516a.5		6.8		**SORL1**	sortilin related receptor 1
twi_ss.20735.1	6.2	9.3	8.9	NA	NA
twi_ss.20795.1	7.5	11.2	10	NA	NA
twi_ss.20959.1		5.4		NA	NA
twi_ss.20961a.1		2.1	2.2	**PRSS12**	serine protease 12
twi_ss.21051.1	3.7	3.8	2.9	**FBLN5**	fibulin 5
twi_ss.21054.4	5.6			NA	NA
twi_ss.21105.1	6.3	6.2	5.2	**HMCN1**	hemicentin 1
twi_ss.21105.11		4.3		NA	NA
twi_ss.21105.2	6	7	6.2	**HMCN1**	hemicentin 1
twi_ss.21105.3	5.4	6.1	5.2	**SCUBE1**	signal peptide, CUB domain and EGF like domain containing 1
twi_ss.21105.6	3.3	4.9	3.9	NA	NA
twi_ss.21105.9		10.6	9.6	**SCUBE1**	signal peptide, CUB domain and EGF like domain containing 1
twi_ss.21143.1		6.9		NA	NA
twi_ss.21197a.2	2.1		2.5	**RPS15A**	ribosomal protein S15a
twi_ss.21205.1		3	4.3	NA	NA
twi_ss.21211.1			2.7	NA	NA
twi_ss.21214.1		2.1	2.2	NA	NA
twi_ss.21288a.1		2.3	2.4	**ANKRD12**	ankyrin repeat domain 12
twi_ss.21316b.3	3	2.9	2.1	**COL6A1**	collagen type VI alpha 1 chain
twi_ss.21329.1	6.8	6.6	6.8	**PRSS12**	serine protease 12
twi_ss.21425.2	3.8	4	4.3	NA	NA
twi_ss.21448.1	2.4			**MRE11**	MRE11 homolog, double strand break repair nuclease
twi_ss.21453b.5			6.1	NA	NA
twi_ss.21458.1	2.1			NA	NA
twi_ss.21482.1	2.4			**DLL4**	delta like canonical Notch ligand 4
twi_ss.21510.1			6.1	**MYCBP2**	MYC binding protein 2
twi_ss.21564.1	2.2			**ALOX5**	arachidonate 5-lipoxygenase
twi_ss.21600.1			3.7	NA	NA
twi_ss.21602.1			5.2	NA	NA

twi_ss.21610.1		**2.2**	**2.9**	NA	NA
twi_ss.21610.4		**2.7**	**2.2**	NA	NA
twi_ss.21610.5		**2.9**	**2.7**	NA	NA
twi_ss.21610.6		**3.5**	**2.7**	NA	NA
twi_ss.21631b.4		**3.9**	**4.1**	NA	NA
twi_ss.21657.1	**2.2**			NA	NA
twi_ss.2166.6			**5.8**	**HERC1**	HECT and RLD domain containing E3 ubiquitin protein ligase family member 1
twi_ss.21663.2		**7.6**	**6.6**	NA	NA
twi_ss.21664c.11			**2.4**	**SSC5D**	scavenger receptor cysteine rich family member with 5 domains
twi_ss.21664c.3		**4.1**		NA	NA
twi_ss.21664c.8		**2.8**		NA	NA
twi_ss.21665.1		**3.1**	**2.7**	NA	NA
twi_ss.21665.3		**7.3**	**6.8**	NA	NA
twi_ss.21665.4		**2.4**		NA	NA
twi_ss.21704.3	**6.6**			**NSUN7**	NOP2/Sun RNA methyltransferase family member 7
twi_ss.2185.2		**4.4**		**LANCL2**	LanC like 2
twi_ss.21908.1	**2.8**			**ESCO1**	establishment of sister chromatid cohesion N-acetyltransferase 1
twi_ss.21964.1			**2.4**	**SLC8A3**	solute carrier family 8 member A3
twi_ss.22010.1			**2.4**	NA	NA
twi_ss.22111.1	**5.4**			**KIAA0895L**	KIAA0895 like
twi_ss.22212.1	**6.9**			NA	NA
twi_ss.22218e.5		**3.7**		NA	NA
twi_ss.22218e.7	**6.9**	**7.8**	**6.9**	NA	NA
twi_ss.22219.1		**2.3**	**2.2**	NA	NA
twi_ss.2232.1		**2.8**		**TST**	thiosulfate sulfurtransferase
twi_ss.22376.1	**2.3**			**LIG3**	DNA ligase 3
twi_ss.2240.1			**2.5**	NA	NA
twi_ss.22433.4		**5.8**		**TNFSF10**	TNF superfamily member 10
twi_ss.22497.1		**2.2**		NA	NA
twi_ss.22524.1			**2**	**ERVK-6**	endogenous retrovirus group K member 6, envelope
twi_ss.22642.1	**8.8**	**8.2**		NA	NA
twi_ss.22647.1	**4**	**3.8**		NA	NA
twi_ss.2267.1		**2.8**		**NOTCH1**	notch receptor 1
twi_ss.22914.1		**2.5**		NA	NA
twi_ss.22998.1			**3.3**	NA	NA
twi_ss.22999.1			**3.3**	NA	NA
twi_ss.23060.1	**2.8**			**LAMA5**	laminin subunit alpha 5

twi_ss.2312b.1		**4.8**	**5.1**	NA	NA
twi_ss.23153.1		**4**		NA	NA
twi_ss.23359.1			**2.1**	**DERA**	deoxyribose-phosphate aldolase
twi_ss.23402.1		**2.7**	**3.2**	NA	NA
twi_ss.235.6			**2.4**	NA	NA
twi_ss.23514.1		**4.4**	**4.1**	**MFSD12**	major facilitator superfamily domain containing 12
twi_ss.23514.2		**4.7**	**4.4**	**MFSD12**	major facilitator superfamily domain containing 12
twi_ss.23516.1		**2.3**	**2.5**	**RNASEH2A**	ribonuclease H2 subunit A
twi_ss.23535.5	**5.3**		**4.7**	**GPHN**	**gephyrin**
twi_ss.23575.1	**2.4**	**2.5**	**2.5**	**MBNL2**	muscleblind like splicing regulator 2
twi_ss.23636.4			**5**	**THG1L**	tRNA-histidine guanylyltransferase 1 like
twi_ss.23649.1		**2.1**		NA	NA
twi_ss.23722.1	**2.1**	**2.3**		**CPNE1**	copine 1
twi_ss.2376.1		**2.7**	**3.6**	NA	NA
twi_ss.23785a.2		**2.1**		NA	NA
twi_ss.23802.1		**5.8**		**ARRDC2**	arrestin domain containing 2
twi_ss.23805.1	**3.9**	**6.1**		**ARRB1**	arrestin beta 1
twi_ss.23819.1		**2.6**	**2.7**	NA	NA
twi_ss.23853b.2		**2.1**	**2.4**	**JAG2**	jagged canonical Notch ligand 2
twi_ss.23880.1		**4.2**	**4.4**	NA	NA
twi_ss.2395.1			**2.4**	NA	NA
twi_ss.2398a.2			**2.7**	NA	NA
twi_ss.24028.2		**3.3**	**2.3**	NA	NA
twi_ss.24034.3		**8**		**KANK1**	KN motif and ankyrin repeat domains 1
twi_ss.24052.1	**5**	**3.8**	**3.6**	NA	NA
twi_ss.24052.2	**5**	**3.8**	**3.6**	NA	NA
twi_ss.24090.1			**2**	**GAR1**	GAR1 ribonucleoprotein
twi_ss.24106.1	**5.2**	**4.7**	**4.2**	**ROBO2**	roundabout guidance receptor 2
twi_ss.24136.1	**3.1**			**FANCI**	FA complementation group I
twi_ss.24166.1	**5.9**	**3.1**	**3.1**	**SEPSECS**	Sep (O-phosphoserine) tRNA:Sec (selenocysteine) tRNA synthase
twi_ss.24215.6		**6.9**		NA	NA
twi_ss.2422.1	**4**	**4.8**	**3.3**	NA	NA
twi_ss.2422.2		**5.4**		NA	NA
twi_ss.24240.1			**2.7**	**DDAH1**	dimethylarginine dimethylaminohydrolase 1
twi_ss.24269.1		**2.2**	**2.5**	NA	NA
twi_ss.24311.2	**7.2**			**RUFY2**	RUN and FYVE domain containing 2
twi_ss.24352c.3	**7.1**	**8**		NA	NA

twi_ss.24497.2	**2.7**	**3.5**	**3.5**	NA	NA
twi_ss.24497.5		**5.7**	**6.4**	NA	NA
twi_ss.24627.2	**5.8**		**6.9**	NA	NA
twi_ss.24697.1			**2.8**	NA	NA
twi_ss.24735.5	**2.6**	**3.1**		**NR2F1**	nuclear receptor subfamily 2 group F member 1
twi_ss.24735.7		**2.7**		**NR5A2**	nuclear receptor subfamily 5 group A member 2
twi_ss.24735.8	**2.4**	**2.3**		**RXRG**	retinoid X receptor gamma
twi_ss.24823.1	**2.4**	**3.8**	**2.5**	**ALOX5**	arachidonate 5-lipoxygenase
twi_ss.2483.2			**7.2**	NA	NA
twi_ss.2485.1		**2.3**		NA	NA
twi_ss.24955.1		**2.2**	**2.3**	**NHLRC3**	NHL repeat containing 3
twi_ss.24962.1	**2.6**			**DDR2**	discoidin domain receptor tyrosine kinase 2
twi_ss.24967.1	**5.4**		**4**	NA	NA
twi_ss.24976.2		**6.8**		**DHPS**	deoxyhypusine synthase
twi_ss.25105.5		**7.1**	**6.7**	NA	NA
twi_ss.2518.1		**3.4**	**2.5**	NA	NA
twi_ss.25243.1			**4.5**	**PRMT8**	protein arginine methyltransferase 8
twi_ss.25299b.2	**2.3**	**5.2**	**4.5**	**MOXD1**	monooxygenase DBH like 1
twi_ss.2531.2		**5.6**	**6.4**	**TBC1D15**	TBC1 domain family member 15
twi_ss.25312.1		**2.8**	**3.1**	**TNR**	tenascin R
twi_ss.25312.3	**4.9**	**5.5**	**5.8**	**FCN1**	ficolin 1
twi_ss.25312.4		**3.1**	**3.4**	**TNR**	tenascin R
twi_ss.25457.1		**2.1**	**2.7**	**SLC25A21**	solute carrier family 25 member 21
twi_ss.25621.2	**7.1**	**6.5**		NA	NA
twi_ss.25693a.1		**5.5**	**5.4**	NA	NA
twi_ss.25693b.2		**4.4**	**4.6**	NA	NA
twi_ss.25742.1	**2.6**	**2.1**		NA	NA
twi_ss.25745.1	**2.7**			**FANCD2**	FA complementation group D2
twi_ss.258.2	**5.4**	**5.2**		**ADCK2**	aarF domain containing kinase 2
twi_ss.25813.1		**2.3**	**2.6**	**AS3MT**	arsenite methyltransferase
twi_ss.25822.1	**5**			**GINS4**	GINS complex subunit 4
twi_ss.25822.3	**5**			**GINS4**	GINS complex subunit 4
twi_ss.25826.1		**2.3**		**MAP3K19**	mitogen-activated protein kinase kinase kinase 19
twi_ss.25842.1	**7.3**			**JAG1**	Jagged Canonical Notch Ligand 1
twi_ss.25970.2		**3.4**		**LTBP1**	latent transforming growth factor beta binding protein 1
twi_ss.25977.1			**5.7**	**NEK4**	NIMA related kinase 4
twi_ss.2604.1		**2.1**	**2.5**	**GLIPR1L1**	GLIPR1 like 1

twi_ss.26082.1			**5**	NA	NA
twi_ss.26090b.4	**5.4**		**5.2**	NA	NA
twi_ss.2611.1	**2.5**	**3.4**	**3.5**	NA	NA
twi_ss.26110.1		**4.5**	**4**	NA	NA
twi_ss.26111.1		**3.6**	**2.7**	NA	NA
twi_ss.26117a.1		**2.4**	**2.9**	**DDX58**	DExD/H-box helicase 58
twi_ss.2619.3		**2.4**		**MUC6**	mucin 6, oligomeric mucus/gel-forming
twi_ss.2619.7		**3.6**		**VWF**	von Willebrand factor
twi_ss.2632.2			**6.5**	**DSCAML1**	DS cell adhesion molecule like 1
twi_ss.26323.1	**2**			NA	NA
twi_ss.26358.1	**7.4**	**9.1**	**7.3**	**ATP6V1H**	ATPase H+ transporting V1 subunit H
twi_ss.26359.1	**4**			NA	NA
twi_ss.26378.1		**2.4**		**COL6A3**	collagen type VI alpha 3 chain
twi_ss.26399.1		**2**	**2.4**	NA	NA
twi_ss.26404.1			**2.6**	NA	NA
twi_ss.26435.1			**5.6**	**CCHCR1**	coiled-coil alpha-helical rod protein 1
twi_ss.26446.1		**2.2**		**MAFB**	MAF bZIP transcription factor B
twi_ss.26480.1		**2.7**	**3**	NA	NA
twi_ss.26516.1	**2.1**			**CTPS1**	CTP synthase 1
twi_ss.2665.8			**5.1**	**DKC1**	dyskerin pseudouridine synthase 1
twi_ss.26708.1			**2.1**	NA	NA
twi_ss.2690.3		**2.3**		NA	NA
twi_ss.26901a.2		**2.1**		**PLA2G6**	phospholipase A2 group VI
twi_ss.26932.1	**2.6**			NA	NA
twi_ss.27012.1		**3.8**	**3.1**	NA	NA
twi_ss.2705.3			**3.7**	NA	NA
twi_ss.27063.1	**8.4**	**9.6**	**7.8**	NA	NA
twi_ss.27066.1		**2**		**DUSP1**	dual specificity phosphatase 1
twi_ss.2715.1		**3.2**	**4.2**	NA	NA
twi_ss.27216.1		**2.1**		NA	NA
twi_ss.27219.1	**2**	**2.5**	**2.4**	NA	NA
twi_ss.27269.2		**7.1**	**6.7**	**TBK1**	TANK binding kinase 1
twi_ss.27318.1			**6.6**	NA	NA
twi_ss.27336a.3	**4.7**	**4.4**		**EPHA4**	EPH receptor A4
twi_ss.27361.1			**2.1**	**GATAD1**	GATA zinc finger domain containing 1
twi_ss.27562.2	**3.8**	**4.1**		**TGM2**	transglutaminase 2
twi_ss.27570.2		**4.2**		NA	NA

twi_ss.27729.1		**2.6**	**2.7**	NA	NA
twi_ss.27731.1		**2.7**	**2.5**	NA	NA
twi_ss.27768.1		**5.4**	**5.3**	NA	NA
twi_ss.27928.4			**6.8**	**ANK1**	ankyrin 1
twi_ss.27976.3		**4.3**		**KAT5**	lysine acetyltransferase 5
twi_ss.28007.1			**2**	**DMBT1**	deleted in malignant brain tumors 1
twi_ss.2800a.2		**2.1**		NA	NA
twi_ss.2800a.4		**2.4**		NA	NA
twi_ss.28010.1			**2.8**	**TRAF4**	TNF receptor associated factor 4
twi_ss.28037.1			**2.4**	NA	NA
twi_ss.28038b.1		**2.6**	**3.1**	**GBP2**	guanylate binding protein 2
twi_ss.28038b.2		**5.1**	**4.6**	**GBP3**	guanylate binding protein 3
twi_ss.28038b.4			**2.3**	**GBP4**	guanylate binding protein 4
twi_ss.28048.1	**4**	**6**	**2.7**	NA	NA
twi_ss.28101b.1		**3.8**		NA	NA
twi_ss.28101b.2		**7.2**		NA	NA
twi_ss.28211.1	**2.1**			**RPA1**	replication protein A1
twi_ss.28214.1	**2.5**			NA	NA
twi_ss.28225.3	**7.9**			**MED25**	mediator complex subunit 25
twi_ss.28284.1	**5.1**			**HSPA1A**	heat shock protein family A (Hsp70) member 1A
twi_ss.28338.2			**4.7**	**HDHD3**	haloacid dehalogenase like hydrolase domain containing 3
twi_ss.28372.1	**3.8**			NA	NA
twi_ss.28414.1	**3.4**	**3**	**3**	NA	NA
twi_ss.28424.1		**3.2**	**2.4**	**VIL1**	villin 1
twi_ss.28429.1	**2.7**	**3.5**	**4**	**TRAF2**	TNF receptor associated factor 2
twi_ss.28459.1			**6.1**	**CASTOR2**	cytosolic arginine sensor for mTORC1 subunit 2
twi_ss.28487.2		**2.3**	**2.2**	NA	NA
twi_ss.28487.3		**2.4**	**2.6**	NA	NA
twi_ss.28510a.22		**4.5**		NA	NA
twi_ss.28569.1			**5.6**	NA	NA
twi_ss.28592.1		**2.3**		NA	NA
twi_ss.28616.1		**2.6**	**2.8**	**TRPM6**	transient receptor potential cation channel subfamily M member 6
twi_ss.28657.1			**2**	NA	NA
twi_ss.2869.1	**5.8**	**6**	**5.8**	**COP1**	COP1 E3 ubiquitin ligase
twi_ss.2869.2	**3.6**	**3.7**	**3.3**	**COP1**	COP1 E3 ubiquitin ligase
twi_ss.28712.2			**4.3**	NA	NA
twi_ss.28718.1			**6.3**	**PRPF19**	pre-mRNA processing factor 19

twi_ss.28718.3			6.3	**PRPF19**	pre-mRNA processing factor 19
twi_ss.28745.6	5.2			**FES**	FES proto-oncogene, tyrosine kinase
twi_ss.28804.1			2	NA	NA
twi_ss.28809.1			2.5	NA	NA
twi_ss.28816.2		2.8	3.5	NA	NA
twi_ss.28866.1		3.2	4	NA	NA
twi_ss.28912.1	5.2	5.3	4.3	**ANK3**	ankyrin 3
twi_ss.28950.1			2.3	**TRPA1**	transient receptor potential cation channel subfamily A member 1
twi_ss.28950.2			2.3	**TRPA1**	transient receptor potential cation channel subfamily A member 1
twi_ss.28977.1			4.7	NA	NA
twi_ss.28981.7			4.3	**FUCA2**	alpha-L-fucosidase 2
twi_ss.28999a.1	4.7			**CYP3A5**	cytochrome P450 family 3 subfamily A member 5
twi_ss.29124.1			2.9	NA	NA
twi_ss.29126.1	3.9	3.2		**PLSCR4**	phospholipid scramblase 4
twi_ss.29191.5		6.8		NA	NA
twi_ss.29211.1	2.4	2.2		NA	NA
twi_ss.29406a.1		2.2	2.2	NA	NA
twi_ss.29406a.2		6		NA	NA
twi_ss.29443.2			2.1	NA	NA
twi_ss.29475.1		3.8	4.3	**TRPA1**	transient receptor potential cation channel subfamily A member 1
twi_ss.29504.3			5.3	**LTA4H**	leukotriene A4 hydrolase
twi_ss.29504.8			3.1	**RNPEPL1**	arginyl aminopeptidase like 1
twi_ss.29511.2			5.6	NA	NA
twi_ss.29512.1	2.3			**TFPI**	tissue factor pathway inhibitor
twi_ss.29546.1		5.4	5.5	**TNC**	tenascin C
twi_ss.29547.1		7	6.3	NA	NA
twi_ss.29566.2		2.6	2.2	NA	NA
twi_ss.29581.3			4.2	**MET**	MET proto-oncogene, receptor tyrosine kinase
twi_ss.29590a.5		4.3	3.2	**FBLN2**	fibulin 2
twi_ss.29617.1	2.8	3.9	2.8	**TRIM71**	tripartite motif containing 71
twi_ss.29721.1	2.2			NA	NA
twi_ss.29759b.1		3.6	3.2	NA	NA
twi_ss.29858.1			6.3	**KDM2B**	lysine demethylase 2B
twi_ss.29902.4			5.2	**RAB3GAP2**	RAB3 GTPase activating non-catalytic protein subunit 2
twi_ss.29994.1			2.2	**FRS2**	fibroblast growth factor receptor substrate 2
twi_ss.30001b.1			2.1	**PLA2G6**	phospholipase A2 group VI
twi_ss.30027a.5	6.3	8.5	7.6	NA	NA

twi_ss.30027a.7		**3.2**		NA	NA
twi_ss.30027b.1	**2.8**	**3.4**		NA	NA
twi_ss.30027b.2	**2.8**	**4.2**	**2.5**	NA	NA
twi_ss.30027c.4		**3.6**	**2.6**	NA	NA
twi_ss.3007.1		**2.2**	**2.1**	NA	NA
twi_ss.30096b.2		**2.2**		**ROR2**	receptor tyrosine kinase like orphan receptor 2
twi_ss.30108.1	**2.1**	**2.2**		**MUSK**	muscle associated receptor tyrosine kinase
twi_ss.30134.4	**3.3**	**3.1**		**PCK2**	phosphoenolpyruvate carboxykinase 2, mitochondrial
twi_ss.30137.1		**2.1**	**2.6**	NA	NA
twi_ss.30208.1		**5.9**	**5.4**	NA	NA
twi_ss.30242b.2	**7.2**		**8.3**	**HPGDS**	hematopoietic prostaglandin D synthase
twi_ss.30351.1			**2**	NA	NA
twi_ss.30479.1		**2.2**		NA	NA
twi_ss.30481.2	**2.6**			**SERBP1**	SERPINE1 mRNA binding protein 1
twi_ss.30546.1	**4.4**	**5**	**4.6**	**C1QTNF9B**	C1q and TNF related 9B
twi_ss.30551.1		**4.5**	**4.3**	**LRP1**	LDL receptor related protein 1
twi_ss.30590.3			**7.2**	NA	NA
twi_ss.30590.6			**6.5**	**RACGAP1**	Rac GTPase activating protein 1
twi_ss.30700b.6			**6.7**	**PHF19**	PHD finger protein 19
twi_ss.30724.1	**2.6**	**3.7**	**4.2**	NA	NA
twi_ss.3076.1		**3.5**	**2.5**	**DMBT1**	deleted in malignant brain tumors 1
twi_ss.30766.1			**4.6**	NA	NA
twi_ss.30803a.1	**2.9**	**3**	**2.1**	NA	NA
twi_ss.30809.1		**2.2**		**ANKRD50**	ankyrin repeat domain 50
twi_ss.30923.2	**4.1**			**C9orf116**	chromosome 9 open reading frame 116
twi_ss.30997.9	**6**			NA	NA
twi_ss.31022.2		**3.6**		NA	NA
twi_ss.31032.3			**5.6**	**AASS**	aminoadipate-semialdehyde synthase
twi_ss.31108.5			**5.1**	NA	NA
twi_ss.31154.2	**7.2**			**NOX5**	NADPH oxidase 5
twi_ss.3117.1		**2.1**	**2.2**	NA	NA
twi_ss.31219.1		**2.1**		**RET**	ret proto-oncogene
twi_ss.31247.1	**8.3**	**5.2**		**ANGPTL1**	angiopoietin like 1
twi_ss.3131.1			**2.2**	**GABBR1**	gamma-aminobutyric acid type B receptor subunit 1
twi_ss.31312.1		**7.3**		**SCRIB**	scribble planar cell polarity protein
twi_ss.31312.12		**7.3**		**SCRIB**	scribble planar cell polarity protein
twi_ss.31312.2		**7.3**		**SCRIB**	scribble planar cell polarity protein

twi_ss.31312.3		**7.3**		**SCRIB**	scribble planar cell polarity protein
twi_ss.31312.4		**7.3**		**SCRIB**	scribble planar cell polarity protein
twi_ss.31312.6		**7.3**		**SCRIB**	scribble planar cell polarity protein
twi_ss.31312.7		**7.3**		**SCRIB**	scribble planar cell polarity protein
twi_ss.31358.1			**2.1**	NA	NA
twi_ss.31413.1		**2.3**		NA	NA
twi_ss.3152.1		**2.7**	**2.7**	**GDF15**	growth differentiation factor 15
twi_ss.31673.1		**2.8**	**2.5**	**NLK**	nemo like kinase
twi_ss.31729.1	**2.1**	**3.4**	**4**	**DAZAP1**	DAZ associated protein 1
twi_ss.31777.1	**4.6**	**5.1**	**4.3**	NA	NA
twi_ss.31853a.1		**5.7**		**COL6A6**	collagen type VI alpha 6 chain
twi_ss.31899b.1	**5.7**			**GPR157**	G protein-coupled receptor 157
twi_ss.31909.2			**4.1**	NA	NA
twi_ss.31915.4			**7.7**	**MIF4GD**	MIF4G domain containing
twi_ss.32065.1			**4.5**	**SVEP1**	sushi, von Willebrand factor type A, EGF and pentraxin domain containing 1
twi_ss.32065.2		**4.1**	**4.9**	NA	NA
twi_ss.3226.1			**2.7**	**SPOPL**	speckle type BTB/POZ protein like
twi_ss.329.1		**2.2**		**ZFP36L1**	ZFP36 ring finger protein like 1
twi_ss.3382.1		**3.1**		**SUSD2**	sushi domain containing 2
twi_ss.339.1		**3.3**	**3.2**	NA	NA
twi_ss.3531.2		**2.5**	**2.6**	**ADGRL2**	adhesion G protein-coupled receptor L2
twi_ss.3533.1	**6.7**	**6.3**	**6.6**	**SUPT6H**	SPT6 homolog, histone chaperone and transcription elongation factor
twi_ss.3533.2	**6.7**	**6.3**	**6.6**	**SUPT6H**	SPT6 homolog, histone chaperone and transcription elongation factor
twi_ss.3533.3	**6.7**	**6.3**	**6.6**	**SUPT6H**	SPT6 homolog, histone chaperone and transcription elongation factor
twi_ss.3533.4	**6.7**	**6.3**	**6.6**	**SUPT6H**	SPT6 homolog, histone chaperone and transcription elongation factor
twi_ss.3623.1	**5.7**			**CRYBG2**	crystallin beta-gamma domain containing 2
twi_ss.3627.1		**2.7**	**2.6**	NA	NA
twi_ss.366.1			**3.3**	NA	NA
twi_ss.3712.1	**7.6**	**6.6**	**5**	NA	NA
twi_ss.3722.1			**3**	NA	NA
twi_ss.3796.3		**4**	**3.7**	**FBN1**	fibrillin 1
twi_ss.3847.1			**5.4**	**SNTA1**	syntrophin alpha 1
twi_ss.3973.1	**3.3**	**3.7**		**MUC4**	mucin 4, cell surface associated
twi_ss.4076.2		**6.2**		**LRRC7**	leucine rich repeat containing 7
twi_ss.4103.1	**2.4**	**2.5**		**PRXL2A**	peroxiredoxin like 2A
twi_ss.4179.1		**3.4**	**4.5**	NA	NA
twi_ss.4180.1			**3.1**	NA	NA

twi_ss.4254.1		**5.1**		NA	NA
twi_ss.4260.1			**3.7**	NA	NA
twi_ss.4264.1		**3.7**	**3**	NA	NA
twi_ss.4286.1			**2.1**	**WARS1**	tryptophanyl-tRNA synthetase 1
twi_ss.4304a.1	**2.3**	**2.4**	**2**	NA	NA
twi_ss.4312.1		**9.2**	**9**	NA	NA
twi_ss.4335.1		**3.1**	**2.8**	**SCARB2**	scavenger receptor class B member 2
twi_ss.4355.1		**2.4**		**TRIM71**	tripartite motif containing 71
twi_ss.4370.1		**3.7**	**2.3**	NA	NA
twi_ss.4371.1		**4.2**	**4**	NA	NA
twi_ss.4463.1		**3.5**	**3.4**	NA	NA
twi_ss.4477.1		**4.8**	**4.9**	NA	NA
twi_ss.4477.2		**4.8**	**4.5**	NA	NA
twi_ss.4477.3			**7.2**	NA	NA
twi_ss.4527.1		**2.5**		NA	NA
twi_ss.4654.1		**2.4**	**3.6**	NA	NA
twi_ss.469.1		**2.1**		**NCAM1**	neural cell adhesion molecule 1
twi_ss.4816.1			**5.4**	NA	NA
twi_ss.4828.1			**3.1**	**ROR2**	receptor tyrosine kinase like orphan receptor 2
twi_ss.4865.5			**5.9**	**PRPF4B**	pre-mRNA processing factor 4B
twi_ss.4876.1	**2.5**			**DDR1**	discoidin domain receptor tyrosine kinase 1
twi_ss.4918.1		**7.3**		NA	NA
twi_ss.4929.1		**2.1**		**DAGLB**	diacylglycerol lipase beta
twi_ss.4972.1			**2.9**	NA	NA
twi_ss.4977.9	**8.8**	**8.5**	**9.5**	**SPG11**	SPG11 vesicle trafficking associated, spatacsin
twi_ss.4989.1	**2.2**			**COP1**	COP1 E3 ubiquitin ligase
twi_ss.4992.1			**2.3**	NA	NA
twi_ss.5058.2	**3.6**	**3.8**		NA	NA
twi_ss.5112.1	**2.7**	**3.1**		NA	NA
twi_ss.5182.1	**3.9**			**DNAJB5**	DnaJ heat shock protein family (Hsp40) member B5
twi_ss.5283.3			**7.3**	**ASNS**	asparagine synthetase (glutamine-hydrolyzing)
twi_ss.5415.4		**3.6**	**3.5**	NA	NA
twi_ss.5416.1			**2.7**	**HPRT1**	hypoxanthine phosphoribosyltransferase 1
twi_ss.5474.2			**3.6**	NA	NA
twi_ss.5474.4			**2.1**	NA	NA
twi_ss.5526a.2	**3.6**	**3.2**		NA	NA
twi_ss.5526b.1	**4.2**	**3.9**		NA	NA

twi_ss.558.1	2.1	2.2		NA	NA
twi_ss.5590a.3		4.3		**UBC**	ubiquitin C
twi_ss.5628.6		6.5		**MET**	MET proto-oncogene, receptor tyrosine kinase
twi_ss.5643.1	2.2	5.3	5.5	NA	NA
twi_ss.5716.1		3.9	3.5	NA	NA
twi_ss.5718.1		2.1	2.2	NA	NA
twi_ss.5738.2		2.2		NA	NA
twi_ss.574.2	2.3			**PIK3R5**	phosphoinositide-3-kinase regulatory subunit 5
twi_ss.5784.2		5.3		NA	NA
twi_ss.5805a.2			3.4	NA	NA
twi_ss.6018.1	4.9	6.5		NA	NA
twi_ss.6037.1		3	3	NA	NA
twi_ss.6040.1		2.1		NA	NA
twi_ss.6045.1		2.7	2.5	**TTN**	titin
twi_ss.6146.8		5.6		**DMBT1**	deleted in malignant brain tumors 1
twi_ss.6202.1		2.1		**POSTN**	periostin
twi_ss.6204.1		2.5		**POSTN**	periostin
twi_ss.6314.1	2.1			**CALCOCO2**	calcium binding and coiled-coil domain 2
twi_ss.6452.1	3.6	4.1	3	**CPAMD8**	C3 and PZP like alpha-2-macroglobulin domain containing 8
twi_ss.6495.1			2.5	NA	NA
twi_ss.6499.1			2.6	NA	NA
twi_ss.6558.5		2.7	3.3	NA	NA
twi_ss.6603b.1	3	3.3	2.9	**MAPK3**	mitogen-activated protein kinase 3
twi_ss.6610.1	2.8	2.5	2.2	NA	NA
twi_ss.6620.1		2.8	2.6	NA	NA
twi_ss.668.1		4.5	3.9	**SZRD1**	SUZ RNA binding domain containing 1
twi_ss.6750.1	2.5	3	2.1	**TM2D1**	TM2 domain containing 1
twi_ss.6760.1		3.3		NA	NA
twi_ss.684.1			2.6	NA	NA
twi_ss.6877.1		3.7	4.3	NA	NA
twi_ss.6881.1		3.3	3.8	NA	NA
twi_ss.6884.1		4	2.7	NA	NA
twi_ss.7080.1			2.1	**DARS1**	aspartyl-tRNA synthetase 1
twi_ss.7082.1		2.6		**ETV4**	ETS variant 4
twi_ss.7156.2	5			**ITGB4**	integrin subunit beta 4
twi_ss.7206.1		2.4	2.8	NA	NA
twi_ss.7208.1			2.1	NA	NA

twi_ss.7208.2		**4.2**	**4.3**	NA	NA
twi_ss.7409.1		**3**	**3.6**	**ANTXR1**	ANTXR cell adhesion molecule 1
twi_ss.7443.1		**2**	**2.3**	NA	NA
twi_ss.7454.1	**2.7**	**2.3**		NA	NA
twi_ss.7494.4			**6**	**SLC25A25**	solute carrier family 25 member 25
twi_ss.7586.1		**2.1**	**2.3**	NA	NA
twi_ss.7623.1		**2**	**3.8**	NA	NA
twi_ss.7626.1		**2**		**DMBT1**	deleted in malignant brain tumors 1
twi_ss.7711.1		**2.8**		**ASB7**	ankyrin repeat and SOCS box protein 7 isoform 1
twi_ss.7757.3			**3.3**	**UBR5**	ubiquitin protein ligase E3 component n-recognin 5
twi_ss.7777.1		**3.5**	**3.2**	NA	NA
twi_ss.7858.12	**3.9**	**4.3**	**4.4**	NA	NA
twi_ss.7858.13		**2.5**		**DSCAML1**	DS cell adhesion molecule like 1
twi_ss.7858.3		**2.5**	**2.2**	**PTPRD**	protein tyrosine phosphatase receptor type D
twi_ss.7858.4		**3.3**		**PTPRD**	protein tyrosine phosphatase receptor type D
twi_ss.799a.3			**3.4**	**MATN2**	matrilin 2
twi_ss.8200.1		**3.1**	**2.1**	NA	NA
twi_ss.8361b.2	**2.1**	**2.8**	**2.7**	**SOCS1**	suppressor of cytokine signaling 1
twi_ss.8420.5		**4.7**		**IFRD1**	interferon related developmental regulator 1
twi_ss.8420.6		**4.7**		**IFRD1**	interferon related developmental regulator 1
twi_ss.8507.1			**2.4**	NA	NA
twi_ss.8555.1		**2**		**TRAF4**	TNF receptor associated factor 4
twi_ss.8573.1			**3.5**	NA	NA
twi_ss.8759.1	**5.6**	**5.2**		**CD163L1**	CD163 molecule like 1
twi_ss.8819.1	**6.2**	**5.5**	**5.2**	NA	NA
twi_ss.8843.1		**6.8**		NA	NA
twi_ss.8873.1		**3**	**5.4**	NA	NA
twi_ss.8875.1		**2.4**	**4.4**	NA	NA
twi_ss.8902.2	**4.7**			NA	NA
twi_ss.908.1			**2.1**	**KLHDC1**	kelch domain containing 1
twi_ss.9298.1		**4.7**		NA	NA
twi_ss.9313.5			**6.8**	NA	NA
twi_ss.9430.1		**4.5**	**3.2**	NA	NA
twi_ss.946.10			**5.9**	**MYO7A**	myosin VIIA
twi_ss.946.3			**5.9**	**MYO7A**	myosin VIIA
twi_ss.946.8			**6**	**MYO7A**	myosin VIIA
twi_ss.9501.1			**2.5**	NA	NA

twi_ss.9509.1		**3.5**		NA	NA
twi_ss.9529.1			**5**	NA	NA
twi_ss.9681.3	**5.1**			**ANK3**	ankyrin 3
twi_ss.9709.1		**6.5**	**6.3**	NA	NA
twi_ss.974.3			**6.7**	NA	NA
twi_ss.9775.1		**4.1**	**5.2**	NA	NA
twi_ss.978.1	**2.3**	**6.3**	**6.6**	NA	NA
twi_ss.981.1		**5.2**	**4.7**	NA	NA
twi_ss.9828.1		**2.5**		NA	NA
twi_ss.982c.1		**2.2**		NA	NA
twi_ss.984.1		**4.6**	**4.1**	NA	NA
twi_ss.9907.1	**3.6**	**4**	**3.1**	NA	NA
twi_ss.9920.2	**5**	**5.2**	**4.5**	NA	NA
twi_ss.9963.1			**2.7**	NA	NA

Table S4: DAVID functional annotation clustering

DAVID Knowledgebase (v2023q4)

Time	Category	Term	Count	%	PValue	Genes ID	List Total	Pop Hits	Pop Total	Fold Enrichment	FDR
24 h	Annotation Cluster 1	Enrichment Score: 3.6									
24 h	INTERPRO	IPR000742:EGF-like_dom	7	10.0	1.8E-04	3691, 54567, 8029, 4585, 28514, 84465, 3911	70	248	20667	8.333	0.030
24 h	Annotation Cluster 2	Enrichment Score: 2.913592099001449									
24 h	INTERPRO	IPR008266:Tyr_kinase_AS	6	8.6	1.9E-05	4593, 2242, 2043, 4921, 7273, 780	70	98	20667	18.076	0.007
24 h	INTERPRO	IPR020635:Tyr_kinase_cat_dom	5	7.1	2.4E-04	4593, 2242, 2043, 4921, 780	70	91	20667	16.222	0.030
24 h	SMART	SM00219:TyrKc	5	7.1	4.7E-04	4593, 2242, 2043, 4921, 780	44	91	10680	13.337	0.033
24 h	GOTERM_BP_DIRECT	GO:0036323~vascular endothelial growth factor receptor-1 signaling pathway	4	5.7	6.5E-04	4593, 2043, 4921, 780	68	49	19416	23.309	0.081
24 h	GOTERM_MF_DIRECT	GO:0005004~GPI-linked ephrin receptor activity	4	5.7	6.7E-04	4593, 2043, 4921, 780	68	49	19208	23.059	0.011
24 h	GOTERM_MF_DIRECT	GO:0005009~insulin receptor activity	4	5.7	6.7E-04	4593, 2043, 4921, 780	68	49	19208	23.059	0.011
24 h	GOTERM_MF_DIRECT	GO:0005011~macrophage colony-stimulating factor receptor activity	4	5.7	6.7E-04	4593, 2043, 4921, 780	68	49	19208	23.059	0.011
24 h	GOTERM_MF_DIRECT	GO:0005018~platelet-derived growth factor alpha-receptor activity	4	5.7	6.7E-04	4593, 2043, 4921, 780	68	49	19208	23.059	0.011
24 h	GOTERM_MF_DIRECT	GO:0005020~stem cell factor receptor activity	4	5.7	6.7E-04	4593, 2043, 4921, 780	68	49	19208	23.059	0.011
24 h	GOTERM_MF_DIRECT	GO:0008288~boss receptor activity	4	5.7	6.7E-04	4593, 2043, 4921, 780	68	49	19208	23.059	0.011
24 h	GOTERM_MF_DIRECT	GO:0036332~placental growth factor receptor activity	4	5.7	6.7E-04	4593, 2043, 4921, 780	68	49	19208	23.059	0.011
24 h	GOTERM_MF_DIRECT	GO:0038062~protein tyrosine kinase collagen receptor activity	4	5.7	6.7E-04	4593, 2043, 4921, 780	68	49	19208	23.059	0.011
24 h	GOTERM_MF_DIRECT	GO:0060175~brain-derived neurotrophic factor receptor activity	4	5.7	6.7E-04	4593, 2043, 4921, 780	68	49	19208	23.059	0.011
24 h	Annotation Cluster 3	Enrichment Score: 2.3									
24 h	INTERPRO	IPR008266:Tyr_kinase_AS	6	8.6	1.9E-05	4593, 2242, 2043, 4921, 7273, 780	70	98	20667	18.076	0.007
24 h	Annotation Cluster 4	Enrichment Score: 1.6									
24 h	GOTERM_MF_DIRECT	GO:0005524~ATP binding	14	20.0	2.3E-03	4593, 56919, 3980, 2242, 2043, 7273, 7052, 780, 1503, 774, 3303, 55687, 90956, 4921	68	1544	19208	2.561	0.029
7 days	Annotation Cluster 1	Enrichment Score: 4									
7 days	UP_SEQ_FEATURE	DOMAIN:VWFD	4	4.3	5.5E-05	7450, 4585, 4588, 56241	92	17	20502	52.435	0.016
7 days	INTERPRO	IPR001846:VWF_type-D	4	4.3	7.9E-05	7450, 4585, 4588, 56241	93	19	20667	46.784	0.013
7 days	SMART	SM00216:VWD	4	4.3	2.3E-04	7450, 4585, 4588, 56241	74	18	10680	32.072	0.017
7 days	Annotation Cluster 2	Enrichment Score: 4									
7 days	UP_SEQ_FEATURE	DOMAIN:EGF-like 1	7	7.5	2.7E-05	120114, 4052, 4585, 9369, 976, 4851, 84465	92	132	20502	11.818	0.016
7 days	UP_SEQ_FEATURE	DOMAIN:EGF-like	8	8.6	5.3E-05	120114, 4052, 4585, 9369, 6653, 976, 4851, 84465	92	218	20502	8.178	0.016
7 days	UP_SEQ_FEATURE	DOMAIN:EGF-like 2	6	6.5	9.1E-05	120114, 4052, 4585, 9369, 4851, 84465	92	102	20502	13.109	0.018
7 days	INTERPRO	IPR000742:EGF-like_dom	8	8.6	1.2E-04	120114, 4052, 4585, 9369, 976, 4851, 84465, 84628	93	248	20667	7.169	0.013
7 days	UP_KW_DOMAIN	KW-0245~EGF-like domain	8	8.6	2.8E-04	120114, 4052, 4585, 9369, 6653, 976, 4851, 84465	74	254	14566	6.200	0.002
7 days	SMART	SM00181:EGF	8	8.6	3.6E-04	120114, 4052, 4585, 9369, 976, 4851, 84465, 84628	74	195	10680	5.921	0.017
7 days	Annotation Cluster 3	Enrichment Score: 3.6									
7 days	GOTERM_BP_DIRECT	GO:0007173~epidermal growth factor receptor signaling pathway	6	6.5	6.8E-05	4593, 4233, 2043, 4920, 7429, 5979	88	95	19416	13.935	0.010
7 days	GOTERM_BP_DIRECT	GO:0036323~vascular endothelial growth factor receptor-1 signaling pathway	5	5.4	6.8E-05	4593, 4233, 2043, 4920, 5979	88	49	19416	22.514	0.010
7 days	GOTERM_MF_DIRECT	GO:0005011~macrophage colony-stimulating factor receptor activity	5	5.4	7.1E-05	4593, 4233, 2043, 4920, 5979	88	49	19208	22.273	0.002
7 days	GOTERM_MF_DIRECT	GO:0005018~platelet-derived growth factor alpha-receptor activity	5	5.4	7.1E-05	4593, 4233, 2043, 4920, 5979	88	49	19208	22.273	0.002
7 days	GOTERM_MF_DIRECT	GO:0005020~stem cell factor receptor activity	5	5.4	7.1E-05	4593, 4233, 2043, 4920, 5979	88	49	19208	22.273	0.002
7 days	GOTERM_MF_DIRECT	GO:0008288~boss receptor activity	5	5.4	7.1E-05	4593, 4233, 2043, 4920, 5979	88	49	19208	22.273	0.002
7 days	GOTERM_MF_DIRECT	GO:0036332~placental growth factor receptor activity	5	5.4	7.1E-05	4593, 4233, 2043, 4920, 5979	88	49	19208	22.273	0.002
7 days	GOTERM_MF_DIRECT	GO:0038062~protein tyrosine kinase collagen receptor activity	5	5.4	7.1E-05	4593, 4233, 2043, 4920, 5979	88	49	19208	22.273	0.002
7 days	GOTERM_MF_DIRECT	GO:0060175~brain-derived neurotrophic factor receptor activity	5	5.4	7.1E-05	4593, 4233, 2043, 4920, 5979	88	49	19208	22.273	0.002
7 days	GOTERM_MF_DIRECT	GO:0005004~GPI-linked ephrin receptor activity	5	5.4	7.1E-05	4593, 4233, 2043, 4920, 5979	88	49	19208	22.273	0.002
7 days	GOTERM_MF_DIRECT	GO:0005009~insulin receptor activity	5	5.4	7.1E-05	4593, 4233, 2043, 4920, 5979	88	49	19208	22.273	0.002
7 days	GOTERM_BP_DIRECT	GO:0035790~platelet-derived growth factor receptor-alpha signaling pathway	5	5.4	7.4E-05	4593, 4233, 2043, 4920, 5979	88	50	19416	22.064	0.010
7 days	INTERPRO	IPR008266:Tyr_kinase_AS	6	6.5	7.7E-05	4593, 4233, 2043, 4920, 7273, 5979	93	98	20667	13.606	0.013
7 days	GOTERM_MF_DIRECT	GO:0005019~platelet-derived growth factor beta-receptor activity	5	5.4	7.7E-05	4593, 4233, 2043, 4920, 5979	88	50	19208	21.827	0.002
7 days	GOTERM_MF_DIRECT	GO:0005006~epidermal growth factor receptor activity	5	5.4	7.7E-05	4593, 4233, 2043, 4920, 5979	88	50	19208	21.827	0.002
7 days	GOTERM_MF_DIRECT	GO:0005007~fibroblast growth factor receptor activity	5	5.4	7.7E-05	4593, 4233, 2043, 4920, 5979	88	50	19208	21.827	0.002
7 days	GOTERM_MF_DIRECT	GO:0005008~hepatocyte growth factor receptor activity	5	5.4	7.7E-05	4593, 4233, 2043, 4920, 5979	88	50	19208	21.827	0.002
7 days	GOTERM_BP_DIRECT	GO:0038109~Kit signaling pathway	5	5.4	8.0E-05	4593, 4233, 2043, 4920, 5979	88	51	19416	21.631	0.010
7 days	GOTERM_BP_DIRECT	GO:0038145~macrophage colony-stimulating factor signaling pathway	5	5.4	8.0E-05	4593, 4233, 2043, 4920, 5979	88	51	19416	21.631	0.010
7 days	GOTERM_MF_DIRECT	GO:0005010~insulin-like growth factor receptor activity	5	5.4	8.3E-05	4593, 4233, 2043, 4920, 5979	88	51	19208	21.399	0.002
7 days	GOTERM_MF_DIRECT	GO:0005021~vascular endothelial growth factor receptor activity	5	5.4	9.0E-05	4593, 4233, 2043, 4920, 5979	88	52	19208	20.988	0.002
7 days	GOTERM_BP_DIRECT	GO:0008543~fibroblast growth factor receptor signaling pathway	6	6.5	9.5E-05	4593, 4233, 131405, 2043, 4920, 5979	88	102	19416	12.979	0.010
7 days	GOTERM_MF_DIRECT	GO:0005005~transmembrane-ephrin receptor activity	5	5.4	9.7E-05	4593, 4233, 2043, 4920, 5979	88	53	19208	20.592	0.002
7 days	GOTERM_BP_DIRECT	GO:0031547~brain-derived neurotrophic factor receptor signaling pathway	5	5.4	1.0E-04	4593, 4233, 2043, 4920, 5979	88	54	19416	20.429	0.010
7 days	GOTERM_BP_DIRECT	GO:0035791~platelet-derived growth factor receptor-beta signaling pathway	5	5.4	1.0E-04	4593, 4233, 2043, 4920, 5979	88	54	19416	20.429	0.010
7 days	GOTERM_BP_DIRECT	GO:0048012~hepatocyte growth factor receptor signaling pathway	5	5.4	1.2E-04	4593, 4233, 2043, 4920, 5979	88	56	19416	19.700	0.011
7 days	GOTERM_BP_DIRECT	GO:0038063~collagen-activated tyrosine kinase receptor signaling pathway	5	5.4	1.3E-04	4593, 4233, 2043, 4920, 5979	88	58	19416	19.020	0.011
7 days	GOTERM_BP_DIRECT	GO:0038084~vascular endothelial growth factor signaling pathway	5	5.4	1.7E-04	4593, 4233, 2043, 4920, 5979	88	62	19416	17.793	0.013
7 days	GOTERM_BP_DIRECT	GO:0048009~insulin-like growth factor receptor signaling pathway	5	5.4	4.8E-04	4593, 4233, 2043, 4920, 5979	88	81	19416	13.620	0.031
7 days	INTERPRO	IPR001245:Ser-Thr/Tyr_kinase_cat_dom	6	6.5	5.7E-04	4593, 100526835, 4233, 2043, 4920, 5979	93	151	20667	8.830	0.021
7 days	GOTERM_BP_DIRECT	GO:0048013~ephrin receptor signaling pathway	5	5.4	5.8E-04	4593, 4233, 2043, 4920, 5979	88	85	19416	12.979	0.035
7 days	GOTERM_BP_DIRECT	GO:0004714~transmembrane receptor protein tyrosine kinase activity	4	4.3	7.3E-04	4593, 4233, 4920, 5979	88	39	19208	22.387	0.012
7 days	GOTERM_BP_DIRECT	GO:0007275~multicellular organism development	4	4.3	1.0E-03	4593, 4233, 4920, 5979	88	44	19416	20.058	0.056
7 days	INTERPRO	IPR050122:RTK	4	4.3	1.1E-03	4593, 4233, 4920, 5979	93	45	20667	19.753	0.028
7 days	GOTERM_BP_DIRECT	GO:0007169~cell surface receptor protein tyrosine kinase signaling pathway	5	5.4	1.5E-03	4593, 9068, 4233, 4920, 5979	88	110	19416	10.029	0.079
7 days	GOTERM_MF_DIRECT	GO:0004713~protein tyrosine kinase activity	5	5.4	1.7E-03	4593, 4233, 2043, 7273, 5979	88	113	19208	9.658	0.024
7 days	GOTERM_BP_DIRECT	GO:0008286~insulin receptor signaling pathway	5	5.4	1.8E-03	4593, 4233, 2043, 4920, 5979	88	116	19416	9.510	0.091
7 days	GOTERM_MF_DIRECT	GO:0035401~histone H3Y41 kinase activity	5	5.4	1.9E-03	4593, 4233, 2043, 4920, 5979	88	115	19208	9.490	0.024
7 days	GOTERM_MF_DIRECT	GO:0140801~histone H2AXY142 kinase activity	5	5.4	1.9E-03	4593, 4233, 2043, 4920, 5979	88	115	19208	9.490	0.024
7 days	Annotation Cluster 4	Enrichment Score: 3.5									
7 days	UP_SEQ_FEATURE	REPEAT:ANK	8	8.6	3.0E-05	54101, 8398, 100526835, 4851, 147463, 23189, 136371, 57182	92	199	20502	8.959	0.016
7 days	UP_SEQ_FEATURE	REPEAT:ANK 5	7	7.5	7.1E-05	54101, 8398, 4851, 147463, 23189, 136371, 57182	92	157	20502	9.936	0.017
7 days	UP_SEQ_FEATURE	REPEAT:ANK 6	6	6.5	1.4E-04	54101, 8398, 4851, 147463, 136371, 57182	92	111	20502	12.046	0.021
7 days	UP_SEQ_FEATURE	REPEAT:ANK 4	7	7.5	1.9E-04	54101, 8398, 4851, 147463, 23189, 136371, 57182	92	187	20502	8.342	0.025
7 days	INTERPRO	IPR002110:Ankyrin_rpt	8	8.6	2.0E-04	54101, 8398, 100526835, 4851, 147463, 23189, 136371, 57182	93	268	20667	6.634	0.016
7 days	INTERPRO	IPR036770:Ankyrin_rpt-contain_sf	8	8.6	2.2E-04	54101, 8398, 100526835, 4851, 147463, 23189, 136371, 57182	93	274	20667	6.488	0.016
7 days	UP_KW_DOMAIN	KW-0040~ANK repeat	8	8.6	4.2E-04	54101, 8398, 100526835, 4851, 147463, 23189, 136371, 57182	74	272	14566	5.789	0.003
7 days	UP_SEQ_FEATURE	REPEAT:ANK 3	7	7.5	4.7E-04	54101, 8398, 4851, 147463, 23189, 136371, 57182	92	222	20502	7.027	0.037
7 days	UP_SEQ_FEATURE	REPEAT:ANK 1	7	7.5	1.1E-03	54101, 8398, 4851, 147463, 23189, 136371, 57182	92	260	20502	6.000	0.064
7 days	UP_SEQ_FEATURE	REPEAT:ANK 2	7	7.5	1.1E-03	54101, 8398, 4851, 147463, 23189, 136371, 57182	92	261	20502	5.977	0.064
7 days	SMART	SM00248:ANK	8	8.6	1.8E-03	54101, 8398, 100526835, 4851, 147463, 23189, 136371, 57182	74	257	10680	4.493	0.043
7 days	Annotation Cluster 5	Enrichment Score: 3.3									
7 days	UP_SEQ_FEATURE	DOMAIN:Fibronectin type-III	7	7.5	1.4E-04	57453, 4684, 6653, 2043, 5789, 1293, 7273	92	178	20502	8.764	0.021
7 days	INTERPRO	IPR003961:FN3_dom	7	7.5	3.5E-04	57453, 4684, 6653, 2043, 5789, 1293, 7273	93	210	20667	7.408	0.018
7 days	INTERPRO	IPR036116:FN3_sf	7	7.5	3.7E-04	57453, 4684, 6653, 2043, 5789, 1293, 7273	93	212	20667	7.338	0.018
7 days	UP_SEQ_FEATURE	DOMAIN:Fibronectin type-III 1	6	6.5	3.9E-04	57453, 4684, 6653, 2043, 5789, 7273	92	139	20502	9.619	0.034
7 days	UP_SEQ_FEATURE	DOMAIN:Fibronectin type-III 2	6	6.5	3.9E-04	57453, 4684, 6653, 2043, 5789, 7273	92	139	20502	9.619	0.034
7 days	Annotation Cluster 6	Enrichment Score: 3.1									
7 days	INTERPRO	IPR036465:vWFA_dom_sf	6	6.5	9.3E-05	7450, 81578, 131873, 1293, 6738, 8904	93	102	20667	13.072	0.013

7 days	INTERPRO	IPR002035:VWF_A	5	5.4	4.3E-04	7450, 81578, 131873, 1293, 8904	93	79	20667	14.065	0.018
7 days	SMART	SM00327:VWA	5	5.4	1.6E-03	7450, 81578, 131873, 1293, 8904	74	74	10680	9.752	0.043
7 days	Annotation Cluster 7	Enrichment Score: 3.1									
7 days	INTERPRO	IPR000719:Prot_kinase_dom	10	10.8	3.9E-04	54101, 4593, 100526835, 4233, 2043, 90956, 4920, 51701, 7273, 5979	93	500	20667	4.445	0.018
7 days	UP_SEQ_FEATURE	DOMAIN:Protein kinase	10	10.8	4.1E-04	54101, 4593, 100526835, 4233, 2043, 90956, 4920, 51701, 7273, 5979	92	505	20502	4.413	0.034
7 days	INTERPRO	IPR011009:Kinase-like_dom_sf	10	10.8	7.2E-04	54101, 4593, 100526835, 4233, 2043, 90956, 4920, 51701, 7273, 5979	93	545	20667	4.078	0.022
7 days	GOTERM_MF_DIRECT	GO:0004672~protein kinase activity	8	8.6	1.0E-03	54101, 100526835, 4233, 2043, 90956, 4920, 51701, 7273	88	349	19208	5.003	0.016
7 days	Annotation Cluster 8	Enrichment Score: 2.8									
7 days	UP_SEQ_FEATURE	DOMAIN:Fibronectin type-III 6	4	4.3	3.5E-04	57453, 6653, 5789, 7273	92	31	20502	28.755	0.034
7 days	Annotation Cluster 9	Enrichment Score: 2.5									
7 days	INTERPRO	IPR050686:SRCR-S1_Scavenger_Rcptor	3	3.2	1.1E-03	1755, 283316, 8492	93	11	20667	60.607	0.028
7 days	UP_SEQ_FEATURE	DOMAIN:SRCR 1	3	3.2	1.5E-03	1755, 283316, 8492	92	13	20502	51.426	0.076
7 days	UP_SEQ_FEATURE	DOMAIN:SRCR 2	3	3.2	1.5E-03	1755, 283316, 8492	92	13	20502	51.426	0.076
7 days	UP_SEQ_FEATURE	DOMAIN:SRCR 3	3	3.2	1.5E-03	1755, 283316, 8492	92	13	20502	51.426	0.076
7 days	Annotation Cluster 10	Enrichment Score: 2.5									
7 days	UP_KW_DOMAIN	KW-0732~Signal	36	38.7	1.0E-03	4052, 7364, 9068, 4593, 9568, 4233, 9369, 6653, 8492, 1755, 81578, 774, 125931, 654, 976, 4588, 84465, 4920, 10631, 10531, 5979, 7450, 64386, 9777, 4684, 131873, 4585, 56241, 2043, 1293, 84628, 120114, 57453, 283316, 4851, 5789	74	4394	14566	1.613	0.005
7 days	Annotation Cluster 11	Enrichment Score: 1.7146059997000407									
7 days	INTERPRO	IPR000152:EGF-type_Asp/Asn_hydroxyl_site	5	5.4	1.2E-03	120114, 4052, 9369, 976, 4851	93	104	20667	10.684	0.029
7 days	Annotation Cluster 12	Enrichment Score: 1.5									
7 days	INTERPRO	IPR013098:Ig_I-set	6	6.5	4.5E-04	4593, 57453, 4684, 4920, 5789, 7273	93	143	20667	9.324	0.018
7 days	INTERPRO	IPR003598:Ig_sub2	7	7.5	1.1E-03	4593, 125931, 57453, 4684, 4920, 5789, 7273	93	260	20667	5.983	0.028
21 days	Annotation Cluster 1	Enrichment Score: 2.4									
21 days	UP_SEQ_FEATURE	REPEAT:ANK 9	5	5.2	3.8E-05	54101, 286, 8398, 57498, 8989	96	41	20502	26.044	0.032
